# Supplementary figures and images for: Gender differences in the impact of fatigue on lower limb landing biomechanics and their association with anterior cruciate ligament (ACL) injuries: A systematic review and meta-analysis
Source: PLoS One. 2025 May 7;20(5):e0321925. doi: 10.1371/journal.pone.0321925 (PMC12058186; doi:10.1371/journal.pone.0321925)

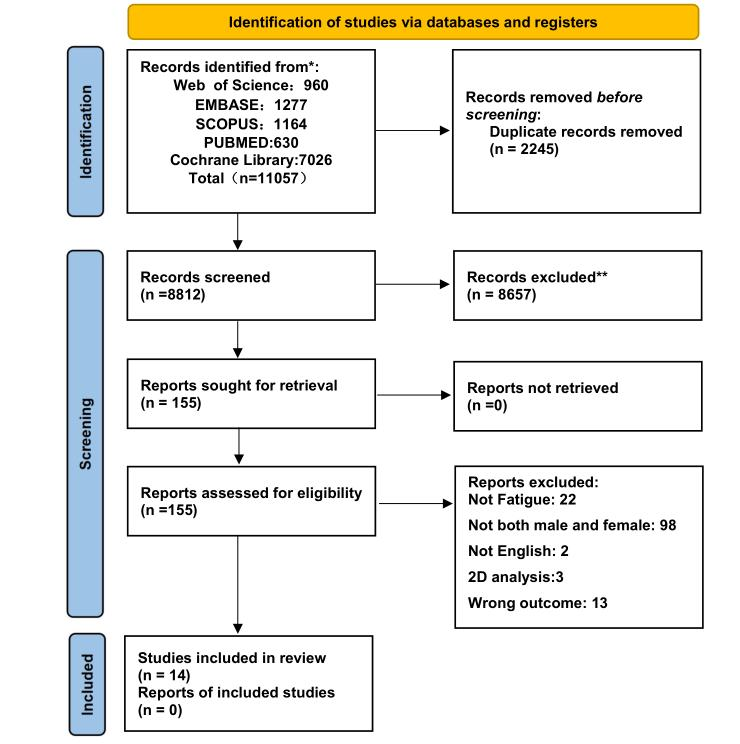

Supplement: S1 Fig — (TIF) [file pone.0321925.s001.tif]

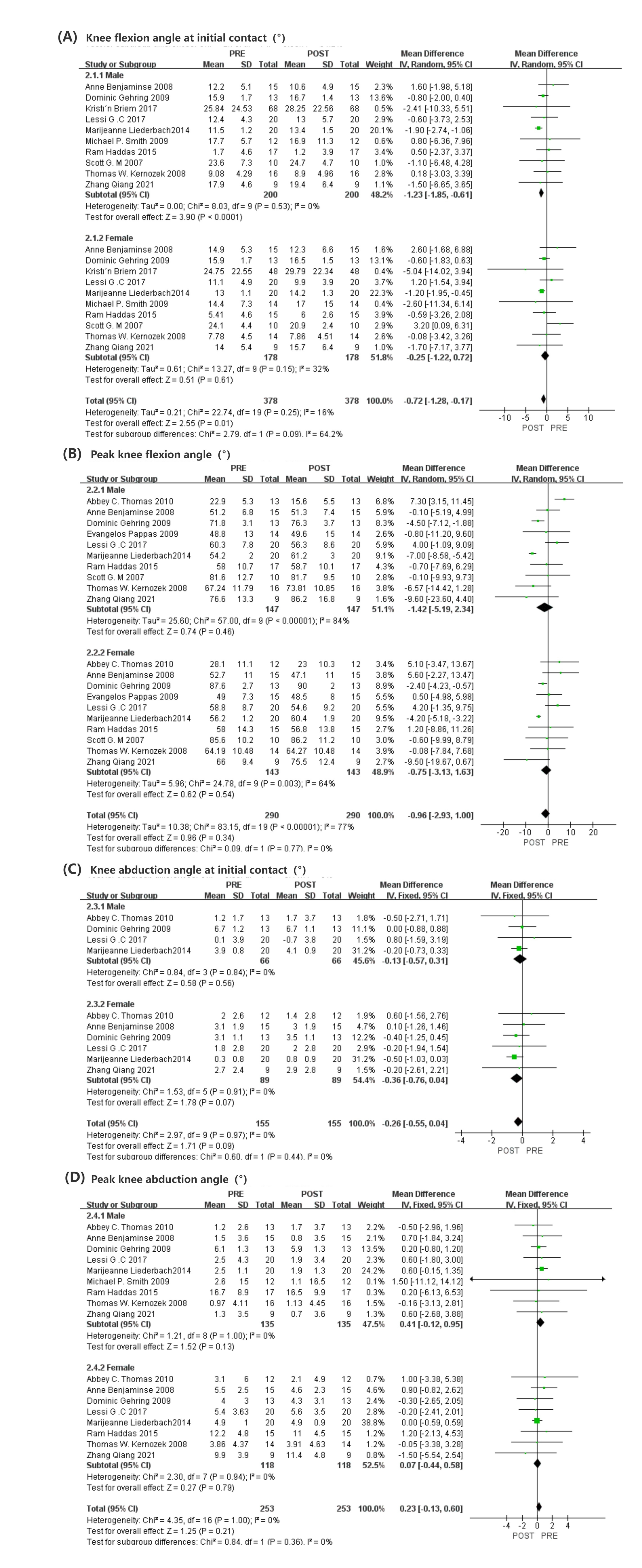

Supplement: S2 Fig — (TIF) [file pone.0321925.s002.tif]

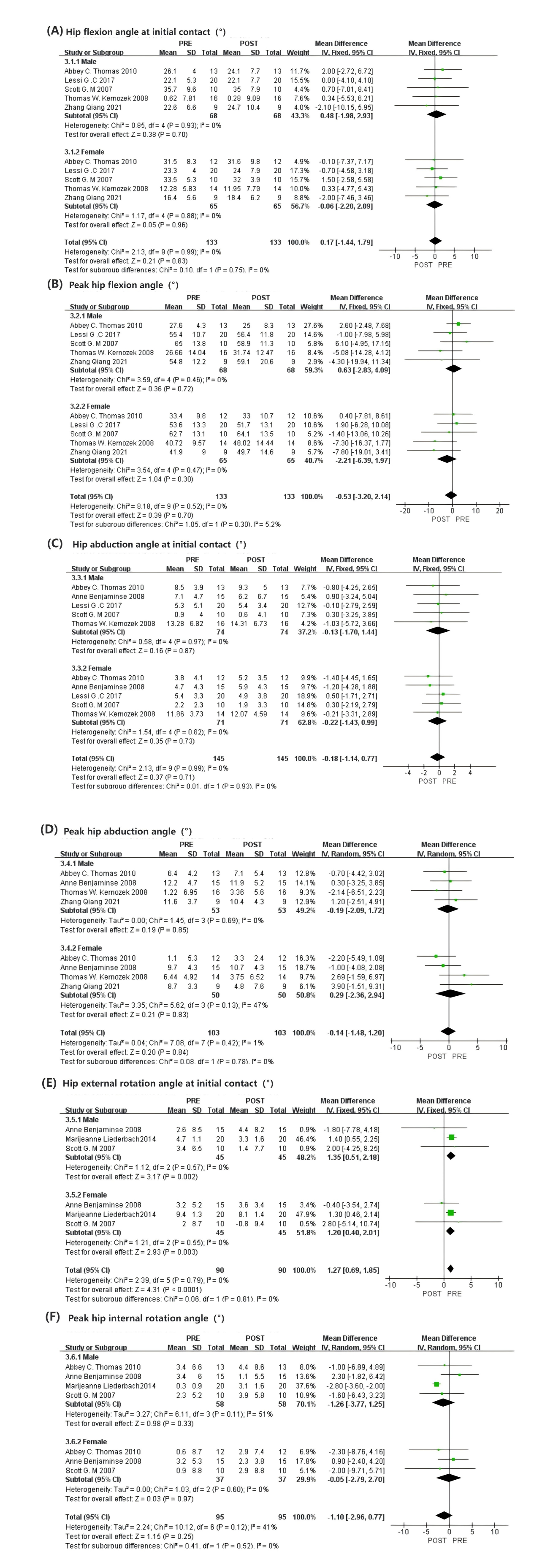

Supplement: S3 Fig — (TIF) [file pone.0321925.s003.tif]

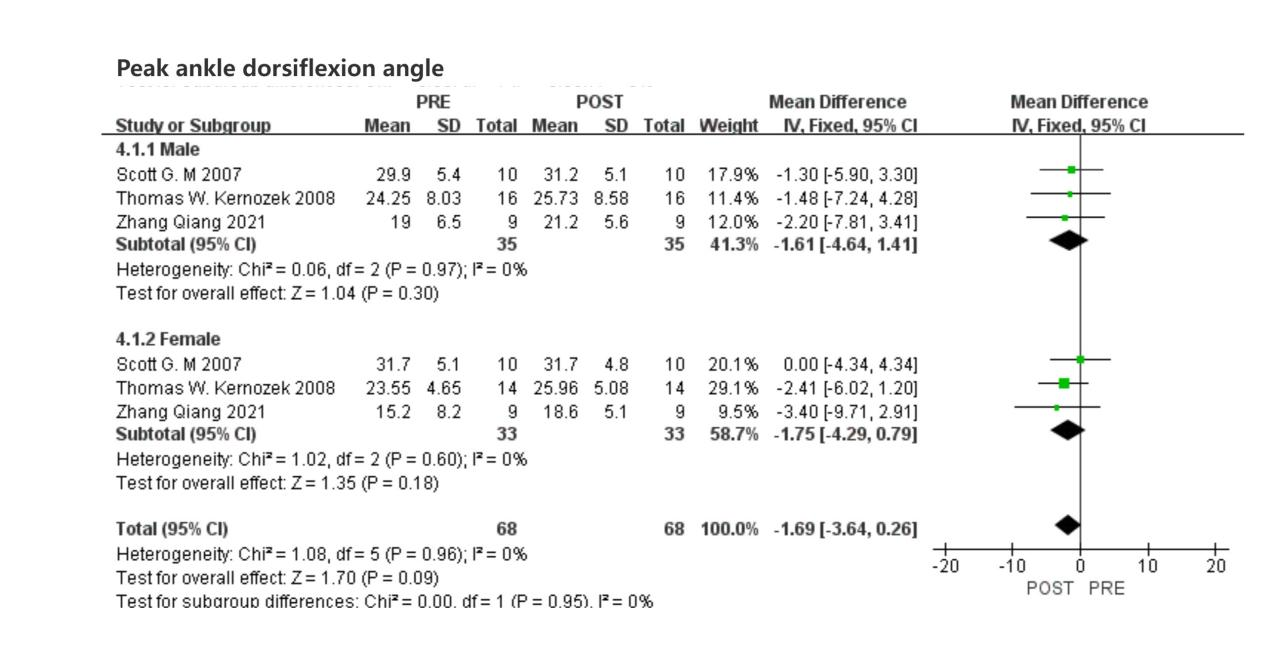

Supplement: S4 Fig — (TIF) [file pone.0321925.s004.tif]

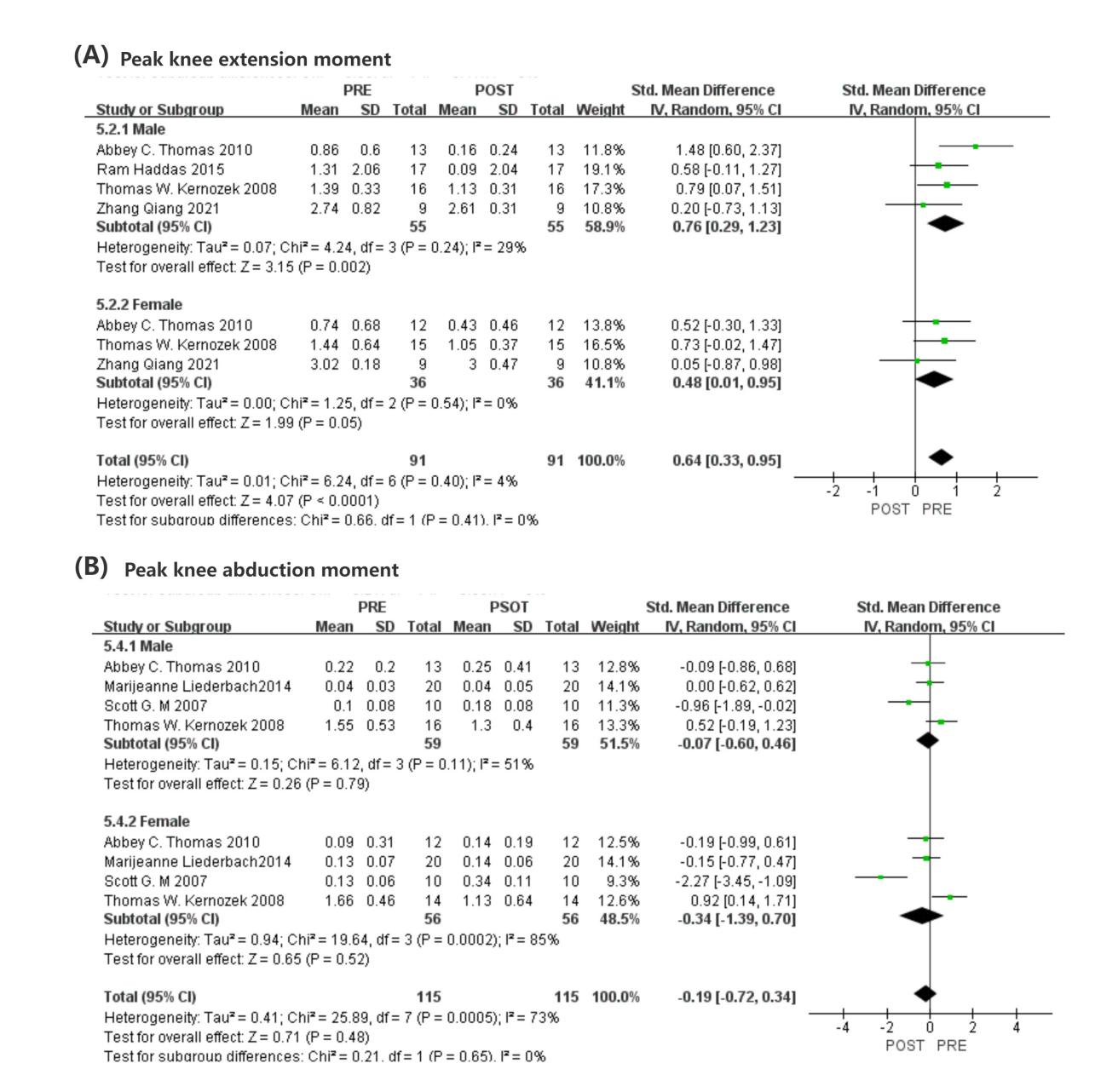

Supplement: S5 Fig — (TIF) [file pone.0321925.s005.tif]

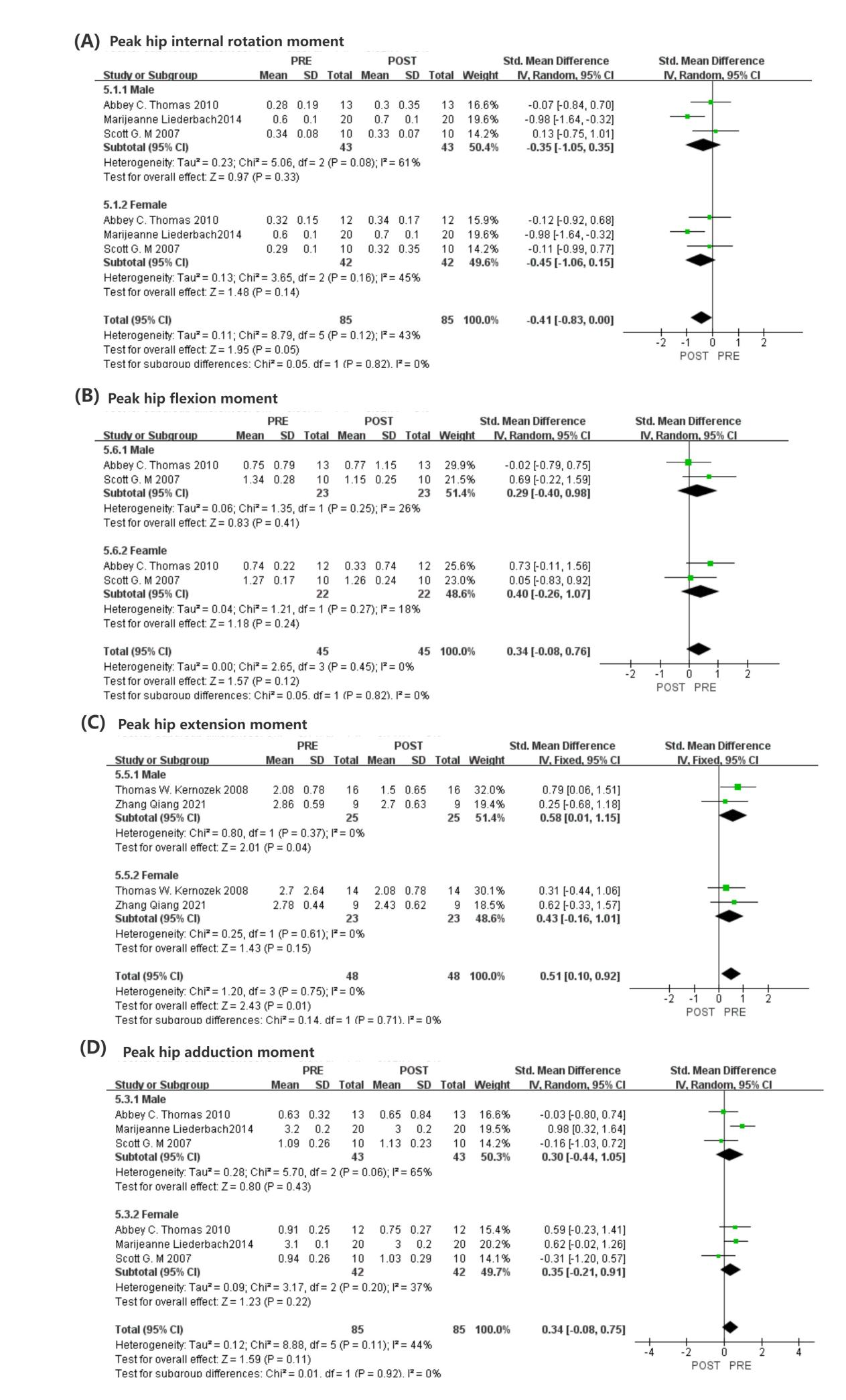

Supplement: S6 Fig — (TIF) [file pone.0321925.s006.tif]

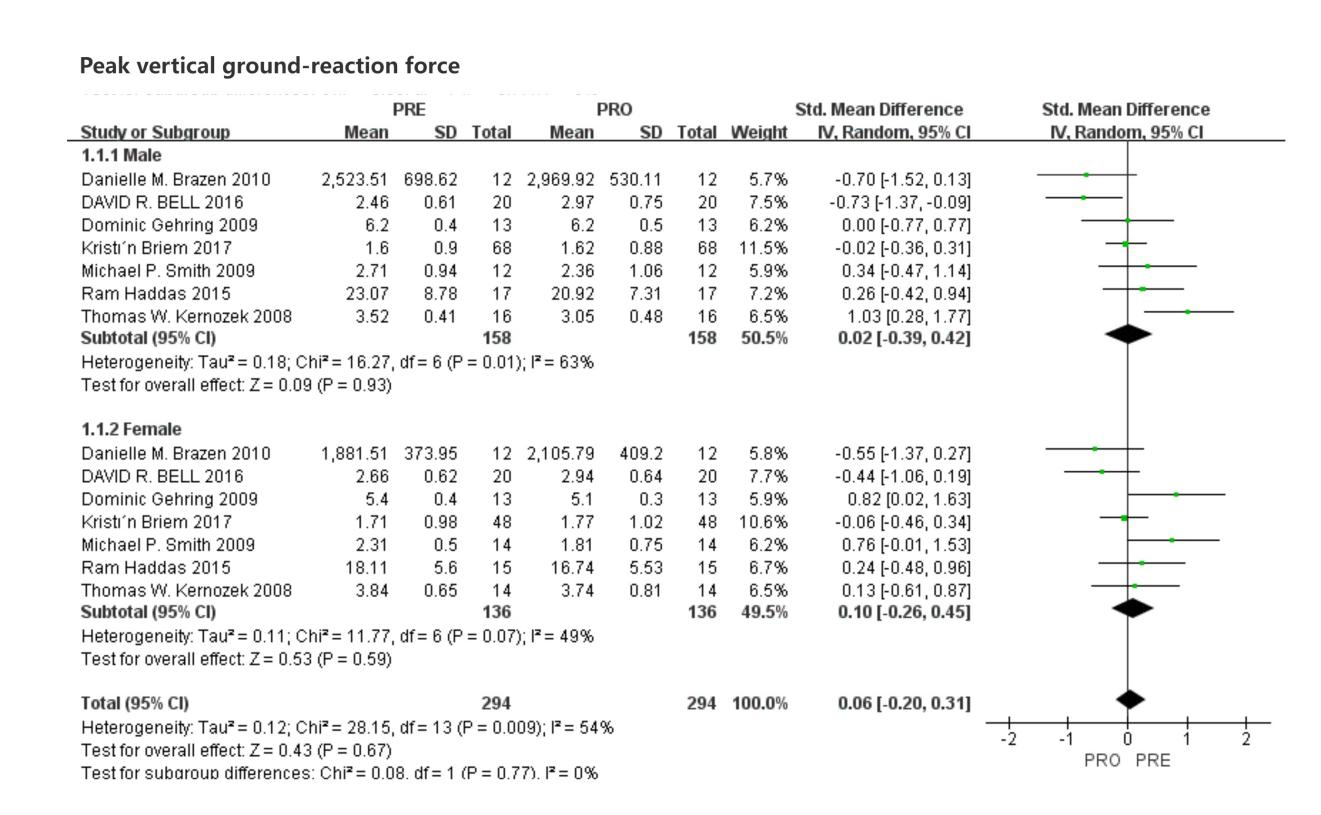

Supplement: S7 Fig — (TIF) [file pone.0321925.s007.tif]
