# Supplementary material for: Gender differences in the impact of fatigue on lower limb landing biomechanics and their association with anterior cruciate ligament (ACL) injuries: A systematic review and meta-analysis
Source: PLoS One. 2025 May 7;20(5):e0321925. doi: 10.1371/journal.pone.0321925 (PMC12058186; doi:10.1371/journal.pone.0321925)
Supplement: S1 File — (DOCX) [file pone.0321925.s008.docx]

| **Section and Topic** | **Item #** | **Checklist item** | **Location where item is reported** |
| --- | --- | --- | --- |
| **TITLE** | | |  |
| Title | 1 | Gender Differences in Lower Limb Landing Mechanics and ACL Injury Risk During Fatigue: A Systematic Review and Meta-Analysis | page 1 |
| **ABSTRACT** | | |  |
| Abstract | 2 | Background  Neuromuscular fatigue has been proposed to influence lower limb biomechanics and potentially contribute to ACL injury, with notable differences between genders.  Objective  This study aims to analyze the impact of neuromuscular fatigue on gender differences in lower limb landing biomechanics and its correlation with ACL injury prevalence through a meta-analysis.  Method  A comprehensive search was conducted in electronic databases including PubMed, Scopus, Web of Science, Embase, and the Cochrane Library up to March 2024. Studies were included if they conducted comparative experiments on adults before and after inducing fatigue, examining gender differences related to fatigue. All references were managed with EndNote X9, duplicates were removed, and two authors independently extracted and confirmed study details, resolving discrepancies with a third researcher.  Result Fourteen studies met the inclusion criteria, with an average quality score of 6.79; nine were rated as high quality. Key findings include:  1.Males showed a significant increase in knee flexion angle at initial contact (effect size -1.23), while females did not (-0.25).  2.Significant changes in hip external rotation were observed in both genders (males: 1.35, females: 1.20).  3.Ankle peak dorsiflexion angle increased (-1.69) with no gender differences.  4.Peak Knee extension moment increased in males (0.76) and females (0.48), with an overall effect size of 0.64, but no change in peak abduction moment.  5.Peak Hip extension moment was significant in males (0.58) and overall (0.51), with no changes in internal rotation or adduction moments.  Peak vertical ground reaction force showed no significant changes for either gender.  Conclusion  Fatigue increases knee flexion angle and decreases knee extension moment in males, raising injury risk. Both genders show decreased hip external rotation angle post-fatigue, increasing knee and ankle load. Other hip moments, ankle dorsiflexion angle, and peak vertical ground reaction force are unchanged. These findings highlight the need for gender-specific fatigue management and exercise strategies to reduce ACL injury risk, suggesting further research into underlying mechanisms and interventions. | page 2 |
| **INTRODUCTION** | | |  |
| Rationale | 3 | Anterior Cruciate Ligament (ACL) tears are common non-contact injuries in sports that involve rapid direction changes, deceleration, jumping, and landing. These injuries typically result from improper landing techniques, which place excessive stress on the ACL. Key biomechanical factors linked to ACL injuries include increased knee abduction angles, knee abduction moments, and hip internal rotation moments during landing.  Fatigue is a critical factor affecting lower limb biomechanics and increasing the risk of ACL injuries. It affects both central and peripheral neuromuscular pathways, leading to reduced neural feedback, slower muscle reaction times, and proprioceptive dysfunction. These changes impair joint stability and dynamic balance control, increasing knee joint loads and the risk of ACL injuries. Studies show that fatigue decreases knee flexion angles, increases knee abduction angles, and heightens hip internal rotation angles, significantly raising stress on the knee joint.  Research has shown gender differences in lower limb landing mechanics, with females typically exhibiting smaller knee flexion angles and larger knee valgus angles, leading to higher ACL injury rates. However, findings on gender differences under fatigue conditions are inconsistent, indicating the need for more systematic research. | page 3-4 |
| Objectives | 4 | This study aims to systematically review and meta-analyze the impact of neuromuscular fatigue on gender differences in lower limb landing biomechanics and its correlation with ACL injury prevalence. | page 5 |
| **METHODS** | | |  |
| Eligibility criteria | 5 | The literature screening criteria for this meta-analysis were established according to the PICOS[27]format (Participants, Interventions, Comparisons, Outcomes, and Study Design) used in evidence-based medicine. The inclusion criteria are: (1) studies involving both male and female participants; (2) studies utilizing a pre-post self-controlled experimental design; (3) studies implementing at least one protocol designed to induce fatigue; and (4) studies with outcomes that include kinematic, kinetic, or electromyographic analyses relevant to sports biomechanics. The exclusion criteria are: (1) studies not employing a self-controlled experimental design; (2) studies with inaccessible data; (3) studies not utilizing three-dimensional motion analysis systems or force platforms for kinematic and kinetic measurements; (4) conference papers, review articles, and opinion pieces; and (5) studies where the full text is not available. | page 7 |
| Information sources | 6 | As of March 2024, a comprehensive search was conducted in the electronic databases of PubMed, Scopus, Web of Science, Embase, and Cochrane Library. | page 6 |
| Search strategy | 7 | #1.“fatigue”[Title/Abstract]OR“tiredness”[Title/Abstract]OR“weariness”[Title/Abstract]OR “exhaustion”[Title/Abstract]OR “induced fatigue”[Title/Abstract] OR “fatigue damage”[Title/Abstract] OR “neuromuscular fatigue”[Title/Abstract]  #2.  “sexuality”[Title/Abstract]OR“sex”[Title/Abstract]OR“gender”[Title/Abstract]  #3.  “landing biomechanics”[Title/Abstract] OR “kinematics”[Title/Abstract] OR “kinetics”[Title/Abstract] OR “landing”[Title/Abstract] OR “Biomechanics”[Title/Abstract] OR “lower extremity”[Title/Abstract] OR “hip” [Title/Abstract]OR “knee”[Title/Abstract] OR “ankle”[Title/Abstract]  #4 #1 AND #2 AND #3 | page 6 |
| Selection process | 8 | Conduct a comparative experiment on adults before and after inducing fatigue, examining gender differences related to fatigue. | page 7-8 |
| Data collection process | 9 | The systematic search was carried out by three independent researchers (C.L., W.P., W.Q.). They initially screened the articles based on titles and abstracts. If the information was ambiguous, they retrieved the full text to assess relevance. After the screening process, the researchers compared their findings and resolved disagreements by jointly reassessing the studies that met the eligibility criteria. | page 6 |
| Data items | 10a | All references were managed using EndNote X9 software, and duplicates were removed. Two authors (C.L and L.X) independently extracted descriptive information from all included studies, including publication details (author, year), participant demographics (age, gender), sample size, participant characteristics, fatigue intervention protocols, implementation methods, and biomechanical outcomes (kinematics, kinetics) . Biomechanical data necessary for calculating effect sizes (mean and standard deviation) were extracted, and corresponding authors were contacted for additional data if needed. Any discrepancies in the data extracted by C.L and L.X were confirmed by a third researcher, W.P. | page 7-8 |
| Study risk of bias assessment | 11 | The quality of the studies was assessed using a modified version of the Strengthening the Reporting of Observational Studies in Epidemiology (STROBE) guidelines for evaluating observational studies[28] . Each included study was scored based on 10 specific criteria derived from items 5, 6, 7, 8, 9, 12, 13, 14, and 15 of the original checklist (Table 1). The overall quality score was the sum of these criteria, with a maximum score of 10 points. Studies scoring 7 points or higher were considered high quality. This scoring threshold was determined by author consensus and based on previously published modifications of the STROBE guidelines[29]. | page 8 |
| Effect measures | 12 | effect size | page 10 |
| Synthesis methods | 13d | To ensure objectivity and robustness, Review Manager 5.3 software (The Nordic Cochrane Centre, Copenhagen, Denmark) was utilized for meta-analyses and subgroup analyses of data from at least two studies reporting on the same variable for both males and females. When measurement units were consistent (e.g., angles), the weighted mean difference (WMD) with a 95% confidence interval was used to assess the overall effect size. If the measurement units differed, the standardized mean difference (SMD) was applied[30]. Heterogeneity among studies was evaluated using the I² statistic, categorized as low (25-50%), moderate (50-75%), and high (greater than 75%)[31]. A fixed-effects model was used when heterogeneity was less than 25%, and a random-effects model was applied when it exceeded 25%[32]. A significance level of 0.05 was employed for all statistical tests. | page 10-11 |
| **RESULTS** | | |  |
| Study selection | 16a | Through electronic database searches, 11,057 records were identified. After the initial screening, 2,245 duplicate records were removed. Titles and abstracts of the remaining 8,812 records were screened, resulting in the exclusion of 8,657 records. A detailed evaluation of the 155 remaining full-text articles led to the exclusion of 136 based on study content and quality: 22 did not involve fatigue, 98 did not include both genders, 2 were non-English articles, 3 used 2D analysis methods, and 13 did not meet study requirements. After rigorous screening, 17 studies were included in the qualitative synthesis, and 14 were used in the quantitative synthesis | page 11 |
| Study characteristics | 17 | This meta-analysis included 14 studies involving different populations: six studies with healthy individuals, three with amateur athletes, two with collegiate athletes, and three with registered athletes, team athletes, and young individuals with a history of recurrent low back pain but currently asymptomatic. These studies encompassed a total of 496 participants, with 259 males and 237 females. Six studies used single-leg landing, while eight used double-leg landing. Fatigue induction protocols were categorized into isolated muscle group fatigue and whole-body fatigue. The included studies provided extensive kinematic and kinetic results, covering peak flexion angles of the knee, hip, and ankle joints; initial contact flexion angles of the hip and knee joints; peak abduction angles of the knee and hip joints and their angles at initial contact; hip external rotation angle at initial contact and peak internal rotation angle; as well as peak internal adduction and internal rotation moments of the hip; peak extension and abduction moments of the knee joint; and peak vertical ground reaction force (PVGRF) | page 12 |
| Risk of bias in studies | 18 | Table 2 details the quality scores of the included studies. Each column corresponds to a specific quality assessment criterion (N1 to N10), with each criterion scored out of 1 point, totaling a maximum of 10 points per study. The table lists the author names, their scores for each criterion, and the total score. Most studies scored between 5 and 8 out of 10, with an average score of 6.79. Nine out of the 14 studies were identified as "high quality," scoring 7 or higher. | page 12 |
| Results of individual studies | 19 | This meta-analysis included a total of 14 studies that examined the kinematic data of the knee, hip, and ankle joints, investigating the effects of fatigue and gender on these parameters. The kinematic data for each joint were represented by three-dimensional joint angles (in degrees). The specific parameters analyzed are detailed in Table 4. | page 26 |
| Results of syntheses | 20b | Kinematic Data of Landing Biomechanics  This meta-analysis included a total of 14 studies that examined the kinematic data of the knee, hip, and ankle joints, investigating the effects of fatigue and gender on these parameters. The kinematic data for each joint were represented by three-dimensional joint angles (in degrees). The specific parameters analyzed are detailed in Table 4.  Kinetics Data of Landing Biomechanics  The dynamic data for the hip and knee joints, as well as peak vertical ground reaction force (PVGRF), were evaluated. | page 26-40 |
| **DISCUSSION** | | |  |
| Discussion | 23a | This meta-analysis systematically reviewed existing literature to understand how fatigue influences gender differences in landing biomechanics and the associated risk of ACL injuries. The results indicate that fatigue significantly affects the kinematics and kinetics of the knee, hip, and ankle joints. However, most gender differences in these effects were not statistically significant, with only a few variables showing notable changes. Specifically, fatigue significantly increased the knee flexion angle at initial contact in males but not in females. Additionally, fatigue led to a significant reduction in hip external rotation angles at initial contact for both genders. In terms of kinetics, fatigue significantly decreased the maximum extension torque in the knee and hip joints of males, while the effect in females was nearly significant. This discussion will delve into our key findings, focusing on the landing biomechanics of the knee, hip, and ankle. | page 48 |
|  | 23b | There are several limitations in this systematic review that need to be considered. First, although 14 studies were included, only a few reported ankle joint biomechanical variables, limiting the scope of our analysis. Second, the review focused solely on lower limb biomechanical indicators without considering the positioning and control of the trunk and the entire kinetic chain, which may significantly impact knee joint biomechanics. This limitation might result in an incomplete understanding of knee joint biomechanics. Finally, although the review included data from both sexes before and after fatigue, studies focusing on only one gender were not included, potentially affecting the comprehensive understanding of gender differences. | page 53 |
|  | 23d | This suggests that future research on ACL injury mechanisms should focus more on the dynamic performance of the hip joint and its coordination with other lower limb joints. | page 53 |
| **OTHER INFORMATION** | | |  |
| Registration and protocol | 24a | The protocol has been registered on PROSPERO (ID: CRD42024545104).Registered person: Chengxun Liu |  |
|  | 24b | No |  |
|  | 24c | No |  |
| Support | 25 | No |  |
| Competing interests | 26 | The authors declare no competing interests. |  |

*From:*  Page MJ, McKenzie JE, Bossuyt PM, Boutron I, Hoffmann TC, Mulrow CD, et al. The PRISMA 2020 statement: an updated guideline for reporting systematic reviews. BMJ 2021;372:n71. doi: 10.1136/bmj.n71
