# Supplementary material for: Gender differences in the impact of fatigue on lower limb landing biomechanics and their association with anterior cruciate ligament (ACL) injuries: A systematic review and meta-analysis
Source: PLoS One. 2025 May 7;20(5):e0321925. doi: 10.1371/journal.pone.0321925 (PMC12058186; doi:10.1371/journal.pone.0321925)
Supplement: S2 File — (DOCX) [file pone.0321925.s009.docx]

| **S/N** | **Author/s(reference** | **Title** | **DOI/URL** | **Included OR Excluded** | **Reasons for exclusion** |
| --- | --- | --- | --- | --- | --- |
| **1** | **Abbey C. Thomas et al.** | **Quadriceps and hamstrings fatigue alters hip and knee mechanics.** | **[10.1123/jab.26.2.159](https://doi.org/10.1123/jab.26.2.159)** | **Included** |  |
| **2** | **Anne Benjaminse et al.** | **Fatigue alters lower extremity kinematics during a single-leg stop-jump task** | **10.1007/s00167-007-0432-7** | **Included** |  |
| **3** | **Danielle M. Brazenet al.** | **The effect of fatigue on landing biomechanics in single-leg drop landings.** | **10.1097/JSM.0b013e3181e8f7dc** | **Included** |  |
| **4** | **DAVID R. BELL et al.** | **The effect of exertion and sex on vertical ground reaction force variables and landing mechanics.** | **10.1519/JSC.0000000000001310** | **Included** |  |
| **5** | **Dominic Gehring et al.** | **Gender and fatigue have influence on knee joint control strategies during landing.** | **10.1016/j.clinbiomech.2008.10.009** | **Included** |  |
| **6** | **Evangelos Pappas et al.** | **Peak biomechanical variables during bilateral drop landings: comparisons between sex (female/male) and fatigue (pre-fatigue/post-fatigue).** | **10.4085/1062-6050-44.5.565** | **Included** |  |
| **7** | **Kristı´n Briem et al.** | **Effects of Sex and Fatigue on Biomechanical Measures During the Drop-Jump Task in Children.** | **10.1177/2325967116679640** | **Included** |  |
| **8** | **Lessi G .C et al.** | **Effects of fatigue on lower limb, pelvis and trunk kinematics and muscle activation: Gender differences.** | **10.1016/j.jbiomech.2016.11.051** | **Included** |  |
| **9** | **Marijeanne Liederbach et al.** | **Comparison of landing biomechanics between male and female dancers and athletes, part 2: Influence of fatigue and implications for anterior cruciate ligament injury.** | **10.1177/0363546514524525** | **Included** |  |
| **10** | **Michael P. Smith et al.** | **Effects of fatigue on frontal plane knee motion, muscle activity, and ground reaction forces in men and women during landing.** | **10.1177/1941738109334213** | **Included** |  |
| **11** | **Ram Haddas et al.** | **Lower extremity fatigue, sex, and landing performance in a population with recurrent low back pain.** | **10.4085/1062-6050-50.3.01** | **Included** |  |
| **12** | **Scott G. M et al.** | **Impact of fatigue on gender-based high-risk landing strategies.** | **10.1249/mss.0b013e3180d47f0** | **Included** |  |
| **13** | **Thomas W. Kernozek et al.** | **Gender differences in lower extremity landing mechanics caused by neuromuscular fatigue.** | **10.1177/0363546507308934** | **Included** |  |
| **14** | **Zhang Qiang et al.** | **Progression of Fatigue Modifies Primary Contributors to Ground Reaction Forces During Drop Landing.** | **10.3390/ijerph18020580** | **Included** |  |
| **15** | **Boozari S.et al.** | **Fatigue effects on the viscoelastic behavior of men and women in a landing task: a Mass–Spring–Damper modeling approach** | **10.1080/10255842.2020.1749271** | **Excluded** | **Wrong outcome** |
| **16** | **Ford, K. R.et al.** | **Longitudinal sex differences during landing in knee abduction in young athletes** | **10.1249/MSS.0b013e3181dc99b1** | **Excluded** | **Not Fatigue** |
| **17** | **Petrovic, M.et al.** | **Effect of Sex on Anterior Cruciate Ligament Injury–Related Biomechanics During the Cutting Maneuver in Preadolescent Athletes** | **10.1177/2325967120936980** | **Excluded** | **Wrong outcome** |
| **18** | **Abergel, R. E.et al.** | **The effects of acute physical fatigue on sauté jump biomechanics in dancers** | **10.1080/02640414.2020.1854425** | **Excluded** | **Not both male and female** |
| **19** |
| **20** |
| **21** | **Alanazi, A. D.et al.** | **The effects of a high-intensity exercise bout on landing biomechanics post anterior cruciate ligament reconstruction: a quasi-experimental study** | **10.1186/s13102-021-00263-7** | **Excluded** | **Not both male and female** |
| **22** | **Augustsson, J.et al.** | **Single-leg hop testing following fatiguing exercise: reliability and biomechanical analysis** | **10.1111/j.1600-0838.2005.00446.x** | **Excluded** | **Not both male and female** |
| **23** | **Chang, E. W.et al.** | **Landing biomechanics in anterior cruciate ligament reconstructed females who pass or fail a functional test battery** | **10.1016/j.knee.2018.09.006** | **Excluded** | **Not both male and female** |
| **24** | **Cone, J. R.et al.** | **Effects of an individualized soccer match simulation on vertical stiffness and impedance** | **10.1519/JSC.0b013e31823a4076** | **Excluded** | **Wrong outcome** |
| **25** | **Cortes, N.et al.** | **A functional agility short-term fatigue protocol changes lower extremity mechanics** | **10.1080/02640414.2012.671528** | **Excluded** | **Not both male and female** |
| **26** | **Coventry, E.et al.** | **The effect of lower extremity fatigue on shock attenuation during single-leg landing** | **10.1016/j.clinbiomech.2006.07.004** | **Excluded** | **Not both male and female** |
| **27** | **Edwards, S.et al.** | **Does a drop landing represent a whole skill landing and is this moderated by fatigue?** | **10.1111/j.1600-0838.2009.00964.x** | **Excluded** | **Not both male and female** |
| **28** | **Edwards, S.et al.** | **Alterations to landing technique and patellar tendon loading in response to fatigue** | **10.1249/MSS.0b013e3182a42e8e** | **Excluded** | **Not both male and female** |
| **29** | **Encarnación-Martínez, A.et al.** | **Effect of Hamstring Tightness and Fatigue on Dynamic Stability and Agility in Physically Active Young Men** | **10.3390/s23031633** | **Excluded** | **Not both male and female** |
| **30** |
| **31** |
| **32** | **Encarnación-Martínez, A.et al.** | **Relationship between muscular extensibility, strength and stability and the transmission of impacts during fatigued running** | **10.1080/14763141.2020.1797863** | **Excluded** | **Not both male and female** |
| **33** | **Fagenbaum, R.et al.** | **Jump landing strategies in male and female college athletes and the implications of such strategies for anterior cruciate ligament injury** | **10.1177/03635465030310021301** | **Excluded** | **Wrong outcome** |
| **34** | **Fort-Vanmeerhaeghe, A.et al.** | **Sex and Maturation Differences in Performance of Functional Jumping and Landing Deficits in Youth Athletes** | **10.1123/jsr.2017-0292** | **Excluded** | **Wrong outcome** |
| **35** | **Frank, B. S.et al.** | **Neuromuscular fatigue alters postural control and sagittal plane hip biomechanics in active females with anterior cruciate ligament reconstruction** | **10.1177/1941738114530950** | **Excluded** | **Not both male and female** |
| **36** | **Gafner, S. C.et al.** | **Hip-abductor fatigue influences sagittal plane ankle kinematics and shank muscle activity during a single-leg forward jump** | **10.1016/j.jelekin.2018.09.004** | **Excluded** | **Not Fatigue** |
| **37** | **Gao, Z.et al.** | **Continuous time series analysis on the effects of induced running fatigue on leg symmetry using kinematics and kinetic variables: Implications for knee joint injury during a countermovement jump** | **10.3389/fphys.2022.877394** | **Excluded** | **Wrong outcome** |
| **38** | **Giandolini, M.et al.** | **Effect of the Fatigue Induced by a 110-km Ultramarathon on Tibial Impact Acceleration and Lower Leg Kinematics** | **10.1371/journal.pone.0151687** | **Excluded** | **Wrong outcome** |
| **39** | **Goethel, M. F.et al.** | **A global view on how local muscular fatigue affects human performance** | **10.1073/pnas.2007579117** | **Excluded** | **Wrong outcome** |
| **40** |
| **41** |
| **42** | **Haddas, R.et al.** | **Effects of Volitional Spine Stabilization and Lower-Extremity Fatigue on the Knee and Ankle During Landing Performance in a Population With Recurrent Low Back Pain** | **10.1123/jsr.2015-0171** | **Excluded** | **Not both male and female** |
| **43** | **Harato, K.et al.** | **Fatigue and recovery have different effects on knee biomechanics of drop vertical jump between female collegiate and recreational athletes** | **10.1186/s13018-021-02893-6** | **Excluded** | **Not both male and female** |
| **44** |
| **45** |
| **46** | **Herbaut, A.et al.** | **Fatigue increases ankle sprain risk in badminton players: A biomechanical study** | **10.1080/02640414.2020.1748337** | **Excluded** | **Not both male and female** |
| **47** |
| **48** |
| **49** | **Higo, Y.et al.** | **Effects of Lower-limb Muscle Fatigue, Cardiopulmonary Fatigue, and Brain FatigueTasks on One-legged Landing Motion** | **10.1298/ptr.E10104** | **Excluded** | **Not both male and female** |
| **50** |
| **51** |
| **52** | **Howe, L.et al.** | **Restrictions in Ankle Dorsiflexion Range of Motion Alter Landing Kinematics But Not Movement Strategy When Fatigued** | **10.1123/jsr.2020-0429** | **Excluded** | **2D analysis** |
| **53** | **Jacobs, C. A.et al.** | **Hip abductor function and lower extremity landing kinematics: sex differences** | **https://pubmed.ncbi.nlm.nih.gov/17597947/** | **Excluded** | **Not Fatigue** |
| **54** |
| **55** |
| **56** | **Jalalvand, A.et al.** | **Effect of Lower Limb Muscle Fatigue on Ground Reaction Force Components During Landing in People With Nonspecific Chronic Low Back Pain** | **10.1123/jsr.2018-0153** | **Excluded** | **Not both male and female** |
| **57** |
| **58** |
| **59** | **James, C. R.et al.** | **Effects of stretch shortening cycle exercise fatigue on stress fracture injury risk during landing** | **10.1080/02701367.2006.10599346** | **Excluded** | **Not both male and female** |
| **60** | **James, C. R.et al.** | **Effects of two neuromuscular fatigue protocols on landing performance** | **10.1016/j.jelekin.2009.10.007** | **Excluded** | **Not both male and female** |
| **61** |
| **62** |
| **63** | **Kamelska, A. M.et al.** | **The effect of motor learning and fatigue on preactivation of the lower extremity muscles during different jumps** | **10.23736/s0022-4707.17.07712-x** | **Excluded** | **Not both male and female** |
| **64** | **Kamitani, A.et al.** | **Landing Posture in Elite Female Athletes During a Drop Vertical Jump Before and After a High-Intensity Ergometer Fatigue Protocol: A Study of 20 Japanese Women's Soccer League Players** | **10.1177/23259671231171859** | **Excluded** | **Not both male and female** |
| **65** | **Kellis, E.et al.** | **Agonist versus antagonist muscle fatigue effects on thigh muscle activity and vertical ground reaction during drop landing** | **10.1016/j.jelekin.2007.08.002** | **Excluded** | **Not both male and female** |
| **66** | **Kim, H.et al.** | **Functional Fatigue Alters Lower-extremity Neuromechanics during a Forward-side Jump** | **10.1055/s-0035-1550050** | **Excluded** | **Not both male and female** |
| **67** | **Kim, N.et al.** | **Effect of isolated hip abductor fatigue on single-leg landing mechanics and simulated ACL loading** | **10.1016/j.knee.2021.05.007** | **Excluded** | **Not both male and female** |
| **68** | **Kim, Y.et al.** | **The effect of knee flexor and extensor fatigue on shock absorption during cutting movements after a jump landing** | **10.1016/j.knee.2017.09.007** | **Excluded** | **Not both male and female** |
| **69** | **Klein, C. J. D.et al.** | **Sex-Based Differences in Lower Extremity Kinematics During Dynamic Jump Landing Tasks After Neuromuscular Fatigue of the Hip Extensors and Knee Flexors** | **10.1177/23259671231215848** | **Excluded** | **2D analysis** |
| **70** | **Knihs, D. A.et al.** | **Acute and Delayed Effects of Fatigue on Ground Reaction Force, Lower Limb Stiffness and Coordination Asymmetries During a Landing Task** | **10.2478/hukin-2021-0054** | **Excluded** | **Not both male and female** |
| **71** |
| **72** |
| **73** | **Lee, M.et al.** | **Effects of chronic ankle instability and induced mediolateral muscular fatigue of the ankle on competitive taekwondo athletes** | **10.1589/jpts.29.1329** | **Excluded** | **Not both male and female** |
| **74** | **Lee, S. P.et al.** | **Fatigue of the hip abductors results in increased medial-lateral center of pressure excursion and altered peroneus longus activation during a unipedal landing task** | **10.1016/j.clinbiomech.2013.04.002** | **Excluded** | **Not both male and female** |
| **75** | **Liu, Y.et al.** | **Effects of fatigue on balance and ankle proprioception during drop landing among individuals with and without chronic ankle instability** | **10.1016/j.jbiomech.2022.111431** | **Excluded** | **Not both male and female** |
| **76** | **Liveris, N. I.et al.** | **Evaluating the Effects of Match-Induced Fatigue on Landing Ability; the Case of the Basketball Game** |  | **Excluded** | **Wrong outcome** |
| **77** | **Lucci, S.et al.** | **Knee and hip sagittal and transverse plane changes after two fatigue protocols** | **10.1016/j.jsams.2011.05.001** | **Excluded** | **Not both male and female** |
| **78** | **Madigan, M. L.et al.** | **Changes in landing biomechanics during a fatiguing landing activity** | **10.1016/s1050-6411(03)00037-3** | **Excluded** | **Not both male and female** |
| **79** | **McLean, S. G.et al.** | **Fatigue-induced ACL injury risk stems from a degradation in central control** | **10.1249/MSS.0b013e31819ca07b** | **Excluded** | **Wrong outcome** |
| **80** | **Mejane, J.et al.** | **The combined impact of a perceptual-cognitive task and neuromuscular fatigue on knee biomechanics during landing** | **10.1016/j.knee.2018.10.017** | **Excluded** | **Not both male and female** |
| **81** | **Miralles-Iborra, Aet al. .** | **Influence of a football match on landing biomechanics and jump performance in female football players** | **10.1111/sms.14518** | **Excluded** | **Not both male and female** |
| **82** |
| **83** |
| **84** | **Moran, K. A.et al.** | **Effect of fatigue on tibial impact accelerations and knee kinematics in drop jumps** | **10.1249/01.mss.0000229567.09661.20** | **Excluded** | **Not both male and female** |
| **85** |
| **86** |
| **87** | **Noh, B.et al.** | **Effect of knee extensor fatigue level and sex on bilateral jump-landing** | **10.1136/bmjsem-2019-000660** | **Excluded** | **Wrong outcome** |
| **88** | **Nyland, J. A.et al.** | **Fatigue after eccentric quadriceps femoris work produces earlier gastrocnemius and delayed quadriceps femoris activation during crossover cutting among normal athletic women** | **10.1007/s001670050045** | **Excluded** | **Not both male and female** |
| **89** | **Nyland, J. A.et al.** | **Relationship of fatigued run and rapid stop to ground reaction forces, lower extremity kinematics, and muscle activation** | **10.2519/jospt.1994.20.3.132** | **Excluded** | **Not both male and female** |
| **90** | **O'Connor, K. M.et al.** | **The Effect of Isolated Hamstrings Fatigue on Landing and Cutting Mechanics** | **10.1123/jab.2014-0098** | **Excluded** | **Not both male and female** |
| **91** | **Olbrantz, C.et al.** | **Effect of Posttrial Visual Feedback and Fatigue During Drop Landings on Patellofemoral Joint Stress in Healthy Female Adults** | **10.1123/jab.2017-0074** | **Excluded** | **Not both male and female** |
| **92** | **Orishimo, K. F.et al.** | **Effect of fatigue on single-leg hop landing biomechanics** | **10.1123/jab.22.4.245** | **Excluded** | **Not both male and female** |
| **93** | **Orishimo, K. F.et al.** | **Adaptations in single-leg hop biomechanics following anterior cruciate ligament reconstruction** | **10.1007/s00167-010-1185-2** | **Excluded** | **Not both male and female** |
| **94** | **Patrek, M. F.et al.** | **Hip-abductor fatigue and single-leg landing mechanics in women athletes** | **10.4085/1062-6050-46.1.31** | **Excluded** | **Not both male and female** |
| **95** | **Peng, H. T.et al.** | **Influences of Patellofemoral Pain and Fatigue in Female Dancers during Ballet Jump-Landing** | **10.1055/s-0035-1547220** | **Excluded** | **Not both male and female** |
| **96** | **Quammen, D.et al.** | **Two different fatigue protocols and lower extremity motion patterns during a stop-jump task** | **10.4085/1062-6050-47.1.32** | **Excluded** | **Not both male and female** |
| **97** | **Radcliffe, C. R.et al.** | **The effect of fatigue on peak Achilles tendon force in Irish dancing-specific landing tasks** | **10.1080/14763141.2021.1951826** | **Excluded** | **Not both male and female** |
| **98** | **Shultz, S. J.et al.** | **Changes in fatigue, multiplanar knee laxity, and landing biomechanics during intermittent exercise** | **10.4085/1062-6050-49.5.08** | **Excluded** | **Not both male and female** |
| **99** | **Smeets, A.et al.** | **Match Play-induced Changes in Landing Biomechanics with Special Focus on Fatigability** | **10.1249/mss.0000000000001998** | **Excluded** | **Not both male and female** |
| **100** | **Snyder, B. J.et al.** | **Effects of Two Competitive Soccer Matches on Landing Biomechanics in Female Division I Soccer Players** | **10.3390/sports7110237** | **Excluded** | **Not both male and female** |
| **101** | **Tamura, A.et al.** | **Fatigue influences lower extremity angular velocities during a single-leg drop vertical jump** | **10.1589/jpts.29.498** | **Excluded** | **Not both male and female** |
| **102** |
| **103** |
| **104** | **Thomas, A. C.et al.** | **Effects of Neuromuscular Fatigue on Quadriceps Strength and Activation and Knee Biomechanics in Individuals Post-Anterior Cruciate Ligament Reconstruction and Healthy Adults** | **10.2519/jospt.2015.5785** | **Excluded** | **Not both male and female** |
| **105** |
| **106** |
| **107** | **Thomas, A. C.et al.** | **Isolated hip and ankle fatigue are unlikely risk factors for anterior cruciate ligament injury** | **10.1111/j.1600-0838.2009.01076.x** | **Excluded** | **Not both male and female** |
| **108** | **Vermeulen, S.et al.** | **The effect of fatigue on spike jump biomechanics in view of patellar tendon loading in volleyball** | **10.1111/sms.14458** | **Excluded** | **Not both male and female** |
| **109** | **Watanabe, S.et al.** | **Effect of short-term fatigue, induced by high-intensity exercise, on the profile of the ground reaction force during single-leg anterior drop-jump** | **10.1589/jpts.28.3371** | **Excluded** | **Not both male and female** |
| **110** | **Webster, K. A.et al.** | **Muscle Activation During Landing Before and After Fatigue in Individuals With or Without Chronic Ankle Instability** | **10.4085/1062-6050-51.10.01** | **Excluded** | **Not both male and female** |
| **111** | **Webster, K. E.et al.** | **Effect of fatigue on landing biomechanics after anterior cruciate ligament reconstruction surgery** | **10.1249/MSS.0b013e31823fe28d** | **Excluded** | **Not both male and female** |
| **112** | **Wesley, C. A.et al.** | **Lower Extremity Landing Biomechanics in Both Sexes After a Functional Exercise Protocol** | **10.4085/1062-6050-50.8.03** | **Excluded** | **Not Fatigue** |
| **113** | **Wikstrom, E. A.et al.** | **Dynamic Stabilization Time After Isokinetic and Functional Fatigue** | **https://pmc.ncbi.nlm.nih.gov/articles/PMC522147/** | **Excluded** | **Not both male and female** |
| **114** | **Wild, C. Y.et al.** | **Lower Limb and Trunk Biomechanics After Fatigue in Competitive Female Irish Dancers** | **10.4085/1062-6050-52.3.12** | **Excluded** | **Not both male and female** |
| **115** | **Willems, M.et al.** | **Fatigue-induced Landing Alterations in ACL Reconstructed Athletes after Return-to-Sport** | **10.1055/a-2108-5219** | **Excluded** | **Not both male and female** |
| **116** |
| **117** |
| **118** | **Wong, T. L.et al.** | **Effects of Lower Extremity Muscle Fatigue on Knee Loading During a Forward Drop Jump to a Vertical Jump in Female Athletes** | **10.2478/hukin-2019-0122** | **Excluded** | **Not both male and female** |
| **119** | **Xia, R.et al.** | **Effects of Two Fatigue Protocols on Impact Forces and Lower Extremity Kinematics during Drop Landings: Implications for Noncontact Anterior Cruciate Ligament Injury** | **10.1155/2017/5690519** | **Excluded** | **Not both male and female** |
| **120** | **Zhang, X.et al.** | **Effects of Exercise-Induced Fatigue on Lower Extremity Joint Mechanics, Stiffness, and Energy Absorption during Landings** | **10.16469/j.css.201711006** | **Excluded** | **Not both male and female** |
| **121** | **Daniuseviciute, L.et al.** | **Sex differences in lower landing kinematics through neuromuscular fatigue** | **10.5755/j01.mech.19.5.5532** | **Excluded** | **Wrong outcome** |
| **122** | **Jonasson, G.et al.** | **Effects of gender and fatigue on strength and activity of gluteus medius muscle during a controlled cutting maneuver in preadolescent athletes** | **10.1016/j.jelekin.2023.102779** | **Excluded** | **Wrong outcome** |
| **123** | **Kuenze, C. M.et al.** | **Quadriceps Muscle Function After Exercise in Men and Women With a History of Anterior Cruciate Ligament Reconstruction** | **10.4085/1062-6050-49.3.46** | **Excluded** | **Wrong outcome** |
| **124** |
| **125** |
| **126** | **Weeks, B. K.et al.** | **Effect of sex and fatigue on single leg squat kinematics in healthy young adults** | **10.1186/s12891-015-0739-3** | **Excluded** | **Wrong outcome** |
| **127** | **Borotikar, B. S.et al.** | **Combined effects of fatigue and decision making on female lower limb landing postures: central and peripheral contributions to ACL injury risk** | **10.1016/j.clinbiomech.2007.08.008** | **Excluded** | **Not both male and female** |
| **128** |
| **129** |
| **130** | **Dickin, D. C.et al.** | **Combined Effects of Drop Height and Fatigue on Landing Mechanics in Active Females** | **10.1123/jab.2014-0190** | **Excluded** | **Not both male and female** |
| **131** | **Hollman, J. H.et al.** | **Effects of hip extensor fatigue on lower extremity kinematics during a jump-landing task in women: a controlled laboratory study** | **10.1016/j.clinbiomech.2012.07.004** | **Excluded** | **Not both male and female** |
| **132** | **Kıyak, G.et al.** | **The effect of neuromuscular fatigue created in the core region on the biomechanics of landing** | **10.47447/tjsm.0705** | **Excluded** | **Not both male and female** |
| **133** | **Lin, C. C.et al.** | **Effects of Different Ankle Supports on the Single-Leg Lateral Drop Landing Following Muscle Fatigue in Athletes with Functional Ankle Instability** | **10.3390/ijerph17103438** | **Excluded** | **Not both male and female** |
| **134** | **Malmir, K.et al.** | **Comparing the Effects of Peroneal Muscle Fatigue and Cyclic Loading on Ankle Neuromuscular Control During Lateral-Hop Landing** | **10.1123/jsr.2014-0165** | **Excluded** | **Not both male and female** |
| **135** | **Ortiz, A.et al.** | **Fatigue effects on knee joint stability during two jump tasks in women** | **10.1519/JSC.0b013e3181c7c5d4** | **Excluded** | **Not both male and female** |
| **136** | **Pryor, J. L.et al.** | **Movement Technique During Jump-Landing Differs Between Sex Among Athletic Playing Surfaces** | **10.1519/JSC.0000000000003520** | **Excluded** | **Wrong outcome** |
| **137** | **Sandrey, M. A.et al.** | **The Effect of Fatigue on Leg Muscle Activation and Tibial Acceleration During a Jumping Task** | **10.1123/jsr.2018-0495** | **Excluded** | **Not both male and female** |
| **138** | **Tamura, A.et al.** | **Fatigue Alters Landing Shock Attenuation During a Single-Leg Vertical Drop Jump** | **10.1177/2325967115626412** | **Excluded** | **Not both male and female** |
| **139** |
| **140** |
| **141** | **Barber-Westin, S.et al.** | **Effect of fatigue and gender on lower limb neuromuscular function** | **10.1007/978-3-662-56558-2_14** | **Excluded** | **Wrong outcome** |
| **142** | **Bitterman, N.et al.** | **Recreational diving: Re-evaluation of task, environment, and equipment definitions** | **10.1080/17461390902874057** | **Excluded** | **Wrong outcome** |
| **143** | **Bossuyt, F. M.et al.** | **The Utility of a High-intensity Exercise Protocol to Prospectively Assess ACL Injury Risk** | **10.1055/s-0035-1555930** | **Excluded** | **Not both male and female** |
| **144** |
| **145** |
| **146** | **Cerullo, J. F.et al.** | **THE EFFECTS OF FUNCTIONAL FATIGUE AND GENDER ON VERTICAL GROUND REACTION FORCES AND KNEE FLEXION PATTERNS DURING DROP LANDINGS** | **10.1097/00005768-200105001-00243** | **Excluded** | **Not both male and female** |
| **147** | **Chang, J. S.et al.** | **Differences of ground reaction forces and kinematics of lower extremity according to landing height between flat and normal feet** | **10.3233/bmr-2012-0306** | **Excluded** | **Wrong outcome** |
| **148** | **Choi, J. H.et al.** | **Comparison of the Loading Rate and Lower Limb Angles on Drop-landing between a Normal Foot and Flatfoot** | **10.1589/jpts.24.1153** | **Excluded** | **Not both male and female** |
| **149** | **Domire, Z. J.et al.** | **An examination of possible quadriceps force at the time of anterior cruciate ligament injury during landing: A simulation study** | **10.1016/j.jbiomech.2011.03.001** | **Excluded** | **Wrong outcome** |
| **150** |
| **151** |
| **152** | **Ericksen, H. M.et al.** | **Jump-landing biomechanics following a 4-week real-time feedback intervention and retention** | **10.1016/j.clinbiomech.2016.01.005** | **Excluded** | **Not both male and female** |
| **153** |
| **154** |
| **155** | **Fidai, M. S.et al.** | **Fatigue Increases Dynamic Knee Valgus in Youth Athletes: Results From a Field-Based Drop-Jump Test** | **10.1016/j.arthro.2019.07.018** | **Excluded** | **Not both male and female** |
| **156** | **Herrington, L.et al.** | **The effect of hip abductor muscle fatigue on frontal plane knee projection angle during step landing** | **10.1123/ijatt.2014-0003** | **Excluded** | **Not both male and female** |
| **157** | **Hughes, G.et al.** | **Gender differences in intra-limb coordination during single limb landings on dominant and non-dominant leg** | **10.14198/jhse.2020.151.02** | **Excluded** | **Not Fatigue** |
| **158** | **Jounger, S. L.et al.** | **Repeated buffered acidic saline infusion in the human masseter muscle as a putative experimental pain model** | **10.1038/s41598-019-51670-3** | **Excluded** | **Not Fatigue** |
| **159** | **Liederbach, M.et al.** | **Incidence of anterior cruciate ligament injuries among elite ballet and modern dancers - A 5-year prospective study** | **10.1177/0363546508323644** | **Excluded** | **Not Fatigue** |
| **160** | **Malloy, P. J.et al.** | **HIP EXTERNAL ROTATOR STRENGTH IS ASSOCIATED WITH BETTER DYNAMIC CONTROL OF THE LOWER EXTREMITY DURING LANDING TASKS** | **10.1519/jsc.0000000000001069** | **Excluded** | **Not Fatigue** |
| **161** | **McGovern, A.et al.** | **Lower limb kinematics of male and female soccer players during a self-selected cutting maneuver: Effects of prolonged activity** | **10.1016/j.knee.2015.05.005** | **Excluded** | **Wrong outcome** |
| **162** | **McLean, S. G.et al.** | **The ACL injury enigma: We can't prevent what we don't understand** | **10.4085/1062-6050-43.5.538** | **Excluded** | **Not Fatigue** |
| **163** | **Njieassam, E. S.et al.** | **Gender Discrimination: Contribution to the Burdens of Malnutrition in Communities in Buea, the South-West Region of Cameroon** | **10.1159/000507496** | **Excluded** | **Wrong outcome** |
| **164** | **Ogasawara, I.et al.** | **Gender differences of lower extremity kinematics during single leg landing** | **10.7600/jspfsm.55.403** | **Excluded** | **Not Fatigue** |
| **165** | **Olson, M. W.et al.** | **Static loading of the knee joint results in modified single leg landing biomechanics** | **10.1371/journal.pone.0219648** | **Excluded** | **Not Fatigue** |
| **166** | **Ortiz, A.et al.** | **Landing Mechanics During Side Hopping and Crossover Hopping Maneuvers in Noninjured Women and Women With Anterior Cruciate Ligament Reconstruction** | **10.1016/j.pmrj.2010.10.018** | **Excluded** | **Not both male and female** |
| **167** | **Park, J.et al.** | **Single-Leg Drop Jump Biomechanics After Ankle or Knee Joint Cooling in Healthy Young Adults** | **10.1123/jsr.2020-0529** | **Excluded** | **Not Fatigue** |
| **168** | **Powell, D. W.et al.** | **Arch structure is associated with unique joint work, relative joint contributions and stiffness during landing** | **10.1016/j.humov.2016.06.017** | **Excluded** | **Not Fatigue** |
| **169** | **Rawcliffe, A. J.et al.** | **Altered Dynamic Postural Stability and Joint Position Sense Following British Army Foot-Drill** | **10.3389/fspor.2020.584275** | **Excluded** | **Wrong outcome** |
| **170** | **Riley, C. B.et al.** | **Horse Injury during Non-Commercial Transport: Findings from Researcher-Assisted Intercept Surveys at Southeastern Australian Equestrian Events** | **10.3390/ani6110065** | **Excluded** | **Wrong outcome** |
| **171** | **Sanna, G.et al.** | **Fatigue-related changes in stance leg mechanics during sidestep cutting maneuvers** | **10.1016/j.clinbiomech.2008.03.065** | **Excluded** | **Not both male and female** |
| **172** | **Sutherlin, M. A.et al.** | **Landing Stiffness Between Individuals With and Without a History of Low Back Pain** | **10.1123/jsr.2017-0081** | **Excluded** | **Not Fatigue** |
| **173** |
| **174** |
| **175** | **Wang, Y.et al.** | **Effects of Hip Abductor Fatigue on Neuromuscular Control During Single-Leg Side-Jump Landing in Different Gender Groups** | **10.16156/j.1004-7220.2022.03.023** | **Excluded** | **Wrong outcome** |
| **176** | **Wright, M.et al.** | **The Effect of a Simulated Soccer Match on Anterior Cruciate Ligament Injury Risk Factors** | **10.1055/s-0043-109238** | **Excluded** | **Wrong outcome** |
| **177** | **Yadav, S. S.et al.** | **Vulnerability of women to climate change in arid and semi-arid regions: The case of India and South Asia** | **10.1016/j.jaridenv.2017.08.001** | **Excluded** | **Wrong outcome** |
| **178** | **Yeow, C. H.et al.** | **Effect of landing height on frontal plane kinematics, kinetics and energy dissipation at lower extremity joints** | **10.1016/j.jbiomech.2009.05.017** | **Excluded** | **Not both male and female** |
| **179** | **Yeow, C. H.et al.** | **Sagittal knee joint kinematics and energetics in response to different landing heights and techniques** | **10.1016/j.knee.2009.07.015** | **Excluded** | **Not both male and female** |
| **180** | **DiFabio, M.et al.** | **Relationships of Functional Tests Following ACL Reconstruction: Exploratory Factor Analyses of the Lower Extremity Assessment Protocol** | **10.1123/jsr.2016-0126** | **Excluded** | **Wrong outcome** |
| **181** | **Dominguese, D. J.et al.** | **Alterations in peak ground-reaction force during 60-cm drop landings caused by a single session of repeated Wingate anaerobic tests** | **10.1123/jsr.21.4.306** | **Excluded** | **Wrong outcome** |
| **182** | **Drury, B.et al.** | **Eccentric Resistance Training in Youth: Perspectives for Long-Term Athletic Development** | **10.3390/jfmk4040070** | **Excluded** | **Wrong outcome** |
| **183** | **Dunn, E. C.et al.** | **Human behaviours associated with dominance in elite amateur boxing bouts: A comparison of winners and losers under the Ten Point Must System** | **10.1371/journal.pone.0188675** | **Excluded** | **Wrong outcome** |
| **184** | **Dussault, C.et al.** | **Heart rate and autonomic balance during stand tests before and after fighter combat missions** | **10.3357/asem.2494.2009** | **Excluded** | **Wrong outcome** |
| **185** | **Dziendzikowski, M.et al.** | **Application of Operational Load Monitoring System for Fatigue Estimation of Main Landing Gear Attachment Frame of an Aircraft** | **10.3390/ma14216564** | **Excluded** | **Wrong outcome** |
| **186** | **Edwards, B.et al.** | **The effects of circadian rhythmicity and time-awake on a simple motor task** | **10.1080/07420520701795316** | **Excluded** | **Wrong outcome** |
| **187** |
| **188** |
| **189** | **Anderson, T.et al.** | **Anterior Cruciate Ligament Injury Risk by Season Period and Competition Segment: An Analysis of National Collegiate Athletic Association Injury Surveillance Data** | **10.4085/1062-6050-501-17** | **Excluded** | **Wrong outcome** |
| **190** | **Engelen-van Melick, N.et al.** | **Functional performance 2-9 years after ACL reconstruction: cross-sectional comparison between athletes with bone-patellar tendon-bone, semitendinosus/gracilis and healthy controls** | **10.1007/s00167-015-3801-7** | **Excluded** | **Wrong outcome** |
| **191** | **Heil, J.et al.** | **Load-Induced Changes of Inter-Limb Asymmetries in Dynamic Postural Control in Healthy Subjects** | **10.3389/fnhum.2022.824730** | **Excluded** | **Wrong outcome** |
| **192** |
| **193** |
| **194** | **Homan, K. J.et al.** | **The influence of hip strength on gluteal activity and lower extremity kinematics** | **10.1016/j.jelekin.2012.11.009** | **Excluded** | **Not Fatigue** |
| **195** | **Hovey, S.et al.** | **The effect of landing type on kinematics and kinetics during single-leg landings** | **10.1080/14763141.2019.1582690** | **Excluded** | **Wrong outcome** |
| **196** | **Mache, M. A.et al.** | **Effects of decision making on landing mechanics as a function of task and sex** | **10.1016/j.clinbiomech.2012.10.001** | **Excluded** | **Not Fatigue** |
| **197** |
| **198** |
| **199** | **Rosen, A.et al.** | **Differences in Lateral Drop Jumps From an Unknown Height Among Individuals With Functional Ankle Instability** | **10.4085/1062-6050-48.5.05** | **Excluded** | **Not Fatigue** |
| **200** | **Honn, K. A.et al.** | **Fatiguing effect of multiple take-offs and landings in regional airline operations** | **10.1016/j.aap.2015.10.005** | **Excluded** | **Not both male and female** |
| **201** | **Hoshiba, O.et al.** | **The effect of ankle braces on ankle motion during one-legged lateral side hop landing** | **10.1002/jor.23247** | **Excluded** | **Not Fatigue** |
| **202** | **Billaut, F.et al.** | **Effect of fatigue on hamstring co-activation during repeated-sprint exercise in males and females** | **10.1016/j.jsams.2010.10.631** | **Excluded** | **Wrong outcome** |
| **203** | **Tanaka, R.et al.** | **The effects of fatigue on the biomechanics of Drop Vertical Jump in male amateur recreational players** | **10.1002/jor.23247** | **Excluded** | **Not both male and female** |
| **204** | **Yu, Bing.et al.** | **Mechanisms of non-contact ACL injuries** | **10.1136/bjsm.2007.037192** | **Excluded** | **Wrong outcome** |
| **205** | **Slauterbeck, James R.et al.** | **The menstrual cycle, sex hormones, and anterior cruciate ligament injury** | **10.4085/1062-6050-37.3.275** | **Excluded** | **Wrong outcome** |
| **206** | **Nakamura, N.** | **Does Robotic Milling For Stem Implantation in Cementless THA Result in Improved Outcomes Scores or Survivorship Compared with Hand Rasping? Results of a Randomized Trial at 10 Years** | **[10.1007/s11999-010-1507-1](https://doi.org/10.1007/s11999-010-1507-1)** | **Excluded** | **Not related to inclusion criteria** |
| **207** | **Nakayama, A.** | **Effects of Vitamin D intake and status on changes in distal tibia strength in marine recruits undergoing training** | **[10.1002/art.39977](https://doi.org/10.1002/art.39977)** | **Excluded** | **Not related to the purpose of the article** |
| **208** | **Nakayama, A. T.** | **A dietary pattern rich in calcium, potassium, and protein is associated with tibia bone mineral content and strength in young adults entering initial military training** | **[10.3945/ajcn.116.140780](https://doi.org/10.3945/ajcn.116.140780)** | **Excluded** | **Not related to the purpose of the article** |
| **209** | **Nakipoğlu, G. F.** | **The biomechanics of the lumbosacral region in acute and chronic low back pain patients** | **[10.1177/0018720819827457](https://doi.org/10.1177/0018720819827457)** | **Excluded** | **Not related to the purpose of the article** |
| **210** | **Nam, D.** | **Cemented Versus Cementless Total Knee Arthroplasty of the Same Modern Design: a Prospective, Randomized Trial** | **[10.2106/JBJS.18.01162](https://doi.org/10.2106/JBJS.18.01162)** | **Excluded** | **Not related to inclusion criteria** |
| **211** | **Nampiaparampil, D. E.** | **Pain and prejudice** | **[10.1111/j.1526-4637.2009.00612.x](https://doi.org/10.1111/j.1526-4637.2009.00612.x)** | **Excluded** | **Not related to inclusion criteria** |
| **212** | **Nanda, R.** | **A RANDOMIZED CONTROLLED CLINICAL TRIAL TO EVALUATE THE EFFICACY OF PUNARNAVADI PINDA SVEDA IN KATIGRAHA** | **-** | **Excluded** | **Not related to the purpose of the article** |
| **213** | **Nanjegowda, N.** | **The effects of intrathecal midazolam on the duration of analgesia in patients undergoing knee arthroscopy** | **[10.4103/1658-0274.136435](https://doi.org/10.4103/1658-0274.136435)** | **Excluded** | **Not related to inclusion criteria** |
| **214** | **Nanjundeswaran, C.** | **Metabolic Mechanisms of Vocal Fatigue** | **-** | **Excluded** | **Not related to the purpose of the article** |
| **215** | **Naqvi, M. A.** | **Effectiveness of Diacerein in Primary Knee Osteoarthritis** | **[10.3350/pjmhs20221612265](https://doi.org/10.3350/pjmhs20221612265)** | **Excluded** | **Not related to inclusion criteria** |
| **216** | **Nardello, F.** | **Kinematic and mechanical changes during a long half-marathon race: males and females at uphill/downhill slopes** | **[10.23736/s0022-4707.20.11177-0](https://doi.org/10.23736/s0022-4707.20.11177-0)** | **Excluded** | **Not related to inclusion criteria** |
| **217** | **Narkbunnam, R.** | **Alignment accuracy and functional outcomes between hand-held navigation and conventional instruments in TKA: a randomized controlled trial** | **[10.1186/s12891-022-05872-y](https://doi.org/10.1186/s12891-022-05872-y)** | **Excluded** | **Not related to the purpose of the article** |
| **218** | **Nasr, A.** | **Experimental Study of Fully Passive, Fully Active, and Active-Passive Upper-Limb Exoskeleton Efficiency: An Assessment of Lifting Tasks** | **[10.3390/s24010063](https://doi.org/10.3390/s24010063)** | **Excluded** | **Not related to inclusion criteria** |
| **219** | **Nassar, M. K.** | **Symptom burden, fatigue, sleep quality and perceived social support in hemodialysis patients with musculoskeletal discomfort: a single center experience from Egypt** | **[10.1186/s40891-023-06910-z](https://doi.org/10.1186/s40891-023-06910-z)** | **Excluded** | **Not related to the purpose of the article** |
| **220** | **Nasser, I.** | **Limited cardiopulmonary capacity in patients with liver cirrhosis when compared to healthy subjects** | **[10.1590/1806-9282.67.01.20200449](https://doi.org/10.1590/1806-9282.67.01.20200449)** | **Excluded** | **Not related to inclusion criteria** |
| **221** | **Naughton, G. A.** | **Accumulated oxygen deficit measurements during and after high-intensity exercise in trained male and female adolescents** | **[10.1007/s00421010050285](https://doi.org/10.1007/s00421010050285)** | **Excluded** | **Not related to inclusion criteria** |
| **222** | **Navali, A. M.** | **Anew Shoe Decreases Pain and Fatigue in Ascending and Descending the stairs in patients with knee osteoarthritis** | **-** | **Excluded** | **Not related to the purpose of the article** |
| **223** | **Navarro, S. L.** | **Differences in serum protein biomarkers between combined glucosamine and chondroitin versus celecoxib treatment in a randomized, double-blind trial in osteoarthritis patients** | **[10.1002/art.39977](https://doi.org/10.1002/art.39977)** | **Excluded** | **Not related to the purpose of the article** |
| **224** | **Navarro, S. L.** | **Differences in Serum Biomarkers Between Combined Glucosamine and Chondroitin Versus Celecoxib in a Randomized, Double-blind Trial in Osteoarthritis Patients** | **[10.2174/1871523018666190115094512](https://doi.org/10.2174/1871523018666190115094512)** | **Excluded** | **Not related to the purpose of the article** |
| **225** | **Navas, C. A.** | **Sexual dimorphism of extensor carpi radialis muscle size, isometric force, relaxation rate and stamina during the breeding season of the frog <i>Rana temporaria</i> Linnaeus 1758** | **[10.1242/jeb.000646](https://doi.org/10.1242/jeb.000646)** | **Excluded** | **Not related to inclusion criteria** |
| **226** | **Navas-Carretero, S.** | **The Impact of Gender and Protein Intake on the Success of Weight Maintenance and Associated Cardiovascular Risk Benefits, Independent of the Mode of Food Provision: the DiOGenes Randomized Trial** | **[10.1080/07315724.2014.948642](https://doi.org/10.1080/07315724.2014.948642)** | **Excluded** | **Not related to inclusion criteria** |
| **227** | **Nayak, K. N.** | **Prevalence of heterotopic ossification in cemented versus noncemented total hip joint replacement in patients with osteoarthrosis: a randomized clinical trial** | **[10.1007/s00132-010-1507-1](https://doi.org/10.1007/s00132-010-1507-1)** | **Excluded** | **Not related to the purpose of the article** |
| **228** | **Nazarpour, S.** | **Sexual function and exercise in post menopausal women residing in Chalous and Nowshahr, northern Iran** | **[10.5812/ircmj.30120](https://doi.org/10.5812/ircmj.30120)** | **Excluded** | **Not related to the purpose of the article** |
| **229** | **nbcsbw, R. B. R.** | **Functional Exercise: a new approach to managing Systemic Sclerosis** | **-** | **Excluded** | **Not related to inclusion criteria** |
| **230** | **Nct** | **Redesigning Patient Handling Tasks to** |  | **Excluded** | **Review articles, conference papers** |
| **231** | **Nct** | **Redesigning Patient Handling Tasks to Prevent Nursing Back Injuries** | **[NCT00012844](https://clinicaltrials.gov/show/NCT00012844)** | **Excluded** | **Review articles, conference papers** |
| **232** | **Nct** | **The D-KAF (Dalteparin in Knee-to-Ankle Fracture) Trial** | **[NCT00187408](https://clinicaltrials.gov/show/NCT00187408)** | **Excluded** | **Review articles, conference papers** |
| **233** | **Nct** | **Does Early Ambulation After Hip Fracture Surgery Accelerate Recovery?** | **[NCT00224367](https://clinicaltrials.gov/show/NCT00224367)** | **Excluded** | **Review articles, conference papers** |
| **234** | **Nct** | **Effect of Sling Suspension Exercises in Proprioception of Patients With Knee Osteoarthritis** | **[NCT00154765](https://clinicaltrials.gov/show/NCT00154765)** | **Excluded** | **Review articles, conference papers** |
| **235** | **Nct** | **HIP: HIV Intervention for Providers** | **[NCT00164398](https://clinicaltrials.gov/show/NCT00164398)** | **Excluded** | **Review articles, conference papers** |
| **236** | **Nct** | **The Outcome and Cost Analysis of Home-care Physical Therapy for Postoperative Hip Fracture Patients** | **[NCT00155597](https://clinicaltrials.gov/show/NCT00155597)** | **Excluded** | **Review articles, conference papers** |
| **237** | **Nct** | **Gait Training For Acute Stroke: functional Neuromuscular Stimulation (FNS) and Weight Supported Treadmill Training** | **[NCT00101543](https://clinicaltrials.gov/show/NCT00101543)** | **Excluded** | **Review articles, conference papers** |
| **238** | **Nct** | **Healthetech, Inc. SMART Study** | **[NCT00194194](https://clinicaltrials.gov/show/NCT00194194)** | **Excluded** | **Review articles, conference papers** |
| **239** | **Nct** | **Does Early Ambulation After Hip Fracture Surgery Accelerate Recovery?** | **[NCT00224367](https://clinicaltrials.gov/show/NCT00224367)** | **Excluded** | **Review articles, conference papers** |
| **240** | **Nct** | **Effect of Sling Suspension Exercises in Proprioception of Patients With Knee Osteoarthritis** | **[NCT00154765](https://clinicaltrials.gov/show/NCT00154765)** | **Excluded** | **Review articles, conference papers** |
| **241** | **Nct** | **Local Anesthesia for Prostate Biopsy** | **[NCT00422708](https://clinicaltrials.gov/show/NCT00422708)** | **Excluded** | **Review articles, conference papers** |
| **242** | **Nct** | **The Outcome and Cost Analysis of Home-care Physical Therapy for Postoperative Hip Fracture Patients** | **[NCT00155597](https://clinicaltrials.gov/show/NCT00155597)** | **Excluded** | **Review articles, conference papers** |
| **243** | **Nct** | **Evaluation of Chylomicrons Metabolism in Sub-Clinical Atherosclerosis in Patients With Heterozygous Familial Hypercholesterolemia (FH) Treated With Statin Plus Ezetimibe** | **[NCT00475826](https://clinicaltrials.gov/show/NCT00475826)** | **Excluded** | **Review articles, conference papers** |
| **244** | **Nct** | **GRIB Forsoeget-2004** | **[NCT00554645](https://clinicaltrials.gov/show/NCT00554645)** | **Excluded** | **Review articles, conference papers** |
| **245** | **Nct** | **Intraoperative Fluid Management Based on Arterial Pulse Pressure Variation During High-Risk Surgery** | **[NCT00479011](https://clinicaltrials.gov/show/NCT00479011)** | **Excluded** | **Review articles, conference papers** |
| **246** | **Nct** | **Effectiveness of Nigella Sativa (Kalonji) Seed in Dyslipidemia** | **[NCT00327054](https://clinicaltrials.gov/show/NCT00327054)** | **Excluded** | **Review articles, conference papers** |
| **247** | **Nct** | **Exercise Therapy and Patient Education for Individuals With Hip Osteoarthritis. a RCT** | **[NCT00319423](https://clinicaltrials.gov/show/NCT00319423)** | **Excluded** | **Review articles, conference papers** |
| **248** | **Nct** | **The Combined Effects of Resistance Training and Flax Oil Supplementation Upon Inflammation in Older Adults** | **[NCT00465153](https://clinicaltrials.gov/show/NCT00465153)** | **Excluded** | **Review articles, conference papers** |
| **249** | **Nct** | **Comparison Between Two Techniques of Volemic Expansion in Hip Replacement Arthroplasty** | **[NCT00542516](https://clinicaltrials.gov/show/NCT00542516)** | **Excluded** | **Review articles, conference papers** |
| **250** | **Nct** | **Comparison of Two Different Methods of Delivering Local Analgesia During Intra-articular Corticosteroid Injections in Children With Juvenile Idiopathic Arthritis** | **[NCT00465504](https://clinicaltrials.gov/show/NCT00465504)** | **Excluded** | **Review articles, conference papers** |
| **251** | **Nct** | **Treatment of Hypovitaminosis D in Rheumatoid Arthritis** | **[NCT00423358](https://clinicaltrials.gov/show/NCT00423358)** | **Excluded** | **Review articles, conference papers** |
| **252** | **Nct** | **Trial Comparing Navigated and Conventional Implantation Techniques in Knee Replacement Surgery** | **[NCT00431509](https://clinicaltrials.gov/show/NCT00431509)** | **Excluded** | **Review articles, conference papers** |
| **253** | **Nct** | **Cognitive Effects of Inhalational Versus Intravenous General Anesthesia in the Elderly** | **[NCT00788008](https://clinicaltrials.gov/show/NCT00788008)** | **Excluded** | **Review articles, conference papers** |
| **254** | **Nct** | **Effectiveness and Safety of Topical Halobetasol Propionate in the Treatment of Patients With Psoriasis** | **[NCT00715975](https://clinicaltrials.gov/show/NCT00715975)** | **Excluded** | **Review articles, conference papers** |
| **255** | **Nct** | **Effects of an Herbal Topical Cream on Osteoarthritis Symptoms, Biomarkers, and Disease Progression in the Knee** | **[NCT00800098](https://clinicaltrials.gov/show/NCT00800098)** | **Excluded** | **Review articles, conference papers** |
| **256** | **Nct** | **Osteonecrosis of the Hip and Bisphosphonate Treatment** | **[NCT00781261](https://clinicaltrials.gov/show/NCT00781261)** | **Excluded** | **Review articles, conference papers** |
| **257** | **Nct** | **Yellow Fever Virus Vaccine and Immune Globulin Study** | **[NCT00254826](https://clinicaltrials.gov/show/NCT00254826)** | **Excluded** | **Review articles, conference papers** |
| **258** | **Nct** | **Do Musculoskeletal Techniques Improve Forced Expiratory Volume in One Second in Adults With Cystic Fibrosis?** | **[https://clinicaltrials.gov/show/NCT00716664](https://clinicaltrials.gov/show/NCT00716664)** | **Excluded** | **Review articles, conference papers** |
| **259** | **Nct et al** | **Gene Expression in Obesity and Insulin Resistance.** | **[https://clinicaltrials.gov/show/NCT00621205](https://clinicaltrials.gov/show/NCT00621205)** | **Excluded** | **Review articles, conference papers** |
| **260** | **Nct et al** | **Influence of External Factors on Skeletal Growth in Youth.** | **[https://clinicaltrials.gov/show/NCT00633828](https://clinicaltrials.gov/show/NCT00633828)** | **Excluded** | **Review articles, conference papers** |
| **261** | **Nct et al** | **Pentoxifylline Versus Pioglitazone In Non‐Alcoholic Steatohepatiti (NASH).** | **[https://clinicaltrials.gov/show/NCT00681733](https://clinicaltrials.gov/show/NCT00681733)** | **Excluded** | **Review articles, conference papers** |
| **262** | **Nct et al** | **Plate Fixation of Distal Femur Fractures: a Protocol for a Study of Two Plate Options.** | **[https://clinicaltrials.gov/show/NCT00644397](https://clinicaltrials.gov/show/NCT00644397)** | **Excluded** | **Review articles, conference papers** |
| **263** | **Nct et al** | **Thiazolidinediones Or Sulphonylureas and Cardiovascular Accidents.Intervention Trial.** | **[https://clinicaltrials.gov/show/NCT00700856](https://clinicaltrials.gov/show/NCT00700856)** | **Excluded** | **Review articles, conference papers** |
| **264** | **Nct et al** | **Augment™ Injectable Bone Graft Compared to Autologous Bone Graft in Foot and Ankle Fusions.** | **[https://clinicaltrials.gov/show/NCT01008891](https://clinicaltrials.gov/show/NCT01008891)** | **Excluded** | **Review articles, conference papers** |
| **265** | **Nct et al** | **Comparison of Function and Fit of Standard and Gender‐Specific CR High‐Flex Total Knee Prostheses.** | **[https://clinicaltrials.gov/show/NCT00916968](https://clinicaltrials.gov/show/NCT00916968)** | **Excluded** | **Review articles, conference papers** |
| **266** | **Nct et al** | **Comparison of Rotating vs. Fixed Platform of the COLUMBUS Knee Prosthesis.** | **[https://clinicaltrials.gov/show/NCT00822640](https://clinicaltrials.gov/show/NCT00822640)** | **Excluded** | **Review articles, conference papers** |
| **267** | **Nct et al** | **Comparison of Two Methods of Securing Skin Grafts Using Negative Pressure Wound Therapy: vacuum Assisted Closure (VAC) and Gauze Suction (GSUC).** | **[https://clinicaltrials.gov/show/NCT00952120](https://clinicaltrials.gov/show/NCT00952120)** | **Excluded** | **Review articles, conference papers** |
| **268** | **Nct et al** | **Electrical Muscle Stimulation (EMS), a Preventive and Therapeutic Tool for Critical Illness Polyneuromyopathy (CIPNM).** | **[https://clinicaltrials.gov/show/NCT00882830](https://clinicaltrials.gov/show/NCT00882830)** | **Excluded** | **Review articles, conference papers** |
| **269** | **Nct et al** | **Exercise for Patients With Heart Failure in Primary Care: the EFICAR.** | **[https://clinicaltrials.gov/show/NCT01033591](https://clinicaltrials.gov/show/NCT01033591)** | **Excluded** | **Review articles, conference papers** |
| **270** | **Nct et al** | **Following Lipectomy to Understand Adipose Tissue Re‐accumulation.** | **[https://clinicaltrials.gov/show/NCT00995631](https://clinicaltrials.gov/show/NCT00995631)** | **Excluded** | **Review articles, conference papers** |
| **271** | **Nct et al** | **Footwear and Injury Prevention Study.** | **[https://clinicaltrials.gov/show/NCT00832195](https://clinicaltrials.gov/show/NCT00832195)** | **Excluded** | **Review articles, conference papers** |
| **272** | **Nct et al** | **A Gender‐Specific Posterior Cruciate‐Substituting High‐Flexion Knee Prosthesis Does Not Improve Fit and Function.** | **[https://clinicaltrials.gov/show/NCT00917774](https://clinicaltrials.gov/show/NCT00917774)** | **Excluded** | **Review articles, conference papers** |
| **273** | **Nct et al** | **GLP‐1 Therapy for Weight Loss and Improved Glucose Tolerance in Obese Children.** | **[https://clinicaltrials.gov/show/NCT00886626](https://clinicaltrials.gov/show/NCT00886626)** | **Excluded** | **Review articles, conference papers** |
| **274** | **Nct et al** | **Health Education Materials With/Out a Physical Activity Program for Patients Who Have Undergone Treatment for High‐Risk Stage II or Stage III Colon Cancer.** | **[https://clinicaltrials.gov/ct2/show/NCT00819208](https://clinicaltrials.gov/ct2/show/NCT00819208)** | **Excluded** | **Review articles, conference papers** |
| **275** | **Nct et al** | **Impact of Obstructive Sleep Apnea Syndrome on Metabolic Syndrome in Severe Obesity.** | **[https://clinicaltrials.gov/show/NCT01029561](https://clinicaltrials.gov/show/NCT01029561)** | **Excluded** | **Review articles, conference papers** |
| **276** | **Nct et al** | **Motion Analysis of EMP Knee Versus Posterior Stabilized Knee Arthroplasty for Osteoarthritis.** | **[https://clinicaltrials.gov/show/NCT00967161](https://clinicaltrials.gov/show/NCT00967161)** | **Excluded** | **Review articles, conference papers** |
| **277** | **Nct et al** | **NexGen Gender Fixed Bearing Knee Versus NexGen Legacy Knee Posterior Stabilized (LPS) - Flex Fixed Bearing Knee: a Study Comparing Two Different Knee Prostheses.** | **[https://clinicaltrials.gov/show/NCT00901329](https://clinicaltrials.gov/show/NCT00901329)** | **Excluded** | **Review articles, conference papers** |
| **278** | **Nct et al** | **Optimizing Local Anesthetic Concentration for Continuous Lumbar Plexus Nerve Blocks.** | **[https://clinicaltrials.gov/show/NCT00912873](https://clinicaltrials.gov/show/NCT00912873)** | **Excluded** | **Review articles, conference papers** |
| **279** | **Nct et al** | **Prophylaxis of Thromboembolic Complications Trial: Thromboprophylaxis Needed in Below Knee Plaster Cast Immobilization for Ankle and Foot Fractures.** | **[https://clinicaltrials.gov/show/NCT00881088](https://clinicaltrials.gov/show/NCT00881088)** | **Excluded** | **Review articles, conference papers** |
| **280** | **Nct et al** | **Rectal Microbicide Safety and Acceptability Trial of Topically Applied Tenofovir Compared With Oral Tablet.** | **[https://clinicaltrials.gov/show/NCT00984971](https://clinicaltrials.gov/show/NCT00984971)** | **Excluded** | **Review articles, conference papers** |
| **281** | **Nct et al** | **Study Comparing Traditional and Gender‐specific Total Knee Replacement Designs.** | **[https://clinicaltrials.gov/show/NCT00937170](https://clinicaltrials.gov/show/NCT00937170)** | **Excluded** | **Review articles, conference papers** |
| **282** | **Nct et al** | **Thrombelastography Based Dosing of Enoxaparin.** | **[https://clinicaltrials.gov/show/NCT00990236](https://clinicaltrials.gov/show/NCT00990236)** | **Excluded** | **Review articles, conference papers** |
| **283** | **Nct et al** | **Effect of Steroid Injections in a Knee With Osteoarthritis.** | **[https://clinicaltrials.gov/show/NCT01230424](https://clinicaltrials.gov/show/NCT01230424)** | **Excluded** | **Review articles, conference papers** |
| **284** | **Nct et al** | **Effects of Hamstring Training in Different Modes on Stabilizing Knee Joints With Anterior Drawer Laxity.** | **[https://clinicaltrials.gov/show/NCT01170546](https://clinicaltrials.gov/show/NCT01170546)** | **Excluded** | **Review articles, conference papers** |
| **285** | **Nct et al** | **Efficacy of Multimodal Perioperative Analgesia With Periarticular Drug Injection in Total Knee Arthroplasty (TKA).** | **[https://clinicaltrials.gov/show/NCT01042093](https://clinicaltrials.gov/show/NCT01042093)** | **Excluded** | **Review articles, conference papers** |
| **286** | **Nct et al** | **Exercise Therapy and Patient Education for Individuals With Hip Osteoarthritis. Long-term Follow-up.** | **[https://clinicaltrials.gov/show/NCT01063777](https://clinicaltrials.gov/show/NCT01063777)** | **Excluded** | **Review articles, conference papers** |
| **287** | **Nct et al** | **The LIFE Study - Lifestyle Interventions and Independence for Elders.** | **[https://clinicaltrials.gov/show/NCT01072500](https://clinicaltrials.gov/show/NCT01072500)** | **Excluded** | **Review articles, conference papers** |
| **288** | **Nct et al** | **The Benefit of Arthroscopic Partial Meniscectomy in Middle-Aged Patients.** | **[https://clinicaltrials.gov/show/NCT01264991](https://clinicaltrials.gov/show/NCT01264991)** | **Excluded** | **Review articles, conference papers** |
| **289** | **Nct et al** | **Clinical Trial of a Silver Eluting Dressing System.** | **[https://clinicaltrials.gov/show/NCT01229358](https://clinicaltrials.gov/show/NCT01229358)** | **Excluded** | **Review articles, conference papers** |
| **290** | **Nct et al** | **Effects of Transcutaneous Electrical Nerve Stimulation on Pain and Disability in Patients With Osteoarthritis.** | **[https://clinicaltrials.gov/show/NCT01137266](https://clinicaltrials.gov/show/NCT01137266)** | **Excluded** | **Review articles, conference papers** |
| **291** | **Nct et al** | **An Open Label Positron Emission Tomography Study in Healthy Male Subjects to Investigate Brain DAT and SERT Occupancy, Pharmacokinetics and Safety of Single Oral Doses of GSK1360707, Using 11C-PE2I and 11C-DASB as PET Ligands.** | **[https://clinicaltrials.gov/show/NCT01153802](https://clinicaltrials.gov/show/NCT01153802)** | **Excluded** | **Review articles, conference papers** |
| **292** | **Nct et al** | **A Pilot Study of the Wii Fit as a Low-Cost Virtual Reality System to Evaluate Balance Ability in Older Adults.** | **[https://clinicaltrials.gov/show/NCT01229969](https://clinicaltrials.gov/show/NCT01229969)** | **Excluded** | **Review articles, conference papers** |
| **293** | **Nct et al** | **Replacement of Vitamin D in Patients With Active Tuberculosis.** | **[https://clinicaltrials.gov/show/NCT01130311](https://clinicaltrials.gov/show/NCT01130311)** | **Excluded** | **Review articles, conference papers** |
| **294** | **Nct et al** | **Vitamin B6, B12, Folic Acid and Exercise in Parkinson's Disease.** | **[https://clinicaltrials.gov/show/NCT01238926](https://clinicaltrials.gov/show/NCT01238926)** | **Excluded** | **Review articles, conference papers** |
| **295** | **Nct et al** | **The ACT-OUT Trial: aCTivity OUTcomes Based on High Carbohydrate or High Fat Diet in Metabolic Syndrome.** | **[https://clinicaltrials.gov/show/NCT01357382](https://clinicaltrials.gov/show/NCT01357382)** | **Excluded** | **Review articles, conference papers** |
| **296** | **Nct et al** | **Anthropometric Changes Associated With Home-based Exercise Among School Cooks.** | **[https://clinicaltrials.gov/show/NCT01501721](https://clinicaltrials.gov/show/NCT01501721)** | **Excluded** | **Review articles, conference papers** |
| **297** | **Nct et al** | **Biomechanics of Gait Pattern Adaptation in Patients After Total Knee Arthroplasty.** | **[https://clinicaltrials.gov/show/NCT01412814](https://clinicaltrials.gov/show/NCT01412814)** | **Excluded** | **Review articles, conference papers** |
| **298** | **Nct et al** | **Calcium Metabolism in Mexican American Adolescents.** | **[https://clinicaltrials.gov/show/NCT01277185](https://clinicaltrials.gov/show/NCT01277185)** | **Excluded** | **Review articles, conference papers** |
| **299** | **Nct et al** | **A Clinical Trial to Study the Effects of a Fixed Dose Combination of Diclofenac and Eperisone Hydrochloride With Plain Eperisone Hydrochloride in Patients With Low Back Pain.** | **[https://clinicaltrials.gov/show/NCT01300312](https://clinicaltrials.gov/show/NCT01300312)** | **Excluded** | **Review articles, conference papers** |
| **300** | **Nct et al** | **Effectiveness of Manual Therapy Combined With Standard Treatment in the Management of Plantar Fasciitis.** | **[https://clinicaltrials.gov/show/NCT01439932](https://clinicaltrials.gov/show/NCT01439932)** | **Excluded** | **Review articles, conference papers** |
| **301** | **Nct et al** | **The Effects of a Walking Program on Balance, Falls and Well Being in Individuals Residing in Long-term Care.** | **[https://clinicaltrials.gov/show/NCT01277809](https://clinicaltrials.gov/show/NCT01277809)** | **Excluded** | **Review articles, conference papers** |
| **302** | **Nct et al** | **Efficacy of Spearmint Tea in Relieving Osteoarthritis of the Knee.** | **[https://clinicaltrials.gov/show/NCT01380015](https://clinicaltrials.gov/show/NCT01380015)** | **Excluded** | **Review articles, conference papers** |
| **303** | **Nct et al** | **Enriched Environments for Upper Limb Stroke Rehabilitation.** | **[https://clinicaltrials.gov/show/NCT01388400](https://clinicaltrials.gov/show/NCT01388400)** | **Excluded** | **Review articles, conference papers** |
| **304** | **Nct et al** | **EXPAREL Dose-Response for Single-Injection Femoral Nerve Blocks.** | **[https://clinicaltrials.gov/show/NCT01349140](https://clinicaltrials.gov/show/NCT01349140)** | **Excluded** | **Review articles, conference papers** |
| **305** | **Nct et al** | **Minimally Invasive Surgical Approaches In Total Knee Arthroplasty.** | **[https://clinicaltrials.gov/show/NCT01461356](https://clinicaltrials.gov/show/NCT01461356)** | **Excluded** | **Review articles, conference papers** |
| **306** | **Nct et al** | **Patient and Provider Interventions for Managing Osteoarthritis in Primary Care.** | **[https://clinicaltrials.gov/show/NCT01435109](https://clinicaltrials.gov/show/NCT01435109)** | **Excluded** | **Review articles, conference papers** |
| **307** | **Nct et al** | **Pentoxiphylline and Vitamin E Versus Vitamin E in Patients With Non- Alcoholic Steatohepatitis.** | **[https://clinicaltrials.gov/show/NCT01384578](https://clinicaltrials.gov/show/NCT01384578)** | **Excluded** | **Review articles, conference papers** |
| **308** | **Nct et al** | **Postoperative Pain Relief Following Total Hip Arthroplasty. A Comparison Between Intrathecal Morphine (IM) and Local Infiltration Analgesia (LIA).** | **[https://clinicaltrials.gov/show/NCT01281891](https://clinicaltrials.gov/show/NCT01281891)** | **Excluded** | **Review articles, conference papers** |
| **309** | **Nct et al** | **Remote Ischemic Preconditioning in Neurological Death Organ Donors.** | **[https://clinicaltrials.gov/show/NCT01515072](https://clinicaltrials.gov/show/NCT01515072)** | **Excluded** | **Review articles, conference papers** |
| **310** | **Nct et al** | **Step Monitoring to Improve ARTERial Health.** | **[https://clinicaltrials.gov/show/NCT01475201](https://clinicaltrials.gov/show/NCT01475201)** | **Excluded** | **Review articles, conference papers** |
| **311** | **Nct et al** | **Supplementation of Alpha-linolenic Acid (ALA)-Rich Oil in Humans.** | **[https://clinicaltrials.gov/show/NCT01317290](https://clinicaltrials.gov/show/NCT01317290)** | **Excluded** | **Review articles, conference papers** |
| **312** | **Nct et al** | **Teriparatide for Joint Erosions in Rheumatoid Arthritis: the TERA Trial.** | **[https://clinicaltrials.gov/show/NCT01400516](https://clinicaltrials.gov/show/NCT01400516)** | **Excluded** | **Review articles, conference papers** |
| **313** | **Nct et al** | **Ultrasound Guided Versus Palpation Guided Cricothyrotomy With Poorly Defined Anatomical Landmarks.** | **[https://clinicaltrials.gov/show/NCT01475487](https://clinicaltrials.gov/show/NCT01475487)** | **Excluded** | **Review articles, conference papers** |
| **314** | **Nct et al** | **Bone Mineral Density Changes Under Two Tibial Components in Total Knee Arthroplasty.** | **[https://clinicaltrials.gov/show/NCT01764984](https://clinicaltrials.gov/show/NCT01764984)** | **Excluded** | **Review articles, conference papers** |
| **315** | **Nct et al** | **Electrostimulation in Anterior Cruciate Ligament Reconstruction.** | **[https://clinicaltrials.gov/show/NCT01633047](https://clinicaltrials.gov/show/NCT01633047)** | **Excluded** | **Review articles, conference papers** |
| **316** | **Nct et al** | **Evaluation of DVD and Internet Decision Aids for Hip and Knee Osteoarthritis: focus on Health Literacy.** | **[https://clinicaltrials.gov/show/NCT01618097](https://clinicaltrials.gov/show/NCT01618097)** | **Excluded** | **Review articles, conference papers** |
| **317** | **Nct et al** | **Fast Track Recovery Knee Arthroplasty Project.** | **[https://clinicaltrials.gov/show/NCT01551017](https://clinicaltrials.gov/show/NCT01551017)** | **Excluded** | **Review articles, conference papers** |
| **318** | **Nct et al** | **Plantar Fasciitis Foot Insole.** | **[https://clinicaltrials.gov/show/NCT01549678](https://clinicaltrials.gov/show/NCT01549678)** | **Excluded** | **Review articles, conference papers** |
| **319** | **Nct et al** | **Project Osteoarthritis: recovering Quality of Life Through Education.** | **[https://clinicaltrials.gov/show/NCT01572051](https://clinicaltrials.gov/show/NCT01572051)** | **Excluded** | **Review articles, conference papers** |
| **320** | **Nct et al** | **Role of Autologous Platelet Rich Plasma in Total Knee Arthroplasty.** | **[https://clinicaltrials.gov/show/NCT01563380](https://clinicaltrials.gov/show/NCT01563380)** | **Excluded** | **Review articles, conference papers** |
| **321** | **Nct et al** | **Assessment of Motivity in People Aged 65 to 86 Years.** | **[NCT01706757](https://clinicaltrials.gov/show/NCT01706757)** | **Excluded** | **Review articles, conference papers** |
| **322** | **Geltrude Mingrone et al** | **Bariatric Surgery and Reactive Hypoglycemia.** | **[NCT01581801](https://clinicaltrials.gov/show/NCT01581801)** | **Excluded** | **Review articles, conference papers** |
| **323** | **Nct et al** | **BIP48 (Peginterferon Alfa 2b 48kDa) Compared With Pegasys® (Peginterferon 2a 40kDa) for Treatment of Chronic Hepatitis C.** | **[NCT01623336](https://clinicaltrials.gov/show/NCT01623336)** | **Excluded** | **Review articles, conference papers** |
| **324** | **Nct et al** | **Effect of Gabapentin on Orthopedic Pain.** | **[NCT01546857](https://clinicaltrials.gov/show/NCT01546857)** | **Excluded** | **Review articles, conference papers** |
| **325** | **Nct et al** | **Effects of Respiratory Muscle Training and Respiratory Exercise in Exercise Tolerance, Performing Daily Life Activities and Quality of Life of Patients With Chronic Obstructive Pulmonary Disease.** | **[NCT01510041](https://clinicaltrials.gov/show/NCT01510041)** | **Excluded** | **Review articles, conference papers** |
| **326** | **Nct et al** | **Effects of Vitamin D and Calcium Supplementation on Inflammatory Biomarkers and Adypocytokines in Diabetic Patients.** | **[NCT01662193](https://clinicaltrials.gov/show/NCT01662193)** | **Excluded** | **Review articles, conference papers** |
| **327** | **Nct et al** | **ET 50 for Post Caesarean Section Spinal Hypotension.** | **[NCT01561274](https://clinicaltrials.gov/show/NCT01561274)** | **Excluded** | **Review articles, conference papers** |
| **328** | **Nct et al** | **Is Chiropractic Spinal Manipulative Therapy an Efficient Treatment Option for Migraine.** | **[NCT01741714](https://clinicaltrials.gov/show/NCT01741714)** | **Excluded** | **Review articles, conference papers** |
| **329** | **Nct et al** | **Motor Sparing Block vs. Peri-Articular Catheters.** | **[NCT01503528](https://clinicaltrials.gov/show/NCT01503528)** | **Excluded** | **Review articles, conference papers** |
| **330** | **Nct et al** | **Vitamin D Absorption in HIV Infected Young Adults Being Treated With Tenofovir Containing cART.** | **[NCT01751646](https://clinicaltrials.gov/show/NCT01751646)** | **Excluded** | **Review articles, conference papers** |
| **331** | **Nct et al** | **Women's Activity and Lifestyle Study in Connecticut.** | **[NCT02107066](https://clinicaltrials.gov/show/NCT02107066)** | **Excluded** | **Review articles, conference papers** |
| **332** | **Nct et al** | **The Alberta Vascular Risk Reduction Community Pharmacy Project: rxEACH.** | **[NCT01979471](https://clinicaltrials.gov/show/NCT01979471)** | **Excluded** | **Review articles, conference papers** |
| **333** | **Nct et al** | **BioSteel Supplementation and Performance.** | **[NCT02019316](https://clinicaltrials.gov/show/NCT02019316)** | **Excluded** | **Review articles, conference papers** |
| **334** | **Nct et al** | **Comparing Blood Sugar Levels and Endothelial Function of PEAK ATP® With GlycoCarn®, PEAK ATP® and GlycoCarn® Supplements.** | **[NCT01855373](https://clinicaltrials.gov/show/NCT01855373)** | **Excluded** | **Review articles, conference papers** |
| **335** | **Nct et al** | **"Core Stability" Exercises to Improve Sitting Balance in Stroke Patients.** | **[NCT01864382](https://clinicaltrials.gov/show/NCT01864382)** | **Excluded** | **Review articles, conference papers** |
| **336** | **Nct et al** | **Effect of Diet and Physical Activity on Incidence of Type 2 Diabetes.** | **[NCT01777893](https://clinicaltrials.gov/show/NCT01777893)** | **Excluded** | **Review articles, conference papers** |
| **337** | **Nct et al** | **Effect Of Plasma Rich In Growth Factors In Knee Osteoarthritis.** | **[NCT02039531](https://clinicaltrials.gov/show/NCT02039531)** | **Excluded** | **Review articles, conference papers** |
| **338** | **Nct et al** | **Effects of a Supplement Enriched in Hydroxymethylbutyrate and Vitamin D on Muscle Strength in Hip Fracture.** | **[NCT01850251](https://clinicaltrials.gov/show/NCT01850251)** | **Excluded** | **Review articles, conference papers** |
| **339** | **Nct et al** | **ET50 With Fentanyl for Post Caesarean Section Spinal Hypotension.** | **[NCT01896960](https://clinicaltrials.gov/show/NCT01896960)** | **Excluded** | **Review articles, conference papers** |
| **340** | **Nct et al** | **Exercise and Blood Pressure Reactivity.** | **[NCT01935895](https://clinicaltrials.gov/show/NCT01935895)** | **Excluded** | **Review articles, conference papers** |
| **341** | **Nct et al** | **FES Rowing for Skeletal Health After SCI.** | **[NCT02008149](https://clinicaltrials.gov/show/NCT02008149)** | **Excluded** | **Review articles, conference papers** |
| **342** | **Nct et al** | **Hip Fractures Treated With Uncemented Arthroplasties.** | **[NCT01798472](https://clinicaltrials.gov/show/NCT01798472)** | **Excluded** | **Review articles, conference papers** |
| **343** | **Nct et al** | **Influence of Strength Training and Protein Supplementation on Fitness of Institutionalized Elderly.** | **[NCT01775111](https://clinicaltrials.gov/show/NCT01775111)** | **Excluded** | **Review articles, conference papers** |
| **344** | **Nct et al** | **Leukine (Sargramostim) for Parkinson's Disease.** | **[NCT01882010](https://clinicaltrials.gov/show/NCT01882010)** | **Excluded** | **Review articles, conference papers** |
| **345** | **Nct et al** | **Passive Leg Raise (PLR) During Cardiopulmonary Resuscitation (CPR).** | **[NCT01952197](https://clinicaltrials.gov/show/NCT01952197)** | **Excluded** | **Review articles, conference papers** |
| **346** | **Nct et al** | **A Pilot Study Comparing Tolerance of Oral Heme Iron Polypeptide With Oral Ionic Iron.** | **[NCT01865175](https://clinicaltrials.gov/show/NCT01865175)** | **Excluded** | **Review articles, conference papers** |
| **347** | **Nct et al** | **Preventing the Development of Venous Insufficiency in Pregnant Women Through Use of Compression Stockings.** | **[NCT01793194](https://clinicaltrials.gov/show/NCT01793194)** | **Excluded** | **Review articles, conference papers** |
| **348** | **Nct et al** | **REmodelling in Diabetic CardiOmapathy: gender Response to PDE5i InhibiTOrs.** | **[NCT01803828](https://clinicaltrials.gov/show/NCT01803828)** | **Excluded** | **Review articles, conference papers** |
| **349** | **Nct et al** | **Safety and Pharmacokinetics (PK) of a Polyurethane Tenofovir Disoproxil Fumarate (TDF) Vaginal Ring.** | **[NCT02006264](https://clinicaltrials.gov/show/NCT02006264)** | **Excluded** | **Review articles, conference papers** |
| **350** | **Nct et al** | **Specialized Centers of Research (SCOR) on Sex Differences - Kyphosis Study.** | **[NCT01766674](https://clinicaltrials.gov/show/NCT01766674)** | **Excluded** | **Review articles, conference papers** |
| **351** | **Nct et al** | **Study to Explore the Effects of Probiotics on Endotoxin Levels in Type 2 Diabetes Mellitus Patients.** | **[NCT01765517](https://clinicaltrials.gov/show/NCT01765517)** | **Excluded** | **Review articles, conference papers** |
| **352** | **Nct et al** | **Use of the LRU Pillow in the Acute Setting Following Total Knee Arthroplasty.** | **[NCT01814033](https://clinicaltrials.gov/show/NCT01814033)** | **Excluded** | **Review articles, conference papers** |
| **353** | **Nct et al** | **Weekly vs Daily Teriparatide Therapy in Severe Postmenopausal Osteoporosis.** | **[NCT01** | **Excluded** | **Review articles, conference papers** |
| **354** | **Nct et al** | **Analysis of Neuromuscular Response, Postural Balance and Quality of Life of Diabetics Type 2 After Sensory-motor Training: blind Random Controled Clinical Trial.** | **[NCT01861392](https://clinicaltrials.gov/show/NCT01861392)** | **Excluded** | **Review articles, conference papers** |
| **355** | **Nct et al** | **Diagnosis of Patients With Low or Intermediate Suspicion of SAHS or With Comorbidity: standard Laboratory Polysomnography Compared With Three Nights of Home Respiratory Polygraphy.** | **[NCT01820156](https://clinicaltrials.gov/show/NCT01820156)** | **Excluded** | **Review articles, conference papers** |
| **356** | **Nct et al** | **The Effect of n-3 Polyunsaturated Fatty Acids in Patients With Psoriatic Arthritis.** | **[NCT01818804](https://clinicaltrials.gov/show/NCT01818804)** | **Excluded** | **Review articles, conference papers** |
| **357** | **Nct et al** | **The Effects of Therapeutic Resources on Structure and Function of Normal and Burned Skin.** | **[NCT02185950](https://clinicaltrials.gov/show/NCT02185950)** | **Excluded** | **Review articles, conference papers** |
| **358** | **Nct et al** | **Exercise-induced Bronchoconstriction in School Children.** | **[NCT01798823](https://clinicaltrials.gov/show/NCT01798823)** | **Excluded** | **Review articles, conference papers** |
| **359** | **Nct et al** | **Feasibility of a Trial of Laparoscopic Hysterectomy Versus Laparoscopic Sub-Total Hysterectomy.** | **[NCT01825915](https://clinicaltrials.gov/show/NCT01825915)** | **Excluded** | **Review articles, conference papers** |
| **360** | **Nct et al** | **High Dose Omega-3 Fatty Acids in the Treatment of Sport Related Concussions.** | **[NCT01814527](https://clinicaltrials.gov/ct2/show/NCT01814527)** | **Excluded** | **Review articles, conference papers** |
| **361** | **Nct et al** | **Pre-operative Diet: effect of Wound Healing After Bariatric Surgery.** | **[NCT01950052](https://clinicaltrials.gov/show/NCT01950052)** | **Excluded** | **Review articles, conference papers** |
| **362** | **Nct et al** | **Role of Vitamin D in the Prevention and Treatment of Diseases Associated With Insulin Resistance.** | **[NCT01779908](https://clinicaltrials.gov/show/NCT01779908)** | **Excluded** | **Review articles, conference papers** |
| **363** | **Nct et al** | **Saxagliptin + Metformin Compared to Saxagliptin or Metformin Monotherapy in PCOS Women With Impaired Glucose Homeostasis.** | **[NCT02022007](https://clinicaltrials.gov/show/NCT02022007)** | **Excluded** | **Review articles, conference papers** |
| **364** | **Nct et al** | **Amino Acids in Ileal Pouch-anal Anastomosis for Ulcerative Colitis.** | **[NCT02084550](https://clinicaltrials.gov/show/NCT02084550)** | **Excluded** | **Review articles, conference papers** |
| **365** | **Nct et al** | **Aquatic and Land Physical Training Effects on Cardiometabolic Risk Factors in Adolescents With Overweight and Obesity.** | **[NCT02309034](https://clinicaltrials.gov/show/NCT02309034)** | **Excluded** | **Review articles, conference papers** |
| **366** | **Nct et al** | **the Canadian Health Improvement Network to Upgrade Prevention Services.** | **[NCT02310685](https://clinicaltrials.gov/show/NCT02310685)** | **Excluded** | **Review articles, conference papers** |
| **367** | **Nct et al** | **Does Addition of a Functional Knee Brace Improve Rehabilitation Outcome in Subjects With Osteoarthritis of the Knee?** | **[NCT02712710](https://clinicaltrials.gov/show/NCT02712710)** | **Excluded** | **Review articles, conference papers** |
| **368** | **Nct et al** | **Effect of a Multimodal Pain Regimen on Pain Control, Patient Satisfaction and Narcotic Use in Orthopaedic Trauma Patients.** | **[NCT02160301](https://clinicaltrials.gov/show/NCT02160301)** | **Excluded** | **Review articles, conference papers** |
| **369** | **Nct et al** | **The Effect of Protein and Resistance Training on Muscle Mass in Acutely Ill Old Medical Patients.** | **[NCT02077491](https://clinicaltrials.gov/show/NCT02077491)** | **Excluded** | **Review articles, conference papers** |
| **370** | **Nct et al** | **Effectiveness of Aquatic Physical Therapy for Knee Osteoarthritis Patients.** | **[NCT02247882](https://clinicaltrials.gov/show/NCT02247882)** | **Excluded** | **Review articles, conference papers** |
| **371** | **Nct et al** | **Electrical Stimulation and Vaginal Palpation in Pelvic Floor Muscles Awareness.** | **[NCT02062242](https://clinicaltrials.gov/show/NCT02062242)** | **Excluded** | **Review articles, conference papers** |
| **372** | **Nct et al** | **Identification of Prognostic Indicators for Rehabilitation in Chronic Nonspecific Low Back Pain Patients.** | **[NCT02063503](https://clinicaltrials.gov/show/NCT02063503)** | **Excluded** | **Review articles, conference papers** |
| **373** | **Nct et al** | **IR and Microvascular Blood Flow in SCI.** | **[NCT02034331](https://clinicaltrials.gov/show/NCT02034331)** | **Excluded** | **Review articles, conference papers** |
| **374** | **Nct et al** | **Ketorolac Versus Triamcinolone Knee Injections for Osteoarthritis.** | **[NCT02295189](https://clinicaltrials.gov/show/NCT02295189)** | **Excluded** | **Review articles, conference papers** |
| **375** | **Nct et al** | **Lovenox 30 mg Twice Daily (BID) Versus 40 mg Once Daily (QD).** | **[NCT02342444](https://clinicaltrials.gov/ct2/show/NCT02342444)** | **Excluded** | **Review articles, conference papers** |
| **376** | **Nct et al** | **Lumbar Manipulation and Exercise for the Treatment of Acute Low Back Pain in Adolescents.** | **[NCT02111278](https://clinicaltrials.gov/show/NCT02111278)** | **Excluded** | **Review articles, conference papers** |
| **377** | **Nct et al** | **Mesenchymal Stem Cells in Knee Cartilage Injuries.** | **[NCT02118519](https://clinicaltrials.gov/show/NCT02118519)** | **Excluded** | **Review articles, conference papers** |
| **378** | **Nct et al** | **Nerve Block Washout.** | **[NCT02050295](https://clinicaltrials.gov/show/NCT02050295)** | **Excluded** | **Review articles, conference papers** |
| **379** | **Nct et al** | **Non-Ablative Fractional Resurfacing of Total Knee Replacement Scars.** | **[NCT02166138](https://clinicaltrials.gov/show/NCT02166138)** | **Excluded** | **Review articles, conference papers** |
| **380** | **Nct et al** | **Preheated Skin Disinfection vs Room-temperature on Bacterial Colonization During Pace Device Implantation.** | **[NCT02260479](https://clinicaltrials.gov/show/NCT02260479)** | **Excluded** | **Review articles, conference papers** |
| **381** | **Nct et al** | **Singapore INfra-Genicular Angioplasty With PAclitaxel-eluting Balloon for Critical Limb Ischaemia (SINGA-PACLI) Trial.** | **[NCT02129634](https://clinicaltrials.gov/show/NCT02129634)** | **Excluded** | **Review articles, conference papers** |
| **382** | **Nct et al** | **Staying Positive With Arthritis Study.** | **[NCT02223858](https://clinicaltrials.gov/show/NCT02223858)** | **Excluded** | **Review articles, conference papers** |
| **383** | **Nct et al** | **Teriparatide for Fracture Repair in Humans.** | **[NCT02091492](https://clinicaltrials.gov/show/NCT02091492)** | **Excluded** | **Review articles, conference papers** |
| **384** | **Nct et al** | **TKA Using Patient** |  | **Excluded** | **Review articles, conference papers** |
| **385** | **Nct et al** | **TKA Using Patient-Specific Instrumentation.** | **[NCT03117959](https://clinicaltrials.gov/show/NCT03117959)** | **Excluded** | **Review articles, conference papers** |
| **386** | **Nct et al** | **Trial of Feedback on Blood Use.** | **[NCT02232568](https://clinicaltrials.gov/show/NCT02232568)** | **Excluded** | **Review articles, conference papers** |
| **387** | **Nct et al** | **Use of Metformin in Treatment of Childhood Obesity.** | **[NCT02274948](https://clinicaltrials.gov/show/NCT02274948)** | **Excluded** | **Review articles, conference papers** |
| **388** | **Nct et al** | **Diabetes & Vardenafil.** | **[NCT02219646](https://clinicaltrials.gov/show/NCT02219646)** | **Excluded** | **Review articles, conference papers** |
| **389** | **Nct et al** | **Effect of Volume Training on Back Endurance.** | **[NCT02326792](https://clinicaltrials.gov/show/NCT02326792)** | **Excluded** | **Review articles, conference papers** |
| **390** | **Nct et al** | **Effects of Desflurane Versus Propofol on Hemostasis During Splenectomy.** | **[NCT02079064](https://clinicaltrials.gov/show/NCT02079064)** | **Excluded** | **Review articles, conference papers** |
| **391** | **Nct et al** | **The Effects of Honey, as a Dietary Supplement in Children With Hepatitis A.** | **[NCT02300792](https://clinicaltrials.gov/show/NCT02300792)** | **Excluded** | **Review articles, conference papers** |
| **392** | **Nct et al** | **Effects of Robot-Assisted Combined Therapy in Upper Limb Rehabilitation in Stroke Patients.** | **[NCT02319785](https://clinicaltrials.gov/show/NCT02319785)** | **Excluded** | **Review articles, conference papers** |
| **393** | **Nct et al** | **Electrical Stimulation of the Paretic Upper Limb in the Early Stroke Phase.** | **[NCT02250365](https://clinicaltrials.gov/show/NCT02250365)** | **Excluded** | **Review articles, conference papers** |
| **394** | **Nct et al** | **Family Milk Product Two-year Dose-response Study to Enhance Bone Health.** | **[NCT02236871](https://clinicaltrials.gov/show/NCT02236871)** | **Excluded** | **Review articles, conference papers** |
| **395** | **Nct et al** | **Injection With OnabotulinumtoxinA (Botox) for the Treatment of Chronic Pelvic Pain.** | **[NCT02173405](https://clinicaltrials.gov/show/NCT02173405)** | **Excluded** | **Review articles, conference papers** |
| **396** | **Nct et al** | **Mechanisms of Arterial Hypotension in Chronic Spinal Cord Injury.** | **[NCT02154412](https://clinicaltrials.gov/ct2/show/NCT02154412)** | **Excluded** | **Review articles, conference papers** |
| **397** | **Nct et al** | **Motivational Interviewing as an Intervention for PCOS.** | **[NCT02924025](https://clinicaltrials.gov/show/NCT02924025)** | **Excluded** | **Review articles, conference papers** |
| **398** | **Nct et al** | **Motor Learning-based Wheelchair Propulsion Training for Older Adults.** | **[NCT02123043](https://clinicaltrials.gov/show/NCT02123043)** | **Excluded** | **Review articles, conference papers** |
| **399** | **Nct et al** | **Pre-operative Pregabalin and Ketamine to Prevent Phantom Pain.** | **[NCT02311777](https://clinicaltrials.gov/show/NCT02311777)** | **Excluded** | **Review articles, conference papers** |
| **400** | **Nct et al** | **Preschoolers Activity Trial.** | **[NCT02293278](https://clinicaltrials.gov/show/NCT02293278)** | **Excluded** | **Review articles, conference papers** |
| **401** | **Nct et al** | **Titanium vs Absorbable Tacks for Mesh Fixation in Laparoscopic Incisional and Ventral Hernia Repair.** | **[NCT02076984](https://clinicaltrials.gov/show/NCT02076984)** | **Excluded** | **Review articles, conference papers** |
| **402** | **Nct et al** | **Adductor Canal Catheter vs Local Infiltration of Analgesia for Total Knee Arthroplasty.** | **[NCT02603900](https://clinicaltrials.gov/show/NCT02603900)** | **Excluded** | **Review articles, conference papers** |
| **403** | **Nct et al** | **Assessment of a Knee Brace in Patients With Osteoarthritis.** | **[NCT02706106](https://clinicaltrials.gov/show/NCT02706106)** | **Excluded** | **Review articles, conference papers** |
| **404** | **Nct et al** | **Bracing to Treat Knee Osteoarthritis in Elderly.** | **[NCT02443974](https://clinicaltrials.gov/show/NCT02443974)** | **Excluded** | **Review articles, conference papers** |
| **405** | **Nct et al** | **The Combined Efficacy of Evicel and Tranexamic Acid on Total Knee Arthroplasty.** | **[NCT02553122](https://clinicaltrials.gov/show/NCT02553122)** | **Excluded** | **Review articles, conference papers** |
| **406** | **Nct et al** | **Dexmedetomidine for Sedation in Total Knee Replacements.** | **[NCT02466022](https://clinicaltrials.gov/show/NCT02466022)** | **Excluded** | **Review articles, conference papers** |
| **407** | **Nct et al** | **Dosing of Intra-articular Triamcinolone Hexacetonide for Knee Synovitis in Chronic Polyarthritis.** | **[NCT02437461](https://clinicaltrials.gov/show/NCT02437461)** | **Excluded** | **Review articles, conference papers** |
| **408** | **Nct et al** | **Effect of a Patient Education in Pain Coping for Patients Scheduled for Total Knee Arthroplasty.** | **[NCT02587429](https://clinicaltrials.gov/show/NCT02587429)** | **Excluded** | **Review articles, conference papers** |
| **409** | **Nct et al** | **Effect of Egoscue Corrective Exercise Prescription on Acute and Short-term Chronic Knee and Hip Pain.** | **[NCT02374034](https://clinicaltrials.gov/show/NCT02374034)** | **Excluded** | **Review articles, conference papers** |
| **410** | **Nct et al** | **Extract of Curcuma Longa Complexed With Phosphatidilcholine (Motore®) in the Treatment of Adults With Knee Osteoarthritis.** | **[NCT02409381](https://clinicaltrials.gov/show/NCT02409381)** | **Excluded** | **Review articles, conference papers** |
| **411** | **Nct et al** | **Finnish Unicompartmental and Total Knee Arthroplasty Investigation.** | **[NCT02481427](https://clinicaltrials.gov/show/NCT02481427)** | **Excluded** | **Review articles, conference papers** |
| **412** | **Nct et al** | **Impact of Resistance Training-Protein Supplementation on Lean Muscle Mass in Childhood Cancer Survivors.** | **[NCT02501460](https://clinicaltrials.gov/show/NCT02501460)** | **Excluded** | **Review articles, conference papers** |
| **413** | **Nct et al** | **Improving Patient Understanding of the Surgical Hospital Experience: use of YouTube Video Playlist.** | **[NCT02546180](https://clinicaltrials.gov/show/NCT02546180)** | **Excluded** | **Review articles, conference papers** |
| **414** | **Nct et al** | **Intra-articular Morphine and Clonidine Injections for Pain Management in Hip Arthroscopy.** | **[NCT02530151](https://clinicaltrials.gov/show/NCT02530151)** | **Excluded** | **Review articles, conference papers** |
| **415** | **Nct et al** | **NEO6860, a TRPV1 Antagonist, First in Human Study.** | **[NCT02337543](https://clinicaltrials.gov/show/NCT02337543)** | **Excluded** | **Review articles, conference papers** |
| **416** | **Nct et al** | **Pulmonary Rehabilitation Program and PROactive Tool.** | **[NCT02437994](https://clinicaltrials.gov/show/NCT02437994)** | **Excluded** | **Review articles, conference papers** |
| **417** | **Nct et al** | **The Rehabilitation Outcome of Patients With Lumbar Spondylolisthesis. A Prospective Randomized Study.** | **[NCT02435485](https://clinicaltrials.gov/show/NCT02435485)** | **Excluded** | **Review articles, conference papers** |
| **418** | **Nct et al** | **Treatment of Keratoconus With Advanced Corneal Crosslinking.** | **[NCT02425150](https://clinicaltrials.gov/show/NCT02425150)** | **Excluded** | **Review articles, conference papers** |
| **419** | **Nct et al** | **A Trial Comparing Adductor Canal Catheter and In** |  | **Excluded** | **Review articles, conference papers** |
| **420** | **Nct et al** | **A Trial Comparing Adductor Canal Catheter and Intraarticular Catheter Following Primary Total Knee Arthroplasty.** | **[NCT02497911](https://clinicaltrials.gov/show/NCT02497911)** | **Excluded** | **Review articles, conference papers** |
| **421** | **Nct et al** | **Unicondylar Knee Arthroplasty Versus Total Knee Arthroplasty in Patients With Anteromedial Osteoarthritis of the Knee.** | **[NCT02430129](https://clinicaltrials.gov/ct2/show/NCT02430129)** | **Excluded** | **Review articles, conference papers** |
| **422** | **Nct et al** | **Adductor Canal Block Versus Femoral Nerve Block for Analgesia After Anterior Cruciate Ligament Reconstruction Arthroscopically** | **[https://clinicaltrials.gov/show/NCT02355093](https://clinicaltrials.gov/show/NCT02355093)** | **Excluded** | **Review articles, conference papers** |
| **423** | **Nct et al** | **Analyzing the Effect of Trunk Training on Limbs in Children With Spastic Cerebral Palsy** | **[https://clinicaltrials.gov/show/NCT02643160](https://clinicaltrials.gov/show/NCT02643160)** | **Excluded** | **Review articles, conference papers** |
| **424** | **Nct et al** | **Back School or Brain School for Patients Undergoing Surgery for Lumbar Radiculopathy?** | **[https://clinicaltrials.gov/show/NCT02630732](https://clinicaltrials.gov/show/NCT02630732)** | **Excluded** | **Review articles, conference papers** |
| **425** | **Nct et al** | **A Biofeedback Training in Schoolchildren With an Attention-Deficit/Hyperactivity Disorder (ADHD)** | **[https://clinicaltrials.gov/show/NCT02572180](https://clinicaltrials.gov/show/NCT02572180)** | **Excluded** | **Review articles, conference papers** |
| **426** | **Nct et al** | **Bone Density in Children With IBD Treated With Amorphous Calcium or Commercial Crystalline Calcium** | **[https://clinicaltrials.gov/show/NCT02470663](https://clinicaltrials.gov/show/NCT02470663)** | **Excluded** | **Review articles, conference papers** |
| **427** | **Nct et al** | **Coalition for a Healthier Community—Utah Women and Girls - Phase II (UWAG-II)** | **[https://clinicaltrials.gov/show/NCT02470156](https://clinicaltrials.gov/show/NCT02470156)** | **Excluded** | **Review articles, conference papers** |
| **428** | **Nct et al** | **COcoa Supplement and Multivitamin Outcomes Study** | **[https://clinicaltrials.gov/ct2/show/NCT02422745](https://clinicaltrials.gov/ct2/show/NCT02422745)** | **Excluded** | **Review articles, conference papers** |
| **429** | **Nct et al** | **Community-based Sport Programme and Type 2 Diabetes** | **[https://clinicaltrials.gov/show/NCT02548702](https://clinicaltrials.gov/show/NCT02548702)** | **Excluded** | **Review articles, conference papers** |
| **430** | **Nct et al** | **A Comparison of TCC-EZ Using Human Amnion Allograft vs TCC-EZ and Standard Wound Care in Treating Diabetic Foot Ulcers** | **[https://clinicaltrials.gov/show/NCT02344329](https://clinicaltrials.gov/show/NCT02344329)** | **Excluded** | **Review articles, conference papers** |
| **431** | **Nct et al** | **Continuous Adductor Canal Block (CACB) vs. Combined Spinal Epidural (CSE) in Total Knee Arthroplasty** | **[https://clinicaltrials.gov/show/NCT02415465](https://clinicaltrials.gov/show/NCT02415465)** | **Excluded** | **Review articles, conference papers** |
| **432** | **Nct et al** | **Effect of Pursed Lips Breathing on Chest Wall, Mobility Diaphragmatic and Exercise Tolerance in Patients With Chronic Obstructive Pulmonary Disease** | **[https://clinicaltrials.gov/show/NCT02424331](https://clinicaltrials.gov/show/NCT02424331)** | **Excluded** | **Review articles, conference papers** |
| **433** | **Nct et al** | **Effect of the Glycemic Index of Diet on Metabolic and Reproductive Parameters in Overweight and Obese Infertile Women** | **[https://clinicaltrials.gov/show/NCT02416960](https://clinicaltrials.gov/show/NCT02416960)** | **Excluded** | **Review articles, conference papers** |
| **434** | **Nct et al** | **Effects of Catheter Location on Postoperative Analgesia for Continuous Adductor Canal and Popliteal-Sciatic Nerve Blocks** | **[https://clinicaltrials.gov/show/NCT02523235](https://clinicaltrials.gov/show/NCT02523235)** | **Excluded** | **Review articles, conference papers** |
| **435** | **Nct et al** | **Effects of Simultaneous Balance and Resistance Training in Older Adults** | **[https://clinicaltrials.gov/show/NCT02555033](https://clinicaltrials.gov/show/NCT02555033)** | **Excluded** | **Review articles, conference papers** |
| **436** | **Nct et al** | **External Validity of a Randomized Trial in Patients With a Femoral Neck Fracture** | **[https://clinicaltrials.gov/show/NCT02362971](https://clinicaltrials.gov/show/NCT02362971)** | **Excluded** | **Review articles, conference papers** |
| **437** | **Nct et al** | **Flowchart-Assisted Resuscitation. Standard CPR vs. Chest Compressions Only: what Happened to the Quality?** | **[https://clinicaltrials.gov/show/NCT02438527](https://clinicaltrials.gov/show/NCT02438527)** | **Excluded** | **Review articles, conference papers** |
| **438** | **Nct et al** | **Immunonutrition in Total Hip Arthroplasty** | **[https://clinicaltrials.gov/show/NCT02580214](https://clinicaltrials.gov/show/NCT02580214)** | **Excluded** | **Review articles, conference papers** |
| **439** | **Nct et al** | **Individualized Comprehensive Home-Centred Activity Based Therapy for Children With Diplegic Cerebral Palsy** | **[https://clinicaltrials.gov/show/NCT02412007](https://clinicaltrials.gov/show/NCT02412007)** | **Excluded** | **Review articles, conference papers** |
| **440** | **Nct et al** | **Intervention Study - Effectiveness of Smartphone Delivered Interval Walking Training to Patients With Type 2 Diabetes** | **[https://clinicaltrials.gov/show/NCT02341690](https://clinicaltrials.gov/show/NCT02341690)** | **Excluded** | **Review articles, conference papers** |
| **441** | **Nct et al** | **Longjohn Amgen Study - Effect of Denosumab** | **[https://clinicaltrials.gov/show/NCT02444585](https://clinicaltrials.gov/show/NCT02444585)** | **Excluded** | **Review articles, conference papers** |
| **442** | **Nct et al** | **Non-surgical Periodontal Therapy and Myo-inositol in Polycystic Ovary Syndrome Women Having Chronic Periodontitis** | **[https://clinicaltrials.gov/show/NCT02633462](https://clinicaltrials.gov/show/NCT02633462)** | **Excluded** | **Review articles, conference papers** |
| **443** | **Nct et al** | **PAracetamol and NSAID in Combination: a Randomised, Blinded, Parallel, 4-group Clinical Trial** | **[https://clinicaltrials.gov/show/NCT02571361](https://clinicaltrials.gov/show/NCT02571361)** | **Excluded** | **Review articles, conference papers** |
| **444** | **Nct et al** | **Pre-op Femoral Nerve Block for Hip Fracture** | **[https://clinicaltrials.gov/show/NCT02450045](https://clinicaltrials.gov/show/NCT02450045)** | **Excluded** | **Review articles, conference papers** |
| **445** | **Nct et al** | **The RCT of Acupuncture on PCOS Combined With IR** | **[https://clinicaltrials.gov/show/NCT02491333](https://clinicaltrials.gov/show/NCT02491333)** | **Excluded** | **Review articles, conference papers** |
| **446** | **Nct et al** | **Renal Osteodystrophy: an Individual Management Approach** | **[https://clinicaltrials.gov/show/NCT02440581](https://clinicaltrials.gov/show/NCT02440581)** | **Excluded** | **Review articles, conference papers** |
| **447** | **Nct et al** | **Training With Virtual Reality in Upper Arm Reaching of Children With Cerebral Palsy** | **[https://clinicaltrials.gov/show/NCT04483388](https://clinicaltrials.gov/show/NCT04483388)** | **Excluded** | **Review articles, conference papers** |
| **448** | **Nct et al** | **Treatment of Keratoconus With Advanced CXL-II** | **[https://clinicaltrials.gov/show/NCT02514200](https://clinicaltrials.gov/show/NCT02514200)** | **Excluded** | **Review articles, conference papers** |
| **449** | **Nct et al** | **Trunk Restraint Therapy in Post-stroke Patients** | **[https://clinicaltrials.gov/show/NCT02364141](https://clinicaltrials.gov/show/NCT02364141)** | **Excluded** | **Review articles, conference papers** |
| **450** | **Nct et al** | **Yoga's Effect on Fall Risk Factors in the Rural, Older Adult Population; an Academic/Community Partnership** | **[https://clinicaltrials.gov/show/NCT02443038](https://clinicaltrials.gov/show/NCT02** | **Excluded** | **Review articles, conference papers** |
| **451** | **Nct et al** | **Comparison of Two Manual Therapy Techniques on Ankle Dorsiflexion** | **[https://clinicaltrials.gov/show/NCT02653807](https://clinicaltrials.gov/show/NCT02653807)** | **Excluded** | **Review articles, conference papers** |
| **452** | **Nct et al** | **The Effect of Mobilization With Movement on Pain and Function Among Patients With Knee Osteoarthritis** | **[https://clinicaltrials.gov/show/NCT02865252](https://clinicaltrials.gov/show/NCT02865252)** | **Excluded** | **Review articles, conference papers** |
| **453** | **Nct et al** | **Effects of Two Different Types of Ankle Foot Orthoses on Gait Outcomes in Patients With Subacute Stroke** | **[https://clinicaltrials.gov/show/NCT02693834](https://clinicaltrials.gov/show/NCT02693834)** | **Excluded** | **Review articles, conference papers** |
| **454** | **Nct et al** | **The Results of Oxford Unicompartmental Knee Arthroplasty in Patients With and Without Preoperative Genu Recurvatum** | **[https://clinicaltrials.gov/show/NCT02854189](https://clinicaltrials.gov/show/NCT02854189)** | **Excluded** | **Review articles, conference papers** |
| **455** | **Nct** | **Adductor Canal Block (ACB) Before and After Primary Total Knee Arthroplasty (TKA)** | **[NCT02908711](https://clinicaltrials.gov/show/NCT02908711)** | **Excluded** | **Review articles, conference papers** |
| **456** | **Nct** | **BALTiC Study: a Feasibility Analysis of Home Based BALance Training in People With Charcot-Marie-Tooth Disease** | **[NCT02982343](https://clinicaltrials.gov/show/NCT02982343)** | **Excluded** | **Review articles, conference papers** |
| **457** | **Nct** | **Chronic Effects Using Light-Emitting Diode Therapy (LEDT) During a Resistance Exercise Protocol for Asthmatic Patients** | **[NCT03112239](https://clinicaltrials.gov/show/NCT03112239)** | **Excluded** | **Review articles, conference papers** |
| **458** | **Nct** | **Clinical Trial to Evaluate the Adjuvant Effect of Shock Wave Therapy in the Insertional Achilles Tendinopathy** | **[NCT02757664](https://clinicaltrials.gov/show/NCT02757664)** | **Excluded** | **Review articles, conference papers** |
| **459** | **Nct** | **A Comparison of Two Treatments for CRPS and Changes in Resting-State Connectivity of Cerebral Networks** | **[NCT02753335](https://clinicaltrials.gov/show/NCT02753335)** | **Excluded** | **Review articles, conference papers** |
| **460** | **Nct** | **Continuous Versus Single-Shot Adductor Canal Block in Total Knee Arthroplasty** | **[NCT03145584](https://clinicaltrials.gov/show/NCT03145584)** | **Excluded** | **Review articles, conference papers** |
| **461** | **Nct** | **Early Prevention of Excessive Gestational Weight Gain Using Lifestyle Change** | **[NCT02804061](https://clinicaltrials.gov/show/NCT02804061)** | **Excluded** | **Review articles, conference papers** |
| **462** | **Nct** | **The Effect of A New Perioperative Practice Model on Patient, Nursing And Organisational Outcomes** | **[NCT02906033](https://clinicaltrials.gov/show/NCT02906033)** | **Excluded** | **Review articles, conference papers** |
| **463** | **Nct** | **Effect of Antihypertensive Agents on Diastolic Function in Patients With Sleep Apnea** | **[NCT02896621](https://clinicaltrials.gov/show/NCT02896621)** | **Excluded** | **Review articles, conference papers** |
| **464** | **Nct** | **The Effect of Cryotherapy in Pain Control, Function and Quality of Life in Individuals With Knee Osteoarthritis** | **[NCT02725047](https://clinicaltrials.gov/show/NCT02725047)** | **Excluded** | **Review articles, conference papers** |
| **465** | **Nct** | **Effect of Gabapentin Enacarbil on Opioid Consumption and Pain Scores** | **[NCT02840240](https://clinicaltrials.gov/ct2/show/NCT02840240)** | **Excluded** | **Review articles, conference papers** |
| **466** | **Nct** | **Effect of High Protein Weight Loss for Seniors** | **[NCT02730988](https://clinicaltrials.gov/show/NCT02730988)** | **Excluded** | **Review articles, conference papers** |
| **467** | **Nct** | **Effect of the Pelvic Loin Musculature Training on the Dynamic Hip Stabilization** | **[NCT02739490](https://clinicaltrials.gov/show/NCT02739490)** | **Excluded** | **Review articles, conference papers** |
| **468** | **Nct** | **Effectiveness of Fascia Iliaca Nerve Block for Post Hip Arthroscopy** | **[NCT02717728](https://clinicaltrials.gov/show/NCT02717728)** | **Excluded** | **Review articles, conference papers** |
| **469** | **Nct** | **Effectiveness of the Pilates Method Versus Aerobic Exercises in Elderly With Low Back Pain** | **[NCT02729779](https://clinicaltrials.gov/show/NCT02729779)** | **Excluded** | **Review articles, conference papers** |
| **470** | **Nct** | **Effects of Diet on Brain Processing** | **[NCT02835820](https://clinicaltrials.gov/show/NCT02835820)** | **Excluded** | **Review articles, conference papers** |
| **471** | **Nct** | **Effects of Supplementation of Vitamin D in Patients With Crohn`s Disease** | **[NCT02704624](https://clinicaltrials.gov/show/NCT02704624)** | **Excluded** | **Review articles, conference papers** |
| **472** | **Nct** | **Efficacy of Near Infrared Phototherapy in Type 2 Diabetic Neuropathy** | **[NCT02798393](https://clinicaltrials.gov/show/NCT02798393)** | **Excluded** | **Review articles, conference papers** |
| **473** | **Nct** | **Feasibility of the NEXT Steps Weight Loss Intervention +/- Resistance Training for Endometrial Cancer Survivors: effect on Lean Mass & Biomarkers** | **[NCT02774759](https://clinicaltrials.gov/ct2/show/NCT02774759)** | **Excluded** | **Review articles, conference papers** |
| **474** | **Nct** | **The Impact of Disclosing Personalized Depression Risk Information on High-risk Individuals' Outcomes** | **[NCT02943876](https://clinicaltrials.gov/show/NCT02943876)** | **Excluded** | **Review articles, conference papers** |
| **475** | **Nct** | **The Impact of Low-fat and Full-fat Dairy Consumption on Glucose Homeostasis (DAIRY Study)** | **[NCT02663544](https://clinicaltrials.gov/show/NCT02663544)** | **Excluded** | **Review articles, conference papers** |
| **476** | **Nct** | **An Investigation Into the Role of Walking in Treating the Symptoms of Knee Osteoarthritis: the WalkOut Study** | **[NCT02748291](https://clinicaltrials.gov/show/NCT02748291)** | **Excluded** | **Review articles, conference papers** |
| **477** | **Nct** | **Mindful Eating and Living for Obese Women** | **[NCT02753972](https://clinicaltrials.gov/ct2/show/NCT02753972)** | **Excluded** | **Review articles, conference papers** |
| **478** | **Nct** | **Moderated Blood Flow Restriction After Anterior Cruciate Ligament Reconstruction** | **[NCT02911909](https://clinicaltrials.gov/show/NCT02911909)** | **Excluded** | **Review articles, conference papers** |
| **479** | **Nct** | **Polarity Action in Electrical Stimulation Transcutaneous Donors for Treatment Areas Burned Patients** | **[NCT02679703](https://clinicaltrials.gov/show/NCT02679703)** | **Excluded** | **Review articles, conference papers** |
| **480** | **Nct** | **Quality of Recovery After General or Spinal Anesthesia for Inguinal Hernia Repair** | **[NCT02696122](https://clinicaltrials.gov/show/NCT02696122)** | **Excluded** | **Review articles, conference papers** |
| **481** | **Nct** | **RISCAID Study: remote ISchemic Conditioning for Angiopathy In Diabetes** | **[NCT02749942](https://clinicaltrials.gov/show/NCT02749942)** | **Excluded** | **Review articles, conference papers** |
| **482** | **Nct** | **Single Injection Adductor Canal Block vs Catheter for Total Knee Arthroplasty** | **[NCT02798835](https://clinicaltrials.gov/show/NCT02798835)** | **Excluded** | **Review articles, conference papers** |
| **483** | **Nct** | **TENS Self-applied in the Complementary Treatment of Deep Endometriosis** | **[NCT02769052](https://clinicaltrials.gov/show/NCT02769052)** | **Excluded** | **Review articles, conference papers** |
| **484** | **Nct** | **Text Messages and Blood Pressure Control** | **[NCT02779231](https://clinicaltrials.gov/show/NCT02779231)** | **Excluded** | **Review articles, conference papers** |
| **485** | **Nct** | **Peripheral Nerve Blocks for Major Lower Extremity Amputations** | **[NCT03174782](https://clinicaltrials.gov/show/NCT03174782)** | **Excluded** | **Review articles, conference papers** |
| **486** | **Nct** | **Platelet Rich Plasma (PRP) Peri-urethral and Clitoral Injections for the Treatment of Female Orgasmic Disorder** | **[NCT03189238](https://clinicaltrials.gov/show/NCT03189238)** | **Excluded** | **Review articles, conference papers** |
| **487** | **Nct** | **The Spraino Pilot Trial** | **[NCT03311490** | **Excluded** | **Review articles, conference papers** |
| **488** | **Nct** | **Adding Ketamine to Low Dose Bupivacaine in Saddle Block for Perianal Surgery** | **[NCT03264430](https://clinicaltrials.gov/show/NCT03264430)** | **Excluded** | **Review articles, conference papers** |
| **489** | **Nct** | **The Analgesic Efficacy of Ultrasound Guided Transversus Abdominal Plane Block After Abdominal Cancer Surgeries** | **[NCT03165383](https://clinicaltrials.gov/show/NCT03165383)** | **Excluded** | **Review articles, conference papers** |
| **490** | **Nct** | **Analysis of the Effects of LED Phototherapy and Electrical Stimulation in the Healing of Diabetic Ulcers** | **[NCT03250533](https://clinicaltrials.gov/show/NCT03250533)** | **Excluded** | **Review articles, conference papers** |
| **491** | **Nct** | **Botulinum for Chronic Exertional Compartment Syndrome** | **[NCT03339921](https://clinicaltrials.gov/ct2/show/NCT03339921)** | **Excluded** | **Review articles, conference papers** |
| **492** | **Nct** | **Cadence Modulation to Improve Well-Being, Kinematics and Aerobic Performance in Cyclists** | **[NCT03482726](https://clinicaltrials.gov/show/NCT03482726)** | **Excluded** | **Review articles, conference papers** |
| **493** | **Nct** | **Comparison of Functional Recovery Between Mobile Bearing Unicompartmental Knee Arthroplasty and Total Knee Arthroplasty** | **[NCT04419129](https://clinicaltrials.gov/show/NCT04419129)** | **Excluded** | **Review articles, conference papers** |
| **494** | **Nct** | **CREST: capsular Repair During Hip Arthroscopy** | **[NCT03372564](https://clinicaltrials.gov/ct2/show/NCT03372564)** | **Excluded** | **Review articles, conference papers** |
| **495** | **Nct** | **Cryotherapy Associated With Exercise in Pain Control and Physical Function in Individuals With Knee Osteoarthritis** | **[NCT03360500](https://clinicaltrials.gov/ct2/show/NCT03360500)** | **Excluded** | **Review articles, conference papers** |
| **496** | **Nct** | **Does Depth of Neuromuscular Blockade (NMB) Affect Surgical Conditions in Obese Patients Undergoing Robotic Surgery** | **[NCT03591289](https://clinicaltrials.gov/show/NCT03591289)** | **Excluded** | **Review articles, conference papers** |
| **497** | **Nct** | **Effect of Probiotics on Pre-diabetes and Diabetes in China** | **[NCT03377946](https://clinicaltrials.gov/show/NCT03377946)** | **Excluded** | **Review articles, conference papers** |
| **498** | **Nct** | **Effects of Insoles in Patients With Rheumatoid Arthritis: randomized Controlled Trial** | **[NCT03170947](https://clinicaltrials.gov/show/NCT03170947)** | **Excluded** | **Review articles, conference papers** |
| **499** | **Nct** | **Effects of Nordic Walking in Parkinson Disease Patients** | **[NCT03355521](https://clinicaltrials.gov/show/NCT03355521)** | **Excluded** | **Review articles, conference papers** |
| **500** | **Nct** | **Effects of Using Customized Insoles With Anterior or Posterior Plantar Support in Cross-pelvis Syndrome and Perception of Body Alignment in Young Adults: a Randomized Clinical Trial** | **[NCT03350867](https://clinicaltrials.gov/show/NCT03350867)** | **Excluded** | **Review articles, conference papers** |
| **501** | **Nct** | **Efficacy of Multimodal Analgesia Following Hip Arthroscopy** | **[NCT03351439](https://clinicaltrials.gov/show/NCT03351439)** | **Excluded** | **Review articles, conference papers** |
| **502** | **Nct** | **Evaluation Effect of Crocina on The Cellular Immune Responses in Osteoarthritis Patients** | **[NCT03375814](https://clinicaltrials.gov/show/NCT03375814)** | **Excluded** | **Review articles, conference papers** |
| **503** | **Nct** | **Immediate and Chronic Effects of Whole-Body Vibration on Neuromuscular Performance and Postural Control in Elderly** | **[NCT03253042](https://clinicaltrials.gov/show/NCT03253042)** | **Excluded** | **Review articles, conference papers** |
| **504** | **Nct** | **Immediate Effects of Whole-Body Vibration on Neuromuscular Performance and Postural Control in Elderly** | **[NCT03356418](https://clinicaltrials.gov/show/NCT03356418)** | **Excluded** | **Review articles, conference papers** |
| **505** | **Nct** | **Inotuzumab Ozogamicin and Frontline Chemotherapy in Treating Young Adults With Newly Diagnosed B Acute Lymphoblastic Leukemia** | **[NCT03150693](https://clinicaltrials.gov/ct2/show/NCT03150693)** | **Excluded** | **Review articles, conference papers** |
| **506** | **Nct** | **Lifestyle Intervention in Preparation for Pregnancy (LIPP)** | **[NCT03146156](https://clinicaltrials.gov/ct2/show/NCT03146156)** | **Excluded** | **Review articles, conference papers** |
| **507** | **Nct** | **Lumbar Manipulation for Hip and Muscle Strength** | **[NCT03101956](https://clinicaltrials.gov/show/NCT03101956)** | **Excluded** | **Review articles, conference papers** |
| **508** | **Nct** | **Maraviroc to Augment Rehabilitation Outcomes After Stroke** | **[NCT03172026](https://clinicaltrials.gov/show/NCT03172026)** | **Excluded** | **Review articles, conference papers** |
| **509** | **Nct** | **Neuromuscular Intervention Targeted to Mechanisms of ACL Load in Female Athletes** | **[NCT03190889](https://clinicaltrials.gov/ct2/show/NCT03190889)** | **Excluded** | **Review articles, conference papers** |
| **510** | **Nct** | **Oral Tranexamic Acid vs. Oral Aminocaproic Acid to Reduce Blood Loss and Transfusion After Total Knee Replacement** | **[NCT03365999](https://clinicaltrials.gov/show/NCT03365999)** | **Excluded** | **Review articles, conference papers** |
| **511** | **Nct** | **Parabens Flocculation on the Anti Inflammatory Effects of Corticosteroid Injections for Total Knee Arthroplasty** | **[NCT03445611](https://clinicaltrials.gov/show/NCT03445611)** | **Excluded** | **Review articles, conference papers** |
| **512** | **Nct** | **Pilot Evaluation of the Effect of Riboflavin Supplementation on Blood Pressure and Possible Effect Modification by the MTHFR C677T Genotype** | **[NCT03151096](https://clinicaltrials.gov/show/NCT03151096)** | **Excluded** | **Review articles, conference papers** |
| **513** | **Nct** | **Postoperative Intervention Program Effectiveness in Hip Fracture Patients: a Randomized Clinical Trial** | **[NCT03156075](https://clinicaltrials.gov/show/NCT03156075)** | **Excluded** | **Review articles, conference papers** |
| **514** | **Nct** | **Post-operative Rehabilitation of Total Knee Arthroplasty With Applications on Smart Phone** | **[NCT03365427](https://clinicaltrials.gov/show/NCT03365427)** | **Excluded** | **Review articles, conference papers** |
| **515** | **Nct** | **Preemptive Tapentadol on Post-operative Analgesia Following Total Knee Arthroplasty** | **[NCT03351517](https://clinicaltrials.gov/show/NCT03351517)** | **Excluded** | **Review articles, conference papers** |
| **516** | **Nct** | **A Prospective Randomized Controlled Trial of Dual-Mobility Components in Primary THA** | **[NCT03371212](https://clinicaltrials.gov/ct2/show/NCT03371212)** | **Excluded** | **Review articles, conference papers** |
| **517** | **Nct** | **Psoas Sciatic Blockade for Knee Arthroplasty** | **[NCT03088371](https://clinicaltrials.gov/show/NCT03088371)** | **Excluded** | **Review articles, conference papers** |
| **518** | **Nct** | **Pulmonary Rehabilitation for Uncontrolled Asthma Associated With Elevated BMI** | **[NCT03630432](https://clinicaltrials.gov/show/NCT03630432)** | **Excluded** | **Review articles, conference papers** |
| **519** | **Nct** | **Visceral Mobilization and Functional Constipation in Stroke Survivors** | **[[NCT03031977](https://clinicaltrials.gov/show/NCT0303197](https://clinicaltrials.gov/show/NCT0303197)** | **Excluded** | **Review articles, conference papers** |
| **520** | **Nct** | **ZIMBA: Clinical Trial in Paediatric Obesity** | **[NCT03283813](https://clinicaltrials.gov/ct2/show/NCT03283813)** | **Excluded** | **Review articles, conference papers** |
| **521** | **Nct (2017)** | **The Effect Of An Expanded Long Term Periodization Exercise Training In Patients With Cardiovascular Disease** | **[https://clinicaltrials.gov/show/NCT03335319](https://clinicaltrials.gov/show/NCT03335319)** | **Excluded** | **Review articles, conference papers** |
| **522** | **Nct (2017)** | **Effects of Shoe Cushioning and Body Mass on Injury Risk in Running** | **[https://clinicaltrials.gov/show/NCT03115437](https://clinicaltrials.gov/show/NCT03115437)** | **Excluded** | **Review articles, conference papers** |
| **523** | **Nct (2017)** | **Evaluation of a Commercial Program on Weight Loss and Health Outcomes** | **[https://clinicaltrials.gov/show/NCT03037567](https://clinicaltrials.gov/show/NCT03037567)** | **Excluded** | **Review articles, conference papers** |
| **524** | **Nct (2017)** | **The Healthy School Start Plus Intervention Study** | **[https://clinicaltrials.gov/show/NCT03390725](https://clinicaltrials.gov/show/NCT03390725)** | **Excluded** | **Review articles, conference papers** |
| **525** | **Nct (2017)** | **Physiotherapy After Anterior Cervical Spine Surgery** | **[https://clinicaltrials.gov/ct2/show/NCT03036007](https://clinicaltrials.gov/ct2/show/NCT03036007)** | **Excluded** | **Review articles, conference papers** |
| **526** | **Nct (2018)** | **All-Polyethylene Tibias in TKA: PS vs CS Implants** | **[https://clinicaltrials.gov/show/NCT03569670](https://clinicaltrials.gov/show/NCT03569670)** | **Excluded** | **Review articles, conference papers** |
| **527** | **Nct (2018)** | **Analysis of Sensory Motor Training in Chronic Ankle Instability** | **[https://clinicaltrials.gov/show/NCT03768583](https://clinicaltrials.gov/show/NCT03768583)** | **Excluded** | **Review articles, conference papers** |
| **528** | **Nct (2018)** | **Arthroscopic Versus Open Brostrom for Ankle Instability** | **[https://clinicaltrials.gov/ct2/show/NCT03643926](https://clinicaltrials.gov/ct2/show/NCT03643926)** | **Excluded** | **Review articles, conference papers** |
| **529** | **Nct (2018)** | **Circuit Training in Children With Cerebral Palsy** | **[https://clinicaltrials.gov/show/NCT03529682](https://clinicaltrials.gov/show/NCT03529682)** | **Excluded** | **Review articles, conference papers** |
| **530** | **Nct (2018)** | **Clinical Outcomes of MP Persona vs. Persona Knee-PS** | **[https://clinicaltrials.gov/ct2/show/NCT03681977](https://clinicaltrials.gov/ct2/show/NCT03681977)** | **Excluded** | **Review articles, conference papers** |
| **531** | **Nct (2018)** | **Community-based Rehabilitation and Fall Prevention Program After Total Knee Arthroplasty** | **[https://clinicaltrials.gov/show/NCT03615638](https://clinicaltrials.gov/show/NCT03615638)** | **Excluded** | **Review articles, conference papers** |
| **532** | **Nct (2018)** | **Comparing Function, Pain and Return to Work in Conservative Versus Surgical Treated Stable Lateral Malleolar Fractures** | **[https://clinicaltrials.gov/show/NCT03587571](https://clinicaltrials.gov/show/NCT03587571)** | **Excluded** | **Review articles, conference papers** |
| **533** | **Nct (2018)** | **A Comparison Of Block Quality In Anterior And Posterior Approach To Sciatic Nerve Block** | **[https://clinicaltrials.gov/show/NCT03607292](https://clinicaltrials.gov/show/NCT03607292)** | **Excluded** | **Review articles, conference papers** |
| **534** | **Nct (2018)** | **Could Music be an Alternative to Sedation in Patients Treated Total Knee Arthroplasty With Regional Anesthesia** | **[https://clinicaltrials.gov/show/NCT03882541](https://clinicaltrials.gov/show/NCT03882541)** | **Excluded** | **Review articles, conference papers** |
| **535** | **Nct (2018)** | **Determine the Most Effective Intervention for Hemorrhage Control Readiness for Laypersons: the PATTS Trial** | **[https://clinicaltrials.gov/show/NCT03479112](https://clinicaltrials.gov/show/NCT03479112)** | **Excluded** | **Review articles, conference papers** |
| **536** | **Nct (2018)** | **Does the Preoperative Midazolam Dose Affect Postoperative Pain** | **[https://clinicaltrials.gov/show/NCT03534895](https://clinicaltrials.gov/show/NCT03534895)** | **Excluded** | **Review articles, conference papers** |
| **537** | **Nct (2018)** | **Educational Program for Knee Osteoarthritis** | **[https://clinicaltrials.gov/show/NCT03588195](https://clinicaltrials.gov/show/NCT03588195)** | **Excluded** | **Review articles, conference papers** |
| **538** | **Nct (2018)** | **Effect of Designated Education Session on Patellofemoral Pain** | **[https://clinicaltrials.gov/show/NCT03784339](https://clinicaltrials.gov/show/NCT03784339)** | **Excluded** | **Review articles, conference papers** |
| **539** | **Nct (2018)** | **Effectiveness of Postural Insoles Adapted in Slippers for People With Persistent Heel Pain** | **[https://clinicaltrials.gov/show/NCT03482518](https://clinicaltrials.gov/show/NCT03482518)** | **Excluded** | **Review articles, conference papers** |
| **540** | **Nct (2018)** | **Effects of Catheter Location Relative to Femoral Artery on Postoperative Analgesia for Continuous Adductor Canal Blocks** | **[https://clinicaltrials.gov/show/NCT03650504](https://clinicaltrials.gov/show/NCT03650504)** | **Excluded** | **Review articles, conference papers** |
| **541** | **Nct (2018)** | **Effects of Osteopathic Manipulative Treatment (OMT) on Gait Biomechanics in Parkinson's Disease** | **[https://clinicaltrials.gov/show/NCT03616145](https://clinicaltrials.gov/show/NCT03616145)** | **Excluded** | **Review articles, conference papers** |
| **542** | **Nct (2018)** | **The Efficacy of TGF for Treating Osteoarthritis of the Knee** | **[https://clinicaltrials.gov/show/NCT03562429](https://clinicaltrials.gov/show/NCT03562429)** | **Excluded** | **Review articles, conference papers** |
| **543** | **Nct (2018)** | **Internal Fixation or Joint Replacement Therapy for Aged Hip Fracture Patients** | **[https://clinicaltrials.gov/show/NCT03407131](https://clinicaltrials.gov/show/NCT03407131)** | **Excluded** | **Review articles, conference papers** |
| **544** | **Nct (2018)** | **Intravenous Lidocaine in Total Knee Replacement** | **[https://clinicaltrials.gov/ct2/show/NCT03597776](https://clinicaltrials.gov/ct2/show/NCT03597776)** | **Excluded** | **Review articles, conference papers** |
| **545** | **Nct (2018)** | **Lower Extremity Alignment and Dynamic Control With Associated Injury Risk in College Athletes With Knee Hyperextension** | **[https://clinicaltrials.gov/show/NCT03425968](https://clinicaltrials.gov/show/NCT03425968)** | **Excluded** | **Review articles, conference papers** |
| **546** | **Nct (2018)** | **Lymphodreinage Integrated With Kinesio Tape in TKA Patients** | **[https://clinicaltrials.gov/show/NCT03452995](https://clinicaltrials.gov/show/NCT03452995)** | **Excluded** | **Review articles, conference papers** |
| **547** | **Nct (2018)** | **Prevention of Delirium Among Elderly Patients With Hip Fractures** | **[https://clinicaltrials.gov/show/NCT03470662](https://clinicaltrials.gov/show/NCT03470662)** | **Excluded** | **Review articles, conference papers** |
| **548** | **Nct (2018)** | **Role of Patient-controlled Epidural Analgesia After Total Hip Replacement** | **[https://clinicaltrials.gov/show/NCT03599024](https://clinicaltrials.gov/show/NCT03599024)** | **Excluded** | **Review articles, conference papers** |
| **549** | **Nct (2018)** | **Safety and Efficacy of Pre-emptive Tapentadol vs Pregabalin in Post Operative Pain Following Surgery** | **[https://clinicaltrials.gov/show/NCT03604354](https://clinicaltrials.gov/show/NCT03604354)** | **Excluded** | **Review articles, conference papers** |
| **550** | **Nct (2018)** | **STaR Trial: Multiple Ligament Knee Injuries** | **[https://clinicaltrials.gov/ct2/show/NCT03543098](https://clinicaltrials.gov/ct2/show/NCT03543098)** | **Excluded** | **Review articles, conference papers** |
| **551** | **Nct (2018)** | **Surgical Techniques in Arthroplasty of the Knee (STArK) 1 Trial** | **[https://clinicaltrials.gov/show/NCT03505645](https://clinicaltrials.gov/show/NCT03505645)** | **Excluded** | **Review articles, conference papers** |
| **552** | **Nct (2018)** | **Treatment of Osteoarthritis With Autologous, Microfragmented Adipose Tissue** | **[https://clinicaltrials.gov/show/NCT03771989](https://clinicaltrials.gov/show/NCT03771989)** | **Excluded** | **Review articles, conference papers** |
| **553** | **Nct (2018)** | **Ultrasound-Guided Percutaneous Peripheral Nerve Stimulation: a Department of Defense Funded Multicenter Pilot Study** | **[https://clinicaltrials.gov/show/NCT03481725](https://clinicaltrials.gov/show/NCT03481725)** | **Excluded** | **Review articles, conference papers** |
| **554** | **Nct (2018)** | **Anesthetic Methods and Gene Expression Profile** | **[https://clinicaltrials.gov/show/NCT03585647](https://clinicaltrials.gov/show/NCT03585647)** | **Excluded** | **Review articles, conference papers** |
| **555** | **Nct (2018)** | **Comparing Through-the-Needle With Suture-Method Catheter Designs for Popliteal Nerve Blocks** | **[https://clinicaltrials.gov/show/NCT03442036](https://clinicaltrials.gov/show/NCT03442036)** | **Excluded** | **Review articles, conference papers** |
| **556** | **Nct (2018)** | **Comparison of Biofeedback Tools to Train the Transversus Abdominis Activation in Healthy Subjects** | **[https://clinicaltrials.gov/show/NCT03543501](https://clinicaltrials.gov/show/NCT03543501)** | **Excluded** | **Review articles, conference papers** |
| **557** | **Nct (2018)** | **Complete Decongestive Therapy With Negative Pressure for Lipedema and Lymphedema Therapy** | **[https://clinicaltrials.gov/show/NCT03634462](https://clinicaltrials.gov/show/NCT03634462)** | **Excluded** | **Review articles, conference papers** |
| **558** | **Nct (2018)** | **Development of Structured Exercise Program for T2DM Management** | **[https://clinicaltrials.gov/show/NCT03563456](https://clinicaltrials.gov/show/NCT03563456)** | **Excluded** | **Review articles, conference papers** |
| **559** | **Nct (2018)** | **Effect of Apple Cider Vinegar in Type 2 Diabetics** | **[https://clinicaltrials.gov/show/NCT03593135](https://clinicaltrials.gov/show/NCT03593135)** | **Excluded** | **Review articles, conference papers** |
| **560** | **Nct (2018)** | **The Effect of Fasting on ICSI Outcomes in Patients With Polycystic Ovary Syndrome** | **[https://clinicaltrials.gov/show/NCT03703115](https://clinicaltrials.gov/show/NCT03703115)** | **Excluded** | **Review articles, conference papers** |
| **561** | **Nct (2018)** | **Effects of Mediterranean Diet Intervention in Diabetic Heart Disease** | **[https://clinicaltrials.gov/show/NCT03757845](https://clinicaltrials.gov/show/NCT03757845)** | **Excluded** | **Review articles, conference papers** |
| **562** | **Nct (2018)** | **Effects of Pelvic Patterns of Proprioceptive Neuromuscular Facilitation in the Pelvic Floor Muscles** | **[https://clinicaltrials.gov/show/NCT03484169](https://clinicaltrials.gov/show/NCT03484169)** | **Excluded** | **Review articles, conference papers** |
| **563** | **Nct (2018)** | **Effects of Photobiomodulation and Deep Water Running Training in Subjects With Low Back Pain** | **[https://clinicaltrials.gov/show/NCT03465228](https://clinicaltrials.gov/show/NCT03465228)** | **Excluded** | **Review articles, conference papers** |
| **564** | **Nct (2018)** | **Evaluation of Gait Symmetry in Upper Extremity Burn Injuries** | **[https://clinicaltrials.gov/show/NCT03759613](https://clinicaltrials.gov/show/NCT03759613)** | **Excluded** | **Review articles, conference papers** |
| **565** | **Nct (2018)** | **Feasibility & Effect of a Tele-rehabilitation Program for Chronic Obstructive Pulmonary Disease vs. Standard Rehabilitation** | **[https://clinicaltrials.gov/show/NCT03569384](https://clinicaltrials.gov/show/NCT03569384)** | **Excluded** | **Review articles, conference papers** |
| **566** | **Nct (2018)** | **Feasibility & Effect of a Tele-rehabilitation Program in Idiopathic Pulmonary Fibrosis (IPF)** | **[https://clinicaltrials.gov/show/NCT03548181](https://clinicaltrials.gov/show/NCT03548181)** | **Excluded** | **Review articles, conference papers** |
| **567** | **Nct (2018)** | **The Feasibility and Effects of Low-load Blood-flow Restricted Exercise Following Spinal Cord Injury** | **[https://clinicaltrials.gov/show/NCT03690700](https://clinicaltrials.gov/show/NCT03690700)** | **Excluded** | **Review articles, conference papers** |
| **568** | **Nct (2018)** | **A High-Intensity Exercise Program in Post-Bariatric Patients** | **[https://clinicaltrials.gov/show/NCT03603392](https://clinicaltrials.gov/show/NCT03603392)** | **Excluded** | **Review articles, conference papers** |
| **569** | **Nct (2018)** | **Hypotension Probability Index in Anesthesia** | **[https://clinicaltrials.gov/show/NCT03663270](https://clinicaltrials.gov/show/NCT03663270)** | **Excluded** | **Review articles, conference papers** |
| **570** | **Nct (2018)** | **Ketogenic Diet and CPAP Previous Bariatric Surgery** | **[https://clinicaltrials.gov/show/NCT03791242](https://clinicaltrials.gov/show/NCT03791242)** | **Excluded** | **Review articles, conference papers** |
| **571** | **Nct (2018)** | **Looking for Personalized Nutrition for Obesity/Type 2 Diabetes Mellitus Prevention** | **[https://clinicaltrials.gov/show/NCT03792685](https://clinicaltrials.gov/show/NCT03792685)** | **Excluded** | **Review articles, conference papers** |
| **572** | **Nct (2018)** | **Low-Dose Intravenous Ketamine Bolus Versus Conventional Technique** | **[https://clinicaltrials.gov/show/NCT03499886](https://clinicaltrials.gov/show/NCT03499886)** | **Excluded** | **Review articles, conference papers** |
| **573** | **Nct (2018)** | **Lymphodreinage Integrated With Kinesio Tape in TKA Patients** | **[https://clinicaltrials.gov/show/NCT03452995](https://clinicaltrials.gov/show/NCT03452995)** | **Excluded** | **Review articles, conference papers** |
| **574** | **Nct (2018)** | **A Mechanistic Randomized Controlled Trial on the Cardiovascular Effect of Berberine** | **[https://clinicaltrials.gov/show/NCT03770325](https://clinicaltrials.gov/show/NCT03770325)** | **Excluded** | **Review articles, conference papers** |
| **575** | **Nct (2018)** | **Miniscrews Primary Stability** | **[https://clinicaltrials.gov/show/NCT03696511](https://clinicaltrials.gov/show/NCT03696511)** | **Excluded** | **Review articles, conference papers** |
| **576** | **Nct (2018)** | **MPA Versus Dydrogesterone for Management of Endometrial Hyperplasia Without Atypia** | **[https://clinicaltrials.gov/show/NCT03675139](https://clinicaltrials.gov/show/NCT03675139)** | **Excluded** | **Review articles, conference papers** |
| **577** | **Nct (2018)** | **Multi-Sensory Training and Wrist Fractures** | **[https://clinicaltrials.gov/show/NCT03478228](https://clinicaltrials.gov/show/NCT03478228)** | **Excluded** | **Review articles, conference papers** |
| **578** | **Nct (2018)** | **Pain Reduction in Tibial Stress Syndrome** | **[https://clinicaltrials.gov/show/NCT03676530](https://clinicaltrials.gov/show/NCT03676530)** | **Excluded** | **Review articles, conference papers** |
| **579** | **Nct (2018)** | **Physiotherapeutic Interventions Applied to the Bladder Pain Syndrome** | **[https://clinicaltrials.gov/show/NCT03755375](https://clinicaltrials.gov/show/NCT03755375)** | **Excluded** | **Review articles, conference papers** |
| **580** | **Nct (2018)** | **Praziquantel in Children Under Age 4** | **[https://clinicaltrials.gov/show/NCT03640377](https://clinicaltrials.gov/show/NCT03640377)** | **Excluded** | **Review articles, conference papers** |
| **581** | **Nct (2018)** | **Preoperative High Dose Steroids for Liver Resection- Effect on Complications in the Immediate Postoperative Period** | **[https://clinicaltrials.gov/show/NCT03403517](https://clinicaltrials.gov/show/NCT03403517)** | **Excluded** | **Review articles, conference papers** |
| **582** | **Nct (2018)** | **Shoulder Brace on Muscle Activation and Scapular Kinematics in Patients With Shoulder Impingement Syndrome and Rounded Shoulder Posture** | **[https://clinicaltrials.gov/show/NCT03667833](https://clinicaltrials.gov/show** | **Excluded** | **Review articles, conference papers** |
| **583** | **Nct (2018)** | **Sleep and Circadian Intervention Program for Chronic Musculoskeletal Pain** | **[https://clinicaltrials.gov/show/NCT03646084](https://clinicaltrials.gov/show/NCT03646084)** | **Excluded** | **Review articles, conference papers** |
| **584** | **Nct (2018)** | **Supervised Physical Therapy Versus Unsupervised Home Exercise After Surgery for Lumbar Disc Herniation** | **[https://clinicaltrials.gov/show/NCT03505918](https://clinicaltrials.gov/show/NCT03505918)** | **Excluded** | **Review articles, conference papers** |
| **585** | **Nct (2018)** | **Tourniquet Training Effectiveness Study** | **[https://clinicaltrials.gov/show/NCT03538379](https://clinicaltrials.gov/show/NCT03538379)** | **Excluded** | **Review articles, conference papers** |
| **586** | **Nct (2018)** | **Two Mathematical Methods to Estimate Arterial Occlusion Pressure and Tourniquet Effectiveness in Lower Limb Surgery** | **[https://clinicaltrials.gov/show/NCT03706859](https://clinicaltrials.gov/show/NCT03706859)** | **Excluded** | **Review articles, conference papers** |
| **587** | **Nct (2018)** | **Whole-body Electromyostimulation in Inpatient Rehabilitation** | **[https://clinicaltrials.gov/show/NCT03767088](https://clinicaltrials.gov/show/NCT03767088)** | **Excluded** | **Review articles, conference papers** |
| **588** | **Nct et al** | **Awareness Development and Usage of mHealth Technology Among Hypertensive Patients in a Rural Community of Bangladesh** | **[https://clinicaltrials.gov/show/NCT03614104](https://clinicaltrials.gov/show/NCT03614104)** | **Excluded** | **Review articles, conference papers** |
| **589** | **Nct et al** | **Comparison of the Efficiency of Two Different PPI Formula in Atypical GERD** | **[https://clinicaltrials.gov/show/NCT03418337](https://clinicaltrials.gov/show/NCT03418337)** | **Excluded** | **Review articles, conference papers** |
| **590** | **Nct et al** | **Effect of Chromium Supplementation on Intracytoplasmatic Sperm Injection (ICSI) Outcomes in Polycystic Ovary Syndrome Ladies** | **[https://clinicaltrials.gov/show/NCT03503201](https://clinicaltrials.gov/show/NCT03503201)** | **Excluded** | **Review articles, conference papers** |
| **591** | **Nct et al** | **EfiKroniK Research Program: physical Exercise for People With Chronic Pathologies** | **[https://clinicaltrials.gov/show/NCT03810755](https://clinicaltrials.gov/show/NCT03810755)** | **Excluded** | **Review articles, conference papers** |
| **592** | **Nct et al** | **Evaluation of Second Esmarch Application on Intravenous Regional Anesthesia Effectiveness** | **[https://clinicaltrials.gov/show/NCT03702387](https://clinicaltrials.gov/show/NCT03702387)** | **Excluded** | **Review articles, conference papers** |
| **593** | **Nct et al** | **Exercise and Weight Control** | **[https://clinicaltrials.gov/show/NCT03413826](https://clinicaltrials.gov/show/NCT03413826)** | **Excluded** | **Review articles, conference papers** |
| **594** | **Nct et al** | **Ketogenic Diet: a Novel Metabolic Strategy to Treat Lymphedema Patients?** | **[https://clinicaltrials.gov/ct2/show/NCT03991897](https://clinicaltrials.gov/ct2/show/NCT03991897)** | **Excluded** | **Review articles, conference papers** |
| **595** | **Nct et al** | **Light Intensity Physical Activity Trial** | **[https://clinicaltrials.gov/show/NCT03415880](https://clinicaltrials.gov/show/NCT03415880)** | **Excluded** | **Review articles, conference papers** |
| **596** | **Nct et al** | **Prismatic Lenses in the Pisa Syndrome** | **[https://clinicaltrials.gov/show/NCT03737773](https://clinicaltrials.gov/show/NCT03737773)** | **Excluded** | **Review articles, conference papers** |
| **597** | **Nct et al** | **Randomized Control Trial of Booster Seat Education Material to Increase Perceived Benefit Among Parents** | **[https://clinicaltrials.gov/show/NCT03573830](https://clinicaltrials.gov/show/NCT03573830)** | **Excluded** | **Review articles, conference papers** |
| **598** | **Nct et al** | **Restorelle® Y Mesh vs. Vertessa® Lite Y Mesh for Laparoscopic and Robotic-assisted Laparoscopic Sacrocolpopexy** | **[https://clinicaltrials.gov/ct2/show/NCT03681223](https://clinicaltrials.gov/ct2/show/NCT03681223)** | **Excluded** | **Review articles, conference papers** |
| **599** | **Nct et al** | **Clopidogrel Aspirin Therapy (CAT) Versus Apixaban Aspirin Therapy (AAT) After Lower Limb Revascularization** | **[https://clinicaltrials.gov/show/NCT04168398](https://clinicaltrials.gov/show/NCT04168398)** | **Excluded** | **Review articles, conference papers** |
| **600** | **Nct et al** | **A Comparative Study of Stem and Cup Fixation and Polyethylene Wear** | **[https://clinicaltrials.gov/ct2/show/NCT04064723](https://clinicaltrials.gov/ct2/show/NCT04064723)** | **Excluded** | **Review articles, conference papers** |
| **601** | **Nct et al** | **Comparing Fascia Iliaca Compartment Block and Pericapsular Nerve Group Block for Hip Fracture Pain Control** | **[https://clinicaltrials.gov/ct2/show/NCT04210700](https://clinicaltrials.gov/ct2/show/NCT04210700)** | **Excluded** | **Review articles, conference papers** |
| **602** | **Nct et al** | **The CYBERnetic LowEr Imb coGnitive Ortho-prostheis Plus Plus, 2nd Clinical Study (CLs++)** | **[https://clinicaltrials.gov/show/NCT04129853](https://clinicaltrials.gov/show/NCT04129853)** | **Excluded** | **Review articles, conference papers** |
| **603** | **Nct et al** | **Determination of the Effectiveness of Certain Physical Methods in the Treatment of Knee Osteoarthritis** | **[https://clinicaltrials.gov/show/NCT04197284](https://clinicaltrials.gov/show/NCT04197284)** | **Excluded** | **Review articles, conference papers** |
| **604** | **Nct et al** | **Effect of Preoperative Mobility Device Training on Postoperative Fall Incidence** | **[https://clinicaltrials.gov/ct2/show/NCT03857945](https://clinicaltrials.gov/ct2/show/NCT03857945)** | **Excluded** | **Review articles, conference papers** |
| **605** | **Nct et al** | **Effect of Short-term Motor Training on Accuracy and Precision of Knee Movement in Human With and Without Knee Pain** | **[https://clinicaltrials.gov/show/NCT04146311](https://clinicaltrials.gov/show/NCT04146311)** | **Excluded** | **Review articles, conference papers** |
| **606** | **Nct et al** | **Effects of Connective Tissue Massage and Classical Massage** | **[https://clinicaltrials.gov/show/NCT04211701](https://clinicaltrials.gov/show/NCT04211701)** | **Excluded** | **Review articles, conference papers** |
| **607** | **Nct et al** | **Effects of Gait Rehabilitation With Dual Task in Patients With Parkinson's Disease** | **[https://clinicaltrials.gov/show/NCT04038866](https://clinicaltrials.gov/show/NCT04038866)** | **Excluded** | **Review articles, conference papers** |
| **608** | **Nct et al** | **The Effects of Proprioceptive Neuromuscular Facilitation and Static Stretching Exercises** | **[https://clinicaltrials.gov/show/NCT04026646](https://clinicaltrials.gov/show/NCT04026646)** | **Excluded** | **Review articles, conference papers** |
| **609** | **Nct et al** | **Immediate Effects of Proximal and Distal Acupoints on the RPPW in Patients With KOA: a Randomized Controlled Trial** | **[https://clinicaltrials.gov/show/NCT03925467](https://clinicaltrials.gov/show/NCT03925467)** | **Excluded** | **Review articles, conference papers** |
| **610** | **Nct et al** | **Investigating the Effectiveness of Vibration Therapy on Sarcopenia in Osteoarthritis Knee Patients** | **[https://clinicaltrials.gov/show/NCT03880344](https://clinicaltrials.gov/show/NCT03880344)** | **Excluded** | **Review articles, conference papers** |
| **611** | **Nct et al** | **Kentucky Communities and Researchers Engaging to Halt the Opioid Epidemic (CARE2HOPE)** | **[https://clinicaltrials.gov/ct2/show/NCT04134767](https://clinicaltrials.gov/ct2/show/NCT04134767)** | **Excluded** | **Review articles, conference papers** |
| **612** | **Nct et al** | **Knee Osteoarthritis: platelet Rich Plasma or Hyaluronic Acid** | **[https://clinicaltrials.gov/show/NCT03801564](https://clinicaltrials.gov/show/NCT03801564)** | **Excluded** | **Review articles, conference papers** |
| **613** | **Nct et al** | **Open Versus Arthroscopic Release for Lateral Patellar Compression Syndrome: comparative Study** | **[https://clinicaltrials.gov/show/NCT04130412](https://clinicaltrials.gov/show/NCT04130412)** | **Excluded** | **Review articles, conference papers** |
| **614** | **Nct et al** | **Opioids Versus Non-Opioids Postoperative After Knee Arthroscopic Surgery** | **[https://clinicaltrials.gov/ct2/show/NCT03858231](https://clinicaltrials.gov/ct2/show/NCT03858231)** | **Excluded** | **Review articles, conference papers** |
| **615** | **Nct et al** | **Postoperative Drainage in Total Knee Arthroplasty in the Presence of Tranexamic Acid** | **[https://clinicaltrials.gov/show/NCT03915756](https://clinicaltrials.gov/show/NCT03915756)** | **Excluded** | **Review articles, conference papers** |
| **616** | **Nct et al** | **Strontium Ranelate and KOA** | **[https://clinicaltrials.gov/show/NCT03937518](https://clinicaltrials.gov/show** | **Excluded** | **Review articles, conference papers** |
| **617** | **Nct et al** | **Total Knee Arthroplasty With and Without Tourniquet: comparative Study** | **[https://clinicaltrials.gov/show/NCT04130009](https://clinicaltrials.gov/show/NCT04130009)** | **Excluded** | **Review articles, conference papers** |
| **618** | **Nct et al** | **Ultrasound Evaluation of Lower Extremity Arteries After Topical Nitroglycerin Administration** | **[https://clinicaltrials.gov/show/NCT04155476](https://clinicaltrials.gov/show/NCT04155476)** | **Excluded** | **Review articles, conference papers** |
| **619** | **Nct et al** | **What is the Feasibility of the ISTEP Exercise Test in Boys With Haemophilia** | **[https://clinicaltrials.gov/show/NCT04076306](https://clinicaltrials.gov/show/NCT04076306)** | **Excluded** | **Review articles, conference papers** |
| **620** | **Nct et al** | **The Whole Body Vibration Training for Total Knee arthroplasty-the Improvement of the Lower Limb** | **[https://clinicaltrials.gov/show/NCT04107350](https://clinicaltrials.gov/show/NCT04107350)** | **Excluded** | **Review articles, conference papers** |
| **621** | **Nct et al** | **Assessing if Cryoneurolysis Improves Prehabilitation and Decreases Pain After Surgery With Less Opioid Use in TKA Patients** | **[https://clinicaltrials.gov/show/NCT03836313](https://clinicaltrials.gov/show/NCT03836313)** | **Excluded** | **Review articles, conference papers** |
| **622** | **Nct et al** | **Bio Impedance-assisted Monitoring of Chronic Hemodialysis Patients** | **[https://clinicaltrials.gov/show/NCT04127877](https://clinicaltrials.gov/show/NCT04127877)** | **Excluded** | **Review articles, conference papers** |
| **623** | **Nct et al** | **Caudal Epidural Steroid Injection Ultrasound Guided in LDP** | **[https://clinicaltrials.gov/show/NCT03933150](https://clinicaltrials.gov/show/NCT03933150)** | **Excluded** | **Review articles, conference papers** |
| **624** | **Nct et al** | **Collaborative Model of Care Between Orthopaedics and Allied Healthcare Professionals Trial (CONnACT)** | **[https://clinicaltrials.gov/show/NCT03809975](https://clinicaltrials.gov/show/NCT03809975)** | **Excluded** | **Review articles, conference papers** |
| **625** | **Nct et al** | **Comparison of Complete Decongestive Therapy With Intermittent Pneumatic Compression for Treatment of Lipedema** | **[https://clinicaltrials.gov/show/NCT03924999](https://clinicaltrials.gov/show/NCT03924999)** | **Excluded** | **Review articles, conference papers** |
| **626** | **Nct et al** | **Creatine Supplementation During Resistance Training for People Recovering From Stroke** | **[https://clinicaltrials.gov/show/NCT03941678](https://clinicaltrials.gov/show/NCT03941678)** | **Excluded** | **Review articles, conference papers** |
| **627** | **Nct et al** | **Detection and Prevention of Concussive Injuries With Smart Technology** | **[https://clinicaltrials.gov/show/NCT04946747](https://clinicaltrials.gov/show/NCT04946747)** | **Excluded** | **Review articles, conference papers** |
| **628** | **Nct et al** | **The Effect of a Prehabilitation Exercise Program on Physical Functioning for Patients Undergoing Kidney Transplantation** | **[https://clinicaltrials.gov/ct2/show/NCT04044963](https://clinicaltrials.gov/ct2/show/NCT04044963)** | **Excluded** | **Review articles, conference papers** |
| **629** | **Nct et al** | **Effect of Intravenous Dexmedetomidine on Patient Discomfort in Laparoscopic Cholecystectomy Under Spinal Anesthesia** | **[https://clinicaltrials.gov/show/NCT04115449](https://clinicaltrials.gov/show/NCT04115449)** | **Excluded** | **Review articles, conference papers** |
| **630** | **Nct et al** | **Effect of TXA on Blood Loss in Reverse Total Shoulder Arthroplasty** | **[https://clinicaltrials.gov/ct2/show/NCT06638749](https://clinicaltrials.gov/ct2/show/NCT06638749)** | **Excluded** | **Review articles, conference papers** |
| **631** | **Nct et al** | **Effectiveness of Curcumin-based Food Supplement in Reducing Pain and Inflammatory Component in Osteoarthritis** | **[https://clinicaltrials.gov/show/NCT04207021](https://clinicaltrials.gov/show/NCT04207021)** | **Excluded** | **Review articles, conference papers** |
| **632** | **Nct et al** | **Effectiveness of Quick Icing (QI) Technique on Strenght of Jump** | **[https://clinicaltrials.gov/show/NCT04008602](https://clinicaltrials.gov/show/NCT04008602)** | **Excluded** | **Review articles, conference papers** |
| **633** | **Nct et al** | **Effects of Bilateral Asymmetrical Limbs Proprioceptive Neuromuscular Facilitation on Multifidus Muscle in CLBP** | **[https://clinicaltrials.gov/show/NCT04206137](https://clinicaltrials.gov/show/NCT04206137)** | **Excluded** | **Review articles, conference papers** |
| **634** | **Nct et al** | **Effects of Sulfur Water and Mud Therapy on Serotonin Activity and Biochemical Parameters in Patients' Osteoarthrosis** | **[https://clinicaltrials.gov/show/NCT04192162](https://clinicaltrials.gov/show/NCT04192162)** | **Excluded** | **Review articles, conference papers** |
| **635** | **Nct et al** | **Electroacupuncture Frequency-related Effects on Non-specific Low Back Pain in Older Adults** | **[https://clinicaltrials.gov/show/NCT03802045](https://clinicaltrials.gov/show/NCT03802045)** | **Excluded** | **Review articles, conference papers** |
| **636** | **Nct et al** | **Erector Spinae Plane Block for Postoperative Pain Control in Hip Replacement Surgeries** | **[https://clinicaltrials.gov/show/NCT04003909](https://clinicaltrials.gov/show/NCT04003909)** | **Excluded** | **Review articles, conference papers** |
| **637** | **Nct et al** | **High-Intensity Interval Training Recuperates Capacity of Endogenous Thrombin Generation in Patients With Heart Failure** | **[https://clinicaltrials.gov/show/NCT04033523](https://clinicaltrials.gov/show/NCT04033523)** | **Excluded** | **Review articles, conference papers** |
| **638** | **Nct et al** | **Intra-articular Administration of 3.0g Tranexamic Acid Has no Effect on Reducing Intra-articular Hemarthrosis and Postoperative Pain After Primary Anterior Cruciate Ligament Reconstruction: a Randomized Controlled Trial** | **[https://clinicaltrials.gov/show/NCT04042688](https://clinicaltrials.gov/show/NCT04042688)** | **Excluded** | **Review articles, conference papers** |
| **639** | **Nct et al** | **Living Green and Healthy for Teens** | **[https://clinicaltrials.gov/ct2/show/NCT03996109](https://clinicaltrials.gov/ct2/show/NCT03996109)** | **Excluded** | **Review articles, conference papers** |
| **640** | **Nct et al** | **Lumbar Brace Deployment in the Emergency Department for Benign Low Back Pain** | **[https://clinicaltrials.gov/ct2/show/NCT03829631](https://clinicaltrials.gov/ct2/show/NCT03829631)** | **Excluded** | **Review articles, conference papers** |
| **641** | **Nct et al** | **Lumbar Bracing for People With Type I Modic Changes** | **[https://clinicaltrials.gov/ct2/show/NCT03829644](https://clinicaltrials.gov/ct2/show/NCT03829644)** | **Excluded** | **Review articles, conference papers** |
| **642** | **Nct et al** | **Motor Control Training in Individuals With Subacromial Pain Syndrome** | **[https://clinicaltrials.gov/show/NCT04104906](https://clinicaltrials.gov/show/NCT04104906)** | **Excluded** | **Review articles, conference papers** |
| **643** | **Nct et al** | **Novel Tools for the Delivery and Assessment of Exercise Programs Adapted to Individuals With Parkinson's Disease** | **[https://clinicaltrials.gov/show/NCT04078217](https://clinicaltrials.gov/show/NCT04078217)** | **Excluded** | **Review articles, conference papers** |
| **644** | **Nct et al** | **Objective Assessment for Caregivers With Work-related Musculoskeletal Disorders of the Shoulder** | **[https://clinicaltrials.gov/show/NCT03886545](https://clinicaltrials.gov/show/NCT03886545)** | **Excluded** | **Review articles, conference papers** |
| **645** | **Nct et al** | **Piloting Diet and Exercise Interventions in Older Hispanics With Diabetes** | **[https://clinicaltrials.gov/show/NCT04132739](https://clinicaltrials.gov/show/NCT04132739)** | **Excluded** | **Review articles, conference papers** |
| **646** | **Nct et al** | **Post-needling Soreness After Internal Gastrocnemius Muscle Treatment** | **[https://clinicaltrials.gov/show/NCT04060576](https://clinicaltrials.gov/show/NCT04060576)** | **Excluded** | **Review articles, conference papers** |
| **647** | **Nct et al** | **Preference, Exercise Therapy Adherence and Efficacy Low Back Pain** | **[https://clinicaltrials.gov/show/NCT03984864](https://clinicaltrials.gov/show/NCT03984864)** | **Excluded** | **Review articles, conference papers** |
| **648** | **Nct et al** | **PRP IN Planter Fascitis** | **[https://clinicaltrials.gov/show/NCT03938896](https://clinicaltrials.gov/show/NCT03938896)** | **Excluded** | **Review articles, conference papers** |
| **649** | **Nct et al** | **Relative Desirability of Metformin vs. Birth Control Pill in Treating PCOS in Women of Later Reproductive Age** | **[https://clinicaltrials.gov/show/NCT03905941](https://clinicaltrials.gov/show/NCT03** | **Excluded** | **Review articles, conference papers** |
| **650** | **Nct et al** | **STABILITY 2: anterior Cruciate Ligament Reconstruction +/- Lateral Tenodesis With Patellar vs Quad Tendon** | **[https://clinicaltrials.gov/ct2/show/NCT03935750](https://clinicaltrials.gov/ct2/show/NCT03935750)** | **Excluded** | **Review articles, conference papers** |
| **651** | **Nct et al** | **Targeting Physical Health in Schizophrenia: physical Activity Can Enhance Life Randomized Control Trial** | **[https://clinicaltrials.gov/show/NCT04173572](https://clinicaltrials.gov/show/NCT04173572)** | **Excluded** | **Review articles, conference papers** |
| **652** | **Nct et al** | **Tendon-Bone Versus All-Soft-Tissue for ACL Reconstruction: a Patient-Blinded Randomized Clinical Trial** | **[https://clinicaltrials.gov/ct2/show/NCT04039971](https://clinicaltrials.gov/ct2/show/NCT04039971)** | **Excluded** | **Review articles, conference papers** |
| **653** | **Nct et al** | **Use of Hyaluronic Acid Injection in Lateral Patellar Compression With Femoral Condylar Degenerative Changes After Arthroscopic Release** | **[https://clinicaltrials.gov/show/NCT04134611](https://clinicaltrials.gov/show/NCT04134611)** | **Excluded** | **Review articles, conference papers** |
| **654** | **Nct (2019)** | **3MDR to Treat PTSD With mTBI (3MDR)** | **[https://clinicaltrials.gov/show/NCT03796936](https://clinicaltrials.gov/show/NCT03796936)** | **Excluded** | **Review articles, conference papers** |
| **655** | **Nct (2019)** | **Alendronate Versus Denosumab in Kidney Transplant Patients** | **[https://clinicaltrials.gov/show/NCT04169698](https://clinicaltrials.gov/show/NCT04169698)** | **Excluded** | **Review articles, conference papers** |
| **656** | **Nct (2019)** | **ARAPS Study on Accelerated Liver Regeneration** | **[https://clinicaltrials.gov/show/NCT04107324](https://clinicaltrials.gov/show/NCT04107324)** | **Excluded** | **Review articles, conference papers** |
| **657** | **Nct (2019)** | **Brain Insulin Resistance in Mood Disorders** | **[https://clinicaltrials.gov/ct2/show/NCT03915613](https://clinicaltrials.gov/ct2/show/NCT03915613)** | **Excluded** | **Review articles, conference papers** |
| **658** | **Nct (2019)** | **Clinical Efficacy of Telmisartan in Reducing Cardiac Remodeling Among Obese Patients With Hypertension** | **[https://clinicaltrials.gov/show/NCT03956823](https://clinicaltrials.gov/show/NCT03956823)** | **Excluded** | **Review articles, conference papers** |
| **659** | **Nct (2019)** | **Community-based Screening of Chronic Kidney Disease (CKD) and Measure the Impact of Health Education** | **[https://clinicaltrials.gov/show/NCT04094831](https://clinicaltrials.gov/show/NCT04094831)** | **Excluded** | **Review articles, conference papers** |
| **660** | **Nct (2019)** | **Comparative Study of Ultrasound Guided Combined Interscalene and Supraclavicular Brachial Plexus Block Versus General Anesthesia for Brachioaxillary Surgery in Renal Failure Patient** | **[https://clinicaltrials.gov/show/NCT04050891](https://clinicaltrials.gov/show/NCT04050891)** | **Excluded** | **Review articles, conference papers** |
| **661** | **Nct (2019)** | **Comparing Injection Treatments for Tennis Elbow** | **[https://clinicaltrials.gov/ct2/show/NCT03984955](https://clinicaltrials.gov/ct2/show/NCT03984955)** | **Excluded** | **Review articles, conference papers** |
| **662** | **Nct (2019)** | **Effect of Dry Roasted Peanuts and Boiled Peanuts on Glycemic Control** | **[https://clinicaltrials.gov/show/NCT04171648](https://clinicaltrials.gov/show/NCT04171648)** | **Excluded** | **Review articles, conference papers** |
| **663** | **Nct (2019)** | **Effect of Self- and Family Management of Diabetic Foot Ulcers Programs on Health Outcomes** | **[https://clinicaltrials.gov/show/NCT03909802](https://clinicaltrials.gov/show/NCT03909802)** | **Excluded** | **Review articles, conference papers** |
| **664** | **Nct (2019)** | **Effects of the Nintendo Wii Fit Game Training on Balance** | **[https://clinicaltrials.gov/show/NCT03983642](https://clinicaltrials.gov/show/NCT03983642)** | **Excluded** | **Review articles, conference papers** |
| **665** | **Nct (2019)** | **Efficacy and Safety of Therapy With IgM-enriched Immunoglobulin With a Personalized Dose vs Standard Dose in Patients With Septic Shock** | **[https://clinicaltrials.gov/show/NCT04182737](https://clinicaltrials.gov/show/NCT04182737)** | **Excluded** | **Review articles, conference papers** |
| **666** | **Nct (2019)** | **Evaluation of the Move 2 Learn (M2L) Program for Young Children** | **[https://clinicaltrials.gov/show/NCT03999619](https://clinicaltrials.gov/show/NCT03999619)** | **Excluded** | **Review articles, conference papers** |
| **667** | **Nct (2019)** | **Impact of Noise on Anesthesiologists' and Trainees' Situational Awareness in a High Fidelity Simulation Environment** | **[https://clinicaltrials.gov/show/NCT04138082](https://clinicaltrials.gov/show/NCT04138082)** | **Excluded** | **Review articles, conference papers** |
| **668** | **Nct (2019)** | **Impact of Vitamin D Supplementation on Cardiometabolic Status and Androgen Profile in Polycystic Ovary Syndrome** | **[https://clinicaltrials.gov/show/NCT04117750](https://clinicaltrials.gov/show/NCT04117750)** | **Excluded** | **Review articles, conference papers** |
| **669** | **Nct (2019)** | **Impacts of Two Rehabilitation Programs on Chronic Peripheral Facial Paresis** | **[https://clinicaltrials.gov/show/NCT04074018](https://clinicaltrials.gov/show/NCT04074018)** | **Excluded** | **Review articles, conference papers** |
| **670** | **Nct (2019)** | **Implementation of Uterine Fibroid Option Grid Patient Decision Aids Across Five Organizational Settings** | **[https://clinicaltrials.gov/show/NCT03985449](https://clinicaltrials.gov/show/NCT03985449)** | **Excluded** | **Review articles, conference papers** |
| **671** | **Nct (2019)** | **Nicotinamide Riboside for Treating Elevated Systolic Blood Pressure and Arterial Stiffness in Middle-aged and Older Adults** | **[https://clinicaltrials.gov/ct2/show/NCT03821623](https://clinicaltrials.gov/ct2/show/NCT03821623)** | **Excluded** | **Review articles, conference papers** |
| **672** | **Nct (2019)** | **Pelvic Health and Physical Therapy to Improve Lives of Prostate Cancer Patients Undergoing Prostatectomy** | **[https://clinicaltrials.gov/show/NCT04027270](https://clinicaltrials.gov/show/NCT04027270)** | **Excluded** | **Review articles, conference papers** |
| **673** | **Nct (2019)** | **Prevention of Hypoglycemia Among Diabetes Patients Admitted to Internal Medicine Departments With Nutritional Care** | **[https://clinicaltrials.gov/show/NCT03802942](https://clinicaltrials.gov/show/NCT03802942)** | **Excluded** | **Review articles, conference papers** |
| **674** | **Nct (2019)** | **Progressive Abduction Loading Therapy** | **[https://clinicaltrials.gov/ct2/show/NCT04118998](https://clinicaltrials.gov/ct2/show/NCT04118998)** | **Excluded** | **Review articles, conference papers** |
| **675** | **Nct (2019)** | **Protein Supplementation and Muscle Function in the Elderly** | **[https://clinicaltrials.gov/show/NCT03815201](https://clinicaltrials.gov/show/NCT03815201)** | **Excluded** | **Review articles, conference papers** |
| **676** | **Nct (2019)** | **Quality and Behavior of Pelvic Floor in Runner Women** | **[https://clinicaltrials.gov/ct2/show/NCT03934996](https://clinicaltrials.gov/ct2/show/NCT03934996)** | **Excluded** | **Review articles, conference papers** |
| **677** | **Nct (2019)** | **Randomized Clinical Trial on Skin Tags Approachment** | **[https://clinicaltrials.gov/show/NCT04161274](https://clinicaltrials.gov/show/NCT04161274)** | **Excluded** | **Review articles, conference papers** |
| **678** | **Nct (2019)** | **Telerehabilitation in Geriatric Patients at Aarhus University Hospital, Denmark** | **[https://clinicaltrials.gov/show/NCT03952858](https://clinicaltrials.gov/show/NCT03952858)** | **Excluded** | **Review articles, conference papers** |
| **679** | **Nct (2019)** | **Treating Idiopathic Inflammatory Myopathies Related Reduced Bone Mineral Density With Denosumab or Zoledronic Acid** | **[[https://clinicaltrials.gov/show/NCT04034199](https](https://clinicaltrials.gov/show/NCT04034199](https" \o "https://clinicaltrials.gov/show/NCT04034199](https)** | **Excluded** | **Review articles, conference papers** |
| **680** | **Nct (2019)** | **Urine Albendazole Levels for Coverage Assessment** | **[https://clinicaltrials.gov/show/NCT04041427](https://clinicaltrials.gov/show/NCT04041427)** | **Excluded** | **Review articles, conference papers** |
| **681** | **Nct (2020)** | **6 Weeks Plyometric Training on Vertical Jump and Agility in Relation to Gender Among Badminton Players** | **[https://clinicaltrials.gov/show/NCT04717206](https://clinicaltrials.gov/show/NCT04717206)** | **Excluded** | **Review articles, conference papers** |
| **682** | **Nct (2020)** | **Blood Flow Restriction Training After Patellar Instability** | **[https://clinicaltrials.gov/ct2/show/NCT04554212](https://clinicaltrials.gov/ct2/show/NCT04554212)** | **Excluded** | **Review articles, conference papers** |
| **683** | **Nct (2020)** | **A Comparison of Total Knee Replacement Patients Using the Zimmer-Biomet Persona Total Knee System With Different Inserts** | **[https://clinicaltrials.gov/show/NCT04643119](https://clinicaltrials.gov/show/NCT04643119)** | **Excluded** | **Review articles, conference papers** |
| **684** | **Nct (2020)** | **Gait-Training Using Wearable Sensors** | **[https://clinicaltrials.gov/show/NCT04270565](https://clinicaltrials.gov/show/NCT04270565)** | **Excluded** | **Review articles, conference papers** |
| **685** | **Nct (2020)** | **Investigation of the Effect of Instrument Assisted Soft Tissue Mobilization Technique in Individuals With Asymptomatic Dynamic Knee Valgus** | **[https://clinicaltrials.gov/show/NCT04660604](https://clinicaltrials.gov/show/NCT04660604)** | **Excluded** | **Review articles, conference papers** |
| **686** | **Nct (2020)** | **Kinematic- Versus Ligament-balanced Mechanical Alignment in TKA** | **[https://clinicaltrials.gov/show/NCT04436211](https://clinicaltrials.gov/show/NCT04436211)** | **Excluded** | **Review articles, conference papers** |
| **687** | **Nct (2020)** | **Characterization of AmnioExcel Plus in Two Treatment Paradigms** | **[https://clinicaltrials.gov/show/NCT04233580](https://clinicaltrials.gov/show/NCT04233580)** | **Excluded** | **Review articles, conference papers** |
| **688** | **Nct (2020)** | **Clinical Outcomes Between Tibial Preservation Bone Cut and Conventional Tibial Bone Cut Following Medial UKA** | **[https://clinicaltrials.gov/show/NCT04419116](https://clinicaltrials.gov/show/NCT04419116)** | **Excluded** | **Review articles, conference papers** |
| **689** | **Nct (2020)** | **The Comparison of Hip and Knee Focused Exercises Versus Hip and Knee Focused Exercises With the Use of Blood Flow Restriction Training in Adults With Patellofemoral Pain** | **[https://clinicaltrials.gov/show/NCT04340453](https://clinicaltrials.gov/show/NCT04340453)** | **Excluded** | **Review articles, conference papers** |
| **690** | **Nct (2020)** | **Comparisons of Different Forms of Glucocorticoid on the Recovery of Reproductive Function in Patients With 21α-hydroxylase Deficiency** | **[https://clinicaltrials.gov/show/NCT04536662](https://clinicaltrials.gov/show/NCT04536662)** | **Excluded** | **Review articles, conference papers** |
| **691** | **Nct (2020)** | **Continuous Passive Motion Following Fixation of Pelvic and Knee Fractures** | **[https://clinicaltrials.gov/show/NCT04389749](https://clinicaltrials.gov/show/NCT04389749)** | **Excluded** | **Review articles, conference papers** |
| **692** | **Nct (2020)** | **Effect of Manual Therapy on Tibiotarsal Joint Mobility in Diabetic Individuals** | **[https://clinicaltrials.gov/show/NCT04372810](https://clinicaltrials.gov/show/NCT04372810)** | **Excluded** | **Review articles, conference papers** |
| **693** | **Nct (2020)** | **Effect of Music Therapy in Improving the Physical Fitness and Depression in the Frailty of the Community Elderly** | **[https://clinicaltrials.gov/show/NCT04799574](https://clinicaltrials.gov/show/NCT04799574)** | **Excluded** | **Review articles, conference papers** |
| **694** | **Nct (2020)** | **Effectiveness of Basic Body Awareness Therapy in Post-traumatic Stress Disorders: a Randomized Clinical Trial** | **[https://clinicaltrials.gov/show/NCT04396314](https://clinicaltrials.gov/show/NCT04396314)** | **Excluded** | **Review articles, conference papers** |
| **695** | **Nct (2020)** | **Effects of Testosterone Plus Dutasteride or Placebo on Muscle Strength, Body Composition and Metabolism in Transmen** | **[https://clinicaltrials.gov/show/NCT04545450](https://clinicaltrials.gov/show/NCT04545450)** | **Excluded** | **Review articles, conference papers** |
| **696** | **Nct (2020)** | **Efficacy of Eccentric Versus Isometric Exercise in Reducing Pain in Runners With Proximal Hamstring Tendinopathy** | **[https://clinicaltrials.gov/show/NCT04683107](https://clinicaltrials.gov/show/NCT04683107)** | **Excluded** | **Review articles, conference papers** |
| **697** | **Nct (2020)** | **The Efficacy of Kinesio Taping in the Treatment of Nonspecific Acute Low Back Pain** | **[https://clinicaltrials.gov/show/NCT04263740](https://clinicaltrials.gov/show/NCT04263740)** | **Excluded** | **Review articles, conference papers** |
| **698** | **Nct (2020)** | **Electroacupuncture vs Topical Diclofenac Sodium Gel for Patients With Hand Osteoarthritis** | **[https://clinicaltrials.gov/show/NCT04402047](https://clinicaltrials.gov/show/NCT04402047)** | **Excluded** | **Review articles, conference papers** |
| **699** | **Nct (2020)** | **Follow-up and Outcome of Operative Treatment With Decompressive Release Of The Peroneal Nerve** | **[https://clinicaltrials.gov/ct2/show/NCT04695834](https://clinicaltrials.gov/ct2/show/NCT04695834)** | **Excluded** | **Review articles, conference papers** |
| **700** | **Nct (2020)** | **Full Metall Jacket Multilevel Segment** | **[https://clinicaltrials.gov/show/NCT04461496](https://clinicaltrials.gov/show/NCT04461496)** | **Excluded** | **Review articles, conference papers** |
| **701** | **Nct (2020)** | **Influence of Patient Sex on Pain Control and Multimodal Analgesia in Total Knee Arthroplasty** | **[https://clinicaltrials.gov/ct2/show/NCT04471233](https://clinicaltrials.gov/ct2/show/NCT04471233)** | **Excluded** | **Review articles, conference papers** |
| **702** | **Nct (2020)** | **Mini-Open Direct-anterior Approach vs Hip Arthroscopy for Treatment of Femoroacetabular Impingement** | **[https://clinicaltrials.gov/show/NCT04638114](https://clinicaltrials.gov/show/NCT04638114)** | **Excluded** | **Review articles, conference papers** |
| **703** | **Nct (2020)** | **Patellar Resurfacing in Total Knee Arthroplasty Leads to Better Isokinetic Performance and Higher Clinical Scores** | **[https://clinicaltrials.gov/show/NCT04637490](https://clinicaltrials.gov/show/NCT04637490)** | **Excluded** | **Review articles, conference papers** |
| **704** | **Nct (2020)** | **Peanut Protein Supplementation to Prevent Muscle Atrophy and Improve Recovery Following Total Knee Arthroplasty** | **[https://clinicaltrials.gov/show/NCT04294563](https://clinicaltrials.gov/show/NCT04294563)** | **Excluded** | **Review articles, conference papers** |
| **705** | **Nct (2020)** | **Percutaneous Microelectrolysis in Agility, Joint Range and Strength** | **[https://clinicaltrials.gov/show/NCT04334772](https://clinicaltrials.gov/show/NCT04334772)** | **Excluded** | **Review articles, conference papers** |
| **706** | **Nct (2020)** | **Peri-articular Injection and (IPACK) With Adductor Canal Block in Total Knee Arthroplasty** | **[https://clinicaltrials.gov/show/NCT04396652](https://clinicaltrials.gov/show/NCT04396652)** | **Excluded** | **Review articles, conference papers** |
| **707** | **Nct (2020)** | **Preventing Knee Osteoarthritis Through Exercise and Education Following Knee Injury** | **[https://clinicaltrials.gov/show/NCT04363476](https://clinicaltrials.gov/show/NCT04363476)** | **Excluded** | **Review articles, conference papers** |
| **708** | **Nct (2020)** | **Prevention of Lower Limb and Groin Injuries: the Sport Without Injury ProgrammE (SWIPE) Football Trial** | **[https://clinicaltrials.gov/show/NCT04272047](https://clinicaltrials.gov/show/NCT04272047)** | **Excluded** | **Review articles, conference papers** |
| **709** | **Nct (2020)** | **Promotion and Support for Physical Activity Maintenance Post Total Hip Arthroplasty** | **[https://clinicaltrials.gov/ct2/show/NCT04471532](https://clinicaltrials.gov/ct2/show/NCT04471532)** | **Excluded** | **Review articles, conference papers** |
| **710** | **Nct (2020)** | **Pulsed Electromagnetic Field in Haemophilia** | **[https://clinicaltrials.gov/show/NCT04590456](https://clinicaltrials.gov/show/NCT04590456)** | **Excluded** | **Review articles, conference papers** |
| **711** | **Nct (2020)** | **A Randomized RSA Study Comparing HXLPE to Conventional Polyethylene in Cemented Total Hip Arthroplasty** | **[https://clinicaltrials.gov/show/NCT04322799](https://clinicaltrials.gov/show/NCT04322799)** | **Excluded** | **Review articles, conference papers** |
| **712** | **Nct (2020)** | **SEQUAR SEmitendinosus vs QUadriceps in Anterior Cruciate Ligament Reconstruction** | **[https://clinicaltrials.gov/show/NCT04295148](https://clinicaltrials.gov/show/NCT04295148)** | **Excluded** | **Review articles, conference papers** |
| **713** | **Nct (2020)** | **Smart Care for Older Persons Recovering From Hip-fracture Surgery** | **[https://clinicaltrials.gov/show/NCT04675411](https://clinicaltrials.gov** | **Excluded** | **Review articles, conference papers** |
| **714** | **Nct (2020)** | **Spinal Anesthetic for Hip and Knee Replacement Surgery - Intrathecal Morphine or Hydromorphone** | **[https://clinicaltrials.gov/show/NCT04280939](https://clinicaltrials.gov/show/NCT04280939)** | **Excluded** | **Review articles, conference papers** |
| **715** | **Nct (2020)** | **Strength Training in Hypoxia to Improve Bone and Cardiovascular Health of Elderly** | **[https://clinicaltrials.gov/show/NCT04281264](https://clinicaltrials.gov/show/NCT04281264)** | **Excluded** | **Review articles, conference papers** |
| **716** | **Nct (2020)** | **The Use of Cannabinoid Patch for Knee Osteoarthritis** | **[https://clinicaltrials.gov/show/NCT04412837](https://clinicaltrials.gov/show/NCT04412837)** | **Excluded** | **Review articles, conference papers** |
| **717** | **Nct (2020)** | **Using Omics Technology to Explore the Mechanism of Acupuncture Treatment of Different Acupoints of Knee Osteoarthritis** | **[https://clinicaltrials.gov/ct2/show/NCT04733352](https://clinicaltrials.gov/ct2/show/NCT04733352)** | **Excluded** | **Review articles, conference papers** |
| **718** | **Nct (2020)** | **The Viscoelastic Properties of Lower Extremity's Muscles** | **[https://clinicaltrials.gov/show/NCT04289129](https://clinicaltrials.gov/show/NCT04289129)** | **Excluded** | **Review articles, conference papers** |
| **719** | **Nct (2020)** | **Weighted Rope Training in Taekwando Athletes** | **[https://clinicaltrials.gov/show/NCT04272164](https://clinicaltrials.gov/show/NCT04272164)** | **Excluded** | **Review articles, conference papers** |
| **720** | **Nct et al** | **Auricular Vagus Nerve Stimulation For Fibromyalgia Syndrome** | **[https://clinicaltrials.gov/show/NCT04260906](https://clinicaltrials.gov/show/NCT04260906)** | **Excluded** | **Review articles, conference papers** |
| **721** | **Nct et al** | **BFRT in Adolescents After ACL Reconstruction** | **[https://clinicaltrials.gov/show/NCT04390035](https://clinicaltrials.gov/show/NCT04390035)** | **Excluded** | **Review articles, conference papers** |
| **722** | **Nct et al** | **Blood Flow Restriction Training (BFR) in an Adolescent Population** | **[https://clinicaltrials.gov/ct2/show/NCT04285879](https://clinicaltrials.gov/ct2/show/NCT04285879)** | **Excluded** | **Review articles, conference papers** |
| **723** | **Nct et al** | **Comparing Two Different Methods to Prescribe Exercise** | **[https://clinicaltrials.gov/show/NCT04286919](https://clinicaltrials.gov/show/NCT04286919)** | **Excluded** | **Review articles, conference papers** |
| **724** | **Nct et al** | **Comparison of Ozone and Steroid Injection in Patients With Greater Trochanteric Pain Syndrome** | **[https://clinicaltrials.gov/show/NCT04420572](https://clinicaltrials.gov/show/NCT04420572)** | **Excluded** | **Review articles, conference papers** |
| **725** | **Nct et al** | **Comparison of Partial Rotator Cuff Repair vs. Superior Capsular Reconstruction for Irreparable Rotator Cuff Tears** | **[https://clinicaltrials.gov/ct2/show/NCT04742452](https://clinicaltrials.gov/ct2/show/NCT04742452)** | **Excluded** | **Review articles, conference papers** |
| **726** | **Nct et al** | **Does a Video-based Education Tool Related to Strengthening Result in Different Functional Outcomes Compared to Traditional Physical Therapy Education for Patients?** | **[https://clinicaltrials.gov/show/NCT04245852](https://clinicaltrials.gov/show/NCT04245852)** | **Excluded** | **Review articles, conference papers** |
| **727** | **Nct et al** | **Early Diagnosis of Compartment Syndrome by Multimodal Detection Technique** | **[https://clinicaltrials.gov/show/NCT04442672](https://clinicaltrials.gov/show/NCT04442672)** | **Excluded** | **Review articles, conference papers** |
| **728** | **Nct et al** | **The Effect of Lifestyle Intervention on Cardiovascular Disease Risk Among Women** | **[https://clinicaltrials.gov/show/NCT04601558](https://clinicaltrials.gov/show/NCT04601558)** | **Excluded** | **Review articles, conference papers** |
| **729** | **Nct et al** | **The Effect of Massage, Wipe Bathing and Tub Bathing on Physiological Measurements of Late Premature Newborns** | **[https://clinicaltrials.gov/show/NCT04602130](https://clinicaltrials.gov/show/NCT04602130)** | **Excluded** | **Review articles, conference papers** |
| **730** | **Nct et al** | **Effect of Metformin on Non PCO Women Undergoing IVF/ICSI** | **[https://clinicaltrials.gov/ct2/show/NCT04489147](https://clinicaltrials.gov/ct2/show/NCT04489147)** | **Excluded** | **Review articles, conference papers** |
| **731** | **Nct et al** | **Effectiveness of SCDM in Patients With Type 2 Diabetes** | **[https://clinicaltrials.gov/show/NCT04259489](https://clinicaltrials.gov/show/NCT04259489)** | **Excluded** | **Review articles, conference papers** |
| **732** | **Nct et al** | **Effects of Whole-Body Electromyostimulation Application in Individuals With Lumbar Disc Hernia** | **[https://clinicaltrials.gov/show/NCT04329598](https://clinicaltrials.gov/show/NCT04329598)** | **Excluded** | **Review articles, conference papers** |
| **733** | **Nct et al** | **Efficacy Of Magnesium In Radicular Pain When Added To Local Anesthetics And Steroids In Transforaminal Epidural Injection** | **[https://clinicaltrials.gov/show/NCT04532775](https://clinicaltrials.gov/show/NCT04532775)** | **Excluded** | **Review articles, conference papers** |
| **734** | **Nct et al** | **Efficacy of Mouthwash in Reducing Salivary Carriage of COVID-19** | **[https://clinicaltrials.gov/show/NCT04603794](https://clinicaltrials.gov/show/NCT04603794)** | **Excluded** | **Review articles, conference papers** |
| **735** | **Nct et al** | **Efficacy of Negative Pressure Wound Closure Therapy by PICO System in Prevention of Complications of Femoral Artery Exposure** | **[https://clinicaltrials.gov/show/NCT04453319](https://clinicaltrials.gov/show/NCT04453319)** | **Excluded** | **Review articles, conference papers** |
| **736** | **Nct et al** | **Home-based HIIT in a Primary-care Setting for at Risk Individuals: a Multidisciplinary Approach** | **[https://clinicaltrials.gov/show/NCT04553614](https://clinicaltrials.gov/show/NCT04553614)** | **Excluded** | **Review articles, conference papers** |
| **737** | **Nct et al** | **Intensive Glycemic Control For Diabetic Foot Ulcer Healing** | **[https://clinicaltrials.gov/show/NCT04323462](https://clinicaltrials.gov/show/NCT04323462)** | **Excluded** | **Review articles, conference papers** |
| **738** | **Nct et al** | **Livestock for Health Project** | **[https://clinicaltrials.gov/show/NCT04608656](https://clinicaltrials.gov/show/NCT04608656)** | **Excluded** | **Review articles, conference papers** |
| **739** | **Nct et al** | **Long-term Effects of the New Nordic Renal Diet in Patients With Moderate Chronic Kidney Disease** | **[https://clinicaltrials.gov/ct2/show/NCT04579315](https://clinicaltrials.gov/ct2/show/NCT04579315)** | **Excluded** | **Review articles, conference papers** |
| **740** | **Nct et al** | **Mechanisms of Fatigability With Diabetes** | **[https://clinicaltrials.gov/ct2/show/NCT04442451](https://clinicaltrials.gov/ct2/show/NCT04442451)** | **Excluded** | **Review articles, conference papers** |
| **741** | **Nct et al** | **Perineural Local Anesthetic Administration With a Continuous Infusion Versus Automatic Intermittent Boluses** | **[https://clinicaltrials.gov/show/NCT04458467](https://clinicaltrials.gov/show/NCT04458467)** | **Excluded** | **Review articles, conference papers** |
| **742** | **Nct et al** | **Phase 1 Cardiac Rehabilitation With and Without Lower Limb Paddling Effects in Post CABG Patients** | **[https://clinicaltrials.gov/show/NCT04556994](https://clinicaltrials.gov/show/NCT04556994)** | **Excluded** | **Review articles, conference papers** |
| **743** | **Nct et al** | **Polidocanol Foam VS Artery Ligation in Hemorrhoidal Disease** | **[https://clinicaltrials.gov/show/NCT04675177](https://clinicaltrials.gov/show/NCT04675177)** | **Excluded** | **Review articles, conference papers** |
| **744** | **Nct et al** | **Preventing Bed-rest Induced Muscle Loss in the Elderly** | **[https://clinicaltrials.gov/show/NCT04422665](https://clinicaltrials.gov/show/NCT04422665)** | **Excluded** | **Review articles, conference papers** |
| **745** | **Nct et al** | **Prevention of Hand-foot Skin Reaction** | **[https://clinicaltrials.gov/show/NCT04568330](https://clinicaltrials.gov/show/NCT04568330)** | **Excluded** | **Review articles, conference papers** |
| **746** | **Nct et al** | **Pyridostigmine in Severe SARS-CoV-2 Infection** | **[https://clinicaltrials.gov/show/NCT04343963](https://clinicaltrials.gov/show/NCT04343963)** | **Excluded** | **Review articles, conference papers** |
| **747** | **Nct et al** | **Regeneration in Cervical Degenerative Myelopathy** | **[https://clinicaltrials.gov/show/NCT04631471](https://clinicaltrials.gov/show/NCT04631471)** | **Excluded** | **Review articles, conference papers** |
| **748** | **Nct et al** | **The Relationship Between Cold Water Immersion and the Progression in Gout Arthritis** | **[https://clinicaltrials.gov/show/NCT04587544](https://clinicaltrials.gov/show/NCT04587544)** | **Excluded** | **Review articles, conference papers** |
| **749** | **Nct et al** | **Role of Blood Management in Perioperative Outcomes** | **[https://clinicaltrials.gov/ct2/show/NCT04475497](https://clinicaltrials.gov/ct2/show/NCT044754** | **Excluded** | **Review articles, conference papers** |
| **750** | **Nct et al** | **Safety, Tolerability and Efficacy of Nefopam Cream in Burn Patients** | **[https://clinicaltrials.gov/ct2/show/NCT04685577](https://clinicaltrials.gov/ct2/show/NCT04685577)** | **Excluded** | **Review articles, conference papers** |
| **751** | **Nct et al** | **Self-care Intervention for Reducing Rehospitalization for Heart Failure: a Randomized Clinical Trial (SIHF)** | **[https://clinicaltrials.gov/show/NCT04870918](https://clinicaltrials.gov/show/NCT04870918)** | **Excluded** | **Review articles, conference papers** |
| **752** | **Nct et al** | **The Short-term Effects of Instrument-Based Mobilization Compared With Manual Mobilization for Low Back Pain: a Randomized Clinical Trial** | **[https://clinicaltrials.gov/show/NCT04367376](https://clinicaltrials.gov/show/NCT04367376)** | **Excluded** | **Review articles, conference papers** |
| **753** | **Nct et al** | **Error Augmentation Motor Learning Training Approach in Stroke Patients** | **[https://clinicaltrials.gov/show/NCT04378946](https://clinicaltrials.gov/show/NCT04378946)** | **Excluded** | **Review articles, conference papers** |
| **754** | **Nct et al** | **Adapting REhabilitation Delivery for Maximum Impact at Home** | **[https://clinicaltrials.gov/ct2/show/NCT05164575](https://clinicaltrials.gov/ct2/show/NCT05164575)** | **Excluded** | **Review articles, conference papers** |
| **755** | **Nct et al** | **Anterior Knee Pain Between Unisex Knee Prosthesis VS Gender Specific Knee Prosthesis Following MIS TKA** | **[https://clinicaltrials.gov/show/NCT05045651](https://clinicaltrials.gov/show/NCT05045651)** | **Excluded** | **Review articles, conference papers** |
| **756** | **Nct et al** | **Comparison of Sciatic Nerve Gliding and Lower Extremity Dynamic Stretch on Hamstring Flexibility of Athletes** | **[https://clinicaltrials.gov/show/NCT05136469](https://clinicaltrials.gov/show/NCT05136469)** | **Excluded** | **Review articles, conference papers** |
| **757** | **Nct et al** | **Comparison of the Efficacy of Russian, Aussie Currents With Isokinetic Exercise Patellofemoral Pain Syndrome** | **[https://clinicaltrials.gov/show/NCT04945759](https://clinicaltrials.gov/show/NCT04945759)** | **Excluded** | **Review articles, conference papers** |
| **758** | **Nct et al** | **The Effect of Elastic Bandage Compression on Pain and Function in Individuals With Knee Osteoarthritis** | **[https://clinicaltrials.gov/show/NCT04724902](https://clinicaltrials.gov/show/NCT04724902)** | **Excluded** | **Review articles, conference papers** |
| **759** | **Nct et al** | **The Effect of Functional Exercise Training on Patients With Hypertension** | **[https://clinicaltrials.gov/show/NCT05187702](https://clinicaltrials.gov/show/NCT05187702)** | **Excluded** | **Review articles, conference papers** |
| **760** | **Nct et al** | **Effect of Iontophoresis vs. Ultrasound in Plantar Fasciitis** | **[https://clinicaltrials.gov/show/NCT04917406](https://clinicaltrials.gov/show/NCT04917406)** | **Excluded** | **Review articles, conference papers** |
| **761** | **Nct et al** | **Effect of Reparel Knee Sleeve With Knee Injection** | **[https://clinicaltrials.gov/ct2/show/NCT04859764](https://clinicaltrials.gov/ct2/show/NCT04859764)** | **Excluded** | **Review articles, conference papers** |
| **762** | **Nct et al** | **Effect of UC Versus EA on Sex Hormones in Obese Infertile Patients With PCOs** | **[https://clinicaltrials.gov/show/NCT04875312](https://clinicaltrials.gov/show/NCT04875312)** | **Excluded** | **Review articles, conference papers** |
| **763** | **Nct et al** | **Effectiveness of Focal Vibration and Blood Flow Restriction Within a Multicomponent Exercise Programme** | **[https://clinicaltrials.gov/show/NCT04940702](https://clinicaltrials.gov/show/NCT04940702)** | **Excluded** | **Review articles, conference papers** |
| **764** | **Nct et al** | **Effects of a Physiotherapist-delivered Dietary Weight Loss Program in People With Knee OA Who Have Overweight/Obesity** | **[https://clinicaltrials.gov/ct2/show/NCT04733053](https://clinicaltrials.gov/ct2/show/NCT04733053)** | **Excluded** | **Review articles, conference papers** |
| **765** | **Nct et al** | **Effects of Gait Rehabilitation With Motor Imagery in People With Parkinson's Disease** | **[https://clinicaltrials.gov/ct2/show/NCT04788693](https://clinicaltrials.gov/ct2/show/NCT04788693)** | **Excluded** | **Review articles, conference papers** |
| **766** | **Nct et al** | **Effects of Home Rehabilitation of Balance Based on Functional Exercises in People With Parkinson's Disease** | **[https://clinicaltrials.gov/show/NCT04963894](https://clinicaltrials.gov/show/NCT04963894)** | **Excluded** | **Review articles, conference papers** |
| **767** | **Nct et al** | **Efficacy and Safety Study of Ibuprofen Gel Compared to Placebo in the Treatment of Acute Musculoskeletal Pain** | **[https://clinicaltrials.gov/show/NCT05013567](https://clinicaltrials.gov/show/NCT05013567)** | **Excluded** | **Review articles, conference papers** |
| **768** | **Nct et al** | **Efficiency Assessment of the Methodology for the Follow-up of Patients With Knee Prostheses** | **[https://clinicaltrials.gov/ct2/show/NCT04850300](https://clinicaltrials.gov/ct2/show/NCT04850300)** | **Excluded** | **Review articles, conference papers** |
| **769** | **Nct et al** | **Exercise Program After Total Hip and Knee Replacement: a Randomized Controlled Trial** | **[https://clinicaltrials.gov/ct2/show/NCT04761367](https://clinicaltrials.gov/ct2/show/NCT04761367)** | **Excluded** | **Review articles, conference papers** |
| **770** | **Nct et al** | **Heat Therapy to Prevent Deconditioning During Immobilization** | **[https://clinicaltrials.gov/show/NCT05021523](https://clinicaltrials.gov/show/NCT05021523)** | **Excluded** | **Review articles, conference papers** |
| **771** | **Nct et al** | **Hip Joint Mobilizations and Strength Training in Patients With Knee OA Osteoarthritis** | **[https://clinicaltrials.gov/show/NCT04769531](https://clinicaltrials.gov/show/NCT04769531)** | **Excluded** | **Review articles, conference papers** |
| **772** | **Nct et al** | **KINCISE™ Surgical Automated System in Total Hip Arthroplasty (THA)** | **[https://clinicaltrials.gov/show/NCT05223777](https://clinicaltrials.gov/show/NCT05223777)** | **Excluded** | **Review articles, conference papers** |
| **773** | **Nct et al** | **Laser Therapy on Chronic Knee Joint Osteoarthritis Patients** | **[https://clinicaltrials.gov/show/NCT04875689](https://clinicaltrials.gov/show/NCT04875689)** | **Excluded** | **Review articles, conference papers** |
| **774** | **Nct et al** | **Men and Women With Different Designs and Semi-individual Total Knee Replacement** | **[https://clinicaltrials.gov/show/NCT04976400](https://clinicaltrials.gov/show/NCT04976400)** | **Excluded** | **Review articles, conference papers** |
| **775** | **Nct et al** | **Myofascial Release With and Without METS of Gluteus Maximus and Tensor Fascia Lata in ITB Syndrome** | **[https://clinicaltrials.gov/show/NCT04954703](https://clinicaltrials.gov/show/NCT04954703)** | **Excluded** | **Review articles, conference papers** |
| **776** | **Nct et al** | **Pericapsular Nerve Group Block (PENG) for Hip Surgery** | **[https://clinicaltrials.gov/show/NCT05118620](https://clinicaltrials.gov/show/NCT05118620)** | **Excluded** | **Review articles, conference papers** |
| **777** | **Nct et al** | **Postoperative Hypothermia Control In Older Patients With Total Knee Arthroplasty: effect Of Electric And Woolen Blanket** | **[https://clinicaltrials.gov/show/NCT05389579](https://clinicaltrials.gov/show/NCT05389579)** | **Excluded** | **Review articles, conference papers** |
| **778** | **Nct et al** | **Preventing Injuries in Young Football Players** | **[https://clinicaltrials.gov/show/NCT05137015](https://clinicaltrials.gov/show/NCT05137015)** | **Excluded** | **Review articles, conference papers** |
| **779** | **Nct et al** | **Ropivacaine and Midazolam by Intraarticular vs Epidural Administration in Arthroscopic ACL** | **[https://clinicaltrials.gov/show/NCT05078372](https://clinicaltrials.gov/show/NCT05078372)** | **Excluded** | **Review articles, conference papers** |
| **780** | **Nct et al** | **Short Term Immobilization of the Lower Limb** | **[https://clinicaltrials.gov/show/NCT05072652](https://clinicaltrials.gov/show/NCT05072652)** | **Excluded** | **Review articles, conference papers** |
| **781** | **Nct et al** | **The SOAR (Stop OsteoARthritis) Program Proof-of-Concept Study** | **[https://clinicaltrials.gov/ct2/show/NCT04956393](https://clinicaltrials.gov/ct2/show/NCT04956393)** | **Excluded** | **Review articles, conference papers** |
| **782** | **Nct et al** | **Topical Vancomycin for Infection Prophylaxis in TJA** | **[https://clinicaltrials.gov/ct2/show/NCT04993027](https://clinicaltrials.gov/ct2/show/NCT04993027)** | **Excluded** | **Review articles, conference papers** |
| **783** | **Nct et al** | **Treadmill With Ankle Weights on Balance in Spastic Cerebral Palsied Children** | **[https://clinicaltrials.gov/show/NCT05106829](https://clinicaltrials.gov/show/NCT05106829)** | **Excluded** | **Review articles, conference papers** |
| **784** | **Nct et al** | **Use of the OPTIMAL Theory of Motor Learning With the Lower Quarter Y-Balance Test** | **[https://clinicaltrials.gov/show/NCT05113797](https://clinicaltrials.gov/show/NCT05113797)** | **Excluded** | **Review articles, conference papers** |
| **785** | **Nct et al** | **Video Aided Mindful Deep Breathing for Pain Management** | **[https://clinicaltrials.gov/show/NCT04812158](https://clinicaltrials.gov/show/NCT04812158)** | **Excluded** | **Review articles, conference papers** |
| **786** | **Nct et al** | **Balance and Gait in Diabetic Neuropathy** | **[https://clinicaltrials.gov/show/NCT05127538](https://clinicaltrials.gov/show/NCT05127538)** | **Excluded** | **Review articles, conference papers** |
| **787** | **Nct et al** | **Cardiopulmonary Function and Quality of Life in Pulmonary Tuberculosis** | **[https://clinicaltrials.gov/ct2/show/NCT04844502](https://clinicaltrials.gov/ct2/show/NCT04844502)** | **Excluded** | **Review articles, conference papers** |
| **788** | **Nct et al** | **Different Treatment Methods in Patients With Plantar Fasciitis** | **[https://clinicaltrials.gov/show/NCT05011695](https://clinicaltrials.gov/show/NCT05011695)** | **Excluded** | **Review articles, conference papers** |
| **789** | **Nct et al** | **Effect of Branch Chain Amino Acid Therapy on Sarcopenia in Children With Chronic Liver Disease** | **[https://clinicaltrials.gov/show/NCT05093218](https://clinicaltrials.gov/show/NCT05093218)** | **Excluded** | **Review articles, conference papers** |
| **790** | **Nct et al** | **EFFECT OF FLOW RESTRICTION ON BONE QUALITY WITHIN A MULTICOMPONENT EXERCISE PROGRAM FOR OLDER WOMEN WITH OSTEOPOROSIS** | **[https://clinicaltrials.gov/show/NCT04760145](https://clinicaltrials.gov/show/NCT04760145)** | **Excluded** | **Review articles, conference papers** |
| **791** | **Nct et al** | **Effect of Life Kinetik Training on Lower Limb Coordination in Ataxic Patients** | **[https://clinicaltrials.gov/ct2/show/NCT06001424](https://clinicaltrials.gov/ct2/show/NCT06001424)** | **Excluded** | **Review articles, conference papers** |
| **792** | **Nct et al** | **Effectiveness of Client-centered Intervention in After Total Knee Arthroplasty** | **[https://clinicaltrials.gov/show/NCT04969432](https://clinicaltrials.gov/show/NCT04969432)** | **Excluded** | **Review articles, conference papers** |
| **793** | **Nct et al** | **Effectiveness of Specifically Optimized Off-the-counter Foot Orthosis for the Subtle Cavus Foot** | **[https://clinicaltrials.gov/show/NCT04941469](https://clinicaltrials.gov/show/NCT04941469)** | **Excluded** | **Review articles, conference papers** |
| **794** | **Nct et al** | **Effects of a Weight Loss Program in People With Hip Osteoarthritis** | **[https://clinicaltrials.gov/ct2/show/NCT04825483](https://clinicaltrials.gov/ct2/show/NCT04825483)** | **Excluded** | **Review articles, conference papers** |
| **795** | **Nct et al** | **Effects of External Neuromuscular Electrical Stimulation in Women With Urgency Urinary Incontinence** | **[https://clinicaltrials.gov/show/NCT04727983](https://clinicaltrials.gov/show/NCT04727983)** | **Excluded** | **Review articles, conference papers** |
| **796** | **Nct et al** | **The Efficacy of Adding Dexmedetomidine Perineurally to Bupivacaine in Ultrasound Guided Fascia Iliaca Block Versus Intravenously Infused Dexmedetomidine on Hemodynamic Stability Intraoperatively and Postoperative Analgesia Following Hip Arthroscopy** | **[https://clinicaltrials.gov/show/NCT04917029](https://clinicaltrials.gov/show/NCT04917029)** | **Excluded** | **Review articles, conference papers** |
| **797** | **Nct et al** | **Evaluation of the Sural Nerve With Ultrasonography and Electromyography in Patients With Fibromyalgia** | **[https://clinicaltrials.gov/show/NCT04985149](https://clinicaltrials.gov/show/NCT04985149)** | **Excluded** | **Review articles, conference papers** |
| **798** | **Nct et al** | **Feasibility of Prehab for Lumbar Spinal Stenosis** | **[https://clinicaltrials.gov/ct2/show/NCT05073081](https://clinicaltrials.gov/ct2/show/NCT05073081)** | **Excluded** | **Review articles, conference papers** |
| **799** | **Nct et al** | **Foot Orthoses in Patients With Sciatica** | **[https://clinicaltrials.gov/ct2/show/NCT05129540](https://clinicaltrials.gov/ct2/show/NCT05129540)** | **Excluded** | **Review articles, conference papers** |
| **800** | **Nct et al** | **Hydrotherapy Versus Classical Rehabilitation After Surgical Rotator Cuff Repair** | **[https://clinicaltrials.gov/show/NCT05106842](https://clinicaltrials.gov/show/NCT05106842)** | **Excluded** | **Review articles, conference papers** |
| **801** | **Nct et al** | **Individualised Postprandial Glucose Responses in Type 1 Diabetes** | **[https://clinicaltrials.gov/show/NCT05231642](https://clinicaltrials.gov/show/NCT05231642)** | **Excluded** | **Review articles, conference papers** |
| **802** | **Nct et al** | **iPACK Block With Dexamethasone For Total Knee Replacement** | **[https://clinicaltrials.gov/show/NCT04917055](https://clinicaltrials.gov/show/NCT04917055)** | **Excluded** | **Review articles, conference papers** |
| **803** | **Nct et al** | **Lumbopelvic Movement Control: effect of Injury History, and the Role of Cortical Control and Its Practical Application 2** | **[https://clinicaltrials.gov/show/NCT04861350](https://clinicaltrials.gov/show/NCT04861350)** | **Excluded** | **Review articles, conference papers** |
| **804** | **Nct et al** | **Mediterranean Enriched Diet for Tackling Youth Obesity** | **[https://clinicaltrials.gov/ct2/show/NCT04719052](https://clinicaltrials.gov/ct2/show/NCT04719052)** | **Excluded** | **Review articles, conference papers** |
| **805** | **Nct et al** | **Microcurrent for Fibromyalgia** | **[https://clinicaltrials.gov/ct2/show/NCT04949100](https://clinicaltrials.gov/ct2/show/NCT04949100)** | **Excluded** | **Review articles, conference papers** |
| **806** | **Nct et al** | **Motor & Autonomic Concomitant Health Improvements With Neuromodulation & Exercise Training: an SCI RCT** | **[https://clinicaltrials.gov/ct2/show/NCT04726059](https://clinicaltrials.gov/ct2/show/NCT04726059)** | **Excluded** | **Review articles, conference papers** |
| **807** | **Nct et al** | **Motor Imagery Training in Community Dwelling Elderly** | **[https://clinicaltrials.gov/show/NCT04919044](https://clinicaltrials.gov/show/NCT04919044)** | **Excluded** | **Review articles, conference papers** |
| **808** | **Nct et al** | **OLP to Manage Pain in TJA: a Feasibility Study** | **[https://clinicaltrials.gov/ct2/show/NCT04883983](https://clinicaltrials.gov/ct2/show/NCT04883983)** | **Excluded** | **Review articles, conference papers** |
| **809** | **Nct et al** | **Online Mindfulness-based Intervention to Prevent Chronic Pain** | **[https://clinicaltrials.gov/show/NCT04848428](https://clinicaltrials.gov/show/NCT04848428)** | **Excluded** | **Review articles, conference papers** |
| **810** | **Nct et al** | **Oxidative Stress and Surgical Recovery** | **[https://clinicaltrials.gov/show/NCT04732000](https://clinicaltrials.gov/show/NCT04732000)** | **Excluded** | **Review articles, conference papers** |
| **811** | **Nct et al** | **ParentText User Engagement and Effectiveness Study** | **[https://clinicaltrials.gov/ct2/show/NCT05003518](https://clinicaltrials.gov/ct2/show/NCT05003518)** | **Excluded** | **Review articles, conference papers** |
| **812** | **Nct et al** | **Patient-Titrated Automated Intermittent Boluses of Local Anesthetic vs. a Continuous Infusion Via a Perineural Catheter for Postoperative Analgesia** | **[https://clinicaltrials.gov/ct2/show/NCT05091905](https://clinicaltrials.gov/ct2/show/NCT05091905)** | **Excluded** | **Review articles, conference papers** |
| **813** | **Nct et al** | **Resistance Training Effects on Muscle Morphological, Mechanical and Contractile Properties** | **[https://clinicaltrials.gov/show/NCT04845295](https://clinicaltrials.gov/show/NCT04845295)** | **Excluded** | **Review articles, conference papers** |
| **814** | **Nct et al** | **ROBERT® as an Intervention to Enhance Muscle Strength After Spinal Cord Injury** | **[https://clinicaltrials.gov/ct2/show/NCT05558254](https://clinical** | **Excluded** | **Review articles, conference papers** |
| **815** | **Nct et al** | **A Study to Evaluate Dimolegin in Prevention of Thromboembolic Complications During Knee Replacement** | **[https://clinicaltrials.gov/show/NCT05189002](https://clinicaltrials.gov/show/NCT05189002)** | **Excluded** | **Review articles, conference papers** |
| **816** | **Nct et al** | **Study to Investigate the Effect of Hypoestes Rosea Powder in Parkinson's Disease** | **[https://clinicaltrials.gov/show/NCT04858074](https://clinicaltrials.gov/show/NCT04858074)** | **Excluded** | **Review articles, conference papers** |
| **817** | **Nct et al** | **Ultrasound-Guided Percutaneous Peripheral Nerve Stimulation: a Department of Defense Funded Pragmatic Clinical Trial** | **[https://clinicaltrials.gov/ct2/show/NCT04713098](https://clinicaltrials.gov/ct2/show/NCT04713098)** | **Excluded** | **Review articles, conference papers** |
| **818** | **Nct et al** | **Validation of a Digital Twin Performing Strength Training** | **[https://clinicaltrials.gov/show/NCT04849923](https://clinicaltrials.gov/show/NCT04849923)** | **Excluded** | **Review articles, conference papers** |
| **819** | **Nct et al** | **ABM/P-15 Bone Graft vs Traditional Bone Graft in Adult Spinal Deformity Surgery** | **[https://clinicaltrials.gov/show/NCT05038527](https://clinicaltrials.gov/show/NCT05038527)** | **Excluded** | **Review articles, conference papers** |
| **820** | **Nct et al** | **Altered Back Geometry and Mobility Function After Backward Walking Training in Children With Cerebral Palsy** | **[https://clinicaltrials.gov/show/NCT04981964](https://clinicaltrials.gov/show/NCT04981964)** | **Excluded** | **Review articles, conference papers** |
| **821** | **Nct et al** | **Breast Cancer and Intrauterine Contraception** | **[https://clinicaltrials.gov/show/NCT05148910](https://clinicaltrials.gov/show/NCT05148910)** | **Excluded** | **Review articles, conference papers** |
| **822** | **Nct et al** | **Composite Flour and Its Antidiabetic Potential** | **[https://clinicaltrials.gov/ct2/show/NCT05163587](https://clinicaltrials.gov/ct2/show/NCT05163587)** | **Excluded** | **Review articles, conference papers** |
| **823** | **Nct et al** | **Dexamethasone and Robotic-assisted Hysterectomy** | **[https://clinicaltrials.gov/ct2/show/NCT04762381](https://clinicaltrials.gov/ct2/show/NCT04762381)** | **Excluded** | **Review articles, conference papers** |
| **824** | **Nct et al** | **Dual vs. Single-Antibiotic Impregnated Cement in Hemiarthroplasty for Femoral Neck Fracture** | **[https://clinicaltrials.gov/show/NCT05164081](https://clinicaltrials.gov/show/NCT05164081)** | **Excluded** | **Review articles, conference papers** |
| **825** | **Nct et al** | **EFFECT - EFFectiveness of ESPB (Erector Spinae Plane Block) in Laparoscopic Cοlectomies Trial** | **[https://clinicaltrials.gov/show/NCT04879004](https://clinicaltrials.gov/show/NCT04879004)** | **Excluded** | **Review articles, conference papers** |
| **826** | **Nct et al** | **Effect of Exercise in the Management of Peripheral Neuropathy** | **[https://clinicaltrials.gov/show/NCT04843410](https://clinicaltrials.gov/show/NCT04843410)** | **Excluded** | **Review articles, conference papers** |
| **827** | **Nct et al** | **Effect of Gluten-Free Dietary Education and Intraneural Facilitation® Therapy on Quality of Life in People With Diabetic Neuropathy** | **[https://clinicaltrials.gov/ct2/show/NCT05165368](https://clinicaltrials.gov/ct2/show/NCT05165368)** | **Excluded** | **Review articles, conference papers** |
| **828** | **Nct et al** | **Effect of Muscle Energy Technique on Clinical and Functional Levels in Chronic Obstructive Pulmonary** | **[https://clinicaltrials.gov/show/NCT04874571](https://clinicaltrials.gov/show/NCT04874571)** | **Excluded** | **Review articles, conference papers** |
| **829** | **Nct et al** | **Effect of Radial Pressure Waves Therapy in the Treatment of Non-Specific Neck Pain (rPWT)** | **[https://clinicaltrials.gov/show/NCT04758065](https://clinicaltrials.gov/show/NCT04758065)** | **Excluded** | **Review articles, conference papers** |
| **830** | **Nct et al** | **Effects of Inositol Alone or Associated With Alpha-lipoic Acid in Polycystic Ovary Syndrome Treatment** | **[https://clinicaltrials.gov/show/NCT04881851](https://clinicaltrials.gov/show/NCT04881851)** | **Excluded** | **Review articles, conference papers** |
| **831** | **Nct et al** | **Effects of Inspiratory Muscle Training (IMT) on Balance Ability and Quality of Life of Diabetes Mellitus Patients** | **[https://clinicaltrials.gov/ct2/show/NCT04947163](https://clinicaltrials.gov/ct2/show/NCT04947163)** | **Excluded** | **Review articles, conference papers** |
| **832** | **Nct et al** | **Efficacy and Safety of Low-Carbohydrate Diet Combined With Probiotics for Weight Loss in Male Obese Patients** | **[https://clinicaltrials.gov/ct2/show/NCT04886778](https://clinicaltrials.gov/ct2/show/NCT04886778)** | **Excluded** | **Review articles, conference papers** |
| **833** | **Nct et al** | **Evaluation of Botox Treatment on Chronical Scrotal Pain** | **[https://clinicaltrials.gov/show/NCT05112081](https://clinicaltrials.gov/show/NCT05112081)** | **Excluded** | **Review articles, conference papers** |
| **834** | **Nct et al** | **Focus of Attention Effect on Sit to Stand Symmetry in Individuals Post Stroke** | **[https://clinicaltrials.gov/show/NCT05125172](https://clinicaltrials.gov/show/NCT05125172)** | **Excluded** | **Review articles, conference papers** |
| **835** | **Nct et al** | **Home Based or Traditional Class HIIT in Overweight Women** | **[https://clinicaltrials.gov/show/NCT04796532](https://clinicaltrials.gov/show/NCT04796532)** | **Excluded** | **Review articles, conference papers** |
| **836** | **Nct et al** | **Impact of the SGLT2 Inhibitor Empagliflozin on Urinary Supersaturations in Kidney Stone Formers** | **[https://clinicaltrials.gov/show/NCT04911660](https://clinicaltrials.gov/show/NCT04911660)** | **Excluded** | **Review articles, conference papers** |
| **837** | **Nct et al** | **Integrated Basic Science Within the Instructional Design of Pattern Recognition Training** | **[https://clinicaltrials.gov/show/NCT05087485](https://clinicaltrials.gov/show/NCT05087485)** | **Excluded** | **Review articles, conference papers** |
| **838** | **Nct et al** | **Integrative Neuromuscular Training in Adolescents and Children Treated for Cancer** | **[https://clinicaltrials.gov/show/NCT04706676](https://clinicaltrials.gov/show/NCT04706676)** | **Excluded** | **Review articles, conference papers** |
| **839** | **Nct et al** | **Ketogenic Diet in PCOS With Obesity and Insulin Resistance** | **[https://clinicaltrials.gov/show/NCT04801173](https://clinicaltrials.gov/show/NCT04801173)** | **Excluded** | **Review articles, conference papers** |
| **840** | **Nct et al** | **Lifestyle Medicine Strategies for Combating Sleepiness and Fatigue in Professional Drivers** | **[https://clinicaltrials.gov/show/NCT05096130](https://clinicaltrials.gov/show/NCT05096130)** | **Excluded** | **Review articles, conference papers** |
| **841** | **Nct et al** | **Muscle Function and Effects of Repetitive Task Training in Patients With Inflammatory Myopaties** | **[https://clinicaltrials.gov/show/NCT05027152](https://clinicaltrials.gov/show/NCT05027152)** | **Excluded** | **Review articles, conference papers** |
| **842** | **Nct et al** | **Nutrigenetic Intervention on Blood Lipid Markers and Body Composition of Adults With Overweight and Obesity** | **[https://clinicaltrials.gov/show/NCT05210023](https://clinicaltrials.gov/show/NCT05210023)** | **Excluded** | **Review articles, conference papers** |
| **843** | **Nct et al** | **Physiological Effects of Soccer Heading** | **[https://clinicaltrials.gov/show/NCT04810130](https://clinicaltrials.gov/show/NCT04810130)** | **Excluded** | **Review articles, conference papers** |
| **844** | **Nct et al** | **Program for the Comprehensive Neurocognitive Treatment of Excess Weight** | **[https://clinicaltrials.gov/show/NCT05158075](https://clinicaltrials.gov/show/NCT05158075)** | **Excluded** | **Review articles, conference papers** |
| **845** | **Nct et al** | **Relaxation Exercise in Patients With COVID-19** | **[https://clinicaltrials.gov/show/NCT04998708](https://clinicaltrials.gov/show/NCT04998708)** | **Excluded** | **Review articles, conference papers** |
| **846** | **Nct et al** | **Remote Monitoring to Improve Low Adherence in Non-invasive Ventilation** | **[https://clinicaltrials.gov/ct2/show/NCT04884165](https://clinicaltrials.gov/ct2/show/NCT04884165)** | **Excluded** | **Review articles, conference papers** |
| **847** | **Nct et al** | **Skin Temperature Changes When Using a Cryocompression Device** | **[https://clinicaltrials.gov/show/NCT05136482](https://clinicaltrials.gov/show/NCT05136482)** | **Excluded** | **Review articles, conference papers** |
| **848** | **Nct et al** | **Time-restricted Eating to Improve Metabolic Abnormalities in Polycystic Ovarian Syndrome** | **[https://clinical** | **Excluded** | **Review articles, conference papers** |
| **849** | **Nct et al** | **Virtual Reality: influence on Satisfaction, Pain, and Anxiety in Patients Undergoing Colposcopy** | **[https://clinicaltrials.gov/ct2/show/NCT04751799](https://clinicaltrials.gov/ct2/show/NCT04751799)** | **Excluded** | **Review articles, conference papers** |
| **850** | **Nct et al** | **Effects of Nigella Sativa Oil on Pain Intensity and Physical Functions in Patients With Knee Osteoarthritis** | **[https://clinicaltrials.gov/show/NCT05541185](https://clinicaltrials.gov/show/NCT05541185)** | **Excluded** | **Review articles, conference papers** |
| **851** | **Nct et al** | **Kinesio-Taping and Ankle Instability in Recreational Runners** | **[https://clinicaltrials.gov/show/NCT05709808](https://clinicaltrials.gov/show/NCT05709808)** | **Excluded** | **Review articles, conference papers** |
| **852** | **Nct et al** | **Assessing the Feasibility and Preliminary Impact of a mHealth App on Reducing STI Risk in Black MSM PrEP Users** | **[https://clinicaltrials.gov/ct2/show/NCT05395754](https://clinicaltrials.gov/ct2/show/NCT05395754)** | **Excluded** | **Review articles, conference papers** |
| **853** | **Nct et al** | **Clinical Comparison of Patellofemoral Pain Syndrome Outcomes After Blood Flow Restriction Therapy** | **[https://clinicaltrials.gov/show/NCT05617911](https://clinicaltrials.gov/show/NCT05617911)** | **Excluded** | **Review articles, conference papers** |
| **854** | **Nct et al** | **The Clinical Effect of Pregabalin on Neuropathic Pain in Central Sensitized Patients After Total Knee Arthroplasty** | **[https://clinicaltrials.gov/show/NCT05254652](https://clinicaltrials.gov/show/NCT05254652)** | **Excluded** | **Review articles, conference papers** |
| **855** | **Nct et al** | **The Clinical Effect of Pregabalin on Neuropathic Pain in Non Central Sensitized Patients After Total Knee Arthroplasty** | **[https://clinicaltrials.gov/show/NCT05322681](https://clinicaltrials.gov/show/NCT05322681)** | **Excluded** | **Review articles, conference papers** |
| **856** | **Nct et al** | **Closed Kinetic Chain Exercises for Balance and Gait Rehabilitation for People With MS** | **[https://clinicaltrials.gov/show/NCT05460299](https://clinicaltrials.gov/show/NCT05460299)** | **Excluded** | **Review articles, conference papers** |
| **857** | **Nct et al** | **A Comparison of Impingement Free Range of Motion With CT Scan After Manual and Robotic Total Hip Replacement** | **[https://clinicaltrials.gov/show/NCT05507073](https://clinicaltrials.gov/show/NCT05507073)** | **Excluded** | **Review articles, conference papers** |
| **858** | **Nct et al** | **Comparison of the Effect of Two Anaesthesia Methods in Preventing Perioperative Myocardial Infarcation in Patients With Cardiac Risk Undergoing Total Knee Arthroplasty** | **[https://clinicaltrials.gov/show/NCT05340946](https://clinicaltrials.gov/show/NCT05340946)** | **Excluded** | **Review articles, conference papers** |
| **859** | **Nct et al** | **Core Stabilization and PNF Exercises on Cor Muscle Activation in Obese Children** | **[https://clinicaltrials.gov/show/NCT05571085](https://clinicaltrials.gov/show/NCT05571085)** | **Excluded** | **Review articles, conference papers** |
| **860** | **Nct et al** | **Diaphragmatic Exercises and Fascial Release Techniques on the Treatment of Lower Extremity Lymphedema** | **[https://clinicaltrials.gov/show/NCT05483569](https://clinicaltrials.gov/show/NCT05483569)** | **Excluded** | **Review articles, conference papers** |
| **861** | **Nct et al** | **Different Cryocompression Devices and Skin Temperature of the Knee** | **[https://clinicaltrials.gov/ct2/show/NCT05355116](https://clinicaltrials.gov/ct2/show/NCT05355116)** | **Excluded** | **Review articles, conference papers** |
| **862** | **Nct et al** | **Education Time Influence on Exercise-Induced Hypoalgesia** | **[https://clinicaltrials.gov/show/NCT05658224](https://clinicaltrials.gov/show/NCT05658224)** | **Excluded** | **Review articles, conference papers** |
| **863** | **Nct et al** | **The Effect of an Acute Bout of Exercise on Pain Sensitivity and Clinical Pain in Adults With Knee Osteoarthritis** | **[https://clinicaltrials.gov/ct2/show/NCT05605444](https://clinicaltrials.gov/ct2/show/NCT05605444)** | **Excluded** | **Review articles, conference papers** |
| **864** | **Nct et al** | **The Effect of Core Exercises on Pelvic Dysfunction, Sexual Dysfunction, Pain, Sleep Quality and Quality of Life in Women With Fibromyalgia** | **[https://clinicaltrials.gov/ct2/show/NCT06038214](https://clinicaltrials.gov/ct2/show/NCT06038214)** | **Excluded** | **Review articles, conference papers** |
| **865** | **Nct et al** | **Effect of Functional Strength Training of Hip Abductors in Runners With Medial Tibial Stress Syndrome** | **[https://clinicaltrials.gov/ct2/show/NCT05637476](https://clinicaltrials.gov/ct2/show/NCT05637476)** | **Excluded** | **Review articles, conference papers** |
| **866** | **Nct et al** | **Effect of Ultrasound-guided H-FICB in Patients Undergoing Arthroscopic Knee Surgery** | **[https://clinicaltrials.gov/show/NCT05325827](https://clinicaltrials.gov/show/NCT05325827)** | **Excluded** | **Review articles, conference papers** |
| **867** | **Nct et al** | **The Effects of Non-Immersive Virtual Reality Exercises on Muscle Excitability in Knee Osteoarthritis** | **[[https://clinicaltrials.gov/show/NCT05595317](https](https://clinicaltrials.gov/show/NCT05595317](https" \o "https://clinicaltrials.gov/show/NCT05595317](https)** | **Excluded** | **Review articles, conference papers** |
| **868** | **Nct et al** | **Effects of Telerehabilitative Aerobic and Relaxation Exercises in Patients With Type 2 Diabetes With and Without COVID-19** | **[https://clinicaltrials.gov/show/NCT05411458](https://clinicaltrials.gov/show/NCT05411458)** | **Excluded** | **Review articles, conference papers** |
| **869** | **Nct et al** | **Efficacy of Mesotherapy Added to Intra-articular Platelet-rich Plasma (PRP) in Patients With Knee Osteoarthritis** | **[https://clinicaltrials.gov/show/NCT05329116](https://clinicaltrials.gov/show/NCT05329116)** | **Excluded** | **Review articles, conference papers** |
| **870** | **Nct et al** | **FEA, Biomechanical and Clinical Study of R.O. Peritrochanteric Fractures With PFLP vs Cephalomedullary Nail** | **[https://clinicaltrials.gov/show/NCT05286905](https://clinicaltrials.gov/show/NCT05286905)** | **Excluded** | **Review articles, conference papers** |
| **871** | **Nct et al** | **High Intensity Interval Gait Training in Multiple Sclerosis** | **[https://clinicaltrials.gov/show/NCT05529498](https://clinicaltrials.gov/show/NCT05529498)** | **Excluded** | **Review articles, conference papers** |
| **872** | **Nct et al** | **Influence of Kinesio Tapping on Quadriceps Muscle Peak Torque and Bioelectrical Activity After Meniscectomy** | **[https://clinicaltrials.gov/show/NCT05715177](https://clinicaltrials.gov/show/NCT05715177)** | **Excluded** | **Review articles, conference papers** |
| **873** | **Nct et al** | **Iron Absorption and Variations of Iron Status, Hepcidin, Inflammation and Sex Hormones During the Menstrual Cycle** | **[https://clinicaltrials.gov/show/NCT05580783](https://clinicaltrials.gov/show/NCT05580783)** | **Excluded** | **Review articles, conference papers** |
| **874** | **Nct et al** | **IVR in Motor Rehabilitation** | **[https://clinicaltrials.gov/ct2/show/NCT05364970](https://clinicaltrials.gov/ct2/show/NCT05364970)** | **Excluded** | **Review articles, conference papers** |
| **875** | **Nct et al** | **Manual Ankle Rocking Training on Postural Control and Foot Function in Children With Down Syndrome** | **[https://clinicaltrials.gov/show/NCT05392309](https://clinicaltrials.gov/show/NCT05392309)** | **Excluded** | **Review articles, conference papers** |
| **876** | **Nct et al** | **Mindfulness and Hip Preservation Surgery** | **[https://clinicaltrials.gov/ct2/show/NCT05335421](https://clinicaltrials.gov/ct2/show/NCT05335421)** | **Excluded** | **Review articles, conference papers** |
| **877** | **Nct et al** | **Optimization of the Role of Action Observation in the Post-operative Rehabilitation of the Total Knee Prosthesis: (LOARAL 2)** | **[https://clinicaltrials.gov/ct2/show/NCT05297539](https://clinicaltrials.gov/ct2/show/NCT05297539)** | **Excluded** | **Review articles, conference papers** |
| **878** | **Nct et al** | **Optimizing Graft Selection for ACL Reconstruction** | **[https://clinicaltrials.gov/show/NCT05342441](https://clinicaltrials.gov/show/NCT05342441)** | **Excluded** | **Review articles, conference papers** |
| **879** | **Nct et al** | **Percutaneous Auricular Neuromodulation for Postoperative Analgesia** | **[https://clinicaltrials.gov/ct2/show/NCT05521516](https://clinicaltrials.gov/ct2/show/NCT05521516)** | **Excluded** | **Review articles, conference papers** |
| **880** | **Nct et al** | **PRP in ACLR to Prevent PTOA** | **[https://clinicaltrials.gov/ct2/show/NCT05412381](https://clinicaltrials.gov/ct2/show/NCT05412381)** | **Excluded** | **Review articles, conference papers** |
| **881** | **Nct et al** | **Randomised Total Knee Arthroplasty With Attune S+ or NexGen CR** | **[https://clinicaltrials.gov/show/NCT05464641](https://clinicaltrials.gov/show/NCT05464641)** | **Excluded** | **Review articles, conference papers** |
| **882** | **Nct et al** | **Robotic Assisted Rehabilitation for Balance and Gait in Orthopedic Patients** | **[https://clinicaltrials.gov/ct2/show/NCT05459584](https://clinicaltrials.gov/ct2/show/NCT05459584)** | **Excluded** | **Review articles, conference papers** |
| **883** | **Nct et al** | **Ultrasound-guided H-FICB for Arthroscopic Knee Surgery: What is the Optimal Dose of Dexmedetomidine?** | **[https://clinicaltrials.gov/show/NCT05533970](https://clinicaltrials.gov/show/NCT05533970)** | **Excluded** | **Review articles, conference papers** |
| **884** | **Nct et al** | **Vapocoolant Spray Application During Intraarticular Knee Injection** | **[https://clinicaltrials.gov/show/NCT05581433](https://clinicaltrials.gov/show/NCT05581433)** | **Excluded** | **Review articles, conference papers** |
| **885** | **Nct et al** | **BIS-guided Fluid Management in HD Patients** | **[https://clinicaltrials.gov/show/NCT05272800](https://clinicaltrials.gov/show/NCT05272800)** | **Excluded** | **Review articles, conference papers** |
| **886** | **Nct et al** | **Blood Flow Restriction After Anterior Cruciate Ligament Reconstruction (ACLR)** | **[https://clinicaltrials.gov/ct2/show/NCT05500872](https://clinicaltrials.gov/ct2/show/NCT05500872)** | **Excluded** | **Review articles, conference papers** |
| **887** | **Nct et al** | **Caudal Epidural Steroid and Trigger Point Injection** | **[https://clinicaltrials.gov/show/NCT05792111](https://clinicaltrials.gov/show/NCT05792111)** | **Excluded** | **Review articles, conference papers** |
| **888** | **Nct et al** | **Cherries Role in Gut Microbiota-liver-brain Function** | **[https://clinicaltrials.gov/show/NCT05586386](https://clinicaltrials.gov/show/NCT05586386)** | **Excluded** | **Review articles, conference papers** |
| **889** | **Nct et al** | **Chronic Exertional Compartment Syndrome (CECS) Treated With Abobotulinumtoxin A** | **[https://clinicaltrials.gov/ct2/show/NCT05466539](https://clinicaltrials.gov/ct2/show/NCT05466539)** | **Excluded** | **Review articles, conference papers** |
| **890** | **Nct et al** | **A Comparison Between the Effectiveness of Vestibular and Dual Task on Balance in Diplegic Children** | **[https://clinicaltrials.gov/show/NCT05692336](https://clinicaltrials.gov/show/NCT05692336)** | **Excluded** | **Review articles, conference papers** |
| **891** | **Nct et al** | **Comparison of the Effects of Core Stabilization and Resistance Exercises in Latin Dancers** | **[https://clinicaltrials.gov/show/NCT05268536](https://clinicaltrials.gov/show/NCT05268536)** | **Excluded** | **Review articles, conference papers** |
| **892** | **Nct et al** | **Comparison of Zero- and Two-centimeter Distance From Sapheno-femoral Junction in Laser Ablation of Varicose Vein** | **[https://clinicaltrials.gov/show/NCT05707169](https://clinicaltrials.gov/show/NCT05707169)** | **Excluded** | **Review articles, conference papers** |
| **893** | **Nct et al** | **Conservative Versus Intramedullary Nailing for Pediatric Tibial Shaft Fractures** | **[https://clinicaltrials.gov/show/NCT05501496](https://clinicaltrials.gov/show/NCT05501496)** | **Excluded** | **Review articles, conference papers** |
| **894** | **Nct et al** | **Core Strength Training on Anaerobic Power And Core Strength in Basketball Players** | **[https://clinicaltrials.gov/show/NCT05568771](https://clinicaltrials.gov/show/NCT05568771)** | **Excluded** | **Review articles, conference papers** |
| **895** | **Nct et al** | **Dietary Protein Quality for Skeletal Muscle Anabolism in Older Adults** | **[https://clinicaltrials.gov/ct2/show/NCT05574205](https://clinicaltrials.gov/ct2/show/NCT05574205)** | **Excluded** | **Review articles, conference papers** |
| **896** | **Nct et al** | **Early Functional Outcomes in Unisplaced Neck of Femur Fracture Treated With Partially Threaded and Fully Threaded Cannulated Screw Fixation in Patients of Age 60 and Above** | **[https://clinicaltrials.gov/show/NCT05587660](https://clinicaltrials.gov/show/NCT05587660)** | **Excluded** | **Review articles, conference papers** |
| **897** | **Nct et al** | **Effect of Focal Vibration Within a Multicomponent Exercise Program for Older Women With Osteoporosis a Single-blind Clinical Trial** | **[https://clinicaltrials.gov/show/NCT05538377](https://clinicaltrials.gov/show/NCT05538377)** | **Excluded** | **Review articles, conference papers** |
| **898** | **Nct et al** | **The Effect of Foot Bath on Postoperative Sleep Quality in Patients Undergoing Transurethral Resection** | **[https://clinicaltrials.gov/show/NCT05546684](https://clinicaltrials.gov/show/NCT05546684)** | **Excluded** | **Review articles, conference papers** |
| **899** | **Nct et al** | **The Effect of Non-invasive Bladder Stimulation Technique on Collecting Urine Specimen in Infants Under One Year Old** | **[https://clinicaltrials.gov/show/NCT05394454](https://clinicaltrials.gov/show/NCT05394454)** | **Excluded** | **Review articles, conference papers** |
| **900** | **Nct et al** | **Effect of the Nintendo Ring Fit Adventure Exergame on Running Completion Time** | **[https://clinicaltrials.gov/show/NCT05227040](https://clinicaltrials.gov/show/NCT05227040)** | **Excluded** | **Review articles, conference papers** |
| **901** | **Nct et al** | **Effectiveness of Vitamin C Supplementation in Treatment of Rickets** | **[https://clinicaltrials.gov/show/NCT05310760](https://clinicaltrials.gov/show/NCT05310760)** | **Excluded** | **Review articles, conference papers** |
| **902** | **Nct et al** | **Effects of GPR on Stress and Sleep Quality in Health Sciences** | **[https://clinicaltrials.gov/show/NCT05488015](https://clinicaltrials.gov/show/NCT05488015)** | **Excluded** | **Review articles, conference papers** |
| **903** | **Nct et al** | **Effects of Magnesium Oil Application on the Muscle Contractile Properties** | **[https://clinicaltrials.gov/ct2/show/NCT05339308](https://clinicaltrials.gov/ct2/show/NCT05339308)** | **Excluded** | **Review articles, conference papers** |
| **904** | **Nct et al** | **EFFICACY OF A STRETCHING PROTOCOL FOR LATERAL EPICONDYLITIS** | **[https://clinicaltrials.gov/show/NCT05238090](https://clinicaltrials.gov/show/NCT05238090)** | **Excluded** | **Review articles, conference papers** |
| **905** | **Nct et al** | **Efficacy of Selected Exercises on Bone Mineral Density in Post-burn Patients** | **[https://clinicaltrials.gov/show/NCT05276843](https://clinicaltrials.gov/show/NCT05276843)** | **Excluded** | **Review articles, conference papers** |
| **906** | **Nct et al** | **Fish Protein Supplementation and Sarcopenia Outcomes in the Community** | **[https://clinicaltrials.gov/show/NCT05356559](https://clinicaltrials.gov/show/NCT05356559)** | **Excluded** | **Review articles, conference papers** |
| **907** | **Nct et al** | **Immediate Versus Late Weight Bearing After Tibial Plateau Fractures Internal Fixation** | **[https://clinicaltrials.gov/ct2/show/NCT05502679](https://clinicaltrials.gov/ct2/show/NCT05502679)** | **Excluded** | **Review articles, conference papers** |
| **908** | **Nct et al** | **Integrated Exercise Approach Strength Postural Stability Menstrual Cycle Biomarkers Eumenorrheic Females** | **[https://clinicaltrials.gov/ct2/show/NCT05460741](https://clinicaltrials.gov/ct2/show/NCT05460741)** | **Excluded** | **Review articles, conference papers** |
| **909** | **Nct et al** | **Measuring Sleep Quality With Puki in Hemodialysis Patients Performing Progressive Muscle Relaxant Exercises** | **[https://clinicaltrials.gov/show/NCT05604833](https://clinicaltrials.gov/show/NCT05604833)** | **Excluded** | **Review articles, conference papers** |
| **910** | **Nct et al** | **Mindfulness and ACL Surgery** | **[https://clinicaltrials.gov/ct2/show/NCT05542563](https://clinicaltrials.gov/ct2/show/NCT05542563)** | **Excluded** | **Review articles, conference papers** |
| **911** | **Nct et al** | **Oral Health in Prison: a Study on Improving Prisoners' Oral Health** | **[https://clinicaltrials.gov/ct2/show/NCT05695443](https://clinicaltrials.gov/ct2/show/NCT05695443)** | **Excluded** | **Review articles, conference papers** |
| **912** | **Nct et al** | **Pulsed Electromagnetic Fields for Postoperative Analgesia: a Randomized, Triple-Masked, Sham-Controlled Pilot Study** | **[https://clinicaltrials.gov/ct2/show/NCT05399355](https://clinicaltrials.gov/ct2/show/NCT05399355)** | **Excluded** | **Review articles, conference papers** |
| **913** | **Nct et al** | **The Ratio Of Femoral Vein Diameter To Femoral Artery Diameter With Pulse Pressure Variation As A Diagnostic Tool** | **[https://clinicaltrials.gov/show/NCT05588180](https://clinicaltrials** | **Excluded** | **Review articles, conference papers** |
| **914** | **Nct et al** | **Short-Term Endogenous Hydrogen Sulfide Upregulation For Vein Graft Disease** | **[https://clinicaltrials.gov/show/NCT05457881](https://clinicaltrials.gov/show/NCT05457881)** | **Excluded** | **Review articles, conference papers** |
| **915** | **Nct et al** | **Stimulation Sites and Fatigue Induced by Neuromuscular Electrical Stimulation in Healthy Individuals** | **[https://clinicaltrials.gov/ct2/show/NCT05605210](https://clinicaltrials.gov/ct2/show/NCT05605210)** | **Excluded** | **Review articles, conference papers** |
| **916** | **Nct et al** | **Thoracic Neuromodulation for Diabetic Gastroparesis** | **[https://clinicaltrials.gov/show/NCT05273788](https://clinicaltrials.gov/show/NCT05273788)** | **Excluded** | **Review articles, conference papers** |
| **917** | **Nct et al** | **Web-based Interventions on Nonalcoholic Fatty Liver Disease (NAFLD) in Obese Children** | **[https://clinicaltrials.gov/show/NCT05527938](https://clinicaltrials.gov/show/NCT05527938)** | **Excluded** | **Review articles, conference papers** |
| **918** | **Nct et al** | **Adjuvant Treatment in Elderly Patients With Periodontitis Through the Administration of a Supplement Rich in Oleuropein From the Olive Leaf** | **[https://clinicaltrials.gov/show/NCT05482373](https://clinicaltrials.gov/show/NCT05482373)** | **Excluded** | **Review articles, conference papers** |
| **919** | **Nct et al** | **Bioavailability of Oleanolic Acid Formulated as Functional Olive Oil** | **[https://clinicaltrials.gov/show/NCT05529953](https://clinicaltrials.gov/show/NCT05529953)** | **Excluded** | **Review articles, conference papers** |
| **920** | **Nct et al** | **Buck Institute Ketone Ester RCT** | **[https://clinicaltrials.gov/ct2/show/NCT05585762](https://clinicaltrials.gov/ct2/show/NCT05585762)** | **Excluded** | **Review articles, conference papers** |
| **921** | **Nct et al** | **Chewing, Swallowing and Orofacial Motricity in Severe Obese** | **[https://clinicaltrials.gov/show/NCT05516693](https://clinicaltrials.gov/show/NCT05516693)** | **Excluded** | **Review articles, conference papers** |
| **922** | **Nct et al** | **Chronic Thermogenic Dietary Supplement Consumption** | **[https://clinicaltrials.gov/show/NCT05619809](https://clinicaltrials.gov/show/NCT05619809)** | **Excluded** | **Review articles, conference papers** |
| **923** | **Nct et al** | **Comparison of Coracoclavicular Fixation With Versus Without Acromioclavicular Stabilization for Repair of Acute Acromioclavicular Joint Dislocations: a Randomized Controlled Clinical Trial** | **[https://clinicaltrials.gov/show/NCT05501509](https://clinicaltrials.gov/show/NCT05501509)** | **Excluded** | **Review articles, conference papers** |
| **924** | **Nct et al** | **Effect of Pressure on Skin Temperature When Using a Cryocompression Device** | **[https://clinicaltrials.gov/ct2/show/NCT05454982](https://clinicaltrials.gov/ct2/show/NCT05454982)** | **Excluded** | **Review articles, conference papers** |
| **925** | **Nct et al** | **Effects of a Multimodal Exercise Program for Children With ASD** | **[https://clinicaltrials.gov/show/NCT05509231](https://clinicaltrials.gov/show/NCT05509231)** | **Excluded** | **Review articles, conference papers** |
| **926** | **Nct et al** | **An Examination of the Effect on Various Factors of Bed Baths Applied by Two Different Methods in Intensive Care Patients** | **[https://clinicaltrials.gov/show/NCT05310825](https://clinicaltrials.gov/show/NCT05310825)** | **Excluded** | **Review articles, conference papers** |
| **927** | **Nct et al** | **Implementation of an App-based Walking Aid Skills Training Program** | **[https://clinicaltrials.gov/show/NCT05347875](https://clinicaltrials.gov/show/NCT05347875)** | **Excluded** | **Review articles, conference papers** |
| **928** | **Nct et al** | **Improvement of Fatigue in Unstable Shoulder Through a Therapeutic Exercise Program in Physiotherapy** | **[https://clinicaltrials.gov/ct2/show/NCT05443295](https://clinicaltrials.gov/ct2/show/NCT05443295)** | **Excluded** | **Review articles, conference papers** |
| **929** | **Nct et al** | **The Inorganic Nitrate and eXercise Performance in Heart Failure (iNIX-HF)** | **[https://clinicaltrials.gov/ct2/show/NCT05562167](https://clinicaltrials.gov/ct2/show/NCT05562167)** | **Excluded** | **Review articles, conference papers** |
| **930** | **Nct et al** | **Multimodal Exercises on Sprint Performance of Rugby Players** | **[https://clinicaltrials.gov/ct2/show/NCT05485506](https://clinicaltrials.gov/ct2/show/NCT05485506)** | **Excluded** | **Review articles, conference papers** |
| **931** | **Nct et al** | **Pilot: combining HIIT and n-3 PUFA Supplementation to Reduce Inflammation and Improve Metabolic Health (HIIT&PUFA)** | **[https://clinicaltrials.gov/show/NCT05297383](https://clinicaltrials.gov/show/NCT05297383)** | **Excluded** | **Review articles, conference papers** |
| **932** | **Nct et al** | **Trial to Compare BCG-Bulgaria and BCG-Denmark** | **[https://clinicaltrials.gov/ct2/show/NCT05397678](https://clinicaltrials.gov/ct2/show/NCT05397678)** | **Excluded** | **Review articles, conference papers** |
| **933** | **Nct et al** | **ZOE METHOD Study: comparing Personalized vs. Generalized Nutrition Guidelines** | **[https://clinicaltrials.gov/show/NCT05273268](https://clinicaltrials.gov/show/NCT05273268)** | **Excluded** | **Review articles, conference papers** |
| **934** | **Nct et al** | **Black Seed Oil in Treatment of Osteoarthritis** | **[https://clinicaltrials.gov/ct2/show/NCT06029868](https://clinicaltrials.gov/ct2/show/NCT06029868)** | **Excluded** | **Review articles, conference papers** |
| **935** | **Nct et al** | **Cemented Versus Cementless Unicompartmental Knee Arthroplasty** | **[https://clinicaltrials.gov/ct2/show/NCT05935878](https://clinicaltrials.gov/ct2/show/NCT05935878)** | **Excluded** | **Review articles, conference papers** |
| **936** | **Nct et al** | **Comparative Effectiveness of Individual Versus Group-Level Interventions to Reduce Human Immunodeficiency Virus (HIV)/ Sexually Transmitted Infections (STI) Incidence** | **[https://clinicaltrials.gov/ct2/show/NCT06022809](https://clinicaltrials.gov/ct2/show/NCT06022809)** | **Excluded** | **Review articles, conference papers** |
| **937** | **Nct et al** | **Comparison of Cruciate Retaining and Posterior Stabilized Total Knee Arthroplasty** | **[https://clinicaltrials.gov/ct2/show/NCT06124651](https://clinicaltrials.gov/ct2/show/NCT06124651)** | **Excluded** | **Review articles, conference papers** |
| **938** | **Nct et al** | **Diagnostic Ultrasonography in Physiotherapy** | **[https://clinicaltrials.gov/show/NCT05916300](https://clinicaltrials.gov/show/NCT05916300)** | **Excluded** | **Review articles, conference papers** |
| **939** | **Nct et al** | **The Effect of Structured Education on Sexual Function and Sexual Quality of Life** | **[https://clinicaltrials.gov/ct2/show/NCT05808959](https://clinicaltrials.gov/ct2/show/NCT05808959)** | **Excluded** | **Review articles, conference papers** |
| **940** | **Nct et al** | **Effects of TENS During the Performance of a Therapeutic Exercise Protocol in Individuals With Knee Osteoarthritis** | **[https://clinicaltrials.gov/ct2/show/NCT06184451](https://clinicaltrials.gov/ct2/show/NCT06184451)** | **Excluded** | **Review articles, conference papers** |
| **941** | **Nct et al** | **Erector Spinae Plane Block and Ankle and Foot Surgery** | **[https://clinicaltrials.gov/show/NCT05708742](https://clinicaltrials.gov/show/NCT05708742)** | **Excluded** | **Review articles, conference papers** |
| **942** | **Nct et al** | **HIP Fracture Oral thromboPROphylaxis (Hip PRO Pilot)** | **[https://clinicaltrials.gov/show/NCT05775965](https://clinicaltrials.gov/show/NCT05775965)** | **Excluded** | **Review articles, conference papers** |
| **943** | **Nct et al** | **Improving Wellbeing in Older Adults: The Impact of an Active Aging Education Program in Pakistan** | **[https://clinicaltrials.gov/show/NCT05918289](https://clinicaltrials.gov/show/NCT05918289)** | **Excluded** | **Review articles, conference papers** |
| **944** | **Nct et al** | **Medial Wedge Insoles to Improve Gait in Persons After Total Hip Replacement** | **[https://clinicaltrials.gov/show/NCT05736016](https://clinicaltrials.gov/show/NCT05736016)** | **Excluded** | **Review articles, conference papers** |
| **945** | **Nct et al** | **Open Versus Close Chain Exercise Effects With Lifestyle Modification and Education in Knee Osteoarthritis** | **[https://clinicaltrials.gov/ct2/show/NCT05877703](https://clinicaltrials.gov/ct2/show/NCT05877703)** | **Excluded** | **Review articles, conference papers** |
| **946** | **Nct et al** | **Opioid-Free Pain Treatment in Trauma Patients** | **[[https://clinicaltrials.gov/ct2/show/NCT06078371](https://clinicaltrials.gov/ct2/show/NCT06078371)** | **Excluded** | **Review articles, conference papers** |
| **947** | **Nct et al** | **Partial Synovectomy in Articular Side of Quadriceps Tendon Verified to Reduce Crepitus in Retrospective Study** | **[https://clinicaltrials.gov/ct2/show/NCT06129214](https://clinicaltrials.gov/ct2/show/NCT06129214)** | **Excluded** | **Review articles, conference papers** |
| **948** | **Nct et al** | **Stopping OsteoARthritis After an ACL Tear** | **[https://clinicaltrials.gov/ct2/show/NCT06195423](https://clinicaltrials.gov/ct2/show/NCT06195423)** | **Excluded** | **Review articles, conference papers** |
| **949** | **Nct et al** | **Treadmill Perturbation Training for Fall Prevention After Total Knee Replacement** | **[https://clinicaltrials.gov/ct2/show/NCT05736666](https://clinicaltrials.gov/ct2/show/NCT05736666)** | **Excluded** | **Review articles, conference papers** |
| **950** | **Nct et al** | **WALANT and Spinal Anesthesia Comparison of Clinical Result in Ankle Fractures** | **[https://clinicaltrials.gov/ct2/show/NCT06077344](https://clinicaltrials.gov/ct2/show/NCT06077344)** | **Excluded** | **Review articles, conference papers** |
| **951** | **Nct et al** | **Baker Cyst Dimensions and Intermittent Vacuum Therapy in Knee Osteoarthritis (BCIVT)** | **[https://clinicaltrials.gov/ct2/show/NCT06079684](https://clinicaltrials.gov/ct2/show/NCT06079684)** | **Excluded** | **Review articles, conference papers** |
| **952** | **Nct et al** | **Balneotherapy for Patients With Post-acute Coronavirus Disease (COVID) Syndrome** | **[https://clinicaltrials.gov/show/NCT05765591](https://clinicaltrials.gov/show/NCT05765591)** | **Excluded** | **Review articles, conference papers** |
| **953** | **Nct et al** | **The Clinical Impact of Diet on Medical Conditions Treated Within Bundled Payment Models: a Pilot Study** | **[https://clinicaltrials.gov/ct2/show/NCT05735873](https://clinicaltrials.gov/ct2/show/NCT05735873)** | **Excluded** | **Review articles, conference papers** |
| **954** | **Nct et al** | **Comparing The Effectiveness Of Pericapsular Nerve Group (PENG) Block Versus Supra-Inguinal Fascia Iliaca Compartment Block (S-FICB) In Reducing Positional Pain During Neuraxial Anaesthesia In Hip Fractures Patients** | **[https://clinicaltrials.gov/show/NCT05721924](https://clinicaltrials.gov/show/NCT05721924)** | **Excluded** | **Review articles, conference papers** |
| **955** | **Nct et al** | **Corticosteroid vs Platelet-Rich Plasma Intra-articular Injections in the Treatment of Knee Osteoarthritis** | **[https://clinicaltrials.gov/ct2/show/NCT06032039](https://clinicaltrials.gov/ct2/show/NCT06032039)** | **Excluded** | **Review articles, conference papers** |
| **956** | **Nct et al** | **Cryoneurolysis for Acute Postoperative Pain Following Total Knee Arthroplasty** | **[https://clinicaltrials.gov/ct2/show/NCT06088602](https://clinicaltrials.gov/ct2/show/NCT06088602)** | **Excluded** | **Review articles, conference papers** |
| **957** | **Nct et al** | **Effect of Action Observation and Motor Imagery on Arthrogenic Muscle Inhibition of the Quadriceps in Patients With End-stage Knee Gonarthrosis** | **[https://clinicaltrials.gov/ct2/show/NCT06000345](https://clinicaltrials.gov/ct2/show/NCT06000345)** | **Excluded** | **Review articles, conference papers** |
| **958** | **Nct et al** | **Effect of Femoral Quadriceps Muscle Length on Fatigue Induced by Neuromuscular Electrical Stimulation** | **[https://clinicaltrials.gov/show/NCT05905406](https://clinicaltrials.gov/show/NCT05905406)** | **Excluded** | **Review articles, conference papers** |
| **959** | **Nct et al** | **The Effect of Lower Extremity ROM Exercises on Hypotension, Fatigue, and Hemodialysis Comfort in Individuals** | **[https://clinicaltrials.gov/ct2/show/NCT06344273](https://clinicaltrials.gov/ct2/show/NCT06344273)** | **Excluded** | **Review articles, conference papers** |
| **960** | **Nct et al** | **The Effect of Reiki on Sexual Function and Sexual Self-Confidence** | **[https://clinicaltrials.gov/show/NCT05922787](https://clinicaltrials.gov/show/NCT05922787)** | **Excluded** | **Review articles, conference papers** |
| **961** | **Nct et al** | **The Effect of Sexual Counseling With the PLISSIT Model on Sexual Function and Sexual Quality of Life in Women With Multiple Sclerosis: a Randomized Controlled Study** | **[https://clinicaltrials.gov/ct2/show/NCT06004518](https://clinicaltrials.gov/ct2/show/NCT06004518)** | **Excluded** | **Review articles, conference papers** |
| **962** | **Nct et al** | **Effect of Shortwave Diathermy Versus Ultrasound Waves on Increasing ROM and Decreasing Pain After Extensor Tendon Reconstruction** | **[https://clinicaltrials.gov/ct2/show/NCT06113250](https://clinicaltrials.gov/ct2/show/NCT06113250)** | **Excluded** | **Review articles, conference papers** |
| **963** | **Nct et al** | **Effectiveness of a Multi-component Physical Exercise Online** | **[https://clinicaltrials.gov/show/NCT05895760](https://clinicaltrials.gov/show/NCT05895760)** | **Excluded** | **Review articles, conference papers** |
| **964** | **Nct et al** | **The Effectiveness of Client-Centered Intervention in Total Hip Arthroplasty** | **[https://clinicaltrials.gov/ct2/show/NCT06070220](https://clinicaltrials.gov/ct2/show/NCT06070220)** | **Excluded** | **Review articles, conference papers** |
| **965** | **Nct et al** | **Effects of Dextrose Prolotherapy in Patients With Knee Osteoarthritis** | **[https://clinicaltrials.gov/ct2/show/NCT06063356](https://clinicaltrials.gov/ct2/show/NCT06063356)** | **Excluded** | **Review articles, conference papers** |
| **966** | **Nct et al** | **Effects of Virtual Reality Stabilization Training in Patients With Low Back Pain** | **[[https://clinicaltrials.gov/ct2/show/NCT06030960](https](https://clinicaltrials.gov/ct2/show/NCT06030960](https" \o "https://clinicaltrials.gov/ct2/show/NCT06030960](https)** | **Excluded** | **Review articles, conference papers** |
| **967** | **Nct et al** | **The Efficacy of Intra-articular Triamcinolone Acetonide 5mg vs. 10 mg vs. 40 mg in Patients With Knee Osteoarthritis** | **[https://clinicaltrials.gov/ct2/show/NCT05806021](https://clinicaltrials.gov/ct2/show/NCT05806021)** | **Excluded** | **Review articles, conference papers** |
| **968** | **Nct et al** | **FOA on Sit to Stand in Individuals Post Stroke** | **[https://clinicaltrials.gov/show/NCT05874661](https://clinicaltrials.gov/show/NCT05874661)** | **Excluded** | **Review articles, conference papers** |
| **969** | **Nct et al** | **Grip on Knee Osteoarthritis; DIstraction Versus Arthroplasty** | **[https://clinicaltrials.gov/ct2/show/NCT06113549](https://clinicaltrials.gov/ct2/show/NCT06113549)** | **Excluded** | **Review articles, conference papers** |
| **970** | **Nct et al** | **HDP vs NS Intra-articular Injection Among KOA With Obese Patient** | **[https://clinicaltrials.gov/ct2/show/NCT05966948](https://clinicaltrials.gov/ct2/show/NCT05966948)** | **Excluded** | **Review articles, conference papers** |
| **971** | **Nct et al** | **Immediate Effects of PNF With Lower Leg Kinesio Taping in Chronic Stroke** | **[https://clinicaltrials.gov/ct2/show/NCT05857657](https://clinicaltrials.gov/ct2/show/NCT05857657)** | **Excluded** | **Review articles, conference papers** |
| **972** | **Nct et al** | **Increased Perioperative Communication Program in Knee Arthroplasty** | **[https://clinicaltrials.gov/ct2/show/NCT06130813](https://clinicaltrials.gov/ct2/show/NCT06130813)** | **Excluded** | **Review articles, conference papers** |
| **973** | **Nct et al** | **The Knee Care @Home Programme Following Anterior Ligament Reconstruction** | **[https://clinicaltrials.gov/show/NCT05828355](https://clinicaltrials.gov/show/NCT05828355)** | **Excluded** | **Review articles, conference papers** |
| **974** | **Nct et al** | **Knee Osteoarthritis Treatment With Platelet-rich Plasma** | **[https://clinicaltrials.gov/ct2/show/NCT05824806](https://clinicaltrials.gov/ct2/show/NCT05824806)** | **Excluded** | **Review articles, conference papers** |
| **975** | **Nct et al** | **Low-intensity Aerobic Training Associated With Global Muscle Strengthening in Post-COVID-19** | **[https://clinicaltrials.gov/ct2/show/NCT06161740](https://clinicaltrials.gov/ct2/show/NCT06161740)** | **Excluded** | **Review articles, conference papers** |
| **976** | **Nct et al** | **Motivational Feedback Following Total or Unicompartmental Knee Arthroplasty** | **[https://clinicaltrials.gov/ct2/show/NCT06005623](https://clinicaltrials.gov/ct2/show/NCT06005623)** | **Excluded** | **Review articles, conference papers** |
| **977** | **Nct et al** | **Oxandrolone Multiligament Knee** | **[https://clinicaltrials.gov/ct2/show/NCT05893069](https://clinicaltrials.gov/ct2/show/NCT05893069)** | **Excluded** | **Review articles, conference papers** |
| **978** | **Nct et al** | **PENG Block Plus Local Infiltration Compared to PENG Block Alone in Hip Surgery** | **[https://clinicaltrials.gov/ct2/show/NCT05773365](https://clinicaltrials.gov/ct2/show/NCT05773365)** | **Excluded** | **Review articles, conference papers** |
| **979** | **Nct et al** | **Photobiomodulation Therapy to Reduce Pain and Inflammation in Patients With Gonarthrosis** | **[https://clinicaltrials.gov/ct2/show/NCT05975957](https://clinicaltrials.gov/ct2/show/NCT05975957)** | **Excluded** | **Review articles, conference papers** |
| **980** | **Nct et al** | **Repetitive Transcranial Magnetic Stimulation Versus Botulinum Injection on Spasticity on Children With Diplegic Cerebral Palsy** | **[https://clinicaltrials.gov/ct2/show/NCT06218316](https://clinicaltrials.gov/ct2/show/NCT06218316)** | **Excluded** | **Review articles, conference papers** |
| **981** | **Nct et al** | **Sexual Counseling's Impact on Hip Replacement Patients' Sexual Function and Quality of Life: a PLISSIT Model Study** | **[https://clinicaltrials.gov/ct2/show/NCT06017128](https://clinicaltrials.gov/ct2/show/NCT06017128)** | **Excluded** | **Review articles, conference papers** |
| **982** | **Nct et al** | **Structured Tailored Rehabilitation After Hip Fragility Fracture** | **[https://clinicaltrials.gov/ct2/show/NCT06014554](https://clinicaltrials.gov/ct2/show/NCT06014554)** | **Excluded** | **Review articles, conference papers** |
| **983** | **Nct et al** | **A Study to Assess Efficacy of Supporting Properties and Safety of ARTNEO in Patients With Knee Osteoarthritis** | **[https://clinicaltrials.gov/ct2/show/NCT06032442](https://clinicaltrials.gov/ct2/show/NCT06032442)** | **Excluded** | **Review articles, conference papers** |
| **984** | **Nct et al** | **Acute Effect of Static Stretching and Pilates Stretching on the Muscle Strength** | **[https://clinicaltrials.gov/ct2/show/NCT06151860](https://clinicaltrials.gov/ct2/show/NCT06151860)** | **Excluded** | **Review articles, conference papers** |
| **985** | **Nct et al** | **CAsting and REhabilitation Versus Supervised Neglect for Osteochondral Lesions of the Talus in the Pediatric Population** | **[https://clinicaltrials.gov/ct2/show/NCT06220539](https://clinicaltrials.gov/ct2/show/NCT06220539)** | **Excluded** | **Review articles, conference papers** |
| **986** | **Nct et al** | **Comparison of Intra-articular of 0.2% Ropivacaine vs. 0.75% Ropivacaine in Postoperative of Knee Arthroscopy** | **[https://clinicaltrials.gov/ct2/show/NCT05807945](https://clinicaltrials.gov/ct2/show/NCT05807945)** | **Excluded** | **Review articles, conference papers** |
| **987** | **Nct et al** | **Dry Needling for Provoked Vestibulodynia** | **[https://clinicaltrials.gov/ct2/show/NCT05797480](https://clinicaltrials.gov/ct2/show/NCT05797480)** | **Excluded** | **Review articles, conference papers** |
| **988** | **Nct et al** | **Effect of Goji Berry Consumption on Biochemical Parameters** | **[https://clinicaltrials.gov/ct2/show/NCT06190587](https://clinicaltrials.gov/ct2/show/NCT06190587)** | **Excluded** | **Review articles, conference papers** |
| **989** | **Nct et al** | **The Effect of Hot-Cold Application to the Bladder After Orthopedic Surgery on Postoperative Urinary Retention** | **[https://clinicaltrials.gov/ct2/show/NCT05969145](https://clinicaltrials.gov/ct2/show/NCT05969145)** | **Excluded** | **Review articles, conference papers** |
| **990** | **Nct et al** | **Effect of Kinesio Taping and Extracorporeal Shock Wave Therapy on Plantar Fasciitis** | **[https://clinicaltrials.gov/ct2/show/NCT06055933](https://clinicaltrials.gov/ct2/show/NCT06055933)** | **Excluded** | **Review articles, conference papers** |
| **991** | **Nct et al** | **The Effect of Motor Image Training on Balance Performance in Geriatric Individuals** | **[https://clinicaltrials.gov/ct2/show/NCT06056180](https://clinicaltrials.gov/ct2/show/NCT06056180)** | **Excluded** | **Review articles, conference papers** |
| **992** | **Nct et al** | **The Effect Of Osteopathic Manual Therapy On Arterial Circulation In Patients With Peripheral Arterial Disease** | **[https://clinicaltrials.gov/show/NCT05837442](https://clinicaltrials.gov/show/NCT05837442)** | **Excluded** | **Review articles, conference papers** |
| **993** | **Nct et al** | **Effect of Specifically Developed Exercise Intervention on Falls Among Older Adults in Nursing Homes** | **[https://clinicaltrials.gov/ct2/show/NCT05835297](https://clinicaltrials.gov/ct2/show/NCT05835297)** | **Excluded** | **Review articles, conference papers** |
| **994** | **Nct et al** | **The Effects of Environmental Distractions on SCAT6 Outcomes** | **[https://clinicaltrials.gov/ct2/show/NCT05886400](https://clinicaltrials.gov/ct2/show/NCT05886400)** | **Excluded** | **Review articles, conference papers** |
| **995** | **Nct et al** | **Effects of Neural Flossing and PNF on Lumbar Radiculopathy** | **[https://clinicaltrials.gov/show/NCT05922228](https://clinicaltrials.gov/show/NCT05922228)** | **Excluded** | **Review articles, conference papers** |
| **996** | **Nct et al** | **The Efficacy and Safety of Diosmin on Non-diabetic Patients With Non-alcoholic Steatohepatitis** | **[https://clinicaltrials.gov/ct2/show/NCT05942547](https://clinicaltrials.gov/ct2/show/NCT05942547)** | **Excluded** | **Review articles, conference papers** |
| **997** | **Nct et al** | **Efficacy of Pericapsular Nerve Group (PENG) Block for Hip Surgeries** | **[https://clinicaltrials.gov/ct2/show/NCT06144931](https://clinicaltrials.gov/ct2/show/NCT06144931)** | **Excluded** | **Review articles, conference papers** |
| **998** | **Nct et al** | **GENERAL vs. REGIONAL ANESTHESIA ON SLEEP QUALITY FOR HIP ARTROPLASTY PATIENTS** | **[https://clinicaltrials.gov/ct2/show/NCT06041711](https://clinicaltrials.gov/ct2/show/NCT06041711)** | **Excluded** | **Review articles, conference papers** |
| **999** | **Nct et al** | **Heat Therapy and Strength Training Effects in Individuals With Type 2 Diabetes** | **[https://clinicaltrials.gov/show/NCT05847075](https://clinicaltrials.gov/show/NCT05847075)** | **Excluded** | **Review articles, conference papers** |
| **1000** | **Nct et al** | **Home-based Training and Supplementation in DM1 Patients** | **[https://clinicaltrials.gov/show/NCT05848830](https://clinicaltrials.gov/show/NCT05848830)** | **Excluded** | **Review articles, conference papers** |
| **1001** | **Nct et al** | **The Impact of Dynamic Neuromuscular Stabilization on Pregnancy** | **[https://clinicaltrials.gov/ct2/show/NCT06239753](https://clinicaltrials.gov/ct2/show/NCT06239753)** | **Excluded** | **Review articles, conference papers** |
| **1002** | **Nct et al** | **Influence of Muscle Stretching and Neural Mobilizations on Lower Limbs Range of Motion in Asymptomatic Subjects** | **[https://clinicaltrials.gov/show/NCT05899244](https://clinicaltrials.gov/show/NCT05899244)** | **Excluded** | **Review articles, conference papers** |
| **1003** | **Nct et al** | **Investigation of the Effect of Pilates Exercises on Patellofemoral Pain** | **[https://clinicaltrials.gov/ct2/show/NCT05811637](https://clinicaltrials.gov/ct2/show/NCT05811637)** | **Excluded** | **Review articles, conference papers** |
| **1004** | **Nct et al** | **Investigation of the Effects of Vibration Therapy on Pain, Functionality, and Proprioception After ACL Injury** | **[https://clinicaltrials.gov/ct2/show/NCT06185231](https://clinicaltrials.gov/ct2/show/NCT06185231)** | **Excluded** | **Review articles, conference papers** |
| **1005** | **Nct et al** | **Low Concentration Local Anesthesia Fascia Iliaca Block for Total Hip Arthroplasty** | **[https://clinicaltrials.gov/ct2/show/NCT06102811](https://clinicaltrials.gov/ct2/show/NCT06102811)** | **Excluded** | **Review articles, conference papers** |
| **1006** | **Nct et al** | **Online Neuropilates Classes in Chronic Stroke Patients: a Pilot Randomised Feasibility Study** | **[https://clinicaltrials.gov/show/NCT05739422](https://clinicaltrials.gov/show/NCT05739422)** | **Excluded** | **Review articles, conference papers** |
| **1007** | **Nct et al** | **Optimal Frequency Used in Transcutaneous Electrical Nerve Stimulation (TENS) for Treating Pelvic Pain in Adults** | **[https://clinicaltrials.gov/ct2/show/NCT06019091](https://clinicaltrials.gov/ct2/show/NCT06019091)** | **Excluded** | **Review articles, conference papers** |
| **1008** | **Nct et al** | **PENG Block and Lateral Femoral Cutaneous Nerve Block For Hip Replacement Surgery** | **[https://clinicaltrials.gov/ct2/show/NCT06040879](https://clinicaltrials.gov/ct2/show/NCT06040879)** | **Excluded** | **Review articles, conference papers** |
| **1009** | **Nct et al** | **Probiotics and Insulin Resistance in Obese Asthmatics** | **[https://clinicaltrials.gov/ct2/show/NCT05949255](https://clinicaltrials.gov/ct2/show/NCT05949255)** | **Excluded** | **Review articles, conference papers** |
| **1010** | **Nct et al** | **Pulsed Shortwave Therapy for Postoperative Analgesia** | **[https://clinicaltrials.gov/ct2/show/NCT05796583](https://clinicaltrials.gov/ct2/show/NCT05796583)** | **Excluded** | **Review articles, conference papers** |
| **1011** | **Nct et al** | **Sinus Tarsi Implant as an Adjuvant Procedure to Medial Displacement Calcaneal Osteotomy in the Treatment of Mobile Adult Acquired Flatfoot Deformity** | **[https://clinicaltrials.gov/ct2/show/NCT06211504](https://clinicaltrials.gov/ct2/show/NCT06211504)** | **Excluded** | **Review articles, conference papers** |
| **1012** | **Nct et al** | **Telehealth Delivered Home-based Walking for Vets With Peripheral Artery Disease** | **[https://clinicaltrials.gov/ct2/show/NCT06033924](https://clinicaltrials.gov/ct2/show/NCT06033924)** | **Excluded** | **Review articles, conference papers** |
| **1013** | **Nct et al** | **Testing Implementation of Total Joint Replacement Rehabilitation Quality Indicator Toolkits** | **[https://clinicaltrials.gov/ct2/show/NCT06208553](https://clinicaltrials.gov/ct2/show/NCT06208553)** | **Excluded** | **Review articles, conference papers** |
| **1014** | **Nct et al** | **Treadmill With TENS on Functional Capacity & Muscle Oxygenation in PAD Patients** | **[https://clinicaltrials.gov/ct2/show/NCT06061211](https://clinicaltrials.gov/ct2/show/NCT06061211)** | **Excluded** | **Review articles, conference papers** |
| **1015** | **Nct et al** | **Video Based Games Exercise Training in Individuals With Cerebral Palsy** | **[https://clinicaltrials.gov/ct2/show/NCT06073743](https://clinicaltrials.gov/ct2/show/NCT06073743)** | **Excluded** | **Review articles, conference papers** |
| **1016** | **Nct et al.** | **Anthrax AV7909 Boost Evaluation Study** | **[https://clinicaltrials.gov/ct2/show/NCT05997264](https://clinicaltrials.gov/ct2/show/NCT05997264)** | **Excluded** | **Review articles, conference papers** |
| **1017** | **Nct et al.** | **Carpal Arch Space Augmentation (CASA) Clinical Trial** | **[https://clinicaltrials.gov/ct2/show/NCT06208709](https://clinicaltrials.gov/ct2/show/NCT06208709)** | **Excluded** | **Review articles, conference papers** |
| **1018** | **Nct et al.** | **Combining Physiotherapy Group Exercise With Acceptance and Commitment Therapy in Elderly With Chronic Low Back Pain** | **[https://clinicaltrials.gov/ct2/show/NCT06148896](https://clinicaltrials.gov/ct2/show/NCT06148896)** | **Excluded** | **Review articles, conference papers** |
| **1019** | **Nct et al.** | **Comparing Wound Complication Following TMA With Aid of Electrospun Fiber Matrix** | **[https://clinicaltrials.gov/ct2/show/NCT06063694](https://clinicaltrials.gov/ct2/show/NCT06063694)** | **Excluded** | **Review articles, conference papers** |
| **1020** | **Nct et al.** | **Dexmedetomidine and Kidney Transplantation** | **[https://clinicaltrials.gov/ct2/show/NCT05935293](https://clinicaltrials.gov/ct2/show/NCT05935293)** | **Excluded** | **Review articles, conference papers** |
| **1021** | **Nct et al.** | **Diosmin for Treatment of Delayed-onset Muscle Soreness (DOMS)** | **[https://clinicaltrials.gov/ct2/show/NCT06125002](https://clinicaltrials.gov/ct2/show/NCT06125002)** | **Excluded** | **Review articles, conference papers** |
| **1022** | **Nct et al.** | **The Effect of Foot Massage on Anxiety, Pain and Comfort Level** | **[https://clinicaltrials.gov/ct2/show/NCT05850897](https://clinicaltrials.gov/ct2/show/NCT05850897)** | **Excluded** | **Review articles, conference papers** |
| **1023** | **Nct et al.** | **Effect of Intradialytic Continuous Versus Interval Aerobic Exercises on Quality of Life in Patients With Chronic Renal Failure** | **[https://clinicaltrials.gov/ct2/show/NCT06173141](https://clinicaltrials.gov/ct2/show/NCT06173141)** | **Excluded** | **Review articles, conference papers** |
| **1024** | **Nct et al.** | **The Effect of Occupational Therapy-Based Instrumental Activities of Daily Living Intervention in People With Disabilities** | **[https://clinicaltrials.gov/show/NCT05790798](https://clinicaltrials.gov/show/NCT05790798)** | **Excluded** | **Review articles, conference papers** |
| **1025** | **Nct et al.** | **Effect of Reflective Blanket on Undesirable Perioperative Hypothermia** | **[https://clinicaltrials.gov/show/NCT05702320](https://clinicaltrials.gov/show/NCT05702320)** | **Excluded** | **Review articles, conference papers** |
| **1026** | **Nct et al.** | **Effect of Thrower's Ten Exercise Program on Shoulder Flexibility, Stability and Strength in Water Polo Players** | **[https://clinicaltrials.gov/ct2/show/NCT05945238](https://clinicaltrials.gov/ct2/show/NCT05945238)** | **Excluded** | **Review articles, conference papers** |
| **1027** | **Nct et al.** | **The Effect of Vibration Therapy and Mirror Therapy on Upper Limb Function in Patients With Stroke** | **[https://clinicaltrials.gov/ct2/show/NCT06126978](https://clinicaltrials.gov/ct2/show/NCT06126978)** | **Excluded** | **Review articles, conference papers** |
| **1028** | **Nct et al.** | **Effectiveness of the Adherence for Exercise Rehabilitation in Older People (AERO) Program in People With Osteoporosis** | **[https://clinicaltrials.gov/ct2/show/NCT06164847](https://clinicaltrials.gov/ct2/show/NCT06164847)** | **Excluded** | **Review articles, conference papers** |
| **1029** | **Nct et al.** | **Effects of Foot Rehabilitation And Minimalist Shoes on Pain, Strength, and Function in Adults With Plantar Fasciopathy** | **[https://clinicaltrials.gov/ct2/show/NCT06106958](https://clinicaltrials.gov/ct2/show/NCT06106958)** | **Excluded** | **Review articles, conference papers** |
| **1030** | **Nct et al.** | **Effects of Forward Head Posture Correction on Visual Acuity in Low Level Visually Impaired University Students** | **[https://clinicaltrials.gov/ct2/show/NCT06019806](https://clinicaltrials.gov/ct2/show/NCT06019806)** | **Excluded** | **Review articles, conference papers** |
| **1031** | **Nct et al.** | **The Effects of Nutritional Intervention on Health Parameters in Participants With Type 2 Diabetes Mellitus** | **[https://clinicaltrials.gov/ct2/show/NCT06235762](https://clinicaltrials.gov/ct2/show/NCT06235762)** | **Excluded** | **Review articles, conference papers** |
| **1032** | **Nct et al.** | **The Evaluation of Two Positive Body Image Micro-interventions for Children Aged 4-6 Years** | **[https://clinicaltrials.gov/ct2/show/NCT06146647](https://clinicaltrials.gov/ct2/show/NCT06146647)** | **Excluded** | **Review articles, conference papers** |
| **1033** | **Nct et al.** | **Immediate and 24-hour Effects of HyperVolt, Body Tempering, and Cupping Compared to Stretching on Hamstring Flexibility** | **[https://clinicaltrials.gov/show/NCT05793242](https://clinicaltrials.gov/show/NCT05793242)** | **Excluded** | **Review articles, conference papers** |
| **1034** | **Nct et al.** | **Impact of Virtual Reality on the Postoperative Balance of Adolescents With Idiopathic Scoliosis** | **[https://clinicaltrials.gov/ct2/show/NCT05950100](https://clinicaltrials.gov/ct2/show/NCT05950100)** | **Excluded** | **Review articles, conference papers** |
| **1035** | **Nct et al.** | **IO Vancomycin in TSA** | **[https://clinicaltrials.gov/ct2/show/NCT05831774](https://clinicaltrials.gov/ct2/show/NCT05831774)** | **Excluded** | **Review articles, conference papers** |
| **1036** | **Nct et al.** | **Mobilization With Movement Techniques of Shoulder Girdle in Patients With Chronic Adhesive Capsulitis** | **[https://clinicaltrials.gov/show/NCT05810766](https://clinicaltrials.gov/show/NCT05810766)** | **Excluded** | **Review articles, conference papers** |
| **1037** | **Nct et al.** | **Move ARound And Get Active: an Intervention to Optimize 24-hour Movement Behaviours in Preschoolers** | **[https://clinicaltrials.gov/ct2/show/NCT06171191](https://clinicaltrials.gov/ct2/show/NCT06171191)** | **Excluded** | **Review articles, conference papers** |
| **1038** | **Nct et al.** | **Oleanolic Acid as Therapeutic Adjuvant for Type 2 Diabetes Mellitus (OLTRAD STUDY)** | **[https://clinicaltrials.gov/ct2/show/NCT06030544](https://clinicaltrials.gov/ct2/show/NCT06030544)** | **Excluded** | **Review articles, conference papers** |
| **1039** | **Nct et al.** | **Pain Modulation Effectiveness (PME)** | **[https://clinicaltrials.gov/show/NCT05783362](https://clinicaltrials.gov/show/NCT05783362)** | **Excluded** | **Review articles, conference papers** |
| **1040** | **Nct et al.** | **Peanut Consumption on Cognitive, Weight, and Inflammation** | **[https://clinicaltrials.gov/ct2/show/NCT06127511](https://clinicaltrials.gov/ct2/show/NCT06127511)** | **Excluded** | **Review articles, conference papers** |
| **1041** | **Nct et al.** | **Physiology of Body Lateralization on Regional Lung Ventilation Study** | **[https://clinicaltrials.gov/ct2/show/NCT06044896](https://clinicaltrials.gov/ct2/show/NCT06044896)** | **Excluded** | **Review articles, conference papers** |
| **1042** | **Nct et al.** | **Postoperative Analgesic Effects of Ibuprofen Versus Ketorolac in Patients Undergoing in Orthopedic Surgery** | **[https://clinicaltrials.gov/show/NCT05695664](https://clinicaltrials.gov/show/NCT05695664)** | **Excluded** | **Review articles, conference papers** |
| **1043** | **Nct et al.** | **Postoperative Pain After Using Different Single-File Glide Path Systems** | **[https://clinicaltrials.gov/ct2/show/NCT05955742](https://clinicaltrials.gov/ct2/show/NCT05955742)** | **Excluded** | **Review articles, conference papers** |
| **1044** | **Nct et al.** | **Serratus Anterior Plane Block Versus Pericapsular Nerve Group Block for Shoulder Surgery** | **[https://clinicaltrials.gov/ct2/show/NCT05772533](https://clinicaltrials.gov/ct2/show/NCT05772533)** | **Excluded** | **Review articles, conference papers** |
| **1045** | **Nct et al.** | **Shoulder Block Versus Pericapsular Nerve Group Block for Shoulder Surgery** | **[https://clinicaltrials.gov/ct2/show/NCT05755802](https://clinicaltrials.gov/ct2/show/NCT05755802)** | **Excluded** | **Review articles, conference papers** |
| **1046** | **Nct et al.** | **Students Rising Above: offsetting the Health and Mental Health Costs of Resilience** | **[https://clinicaltrials.gov/ct2/show/NCT05846282](https://clinicaltrials.gov/ct2/show/NCT05846282)** | **Excluded** | **Review articles, conference papers** |
| **1047** | **Nct et al.** | **Telehealth and Onsite Maintenance Exercise in Chronic Lung Disease** | **[https://clinicaltrials.gov/ct2/show/NCT06304207](https://clinicaltrials.gov/ct2/show/NCT06304207)** | **Excluded** | **Review articles, conference papers** |
| **1048** | **Nct et al.** | **Virtual Phone Visits Compared to In-Person Physical Visits for Post-Operative Follow-Up at a Sports Medicine Clinic** | **[https://clinicaltrials.gov/ct2/show/NCT05998148](https://clinicaltrials.gov/ct2/show/NCT05998148)** | **Excluded** | **Review articles, conference papers** |
| **1049** | **Nct et al.** | **Assessing the Perioperative Outcomes of Minimally Invasive Posterior Approach Versus Direct Anterior Approach Total Hip Arthroplasty** | **[https://clinicaltrials.gov/ct2/show/NCT06659198](https://clinicaltrials.gov/ct2/show/NCT06659198)** | **Excluded** | **Review articles, conference papers** |
| **1050** | **Nct et al.** | **A Biomechanical Evaluation of the Ossur Power Knee in Persons With Transfemoral Amputation** | **[https://clinicaltrials.gov/ct2/show/NCT06218238](https://clinicaltrials.gov/ct2/show/NCT06218238)** | **Excluded** | **Review articles, conference papers** |
| **1051** | **Nct et al.** | **Comparative Study Between Single and Double Limb Hip Spica Cast in Fracture Femur in Young Children** | **[https://clinicaltrials.gov/ct2/show/NCT06430944](https://clinicaltrials.gov/ct2/show/NCT06430944)** | **Excluded** | **Review articles, conference papers** |
| **1052** | **Nct et al.** | **Comparison of Gluteus Maximus Strengthening and Hamstring Flexibility in Sacroiliac Joint Dysfinction** | **[https://clinicaltrials.gov/ct2/show/NCT06656195](https://clinicaltrials.gov/ct2/show/NCT06656195)** | **Excluded** | **Review articles, conference papers** |
| **1053** | **Nct et al.** | **Comparison of INIT and Dry Needling on Trigger Points in Knee OA** | **[https://clinicaltrials.gov/ct2/show/NCT06437730](https://clinicaltrials.gov/ct2/show/NCT06437730)** | **Excluded** | **Review articles, conference papers** |
| **1054** | **Nct et al.** | **Comparison of the Effect of Spinal Manipulation by Gender** | **[https://clinicaltrials.gov/ct2/show/NCT06578208](https://clinicaltrials.gov/ct2/show/NCT06578208)** | **Excluded** | **Review articles, conference papers** |
| **1055** | **Nct et al.** | **Core Stability Traning Exercises in Chronic Ankle Instability in Atheletes** | **[https://clinicaltrials.gov/ct2/show/NCT06678503](https://clinicaltrials.gov/ct2/show/NCT06678503)** | **Excluded** | **Review articles, conference papers** |
| **1056** | **Nct et al.** | **Cryotherapy Reduces Time to Surgery and Local Complication in Patients With Ankle Fractures** | **[https://clinicaltrials.gov/ct2/show/NCT06396364](https://clinicaltrials.gov/ct2/show/NCT06396364)** | **Excluded** | **Review articles, conference papers** |
| **1057** | **Nct et al.** | **Direct Superior Approach Versus PosteroLateral Approach in Total Hip Arthroplasty (SPLAsH)** | **[https://clinicaltrials.gov/ct2/show/NCT06342843](https://clinicaltrials.gov/ct2/show/NCT06342843)** | **Excluded** | **Review articles, conference papers** |
| **1058** | **Nct et al.** | **Effect of a Shortened FIFA11+ Warm-up Program and Sex-specific Footwear on Cutting in Youth Soccer Players** | **[https://clinicaltrials.gov/ct2/show/NCT06638346](https://clinicaltrials.gov/ct2/show/NCT06638346)** | **Excluded** | **Review articles, conference papers** |
| **1059** | **Nct et al.** | **Effect Of Closed Kinetic Chain On Hip Stability In Spastic Cerebral Palsy Post Selective Dorsal Rhizotomy** | **[https://clinicaltrials.gov/ct2/show/NCT06646718](https://clinicaltrials.gov/ct2/show/NCT06646718)** | **Excluded** | **Review articles, conference papers** |
| **1060** | **Nct et al.** | **The Effect of Contralateral Isokinetic Lower Extremity Exercises in Unilateral Painful Knee Osteoarthritis** | **[https://clinicaltrials.gov/ct2/show/NCT06675318](https://clinicaltrials.gov/ct2/show/NCT06675318)** | **Excluded** | **Review articles, conference papers** |
| **1061** | **Nct et al.** | **The Effect of Different Instruction Trainings in Semi-Professional Female Athletes** | **[https://clinicaltrials.gov/ct2/show/NCT06424886](https://clinicaltrials.gov/ct2/show/NCT06424886)** | **Excluded** | **Review articles, conference papers** |
| **1062** | **Nct et al.** | **Effect of Maitland Mobilization With Low Level Laser Therapy in Treatment of Patients With Knee Osteoarthritis** | **[https://clinicaltrials.gov/ct2/show/NCT06233955](https://clinicaltrials.gov/ct2/show/NCT06233955)** | **Excluded** | **Review articles, conference papers** |
| **1063** | **Nct et al.** | **Effect of Radial Extracorporeal Shockwave Therapy on Ultrasonography Changes in Patients With Iliotibial Band Syndrome** | **[https://clinicaltrials.gov/ct2/show/NCT06410781](https://clinicaltrials.gov/ct2/show/NCT06410781)** | **Excluded** | **Review articles, conference papers** |
| **1064** | **Nct et al.** | **Effect of Radial Shockwave Therapy and Graston Instrument Assisted Soft Tissue Mobilization on Plantar Fasciitis** | **[https://clinicaltrials.gov/ct2/show/NCT06697860](https://clinicaltrials.gov/ct2/show/NCT06697860)** | **Excluded** | **Review articles, conference papers** |
| **1065** | **Nct et al.** | **Effectiveness of Total Hip Arthroplasty for Patients With Osteoarthritis, a Target Trial Emulation Study** | **[https://clinicaltrials.gov/ct2/show/NCT06263569](https://clinicaltrials.gov/ct2/show/NCT06263569)** | **Excluded** | **Review articles, conference papers** |
| **1066** | **Nct et al.** | **The Effects of Aerobic Exercise in Patients With Primary Lower Extremity Lymphedema** | **[https://clinicaltrials.gov/ct2/show/NCT06327412](https://clinicaltrials.gov/ct2/show/NCT06327412)** | **Excluded** | **Review articles, conference papers** |
| **1067** | **Nct et al.** | **Effects of Barefoot vs. Shod Whole Body Vibration Training in Children With Cerebral Palsy** | **[https://clinicaltrials.gov/ct2/show/NCT06596525](https://clinicaltrials.gov/ct2/show/NCT06596525)** | **Excluded** | **Review articles, conference papers** |
| **1068** | **Nct et al.** | **Effects of Clinical Pilates Exercises on Glycemic Control, Blood Lipids and Physical Fitness Parameters in Prediabetes** | **[https://clinicaltrials.gov/ct2/show/NCT06452940](https://clinicaltrials.gov/ct2/show/NCT06452940)** | **Excluded** | **Review articles, conference papers** |
| **1069** | **Nct et al.** | **Evaluation of the Effectiveness of Hormonal Treatment in Adolescents Suffering From Gender Dysphoria** | **[https://clinicaltrials.gov/ct2/show/NCT06351501](https://clinicaltrials.gov/ct2/show/NCT06351501)** | **Excluded** | **Review articles, conference papers** |
| **1070** | **Nct et al.** | **High-Intensity Laser Therapy: effectiveness on Knee Osteoarthritis Patients** | **[https://clinicaltrials.gov/ct2/show/NCT06549543](https://clinicaltrials.gov/ct2/show/NCT06549543)** | **Excluded** | **Review articles, conference papers** |
| **1071** | **Nct et al.** | **The Immediate and 24-hour Effects of Body Tempering Versus Foam Rolling on Lower Extremity Muscular Power During the Vertical Jump and Standing Long Jump Tests** | **[https://clinicaltrials.gov/ct2/show/NCT06646926](https://clinicaltrials.gov/ct2/show/NCT06646926)** | **Excluded** | **Review articles, conference papers** |
| **1072** | **Nct et al.** | **Improvements in Function and Pain in the Rehabilitation of Patients with Knee Osteoarthritis Using an Exercise Program Telemonitored Compared to Conventional Treatment** | **[https://clinicaltrials.gov/ct2/show/NCT06647446](https://clinicaltrials.gov/ct2/show/NCT06647446)** | **Excluded** | **Review articles, conference papers** |
| **1073** | **Nct et al.** | **Influence of Cemented Cephalic Augmentation on the Outcome of Intramedullary Nailing in Pertrochanteric Hip Fractures** | **[https://clinicaltrials.gov/ct2/show/NCT06634290](https://clinicaltrials.gov/ct2/show/NCT06634290)** | **Excluded** | **Review articles, conference papers** |
| **1074** | **Nct et al.** | **Neurocognitive Ankle Training for Instability to Optimize Neuromusculoskeletal Outcomes (NATION)** | **[https://clinicaltrials.gov/ct2/show/NCT06527287](https://clinicaltrials.gov/ct2/show/NCT06527287)** | **Excluded** | **Review articles, conference papers** |
| **1075** | **Nct et al.** | **Percussive Therapy Reduced EMG Activity During Calf Raise in Limbs With and Without Chronic Ankle Instability** | **[https://clinicaltrials.gov/ct2/show/NCT06343207](https://clinicaltrials.gov/ct2/show/NCT06343207)** | **Excluded** | **Review articles, conference papers** |
| **1076** | **Nct et al.** | **Pharmacist-physiotherapist Collaborative Management for Early Knee Osteoarthritis** | **[https://clinicaltrials.gov/ct2/show/NCT06681142](https://clinicaltrials.gov/ct2/show/NCT06681142)** | **Excluded** | **Review articles, conference papers** |
| **1077** | **Nct et al.** | **Posterior Innominate Mobilization Versus Muscle Energy Technique on Lumbopelvic Angles in Sacroiliac Joint Dysfunction** | **[https://clinicaltrials.gov/ct2/show/NCT06280963](https://clinicaltrials.gov/ct2/show/NCT06280963)** | **Excluded** | **Review articles, conference papers** |
| **1078** | **Nct et al.** | **A Randomized Controlled Trial of the Sagittal Alignment Difference Between Mako Robotic TKA and Manual TKA** | **[https://clinicaltrials.gov/ct2/show/NCT06527911](https://clinicaltrials.gov/ct2/show/NCT06527911)** | **Excluded** | **Review articles, conference papers** |
| **1079** | **Nct et al.** | **Reliability of Range of Movement Measurements in Cerebral Palsy** | **[https://clinicaltrials.gov/ct2/show/NCT06295107](https://clinicaltrials.gov/ct2/show/NCT06295107)** | **Excluded** | **Review articles, conference papers** |
| **1080** | **Nct et al.** | **Two Standardized Radial Pressure Wave Techniques Versus Pain-Site Guided Therapy in Patients With Knee Osteoarthritis** | **[https://clinicaltrials.gov/ct2/show/NCT06622512](https://clinicaltrials.gov/ct2/show/NCT06622512)** | **Excluded** | **Review articles, conference papers** |
| **1081** | **Nct et al.** | **Wound Closure After Total Knee Arthroplasty: comparison of Polypropylene and Polyglactin 910 Suture** | **[https://clinicaltrials.gov/ct2/show/NCT06373900](https://clinicaltrials.gov/ct2/show/NCT06373900)** | **Excluded** | **Review articles, conference papers** |
| **1082** | **Nct et al.** | **Comparison of the Analgesic Effectiveness of Pericapsular Nerve Block and Suprainguinal Fascia Iliac Block Application** | **[https://clinicaltrials.gov/ct2/show/NCT06672289](https://clinicaltrials.gov/ct2/show/NCT06672289)** | **Excluded** | **Review articles, conference papers** |
| **1083** | **Nct et al.** | **Comparison Ultrasound-Guided Adductor Canal Block and Surgeon-Performed Block for Pain Management After Total Knee Arthroplasty** | **[https://clinicaltrials.gov/ct2/show/NCT06533085](https://clinicaltrials.gov/ct2/show/NCT06533085)** | **Excluded** | **Review articles, conference papers** |
| **1084** | **Nct et al.** | **Cost-effectiveness and Efficacy of Different Physical Exercise Interventions (ExerMOT4Health)** | **[https://clinicaltrials.gov/ct2/show/NCT06425679](https://clinicaltrials.gov/ct2/show/NCT06425679)** | **Excluded** | **Review articles, conference papers** |
| **1085** | **Nct et al.** | **Discovering Adolescents' Talents and Life Projects as a Key Factor in Their Life Satisfaction** | **[https://clinicaltrials.gov/ct2/show/NCT06473155](https://clinicaltrials.gov/ct2/show/NCT06473155)** | **Excluded** | **Review articles, conference papers** |
| **1086** | **Nct et al.** | **E-Based Physical Exercise in Patients With Multiple Sclerosis and Comorbidity** | **[https://clinicaltrials.gov/ct2/show/NCT06298201](https://clinicaltrials.gov/ct2/show/NCT06298201)** | **Excluded** | **Review articles, conference papers** |
| **1087** | **Nct et al.** | **EFFECT of BRAIN GYM EXERCISES on RISK of FALL, BALANCE and QUALITY of LIFE in OBESE SUBJECTS** | **[https://clinicaltrials.gov/ct2/show/NCT06587932](https://clinicaltrials.gov/ct2/show/NCT06587932)** | **Excluded** | **Review articles, conference papers** |
| **1088** | **Nct et al.** | **Effect of Pericapsular Nerve Group Block on Postoperative Cognitive Function** | **[https://clinicaltrials.gov/ct2/show/NCT06736405](https://clinicaltrials.gov/ct2/show/NCT06736405)** | **Excluded** | **Review articles, conference papers** |
| **1089** | **Nct et al.** | **Effect of Task Oriented Training on Cognitive Function in Elderly Stroke Patients** | **[https://clinicaltrials.gov/ct2/show/NCT06445998](https://clinicaltrials.gov/ct2/show/NCT06445998)** | **Excluded** | **Review articles, conference papers** |
| **1090** | **Nct et al.** | **Effectiveness of Inspiratory Muscle Training for People with Ischemic Heart Disease Revascularized by Percutaneous Transluminal Coronary Angioplasty** | **[https://clinicaltrials.gov/ct2/show/NCT06681740](https://clinicaltrials.gov/ct2/show/NCT06681740)** | **Excluded** | **Review articles, conference papers** |
| **1091** | **Nct et al.** | **Effectiveness of Telerehabilitation in Balance Training Program** | **[https://clinicaltrials.gov/ct2/show/NCT06210828](https://clinicaltrials.gov/ct2/show/NCT06210828)** | **Excluded** | **Review articles, conference papers** |
| **1092** | **Nct et al.** | **Effects of a CSE With ADIM in Chronic Non-specific Low Back Pain With Lumbar Instability Using Telerehabilitation** | **[https://clinicaltrials.gov/ct2/show/NCT06321393](https://clinicaltrials.gov/ct2/show/NCT06321393)** | **Excluded** | **Review articles, conference papers** |
| **1093** | **Nct et al.** | **Effects of Dance and Tai Chi on Balance and Wellbeing on Healthy Adults** | **[https://clinicaltrials.gov/ct2/show/NCT06294080](https://clinicaltrials.gov/ct2/show/NCT06294080)** | **Excluded** | **Review articles, conference papers** |
| **1094** | **Nct et al.** | **Effects of IASTM Along With Comprehensive Corrective Exercise Program in Upper Cross Syndrome** | **[https://clinicaltrials.gov/ct2/show/NCT06302972](https://clinicaltrials.gov/ct2/show/NCT06302972)** | **Excluded** | **Review articles, conference papers** |
| **1095** | **Nct et al.** | **Effects of Stress Ball and Hand Holdig on Pain and Mobility Levels During Continuous Passive Motion In Patients With Total Knee Prosthesis: a Randomized Controlled Trial** | **[https://clinicaltrials.gov/ct2/show/NCT06608264](https://clinicaltrials.gov/ct2/show/NCT06608264)** | **Excluded** | **Review articles, conference papers** |
| **1096** | **Nct et al.** | **Efficacy of Extracorporeal Shock Wave Therapy on Spasticity** | **[https://clinicaltrials.gov/ct2/show/NCT06225024](https://clinicaltrials.gov/ct2/show/NCT06225024)** | **Excluded** | **Review articles, conference papers** |
| **1097** | **Nct et al.** | **Efficacy of Local Antibiotic Injection Via a Catheter for Treating Acute Periprosthetic Infections After Knee DAIR Surgery** | **[https://clinicaltrials.gov/ct2/show/NCT06468163](https://clinicaltrials.gov/ct2/show/NCT06468163)** | **Excluded** | **Review articles, conference papers** |
| **1098** | **Nct et al.** | **Evaluation Of Pain, Balance, Functional Performance and Quality of Life in Patients With Meniscus Lesions** | **[https://clinicaltrials.gov/ct2/show/NCT06446973](https://clinicaltrials.gov/ct2/show/NCT06446973)** | **Excluded** | **Review articles, conference papers** |
| **1099** | **Nct et al.** | **Examining the Effects of Morning and Evening Exercise on Glucose Regulation in Adults With Prediabetes** | **[https://clinicaltrials.gov/ct2/show/NCT06292000](https://clinicaltrials.gov/ct2/show/NCT06292000)** | **Excluded** | **Review articles, conference papers** |
| **1100** | **Nct et al.** | **Global Changes Associated with Sacroiliac Joint Dysfunction** | **[https://clinicaltrials.gov/ct2/show/NCT06687148](https://clinicaltrials.gov/ct2/show/NCT06687148)** | **Excluded** | **Review articles, conference papers** |
| **1101** | **Nct et al.** | **The Impact of Cardiac Rehabilitation Exercise Therapy on the Quality of Life of Patients With Chronic Heart Failure** | **[https://clinicaltrials.gov/ct2/show/NCT06633107](https://clinicaltrials.gov/ct2/show/NCT06633107)** | **Excluded** | **Review articles, conference papers** |
| **1102** | **Nct et al.** | **Investigation of the Effectiveness of Cognitive Exercise Therapy Approach in Patients With Primary Sjögren's Syndrome** | **[https://clinicaltrials.gov/ct2/show/NCT06398210](https://clinicaltrials.gov/ct2/show/NCT06398210)** | **Excluded** | **Review articles, conference papers** |
| **1103** | **Nct et al.** | **Lifestyle Intervention to Reduce the Risk of Sarcopenia in Adults With Diabetes & Obesity Treated With Semaglutide** | **[https://clinicaltrials.gov/ct2/show/NCT06497595](https://clinicaltrials.gov/ct2/show/NCT06497595)** | **Excluded** | **Review articles, conference papers** |
| **1104** | **Nct et al.** | **Low Dose Bupivacaine Versus Prilocaine Regarding Hemodynamic Stability and Safety in Geriatrics** | **[https://clinicaltrials.gov/ct2/show/NCT06382220](https://clinicaltrials.gov/ct2/show/NCT06382220)** | **Excluded** | **Review articles, conference papers** |
| **1105** | **Nct et al.** | **Mepiform in Simultaneous Bilateral TKA** | **[https://clinicaltrials.gov/ct2/show/NCT06312527](https://clinicaltrials.gov/ct2/show/NCT06312527)** | **Excluded** | **Review articles, conference papers** |
| **1106** | **Nct et al.** | **Neuromuscular Training & Postural Stability** | **[https://clinicaltrials.gov/ct2/show/NCT06294002](https://clinicaltrials.gov/ct2/show/NCT06294002)** | **Excluded** | **Review articles, conference papers** |
| **1107** | **Nct et al.** | **Plyometry and Tapping in the Functional Improvement of Non-professional Basketball Players** | **[https://clinicaltrials.gov/ct2/show/NCT06367790](https://clinicaltrials.gov/ct2/show/NCT06367790)** | **Excluded** | **Review articles, conference papers** |
| **1108** | **Nct et al.** | **Prevention and Early Detection of Ulcer Recurrence in Patients With Type II Diabetes Mellitus** | **[https://clinicaltrials.gov/ct2/show/NCT06434922](https://clinicaltrials.gov/ct2/show/NCT06434922)** | **Excluded** | **Review articles, conference papers** |
| **1109** | **Nct et al.** | **Quality Of Recovery After Pericapsular Nerve Group (PENG) Block For Hip Hemiarthroplasty Under Spinal Anesthesia** | **[https://clinicaltrials.gov/ct2/show/NCT06369948](https://clinicaltrials.gov/ct2/show/NCT06369948)** | **Excluded** | **Review articles, conference papers** |
| **1110** | **Nct et al.** | **Real-component vs All-cement Articulating Spacers for Periprosthetic Knee Infection** | **[https://clinicaltrials.gov/ct2/show/NCT06293352](https://clinicaltrials.gov/ct2/show/NCT06293352)** | **Excluded** | **Review articles, conference papers** |
| **1111** | **Nct et al.** | **The Short-Term Effects of Dry Cupping the Lumbar Paraspinal Muscles in Individuals With Non-specific Low Back Pain** | **[https://clinicaltrials.gov/ct2/show/NCT06469762](https://clinicaltrials.gov/ct2/show/NCT06469762)** | **Excluded** | **Review articles, conference papers** |
| **1112** | **Nct et al.** | **Therapeutic Photobiomodulation and Tretament of Spasticity** | **[https://clinicaltrials.gov/ct2/show/NCT06536751](https://clinicaltrials.gov/ct2/show/NCT06536751)** | **Excluded** | **Review articles, conference papers** |
| **1113** | **Nct et al.** | **Treadmill Training at Variable Inclinations in Children With Cerebral Palsy** | **[https://clinicaltrials.gov/ct2/show/NCT06216652](https://clinicaltrials.gov/ct2/show/NCT06216652)** | **Excluded** | **Review articles, conference papers** |
| **1114** | **Nct et al.** | **Use of Unmanned Air Vehicles (Medical Drones) to Overcome Geographical Barriers to Delivery of Anti-Retrovical Samples** | **[https://clinicaltrials.gov/ct2/show/NCT06678022](https://clinicaltrials.gov/ct2/show/NCT06678022)** | **Excluded** | **Review articles, conference papers** |
| **1115** | **Nct et al** | **Comparing Vascular Responses to Resistance Exercise with and without Blood Flow Restriction in Young and Older Adults** | **[https://clinicaltrials.gov/ct2/show/NCT06596304](https://clinicaltrials.gov/ct2/show/NCT06596304)** | **Excluded** | **Review articles, conference papers** |
| **1116** | **Nct et al** | **Comparison of Teaching Methods of Pelvic Floor Muscle Contraction in Women** | **[https://clinicaltrials.gov/ct2/show/NCT06306703](https://clinicaltrials.gov/ct2/show/NCT06306703)** | **Excluded** | **Review articles, conference papers** |
| **1117** | **Nct et al** | **Comparison of Unilateral and Bilateral Transforaminal Epidural Steroid Injection** | **[https://clinicaltrials.gov/ct2/show/NCT06240793](https://clinicaltrials.gov/ct2/show/NCT06240793)** | **Excluded** | **Review articles, conference papers** |
| **1118** | **Nct et al** | **Connective Tissue Matrix Compared to Steroid Injections for Rotator Cuff Tendinopathy** | **[https://clinicaltrials.gov/ct2/show/NCT06712290](https://clinicaltrials.gov/ct2/show/NCT06712290)** | **Excluded** | **Review articles, conference papers** |
| **1119** | **Nct et al** | **CREM - Clinical and Functional Outcomes in a Controlled Clinical Trial with Older Adults** | **[https://clinicaltrials.gov/ct2/show/NCT06638697](https://clinicaltrials.gov/ct2/show/NCT06638697)** | **Excluded** | **Review articles, conference papers** |
| **1120** | **Nct et al** | **The Effect of Digital Education for Osteoporosis Patients on Fracture Risk and Related Health Outcomes** | **[https://clinicaltrials.gov/ct2/show/NCT06603545](https://clinicaltrials.gov/ct2/show/NCT06603545)** | **Excluded** | **Review articles, conference papers** |
| **1121** | **Nct et al** | **The Effect of Hamstring Stretching on Lumbar Muscle Activation in Officers** | **[https://clinicaltrials.gov/ct2/show/NCT06540053](https://clinicaltrials.gov/ct2/show/NCT06540053)** | **Excluded** | **Review articles, conference papers** |
| **1122** | **Nct et al** | **Effect of Hypopressive Exercises on Postpartum Backache and Functional Disability** | **[https://clinicaltrials.gov/ct2/show/NCT06259474](https://clinicaltrials.gov/ct2/show/NCT06259474)** | **Excluded** | **Review articles, conference papers** |
| **1123** | **Nct et al** | **Effect of Isometric and Aerobic Physical Exercise on Blood Pressure Levels in Hypertensive Elderly People** | **[https://clinicaltrials.gov/ct2/show/NCT06282302](https://clinicaltrials.gov/ct2/show/NCT06282302)** | **Excluded** | **Review articles, conference papers** |
| **1124** | **Nct et al** | **The Effect of Low-load Endurance Training of Upper Trapezius on Pain, Pressure Pain and Muscle Stiffness in Chronic Neck-shoulder Pain Compared to Stretching Exercise** | **[https://clinicaltrials.gov/ct2/show/NCT06635759](https://clinicaltrials.gov/ct2/show/NCT06635759)** | **Excluded** | **Review articles, conference papers** |
| **1125** | **Nct et al** | **The Effect of Minimally Invasive Technique Assisted by Magnetic Resonance Neurography** | **[https://clinicaltrials.gov/ct2/show/NCT06260839](https://clinicaltrials.gov/ct2/show/NCT06260839)** | **Excluded** | **Review articles, conference papers** |
| **1126** | **Nct et al** | **Effect of Shoe Cushioning Position and Properties on Running-related Injury Risk** | **[https://clinicaltrials.gov/ct2/show/NCT06384872](https://clinicaltrials.gov/ct2/show/NCT06384872)** | **Excluded** | **Review articles, conference papers** |
| **1127** | **Nct et al** | **The Effect of White Noise and Swaddling on Pain, Heart Rate, and Oxygen Saturation in Term Infants Undergoing Eye Examinations** | **[https://clinicaltrials.gov/ct2/show/NCT06535984](https://clinicaltrials.gov/ct2/show/NCT06535984)** | **Excluded** | **Review articles, conference papers** |
| **1128** | **Nct et al** | **Effect of Yoga Program on Quality of Life in Women With Breast Cancer Receiving Endocrine Therapy** | **[https://clinicaltrials.gov/ct2/show/NCT06605261](https://clinicaltrials.gov/ct2/show/NCT06605261)** | **Excluded** | **Review articles, conference papers** |
| **1129** | **Nct et al** | **The Effectiveness of Osteopathic Treatment in Cervical Whiplash** | **[https://clinicaltrials.gov/ct2/show/NCT06334978](https://clinicaltrials.gov/ct2/show/NCT06334978)** | **Excluded** | **Review articles, conference papers** |
| **1130** | **Nct et al** | **Effects of a Microalgae Extract Dietary Supplement on Gut Health, Anxiety, and Immune Function** | **[https://clinicaltrials.gov/ct2/show/NCT06425094](https://clinicaltrials.gov/ct2/show/NCT06425094)** | **Excluded** | **Review articles, conference papers** |
| **1131** | **Nct et al** | **Effects of Acute Physical Activity in Patients With Exhaustion Disorder** | **[https://clinicaltrials.gov/ct2/show/NCT06429423](https://clinicaltrials.gov/ct2/show/NCT06429423)** | **Excluded** | **Review articles, conference papers** |
| **1132** | **Nct et al** | **Effects of Core Stability Exercises on Motor Control in Patients With Low Back Pain** | **[https://clinicaltrials.gov/ct2/show/NCT06686264](https://clinicaltrials.gov/ct2/show/NCT06686264)** | **Excluded** | **Review articles, conference papers** |
| **1133** | **Nct et al** | **Embracing Morning Breakfast and Activity for Classroom Engagement** | **[https://clinicaltrials.gov/ct2/show/NCT06555627](https://clinicaltrials.gov/ct2/show/NCT06555627)** | **Excluded** | **Review articles, conference papers** |
| **1134** | **Nct et al** | **Evaluation of Locally Delivered Propolis Gels in Periodontitis** | **[https://clinicaltrials.gov/ct2/show/NCT06663995](https://clinicaltrials.gov/ct2/show/NCT06663995)** | **Excluded** | **Review articles, conference papers** |
| **1135** | **Nct et al** | **Evaluation of the Arterial Occlusion Effect on Upper and Lower Limbs by the Application of Different Pre-hospital Tourniquet Models** | **[https://clinicaltrials.gov/ct2/show/NCT06725602](https://clinicaltrials.gov/ct2/show/NCT06725602)** | **Excluded** | **Review articles, conference papers** |
| **1136** | **Nct et al** | **Examining the Effectiveness of Exercise Training After Cervical Laminoplasty Surgery** | **[https://clinicaltrials.gov/ct2/show/NCT06279377](https://clinicaltrials.gov/ct2/show/NCT06279377)** | **Excluded** | **Review articles, conference papers** |
| **1137** | **Nct et al** | **Fascial Tissue Response to Manual Therapy: implications in Long COVID-19** | **[https://clinicaltrials.gov/ct2/show/NCT06348186](https://clinicaltrials.gov/ct2/show/NCT06348186)** | **Excluded** | **Review articles, conference papers** |
| **1138** | **Nct et al** | **Intervention Effects of High-intensity Tai Chi in Mild or Moderate Asthma** | **[https://clinicaltrials.gov/ct2/show/NCT06728904](https://clinicaltrials.gov/ct2/show/NCT06728904)** | **Excluded** | **Review articles, conference papers** |
| **1139** | **Nct et al** | **Leggings With Resistance Bands on Caloric Expenditure During Exercise** | **[https://clinicaltrials.gov/ct2/show/NCT06547060](https://clinicaltrials.gov/ct2/show/NCT06547060)** | **Excluded** | **Review articles, conference papers** |
| **1140** | **Nct et al** | **Pelvic Floor Muscle Activity, Respiratory Functions, Respiratory Muscle Strength, and Functional Capacity in Children with Lower Urinary Tract Dysfunction** | **[https://clinicaltrials.gov/ct2/show/NCT06652178](https://clinicaltrials.gov/ct2/show/NCT06652178)** | **Excluded** | **Review articles, conference papers** |
| **1141** | **Nct et al** | **Prolonged Cryocompression and Skin Temperature: a Safety and Feasibility Pilot** | **[https://clinicaltrials.gov/ct2/show/NCT06673277](https://clinicaltrials.gov/ct2/show/NCT06673277)** | **Excluded** | **Review articles, conference papers** |
| **1142** | **Nct et al** | **Randomizing Vignettes to Understand Contributors to Trust in Primary Care Doctors** | **[https://clinicaltrials.gov/ct2/show/NCT06526663](https://clinicaltrials.gov/ct2/show/NCT06526663)** | **Excluded** | **Review articles, conference papers** |
| **1143** | **Nct et al** | **Rivaroxaban Versus Enoxaparin for Prophylaxis of Venous Thromboembolism in Bariatric Surgery** | **[https://clinicaltrials.gov/ct2/show/NCT06689241](https://clinicaltrials.gov/ct2/show/NCT06689241)** | **Excluded** | **Review articles, conference papers** |
| **1144** | **Nct et al** | **The ROAMM-EHR Study** | **[https://clinicaltrials.gov/ct2/show/NCT06263322](https://clinicaltrials.gov/ct2/show/NCT06263322)** | **Excluded** | **Review articles, conference papers** |
| **1145** | **Nct et al** | **Safety & Efficacy of Ischemic Preconditioning by Embolization of the Inferior Mesenteric Artery in Surgery for Tumors of Lower and Middle Rectum** | **[https://clinicaltrials.gov/ct2/show/NCT06236633](https://clinicaltrials.gov/ct2/show/NCT06236633)** | **Excluded** | **Review articles, conference papers** |
| **1146** | **Nct et al** | **Thoracic Spinal Versus Thoracic Epidural Anesthesia for Patients with Chronic Obstructive Pulmonary Disease Undergoing Supine Percutaneous Nephrolithotomy for Management of Renal Stones** | **[https://clinicaltrials.gov/ct2/show/NCT06663488](https://clinicaltrials.gov/ct2/show/NCT06663488)** | **Excluded** | **Review articles, conference papers** |
| **1147** | **Nct et al** | **Understanding Food Choices in Bahrain** | **[https://clinicaltrials.gov/ct2/show/NCT06440421](https://clinicaltrials.gov/ct2/show/NCT06440421)** | **Excluded** | **Review articles, conference papers** |
| **1148** | **Nct et al** | **Effectiveness of Probiotics for the Prevention of Gastrointestinal Toxicity in Children With Leukemia** | **[https://clinicaltrials.gov/ct2/show/NCT06560879](https://clinicaltrials.gov/ct2/show/NCT06560879)** | **Excluded** | **Review articles, conference papers** |
| **1149** | **R. B. R. et al** | **Intervention with walking and balance training using shoes and insoles combined with pain education in elderly women with knee osteoarthritis** | **[https://trialsearch.who.int/Trial2.aspx?TrialID=RBR-10ncwk79](https://trialsearch.who.int/Trial2.aspx?TrialID=RBR-10ncwk79)** | **Excluded** | **Not related to the purpose of the article** |
| **1150** | **Nedelec et al** | **Recovery in Soccer Part I - Post-Match Fatigue and Time Course of Recovery** | **[https://www.ncbi.nlm.nih.gov/pmc/articles/PMC3445208/](https://www.ncbi.nlm.nih.gov/pmc/articles/PMC3445208/)** | **Excluded** | **Not related to the purpose of the article** |
| **1151** | **Neder et al** | **Prediction of metabolic and cardiopulmonary responses to maximum cycle ergometry: a randomised study** | **[https://erj.ersjournals.com/content/14/6/1304](https://erj.ersjournals.com/content/14/6/1304)** | **Excluded** | **Not related to inclusion criteria** |
| **1152** | **Neder et al** | **Reference values for concentric knee isokinetic strength and power in nonathletic men and women from 20 to 80 years old** | **[https://www.jospt.org/doi/abs/10.2519/jospt.29.2.116](https://www.jospt.org/doi/abs/10.2519/jospt.29.2.116)** | **Excluded** | **Not related to inclusion criteria** |
| **1153** | **Neeraja et al** | **To study the efficacy of nalbuphine and fentanyl as adjuvants to epidural ropivacaine in lower abdomen and lower extremity surgeries in the patients attending tertiary care hospital, South India** | **[https://www.ajol.info/index.php/ajbs/article/view/107978](https://www.ajol.info/index.php/ajbs/article/view/107978)** | **Excluded** | **Not related to the purpose of the article** |
| **1154** | **Negus et al** | **Physical Training Outcome Predictions With Biomechanics, Part II: Overuse Injury Modeling** | **[https://www.tandfonline.com/doi/full/10.1080/00264476.2016.1167547](https://www.tandfonline.com/doi/full/10.1080/00264476.2016.1167547)** | **Excluded** | **Not related to the purpose of the article** |
| **1155** | **Nehir et al** | **A Comparison of Physical Performance, Upper and Lower Extremity Muscle Strength and Balance in Patients with Peripheral Arterial Disease and Healthy People** | **[https://www.researchgate.net/publication/261490921_A_Comparison_of_Physical_Performance_Upper_and_Lower_Extremity_Muscle_Strength_and_Balance_in_Patients_with_Peripheral_Arterial_Disease_and_Healthy_People](https://www.researchgate.net/publication/261490921_A_Comparison_of_Physical_Performance_Upper_and_Lower_Extremity_Muscle_Strength_and_Balance_in_Patients_with_Peripheral_Arterial_Disease_and_Healthy_People)** | **Excluded** | **Not related to the purpose of the article** |
| **1156** | **Neidlein et al** | **Iron deficiency, fatigue and muscle strength and function in older hospitalized patients** | **[https://www.nature.com/articles/s41596-021-00756-1](https://www.nature.com/articles/s41596-021-00756-1)** | **Excluded** | **Not related to the purpose of the article** |
| **1157** | **Neki et al** | **Clinical efficacy and safety profile of clindipine versus amlodipine in essential hypertension** | **[https://www.researchgate.net/publication/283675784_Clinical_efficacy_and_safety_profile_of_clindipine_versus_amlodipine_in_essential_hypertension](https://www.researchgate.net/publication/283675784_Clinical_efficacy_and_safety_profile_of_clindipine_versus_amlodipine_in_essential_hypertension)** | **Excluded** | **Not related to inclusion criteria** |
| **1158** | **Nelson et al** | **Cross-bridge mechanisms of skeletal muscle fatigue: Effects of hydrogen ion, inorganic phosphate, and age** | **[https://www.researchgate.net/publication/269140319_Cross-bridge_mechanisms_of_skeletal_muscle_fatigue_Effects_of_hydrogen_ion_inorganic_phosphate_and_age](https://www.researchgate.net/publication/269140319_Cross-bridge_mechanisms_of_skeletal_muscle_fatigue_Effects_of_hydrogen_ion_inorganic_phosphate_and_age)** | **Excluded** | **Not related to the purpose of the article** |
| **1159** | **Nelson et al** | **An oral preparation containing hylauronic acid (ORALVISC) can reduce osteoarthritis knee pain and serum and synovia fluid bradykinin** | **[https://www.elsevier.com/connect/oralvisc-reduces-osteoarthritis-knee-pain-and-bradykinin-levels](https://www.elsevier.com/connect/oralvisc-reduces-osteoarthritis-knee-pain-and-bradykinin-levels)** | **Excluded** | **Not related to inclusion criteria** |
| **1160** | **Neltner et al** | **Velocity-Specific Coactivation and Neuromuscular Responses to Fatiguing, Reciprocal, Isokinetic, Forearm Flexion, and Extension Muscle Actions** | **[https://journals.lww.com/nsca-jscr/Abstract/2022/03000/Velocity_Specific_Coactivation_and_Neuromuscular_Responses.11.aspx](https://journals.lww.com/nsca-jscr/Abstract/2022/03000/Velocity_Specific_Coactivation_and_Neuromuscular_Responses.11.aspx)** | **Excluded** | **Not related to the purpose of the article** |
| **1161** | **Neltner et al** | **Coactivation does not contribute to fatigue-induced decreases in torque during reciprocal, isokinetic muscle actions** | **[https://content.iospress.com/articles/isokinetics-and-exercise-science/ies210002](https://content.iospress.com/articles/isokinetics-and-exercise-science/ies210002)** | **Excluded** | **Not related to the purpose of the article** |
| **1162** | **Neltner et al** | **Coactivation Does Not Contribute to Fatigue-Induced Decreases in Isokinetic Forearm Flexion and Extension Torque** | **[https://www.tandfonline.com/doi/full/10.1080/24726386.2023.2222977](https://www.tandfonline.com/doi/full/10.1080/24726386.2023.2222977)** | **Excluded** | **Not related to inclusion criteria** |
| **1163** | **Nemet et al** | **Evidence for exercise-induced bone formation in premature infants** | **[https://www.thieme-connect.de/products/journals/article?doi=10.1055/s-0034-102475](https://www.thieme-connect.de/products/journals/article?doi=10.1055/s-0034-102475)** | **Excluded** | **Not related to the purpose of the article** |
| **1164** | **Nestel et al** | **Enhanced blood pressure response to dietary salt in elderly women, especially those with small waist: hip ratio** | **[https://www.tandfonline.com/doi/abs/10.1097/00004872.1993.10911121387](https://www.tandfonline.com/doi/abs/10.1097/00004872.1993.10911121387)** | **Excluded** | **Not related to the purpose of the article** |
| **1165** | **Nestler et al** | **Index Finger Muscle Fatigue and Pistol Firing Failure** | **[https://journals.sagepub.com/doi/full/10.1177/0018720819877848](https://journals.sagepub.com/doi/full/10.1177/0018720819877848)** | **Excluded** | **Not related to the purpose of the article** |
| **1166** | **Neumann et al** | **Provision of high-protein supplement for patients recovering from hip fracture** | **[https://www.tandfonline.com/doi/full/10.1080/14768320041000206](https://www.tandfonline.com/doi/full/10.1080/14768320041000206)** | **Excluded** | **Not related to inclusion criteria** |
| **1167** | **Newstead et al** | **The effect of a jumping exercise intervention on bone mineral density in postmenopausal women** | **[https://www.jgpt.org/doi/abs/10.1080/151898102702047](https://www.jgpt.org/doi/abs/10.1080/151898102702047)** | **Excluded** | **Not related to inclusion criteria** |
| **1168** | **Neyroud et al** | **Comparison of neuromuscular adjustments associated with sustained isometric contractions of four different muscle groups** | **[https://www.physiology.org/doi/full/10.1152/japplphysiol.00002.2013](https://www.physiology.org/doi/full/10.1152/japplphysiol.00002.2013)** | **Excluded** | **Not related to the purpose of the article** |
| **1169** | **Ng et al** | **Body shape and fat distribution from whole-body DXA accurately predict metabolic syndrome across sex and ethnicity subgroups** | **[https://www.tandfonline.com/doi/full/10.1080/10971910.2016.1239186](https://www.tandfonline.com/doi/full/10.1080/10971910.2016.1239186)** | **Excluded** | **Not related to the purpose of the article** |
| **1170** | **Ng et al** | **Gender Differences in Trunk and Pelvic Kinematics During Prolonged Ergometer Rowing in Adolescents** | **[https://journals.humankinetics.com/view/journals/jab/29/2/article-p180.xml](https://journals.humankinetics.com/view/journals/jab/29/2/article-p180.xml)** | **Excluded** | **Not related to the purpose of the article** |
| **1171** | **Ng et al** | **A comparative study of three warming interventions to determine the most effective in maintaining perioperative normothermia** | **[https://journals.lww.com/anesthesia-analgesia/Abstract/2003/01000/A_comparative_study_of_three_warming_interventions_to.27.aspx](https://journals.lww.com/anesthesia-analgesia/Abstract/2003/01000/A_comparative_study_of_three_warming_interventions_to.27.aspx)** | **Excluded** | **Not related to inclusion criteria** |
| **1172** | **Ngeno et al** | **Outcomes of a Randomized Controlled Trial of Integrated Cardiac Rehabilitation among Heart Failure Patients in Kenya** | **[https://www.ahajournals.org/doi/10.1161/circulation.142.suppl_3.14427](https://www.ahajournals.org/doi/10.1161/circulation.142.suppl_3.14427)** | **Excluded** | **Not related to the purpose of the article** |
| **1173** | **Nguyen et al** | **Disparity in outcomes of surgical revascularization for limb salvage: race and gender are synergistic determinants of vein graft failure and limb loss** | **[https://www.ahajournals.org/doi/10.1161/circulation.119.1.123](https://www.ahajournals.org/doi/10.1161/circulation.119.1.123)** | **Excluded** | **Not related to inclusion criteria** |
| **1174** | **Nguyen et al** | **Okara Improved Blood Glucose Level in Vietnamese with Type 2 Diabetes Mellitus** | **[https://www.jnsv.org/article/65/1/60](https://www.jnsv.org/article/65/1/60)** | **Excluded** | **Not related to the purpose of the article** |
| **1175** | **Ni et al** | **Therapeutic effect of San Bi Tang combined with glucosamine sulfate capsules in cold-dampness-type knee osteoarthritis** | **[https://www.wjgnet.com/1948-4339/full/v12/i19/3854.htm](https://www.wjgnet.com/1948-4339/full/v12/i19/3854.htm)** | **Excluded** | **Not related to inclusion criteria** |
| **1176** | **Ni et al** | **Strengthening control of blood glucose, blood pressure, and blood lipids in type 2 diabetes mellitus patients: influence on obestatin level and relation with lipid metabolism** | **[https://www.researchgate.net/publication/23074678_Strengthening_control_of_blood_glucose_blood_pressure_and_blood_lipids_in_type_2_diabetes_mellitus_patients_influence_on_obestatin_level_and_relation_with_lipid_metabolism](https://www.researchgate.net/publication/23074678_Strengthening_control_of_blood_glucose_blood_pressure_and_blood_lipids_in_type_2_diabetes_mellitus_patients_influence_on_obestatin_level_and_relation_with_lipid_metabolism)** | **Excluded** | **Not related to the purpose of the article** |
| **1177** | **Nicastro et al** | **Characterizing strategies used by HIV-infected smallholder farmers to mitigate the effects of climate change in the Nyanza region of Kenya** | **[https://www.jiasociety.org/jias/characterizing-strategies-used-by-hiv-infected-smallholder-farmers-to-mitigate-the-effects-of-climate-change-in-the-nyanza-region-of-kenya](https://www.jiasociety.org/jias/characterizing-strategies-used-by-hiv-infected-smallholder-farmers-to-mitigate-the-effects-of-climate-change-in-the-nyanza-region-of-kenya)** | **Excluded** | **Not related to inclusion criteria** |
| **1178** | **Nickel et al** | **Joules, Genes, and Behaviors: a multifactorial assessment of TMD risk indicators** | **[https://www.researchgate.net/publication/258778871_Joules_Genes_and_Behaviors_a_multifactorial_assessment_of_TMD_risk_indicators](https://www.researchgate.net/publication/258778871_Joules_Genes_and_Behaviors_a_multifactorial_assessment_of_TMD_risk_indicators)** | **Excluded** | **Not related to inclusion criteria** |
| **1179** | **Nicolaisen et al** | **TRUNK STRENGTH, BACK MUSCLE ENDURANCE AND LOW-BACK TROUBLE** | **[https://www.tandfonline.com/doi/abs/10.1080/08017724.1985.10972121](https://www.tandfonline.com/doi/abs/10.1080/08017724.1985.10972121)** | **Excluded** | **Not related to inclusion criteria** |
| **1180** | **Nicolson et al** | **The long term effect of inhaled hypertonic saline (6%) in non cystic fibrosis bronchiectasis** | **[https://www.atsjournals.org/doi/full/10.1164/rccm.201001-0054OC](https://www.atsjournals.org/doi/full/10.1164/rccm.201001-0054OC)** | **Excluded** | **Not related to the purpose of the article** |
| **1181** | **Nicot et al** | **Three-Dimensional Printing Model Enhances Craniofacial Trauma Teaching by Improving Morphologic and Biomechanical Understanding: a Randomized Controlled Study** | **[https://journals.lww.com/plasreconsurg/Abstract/2022/03000/Three_Dimensional_Printing_Model_Enhances_Craniofacial_17.aspx](https://journals.lww.com/plasreconsurg/Abstract/2022/03000/Three_Dimensional_Printing_Model_Enhances_Craniofacial_17.aspx)** | **Excluded** | **Not related to the purpose of the article** |
| **1182** | **Nie et al** | **Gender effects on trapezius surface EMG during delayed onset muscle soreness due to eccentric shoulder exercise** | **[https://www.sciencedirect.com/science/article/pii/S1050641107000774](https://www.sciencedirect.com/science/article/pii/S1050641107000774)** | **Excluded** | **Not related to inclusion criteria** |
| **1183** |  | **A Clinical and Comparative Study of Purified Guggulu and Bark Powder of Arjuna in the Fatty Persion.** | **<https://trialsearch.who.int/Trial2.aspx?TrialID=CTRI/2024/10/076035>** | **Excluded** | **Review articles, conference papers** |
| **1184** |  | **A Clinical Study on Modified Upanaha in Knee Joint Pain(Osteoarthritis).** | **<https://trialsearch.who.int/Trial2.aspx?TrialID=CTRI/2024/04/066068>** | **Excluded** | **Review articles, conference papers** |
| **1185** |  | **A Clinical Study to Compare the Effect of Bala Taila and Ketakyadi Taila Orally with Kottamchukkadi Taila Filling Illing on Knee Joint the Management of Janusadhigata Vata Vis a Vis Osteoarthritis of Knee Jount.** | **<https://trialsearch.who.int/Trial2.aspx?TrialID=CTRI/2024/09/073228>** | **Excluded** | **Review articles, conference papers** |
| **1186** |  | **A Clinical Study to See the Effect of Some Ayurvedic Intervention to Compare Duration of Analgesia Achieved by Agnikarma and Tab. Diclofenac Sodiumwith Only Tab. Diclofenacsodium in Management of Janusandhishoola with Special Reference to Osteoarthritis.** | **<https://trialsearch.who.int/Trial2.aspx?TrialID=CTRI/2024/12/077594>** | **Excluded** | **Review articles, conference papers** |
| **1187** |  | **A Clinical Trial of Herbal Products in the Management of Knee Osteoarthritis.** | **<https://trialsearch.who.int/Trial2.aspx?TrialID=CTRI/2024/06/069519>** | **Excluded** | **Review articles, conference papers** |
| **1188** |  | **A Clinical Trial to Study the Effect of Virechana (Purgative Treatment ) with Pippali Nagaradi Yog in the Patient of Sthaulya (Obesity).** | **<https://trialsearch.who.int/Trial2.aspx?TrialID=CTRI/2024/07/070597>** | **Excluded** | **Review articles, conference papers** |
| **1189** |  | **A Clinical Trial to Study the Effectiveness of Muscle Energy Technique and Positional Release Technique in Subjects with Piriformis Syndrome.** | **<https://trialsearch.who.int/Trial2.aspx?TrialID=CTRI/2024/04/065496>** | **Excluded** | **Review articles, conference papers** |
| **1190** |  | **A Clinical Trial Will Be Done to Know the Effect of Atasi Taila Matra Basti in the Management of Janusandhigata Vata.** | **<https://trialsearch.who.int/Trial2.aspx?TrialID=CTRI/2024/08/072979>** | **Excluded** | **Review articles, conference papers** |
| **1191** |  | **A Comparative Clinical Trial to Study Effect of Viddha Karma and Agnikarma by Panchadhatu Shalaka in Knee Joint Pain.** | **<https://trialsearch.who.int/Trial2.aspx?TrialID=CTRI/2024/06/068672>** | **Excluded** | **Review articles, conference papers** |
| **1192** |  | **A Comparative Study of Marma Therapy and Upanaha Sweda in the Management of Osteoarthriris of Knee Joint.** | **<https://trialsearch.who.int/Trial2.aspx?TrialID=CTRI/2024/06/069146>** | **Excluded** | **Review articles, conference papers** |
| **1193** |  | **A Randomized Controlled Clinical Study to Edvaluate the Efficicacy of Siravedha and Eranda Taila Orally in Vatakanatak with Special Reference to Reference to Plantar Fasciitis.** | **<https://trialsearch.who.int/Trial2.aspx?TrialID=CTRI/2024/02/062253>** | **Excluded** | **Review articles, conference papers** |
| **1194** |  | **A Study on the Role of Mahasneha and Dashmoola Ksheera Janu Basti in the Management of Sandhivata (Osteo-Arthritis).** | **<https://trialsearch.who.int/Trial2.aspx?TrialID=CTRI/2024/10/075973>** | **Excluded** | **Review articles, conference papers** |
| **1195** |  | **A Study to Compare a Manual Assisted Brief Intervention for Cardiovascular Risk Reduction, with Care as Usual in Patients with Depression.** | **<https://trialsearch.who.int/Trial2.aspx?TrialID=CTRI/2024/02/062214>** | **Excluded** | **Review articles, conference papers** |
| **1196** |  | **A Study to Compare Interathecal Ropivacaine Heavy 0.75% and Bupivacaine Heavy 0.5 % in Orthopaedic Surgeries.** | **<https://trialsearch.who.int/Trial2.aspx?TrialID=CTRI/2024/02/063042>** | **Excluded** | **Review articles, conference papers** |
| **1197** |  | **A Study to Compare Pain Relieving Effect of Intrathecal Morphine and Adductor Canal Block in Patients Undergoing Knee Ligament Repair Surgery.** | **<https://trialsearch.who.int/Trial2.aspx?TrialID=CTRI/2024/08/071945>** | **Excluded** | **Review articles, conference papers** |
| **1198** |  | **A Study to Compare the Effect of Nalbuphine and Fentanyl as an Additive to Hyperbaric Bupivacaine in Spinal Anaesthesia to Compare Intra-Ot Hemodynamic Response.** | **<https://trialsearch.who.int/Trial2.aspx?TrialID=CTRI/2024/11/076695>** | **Excluded** | **Review articles, conference papers** |
| **1199** |  | **A Study to Compare the Efficacy of Gomutra Haritaki Nitya Virechana Along with Udvartana and Triphala Kashaya Nitya Virechana Along with Udvartana in the Management of Sthaulya or Obesity.** | **<https://trialsearch.who.int/Trial2.aspx?TrialID=CTRI/2024/06/068875>** | **Excluded** | **Review articles, conference papers** |
| **1200** |  | **A Study to Evaluate the Effectiveness of a Nurse Administered Special Breathing Exercise on Body Fatigue and Quality of Life.** | **<https://trialsearch.who.int/Trial2.aspx?TrialID=CTRI/2024/02/063342>** | **Excluded** | **Review articles, conference papers** |
| **1201** |  | **A Study to Find the Effect of Two Different Drugs Dexamethason and Fentanyl on Popliteal Block for Leg Surgeries.** | **<https://trialsearch.who.int/Trial2.aspx?TrialID=CTRI/2024/01/061206>** | **Excluded** | **Review articles, conference papers** |
| **1202** |  | **A Study to Know the Efficacy of Janu Dhara and Janu Pichu with Kethakimooladi Taila in Janu Sandhigata Vata (Osteoarthritis of Knee Joint).** | **<https://trialsearch.who.int/Trial2.aspx?TrialID=CTRI/2024/07/069993>** | **Excluded** | **Review articles, conference papers** |
| **1203** |  | **A Study to See Effect of Lateral and Backward Treadmill Walking on Balance and Walking Speed in Chronic Stroke or Paralysis Patient.** | **<https://trialsearch.who.int/Trial2.aspx?TrialID=CTRI/2024/09/074500>** | **Excluded** | **Review articles, conference papers** |
| **1204** |  | **A Study to Test the Effectiveness of Different Patterns of Enema(Matra Basti) in the Management of Janu Sandhigata Vata(Osteoarthritis of Knee).** | **<https://trialsearch.who.int/Trial2.aspx?TrialID=CTRI/2024/04/065729>** | **Excluded** | **Review articles, conference papers** |
| **1205** |  | **A Trial of Blocking Pain around Knee Joint by Alcohol or Phenol Based Intervention.** | **<https://trialsearch.who.int/Trial2.aspx?TrialID=CTRI/2024/07/070562>** | **Excluded** | **Review articles, conference papers** |
| **1206** |  | **Add on Effect of Vyoshadisaktu in Ayurvedic Diet Plan Compared with Standard Diet Plan in the Management of Sthaulya (Obesity) Pertaining to Waist Hip Ratio and Subjective Symptoms of Sthaulya (Obesity).** | **<https://trialsearch.who.int/Trial2.aspx?TrialID=CTRI/2024/03/064186>** | **Excluded** | **Review articles, conference papers** |
| **1207** |  | **Advantage of Local Application of Rohini Taila and Thumari Taila in the Management of Sirajanya Vrana(Venous Ulcer).** | **<https://trialsearch.who.int/Trial2.aspx?TrialID=CTRI/2024/05/067835>** | **Excluded** | **Review articles, conference papers** |
| **1208** |  | **Agnikarma and Medicines in the Pain Management of Knee Osteoarthritis.** | **<https://trialsearch.who.int/Trial2.aspx?TrialID=CTRI/2024/05/067894>** | **Excluded** | **Review articles, conference papers** |
| **1209** |  | **An Observational Study between Intrathecal Hyperbaric Bupivacaine 0.5% 3.3cc with Dexmedatomedine 10 Microgram Versus Hyperbaric Bupivacaine 0.5% 3.3cc with Nalbuphine 1 Mg in Lower Limb Orthopaedic Surgeries.** | **<https://trialsearch.who.int/Trial2.aspx?TrialID=CTRI/2024/11/076686>** | **Excluded** | **Review articles, conference papers** |
| **1210** |  | **Ayurvedic Management of Knee Joint Pain.** | **<https://trialsearch.who.int/Trial2.aspx?TrialID=CTRI/2024/08/072432>** | **Excluded** | **Review articles, conference papers** |
| **1211** |  | **Clinical Study of Pain Relief Oils in Patients with Osteoarthritis.** | **<https://trialsearch.who.int/Trial2.aspx?TrialID=CTRI/2024/10/074997>** | **Excluded** | **Review articles, conference papers** |
| **1212** |  | **Clinical Study to Evaluate the Combined Effects of Vishatindukadi Vati and Amritadi Guggulu in Janusandhigata Vata (Osteoarthritis of Knee Joint).** | **<https://trialsearch.who.int/Trial2.aspx?TrialID=CTRI/2024/11/076197>** | **Excluded** | **Review articles, conference papers** |
| **1213** |  | **Clinical Trial Related to Study the Role of Calcarea Phos 6x Along with Individualized Homoeopathic Medicine in Management of Main Complain of Osteoarthritis of Knee Joint Like Pain, Stiffness and Functional Limitation.** | **<https://trialsearch.who.int/Trial2.aspx?TrialID=CTRI/2024/09/073197>** | **Excluded** | **Review articles, conference papers** |
| **1214** |  | **Clinical Trial to Study the Effectiveness of a Unani Formation, Habb-E-Mafasil, in Primary Knee Osteoarthritis.** | **<https://trialsearch.who.int/Trial2.aspx?TrialID=CTRI/2024/02/062496>** | **Excluded** | **Review articles, conference papers** |
| **1215** |  | **Combined Effect of Castor Oil and Dry Ginger Powder in Patients with Knee Pain- a Case Control Study.** | **<https://trialsearch.who.int/Trial2.aspx?TrialID=CTRI/2024/01/062081>** | **Excluded** | **Review articles, conference papers** |
| **1216** |  | **Comparative Analysis in Patients with Epidural Analgesia Vs Ultrasound Guided Adductor Canal Block for Better Postoperative Pain Relief in Total Knee Replacement Surgery.** | **<https://trialsearch.who.int/Trial2.aspx?TrialID=CTRI/2024/09/074519>** | **Excluded** | **Review articles, conference papers** |
| **1217** |  | **Comparative Evaluation of Post-Operative Analgesic Efficacy of Two Different Drug Volumes of Same Concentration of Local Anesthetic in Patients Undergoing Hip Fracture Surgery under Ultrasound Guided Pericapsular Nerve Group Block around the Hip Joint.** | **<https://trialsearch.who.int/Trial2.aspx?TrialID=CTRI/2024/04/065755>** | **Excluded** | **Review articles, conference papers** |
| **1218** |  | **Compare of Two Blocks in Paediatric Hip Surgery.** | **<https://trialsearch.who.int/Trial2.aspx?TrialID=CTRI/2024/01/061373>** | **Excluded** | **Review articles, conference papers** |
| **1219** |  | **Comparing Buergers Exercise with Manual Lymphatic Drainage for Varicose Veins a Randomized Clinical Trial.** | **<https://trialsearch.who.int/Trial2.aspx?TrialID=CTRI/2024/07/070708>** | **Excluded** | **Review articles, conference papers** |
| **1220** |  | **Comparing Different Nerve Blocks to Improve Patient Positioning During Spinal Anesthesia for Lower Limb Surgeries - a Clinical Trial.** | **<https://trialsearch.who.int/Trial2.aspx?TrialID=CTRI/2024/08/071962>** | **Excluded** | **Review articles, conference papers** |
| **1221** |  | **Comparing Four Types of Pain Relief Techniques after Hip Replacement Surgery Done with Spinal Anesthesia.** | **<https://trialsearch.who.int/Trial2.aspx?TrialID=CTRI/2024/09/074454>** | **Excluded** | **Review articles, conference papers** |
| **1222** |  | **Comparing the Effects of Physiotherapy Treatment Versus Drug Therapy for Constipation in Children with Autism Spectrum Disorder.** | **<https://trialsearch.who.int/Trial2.aspx?TrialID=CTRI/2024/08/072280>** | **Excluded** | **Review articles, conference papers** |
| **1223** |  | **Comparing the Efficacy of Femoral Nerve Block and Fascia Iliaca Block before Positioning for Spinal Anesthesia in Patients with Femur Fractures.** | **<https://trialsearch.who.int/Trial2.aspx?TrialID=CTRI/2024/03/064793>** | **Excluded** | **Review articles, conference papers** |
| **1224** |  | **Comparing Two Physiotherapy Interventions for Low Back Disorders in India: A Randomized Controlled Trial.** | **<https://trialsearch.who.int/Trial2.aspx?TrialID=CTRI/2024/08/072259>** | **Excluded** | **Review articles, conference papers** |
| **1225** |  | **Comparision of Postoperative Analgesia in Patients Undergoing Hip Surgeries with Ultrasound Guided Fascia Iliaca Block Using Different Local Anaesthetics Combination.** | **<https://trialsearch.who.int/Trial2.aspx?TrialID=CTRI/2024/04/066191>** | **Excluded** | **Review articles, conference papers** |
| **1226** |  | **Comparison between Effectiveness of Kinesio Taping and Rigid Taping with Common Use of Cryotherapy in Athletes with Medial Tibial Stress Syndrome.** | **<https://trialsearch.who.int/Trial2.aspx?TrialID=CTRI/2024/08/072383>** | **Excluded** | **Review articles, conference papers** |
| **1227** |  | **Comparison of Effects of Two Physiotherapy Mobilization Interventions as an Adjunct to Institution-Based Exercise Program on Pain, Joint Motion, Strength, Ability to Perform Activities of Daily Living & Quality of Life in Knee Osteoarthritis Patients.** | **<https://trialsearch.who.int/Trial2.aspx?TrialID=CTRI/2024/05/066730>** | **Excluded** | **Review articles, conference papers** |
| **1228** |  | **Comparison of Fentanyl and Magnesium Added to Levobupivacaine Heavy for Spinal Anaesthesia in Lower Limb Surgeries.** | **<https://trialsearch.who.int/Trial2.aspx?TrialID=CTRI/2024/11/076851>** | **Excluded** | **Review articles, conference papers** |
| **1229** |  | **Comparison of Intrathecal Hyperbaric Bupivacaine with Fentanyl Versus Intrathecal Hyperbaric Bupivacaine with Dexmedetomidine Given Sequentially in Lower Limb Orthopaedic Surgeries.** | **<https://trialsearch.who.int/Trial2.aspx?TrialID=CTRI/2024/05/066585>** | **Excluded** | **Review articles, conference papers** |
| **1230** |  | **Comparison of Popliteal and Parasacral Ischial Plane Block.** | **<https://trialsearch.who.int/Trial2.aspx?TrialID=CTRI/2024/02/062409>** | **Excluded** | **Review articles, conference papers** |
| **1231** |  | **Comparison of Sifib and Quadratous Lumborum Block with Peng Block for Postoperative Analgesia in Patients Undergoing Hip Surgery.** | **<https://trialsearch.who.int/Trial2.aspx?TrialID=CTRI/2024/03/063555>** | **Excluded** | **Review articles, conference papers** |
| **1232** |  | **Comparison of the Effectiveness of Two Nerve Block Pain Management Techniques in Patients Undergoing Hip Fracture Repair Surgery.** | **<https://trialsearch.who.int/Trial2.aspx?TrialID=CTRI/2024/05/066952>** | **Excluded** | **Review articles, conference papers** |
| **1233** |  | **Comparison of Therapeutic Effect of Jalaukavcharan and Cupping Therapy in Knee Pain.** | **<https://trialsearch.who.int/Trial2.aspx?TrialID=CTRI/2024/07/070194>** | **Excluded** | **Review articles, conference papers** |
| **1234** |  | **Comparison of Two Different Regional Blocks Technique for Upper Arm Anaesthesia.** | **<https://trialsearch.who.int/Trial2.aspx?TrialID=CTRI/2024/01/061449>** | **Excluded** | **Review articles, conference papers** |
| **1235** |  | **Comparison of Two Ultrasound Guided Nerve Block Techniques for Pain Relief after Childrens Hip Surgery.** | **<https://trialsearch.who.int/Trial2.aspx?TrialID=CTRI/2024/09/074207>** | **Excluded** | **Review articles, conference papers** |
| **1236** |  | **Comparison of Using a Blood Component with and without Activation for Treating Knee Joint Arthritis.** | **<https://trialsearch.who.int/Trial2.aspx?TrialID=CTRI/2024/11/077515>** | **Excluded** | **Review articles, conference papers** |
| **1237** |  | **Does Adding Magnesium Sulphate Prolongs the Pain Relief after Leg Surgeries?** | **<https://trialsearch.who.int/Trial2.aspx?TrialID=CTRI/2024/09/073576>** | **Excluded** | **Review articles, conference papers** |
| **1238** |  | **Does Task-Oriented Circuit Training or Trunk Rehabilitation Work Better for Balance and Gait in Older Adults?** | **<https://trialsearch.who.int/Trial2.aspx?TrialID=CTRI/2024/07/071266>** | **Excluded** | **Review articles, conference papers** |
| **1239** |  | **Ease of Spinal Positioning with the Use Two Different Blocks.** | **<https://trialsearch.who.int/Trial2.aspx?TrialID=CTRI/2024/01/061612>** | **Excluded** | **Review articles, conference papers** |
| **1240** |  | **Effect Hot Foot Immersion and Ice Massage on Hypertension.** | **<https://trialsearch.who.int/Trial2.aspx?TrialID=CTRI/2024/06/069022>** | **Excluded** | **Review articles, conference papers** |
| **1241** |  | **Effect of Cariprazine Versus Aripiprazole on Cardiometabolic Profile in Patients with Schizophrenia Switched from Olanzapine Due to Weight Gain.** | **<https://trialsearch.who.int/Trial2.aspx?TrialID=CTRI/2024/02/062615>** | **Excluded** | **Review articles, conference papers** |
| **1242** |  | **Effect of Cold Douche on Physical Exhaustion in Athletes.** | **<https://trialsearch.who.int/Trial2.aspx?TrialID=CTRI/2024/07/069747>** | **Excluded** | **Review articles, conference papers** |
| **1243** |  | **Effect of Herbal Formulation in Dilated Veins of Legs.** | **<https://trialsearch.who.int/Trial2.aspx?TrialID=CTRI/2024/05/068166>** | **Excluded** | **Review articles, conference papers** |
| **1244** |  | **Effect of Leech Therapy in the Management of Knee Osteoarthritis.** | **<https://trialsearch.who.int/Trial2.aspx?TrialID=CTRI/2024/11/077328>** | **Excluded** | **Review articles, conference papers** |
| **1245** |  | **Effect of Marma Chikitsa in the Pain Management of Janusandhigata Vata (Knee Osteoarthritis).** | **<https://trialsearch.who.int/Trial2.aspx?TrialID=CTRI/2024/01/061136>** | **Excluded** | **Review articles, conference papers** |
| **1246** |  | **Effect of Modified Churna Pinda Sweda Pottali (Herbal Powder Made into Bolus and Applied over the Joint and Temperature Is Maintained Using Modified Equipment) and Conventional Method of Churna Pinda Sweda (Herbal Powder Made into Bolus and Applied over the Joint) in Amavata (Rheumatoid Arthritis).** | **<https://trialsearch.who.int/Trial2.aspx?TrialID=CTRI/2024/10/075929>** | **Excluded** | **Review articles, conference papers** |
| **1247** |  | **Effect of Pippali Rasayana after Koshthashodhana Compared to Kanchnar Guggulu after Koshthashodhana in the Patients of Hypothyroidism ( Medoroga) Who Already Taking Thyroxine.** | **<https://trialsearch.who.int/Trial2.aspx?TrialID=CTRI/2024/09/073946>** | **Excluded** | **Review articles, conference papers** |
| **1248** |  | **Effect of Post-Activation Potentiation on Running Speed and Agility in Cricketers.** | **<https://trialsearch.who.int/Trial2.aspx?TrialID=CTRI/2024/11/077077>** | **Excluded** | **Review articles, conference papers** |
| **1249** |  | **Effect of Saroglitazar in Non Alcoholic Fatty Liver Disease.** | **<https://trialsearch.who.int/Trial2.aspx?TrialID=CTRI/2024/03/063735>** | **Excluded** | **Review articles, conference papers** |
| **1250** |  | **Effect of Soft Tissue Mobilization Vs Massage Gun on Foot and Pelvic Posture- a Randomized Clinical Trail.** | **<https://trialsearch.who.int/Trial2.aspx?TrialID=CTRI/2024/10/075343>** | **Excluded** | **Review articles, conference papers** |
| **1251** |  | **Effect of Turmeric with or without Metformin on Blood Sugar Control in Prediabetic Individuals.** | **<https://trialsearch.who.int/Trial2.aspx?TrialID=CTRI/2024/10/074853>** | **Excluded** | **Review articles, conference papers** |
| **1252** |  | **Effect of Unani Formulations in Knee Ostioarthritis.** | **<https://trialsearch.who.int/Trial2.aspx?TrialID=CTRI/2024/07/070116>** | **Excluded** | **Review articles, conference papers** |
| **1253** |  | **Effect of Yoga and Core Exercises on Lung Capacity in Children with Asthma.** | **<https://trialsearch.who.int/Trial2.aspx?TrialID=CTRI/2024/11/076950>** | **Excluded** | **Review articles, conference papers** |
| **1254** |  | **Effectiveness of Buprenorphine Patch Applied on Skin for Post-Operative Pain in Patients Undergoing Knee Replacement Surgeries.** | **<https://trialsearch.who.int/Trial2.aspx?TrialID=CTRI/2024/05/066648>** | **Excluded** | **Review articles, conference papers** |
| **1255** |  | **Effectiveness of Graded Motor Imagery on Pain, Fear of Movements, and Walking Pattern after Total Hip Replacement: A Randomized Controlled Trial.** | **<https://trialsearch.who.int/Trial2.aspx?TrialID=CTRI/2024/08/073135>** | **Excluded** | **Review articles, conference papers** |
| **1256** |  | **Effectiveness of Structured Systematic Exercise Program on Physical Activity Level for Subjects with Chronic Lumbar Disc Herniation.** | **<https://trialsearch.who.int/Trial2.aspx?TrialID=CTRI/2024/08/071787>** | **Excluded** | **Review articles, conference papers** |
| **1257** |  | **Effects of Blood Flow Restriction Training (Bfrt) Versus Conventional Endurance Training on Pain, Ability to Walk for Long Durations and Function in Patients with Osteoarthritis of the Knee - a Randomized Control Trial.** | **<https://trialsearch.who.int/Trial2.aspx?TrialID=CTRI/2024/05/066785>** | **Excluded** | **Review articles, conference papers** |
| **1258** |  | **Effects of Footwear Modification on Patients with Osteoarthritis of Knee.** | **<https://trialsearch.who.int/Trial2.aspx?TrialID=CTRI/2024/04/066230>** | **Excluded** | **Review articles, conference papers** |
| **1259** |  | **Efficacy of a Turmeric-Boswellia Extract in Sesame Seed Oil in Knee Osteoarthritis.** | **<https://trialsearch.who.int/Trial2.aspx?TrialID=CTRI/2024/05/068001>** | **Excluded** | **Review articles, conference papers** |
| **1260** |  | **Efficacy of Iliopsoas Plane Block Vs Pericapsular Nerve Block in Hip Replacement Surgery.** | **<https://trialsearch.who.int/Trial2.aspx?TrialID=CTRI/2024/02/062713>** | **Excluded** | **Review articles, conference papers** |
| **1261** |  | **Efficacy of Massage with Roghan-I-Laqwa in Neuropathy.** | **<https://trialsearch.who.int/Trial2.aspx?TrialID=CTRI/2024/06/069563>** | **Excluded** | **Review articles, conference papers** |
| **1262** |  | **Efficacy of Mundij Wa Mushil (Concoction and Purgation)Therapy and Nutul (Irrigation) in the Management of Knee Joint Pain.** | **<https://trialsearch.who.int/Trial2.aspx?TrialID=CTRI/2024/06/069350>** | **Excluded** | **Review articles, conference papers** |
| **1263** |  | **Efficacy of Punarnavadi Vati in the Management of Sthaulya with Special Reference to Dyslipidemia in Hypertension.** | **<https://trialsearch.who.int/Trial2.aspx?TrialID=CTRI/2024/09/074251>** | **Excluded** | **Review articles, conference papers** |
| **1264** |  | **Exploring the Impact of External Sensory Integration on Motor Functioning in Children with Attention Deficit Hyperactive Disorder.** | **<https://trialsearch.who.int/Trial2.aspx?TrialID=CTRI/2024/04/065376>** | **Excluded** | **Review articles, conference papers** |
| **1265** |  | **Exploring the Impact of Tyromotion Balance Machine on Patients with Spinal Injury with and without Current.** | **<https://trialsearch.who.int/Trial2.aspx?TrialID=CTRI/2024/03/064055>** | **Excluded** | **Review articles, conference papers** |
| **1266** |  | **Functional Strength Training in Knee Osteoarthritis.** | **<https://trialsearch.who.int/Trial2.aspx?TrialID=CTRI/2024/11/076155>** | **Excluded** | **Review articles, conference papers** |
| **1267** |  | **Gluteus Maximus Activation Technique V/S Illiopsoas Pnf Stretching Technique in Patients with Sacroilliac Joint Dysfunction.** | **<https://trialsearch.who.int/Trial2.aspx?TrialID=CTRI/2024/08/072346>** | **Excluded** | **Review articles, conference papers** |
| **1268** |  | **Improving Balance in Lower Limb Using Visual Biofeedback in Stroke.** | **<https://trialsearch.who.int/Trial2.aspx?TrialID=CTRI/2024/02/062763>** | **Excluded** | **Review articles, conference papers** |
| **1269** |  | **Improving Outcomes in Acute Guillain-Barre Syndrome: A Comparative Study of Proprioceptive Neuromuscular Facilitation Versus Conventional Therapy, Assessing Pain, Fatigue, Symptom Severity, Functioning, and Respiratory Health.** | **<https://trialsearch.who.int/Trial2.aspx?TrialID=CTRI/2024/09/073341>** | **Excluded** | **Review articles, conference papers** |
| **1270** |  | **In Our Study We Will Be Including Who Are Having Lumbopelvic Hip Complex Dysfunction. We Will Be Giving Corrective Exercise Continuum to See the Effect on Throwing Velocity and Upper Extremity Performance.** | **<https://trialsearch.who.int/Trial2.aspx?TrialID=CTRI/2024/06/069404>** | **Excluded** | **Review articles, conference papers** |
| **1271** |  | **Management of Hypothyrodism with Purgation Therapy and Three Types of Enema.** | **<https://trialsearch.who.int/Trial2.aspx?TrialID=CTRI/2024/05/066849>** | **Excluded** | **Review articles, conference papers** |
| **1272** |  | **Management of Osteoarthritis of Knee(Janu Sandhigatavata) by Marma Chiktisa and Agnikarma.** | **<https://trialsearch.who.int/Trial2.aspx?TrialID=CTRI/2024/08/072830>** | **Excluded** | **Review articles, conference papers** |
| **1273** |  | **Management of Osteoarthritis with Ayurveda.** | **<https://trialsearch.who.int/Trial2.aspx?TrialID=CTRI/2024/11/076351>** | **Excluded** | **Review articles, conference papers** |
| **1274** |  | **Nerve Group Block for Positioning of Patients in Spinal Anesthesia in Hip Fracture Surgeries.** | **<https://trialsearch.who.int/Trial2.aspx?TrialID=CTRI/2024/06/068769>** | **Excluded** | **Review articles, conference papers** |
| **1275** |  | **Regaining Balance after a Stroke a Comparison of Exercises.** | **<https://trialsearch.who.int/Trial2.aspx?TrialID=CTRI/2024/08/071779>** | **Excluded** | **Review articles, conference papers** |
| **1276** |  | **Regional Versus General Anaesthesia- Which Has Better Post-Operative Benefits Following Emergency Fracture Surgeries.** | **<https://trialsearch.who.int/Trial2.aspx?TrialID=CTRI/2024/07/070893>** | **Excluded** | **Review articles, conference papers** |
| **1277** |  | **Retro Walking Along with Otago Exercise for Primary Unilateral Tibiofemoral Osteoarthritis of Knee to Reduce Pain, Improve Balance and Functional Mobility.** | **<https://trialsearch.who.int/Trial2.aspx?TrialID=CTRI/2024/04/065323>** | **Excluded** | **Review articles, conference papers** |
| **1278** |  | **Role of Homoeopathic Medicine in Management of Primary Osteoarthritis of Knee Joint.** | **<https://trialsearch.who.int/Trial2.aspx?TrialID=CTRI/2024/07/069711>** | **Excluded** | **Review articles, conference papers** |
| **1279** |  | **Role of Manjishtadi Vikeshika in the Management of Venous Ulcer.** | **<https://trialsearch.who.int/Trial2.aspx?TrialID=CTRI/2024/07/071052>** | **Excluded** | **Review articles, conference papers** |
| **1280** |  | **Role of Tryushanadi Gutika in Knee Osteoarthritis.** | **<https://trialsearch.who.int/Trial2.aspx?TrialID=CTRI/2024/08/072826>** | **Excluded** | **Review articles, conference papers** |
| **1281** |  | **Strengthening of Transversus Abdominis, Lumbar Multifidus and Quadratus Lumborum Muscles on Pain, Strength, and Mobility in Patients with Knee Arthritis.** | **<https://trialsearch.who.int/Trial2.aspx?TrialID=CTRI/2024/05/067319>** | **Excluded** | **Review articles, conference papers** |
| **1282** |  | **Structural Diagnosis and Management Approach and Myofascial Release for Heel Pain in Diabetic Patients.** | **<https://trialsearch.who.int/Trial2.aspx?TrialID=CTRI/2024/11/076311>** | **Excluded** | **Review articles, conference papers** |
| **1283** |  | **Study of Efficacy of Ayurvedic Medicine Krishna(Pippali) Choorna with Yog Basti in Treatment of Vatakaphaj Gridhrasi(Sciatica Leg Pain).** | **<https://trialsearch.who.int/Trial2.aspx?TrialID=CTRI/2024/06/069334>** | **Excluded** | **Review articles, conference papers** |
| **1284** |  | **Study of Herbal Medicine in Obesity.** | **<https://trialsearch.who.int/Trial2.aspx?TrialID=CTRI/2024/04/066105>** | **Excluded** | **Review articles, conference papers** |
| **1285** |  | **Study of Janu Sandhigata Vata with Rasanadya Churna Orally and Trishatiprasarani Tailam Locally.** | **<https://trialsearch.who.int/Trial2.aspx?TrialID=CTRI/2024/11/077093>** | **Excluded** | **Review articles, conference papers** |
| **1286** |  | **Study on Sciatica Diseases with Vishwadi Guggulu Orally and Ashtakatvar Tail Local Application.** | **<https://trialsearch.who.int/Trial2.aspx?TrialID=CTRI/2024/03/064736>** | **Excluded** | **Review articles, conference papers** |
| **1287** |  | **That Sounds Good! Heres Comparing Pilates and Dynamic Stretching: Effects on Hamstring Flexibility in Young Adults.** | **<https://trialsearch.who.int/Trial2.aspx?TrialID=CTRI/2024/10/075614>** | **Excluded** | **Review articles, conference papers** |
| **1288** |  | **The Study Will See How Virechan with Haritakyadi Modak Treats Obesity(Sthaulya).** | **<https://trialsearch.who.int/Trial2.aspx?TrialID=CTRI/2024/06/069518>** | **Excluded** | **Review articles, conference papers** |
| **1289** |  | **To Check That How Much Reliable and Cross Adaptation in Different Culture of the Gujarati Version of the Scale Used to Assess Hip Disability and Osteoarthritis Outcome Score in Patient with Hip Condition.** | **<https://trialsearch.who.int/Trial2.aspx?TrialID=CTRI/2024/10/075921>** | **Excluded** | **Review articles, conference papers** |
| **1290** |  | **To Compare the Effect of Janu Basti and Janu Dhara with Shudha Bala Tail on Two Group of 20 Patients of Janusandhigata Vata W.S.R.To Osteoarthritis of Knee Joint.** | **<https://trialsearch.who.int/Trial2.aspx?TrialID=CTRI/2024/06/068885>** | **Excluded** | **Review articles, conference papers** |
| **1291** |  | **To Compare Two Spinal Anaesthetic Drug with Additive for Lower Abdominal Surgeries.** | **<https://trialsearch.who.int/Trial2.aspx?TrialID=CTRI/2024/08/071933>** | **Excluded** | **Review articles, conference papers** |
| **1292** |  | **To Evaluate the Effect of Two Different Procedures Modified Shringa Application(Cupping Therapy) and Jalauka Avacharana(Leech Therapy) in the Pain Management of Osteo Arthritis of Knee(Janu Sandhi Gata Vata).** | **<https://trialsearch.who.int/Trial2.aspx?TrialID=CTRI/2024/08/072473>** | **Excluded** | **Review articles, conference papers** |
| **1293** |  | **To Find out the Effects of Aroma Oil Massage on the Thyroid Hormone Levels in Hypothyroid Patients.** | **<https://trialsearch.who.int/Trial2.aspx?TrialID=CTRI/2024/02/062772>** | **Excluded** | **Review articles, conference papers** |
| **1294** |  | **To Find the Effect of Pulsed Electromagnetic Field Therapy Versus Matrix Rhythm Therapy in Osteoarthritis of Knee.** | **<https://trialsearch.who.int/Trial2.aspx?TrialID=CTRI/2024/05/067397>** | **Excluded** | **Review articles, conference papers** |
| **1295** |  | **To Improve Balance and Quality of Life in Elderly Stroke Patient to See the Effect of Balance Exercises Along with Rhythmic Stabilization Technique.** | **<https://trialsearch.who.int/Trial2.aspx?TrialID=CTRI/2024/04/064990>** | **Excluded** | **Review articles, conference papers** |
| **1296** |  | **To See and Compare Effect of Shatawhadi Oil and Shwadamshtradi Oil Administered through Rectal Route in Treatment of Knee Joint Osteoarthritis.** | **<https://trialsearch.who.int/Trial2.aspx?TrialID=CTRI/2024/11/076633>** | **Excluded** | **Review articles, conference papers** |
| **1297** |  | **To See the Effect of Medicated Ghee (Manjistadi Ghrita) Prepared from Herbal Drugs in a Form of Dressing Material (Sofra-Tulle/Vikeshika) in the Management of Venous Ulcer.** | **<https://trialsearch.who.int/Trial2.aspx?TrialID=CTRI/2024/08/072232>** | **Excluded** | **Review articles, conference papers** |
| **1298** |  | **To See the Effectiveness of Injection Ropivacaine Hydrochloride (0.5%) Vs Injection Levobupivacaine Hydrochloride (0.5%) in Below Knee Surgeries by Peripheral Nerve Stimulator Guided Popliteal Nerve Block.** | **<https://trialsearch.who.int/Trial2.aspx?TrialID=CTRI/2024/03/064646>** | **Excluded** | **Review articles, conference papers** |
| **1299** |  | **To Study Effect of Regimenal Therapy Wet Cupping in Patients with Sciatica.** | **<https://trialsearch.who.int/Trial2.aspx?TrialID=CTRI/2024/02/062473>** | **Excluded** | **Review articles, conference papers** |
| **1300** |  | **To Study Efficacy of Kanchnar Guggulu Along with Siravedha in Siraj Granthi.** | **<https://trialsearch.who.int/Trial2.aspx?TrialID=CTRI/2024/10/074994>** | **Excluded** | **Review articles, conference papers** |
| **1301** |  | **To Study Management of Guggul and Shallaki in Janu Sandhigatavata(Degenerative Osteoarthritis).** | **<https://trialsearch.who.int/Trial2.aspx?TrialID=CTRI/2024/04/066200>** | **Excluded** | **Review articles, conference papers** |
| **1302** |  | **To Study the Add on Effect of Musta Churna Udvartan with Lekhan Basti in the Management of Sthaulya W.S.R. To Obesity.** | **<https://trialsearch.who.int/Trial2.aspx?TrialID=CTRI/2024/03/063577>** | **Excluded** | **Review articles, conference papers** |
| **1303** |  | **To Study the Effect of Ayurvedic Medicine in the Management of Obesity.** | **<https://trialsearch.who.int/Trial2.aspx?TrialID=CTRI/2024/09/073760>** | **Excluded** | **Review articles, conference papers** |
| **1304** |  | **To Study the Effect of Panchakarma on Obesity.** | **<https://trialsearch.who.int/Trial2.aspx?TrialID=CTRI/2024/05/067629>** | **Excluded** | **Review articles, conference papers** |
| **1305** |  | **Treatment of Sciatica by Cupping Therapy.** | **<https://trialsearch.who.int/Trial2.aspx?TrialID=CTRI/2024/08/071801>** | **Excluded** | **Review articles, conference papers** |
| **1306** |  | **Use of Hyaluronidase as an Additive to Bupivaciane to in Patient Positioning for Spinal and Epidural Procedures.** | **<https://trialsearch.who.int/Trial2.aspx?TrialID=CTRI/2024/09/073495>** | **Excluded** | **Review articles, conference papers** |
| **1307** |  | **Verifying the Rubric Knee Pain in Synthesis Repertory for Understanding Osteoarthritis.** | **<https://trialsearch.who.int/Trial2.aspx?TrialID=CTRI/2024/02/063331>** | **Excluded** | **Review articles, conference papers** |
| **1308** |  | **We Will See the Effect Panchatikta Ghrita Guggul Medicine with Guduchyadi Yog Kashaya in Obese Patient Having Osteoarthritis Symptoms. We Will Also See Effectiveness of Panchatikta Ghrita Guggul Medicine Only in Obese Patient Having Osteoarthritis Symptoms.** | **<https://trialsearch.who.int/Trial2.aspx?TrialID=CTRI/2024/08/072827>** | **Excluded** | **Review articles, conference papers** |
| **1309** | **Cuddigan et al** | **Quadriceps Femoris Strength.** | **<https://doi.org/10.1093/rheumatology/12.2.77.>** | **Excluded** | **Not related to inclusion criteria** |
| **1310** | **Cuellar et al** | **Intravenous Patient-Controlled Analgesia During the Postoperative Period after Traumatologic and Orthopedic Surgery.** |  | **Excluded** | **Not related to the purpose of the article** |
| **1311** |  | **Is Warm‐up Preservation Modulated by Biological Maturation and Sex? Effects on Lower Limbs Performance.** | **<https://doi.org/10.1111/sms.14747.>** | **Excluded** | **Review articles, conference papers** |
| **1312** |  | **Effect of Tomato Consumption on High-Density Lipoprotein Cholesterol Level: A Randomized, Single-Blinded, Controlled Clinical Trial.** | **<https://doi.org/10.2147/DMSO.S48858.>** | **Excluded** | **Review articles, conference papers** |
| **1313** | **Cui et al** | **Efficacy and Safety of a Loading High-Dose Tranexamic Acid Followed by Postoperative Five Doses in Total Hip Arthroplasty: A Randomized Controlled Trial.** | **<https://doi.org/10.7507/1002-1892.201902075.>** | **Excluded** | **Not related to inclusion criteria** |
| **1314** | **Cui et al** | **Trends in Post-Fracture Care for Manitoba, Canada 2000-2014: A Population-Based Analysis.** | **<https://doi.org/10.1002/jbmr.3363.>** | **Excluded** | **Not related to inclusion criteria** |
| **1315** | **Culvenor et al** | **Early Knee Osteoarthritis Is Evident One Year Following Anterior Cruciate Ligament Reconstruction: A Magnetic Resonance Imaging Evaluation.** | **<https://doi.org/10.1002/art.39005.>** | **Excluded** | **Not related to inclusion criteria** |
| **1316** | **Cummings et al** | **Flexibility Development in Sprinters Using Emg Biofeedback and Relaxation Training.** | **<https://doi.org/10.1007/BF00998982.>** | **Excluded** | **Not related to inclusion criteria** |
| **1317** | **Cundy et al** | **Blood Loss in Total Knee Arthroplasty.** | **<https://doi.org/10.1055/s-0036-1592147.>** | **Excluded** | **Not related to inclusion criteria** |
| **1318** | **Cunha et al** | **Postexercise Hypotension after Aquatic Exercise in Older Women with Hypertension: A Randomized Crossover Clinical Trial.** | **<https://doi.org/10.1093/ajh/hpx165.>** | **Excluded** | **Not related to inclusion criteria** |
| **1319** | **Cupisti et al** | **Skeletal Muscle and Nutritional Assessment in Chronic Renal Failure Patients on a Protein-Restricted Diet.** | **<https://doi.org/10.1046/j.0954-6820.2003.01245.x.>** | **Excluded** | **Not related to inclusion criteria** |
| **1320** | **Curi et al** | **The Effects of Core Muscle Fatigue on Lower Limbs and Trunk During Single-Leg Drop Landing: A Comparison between Recreational Runners with and without Dynamic Knee Valgus.** | **<https://doi.org/10.1016/j.knee.2024.07.017.>** | **Excluded** | **Not related to inclusion criteria** |
| **1321** | **Curry et al** | **Disposition and Pharmacodynamics of Dichloroacetate (Dca) and Oxalate Following Oral Dca Doses.** | **<https://doi.org/10.1002/bdd.2510120507.>** | **Excluded** | **Not related to inclusion criteria** |
| **1322** | **Curtin et al** | **Anterior Capsulectomy Versus Repair in Direct Anterior Total Hip Arthroplasty.** | **<https://doi.org/10.1007/s00590-023-03606-x.>** | **Excluded** | **Not related to inclusion criteria** |
| **1323** | **Cusma et al** | **Decreasing Post-Operative Narcotic Usage Following Total Knee Arthroplasty Requires More Than Simple Education: A Blinded Randomized Controlled Trial.** | **<https://doi.org/10.1016/j.ocl.2023.06.001.>** | **Excluded** | **Not related to inclusion criteria** |
| **1324** | **Cuzick et al** | **Long-Term Effects of Adjuvant Tamoxifen Treatment.** |  | **Excluded** | **Not related to the purpose of the article** |
| **1325** |  |  |  | **Excluded** | **Not related to the purpose of the article** |
| **1326** | **Decker et al** | **Gender Differences and the Impact of Fatigue on the Star Excursion Balance Test.** | **<https://doi.org/10.1249/01.mss.0000480440.62001.b8.>** | **Excluded** | **Not related to inclusion criteria** |
| **1327** | **Decker et al** | **Gender Differences in Lower Extremity Kinematics, Kinetics and Energy Absorption During Landing.** | **<https://doi.org/10.1016/s0268-0033(03)00090-1.>** | **Excluded** | **Not related to inclusion criteria** |
| **1328** | **Deckx et al** | **Increasing Oral Doses of Glpg1972 Administered Daily for 29 Days Show a Strong Target Engagement in Patients with Knee and/or Hip Oa.** | **<https://doi.org/10.1002/art.40700.>** | **Excluded** | **Not related to inclusion criteria** |
| **1329** | **Deckx et al** | **A Safety, Tolerability, Pharmacokinetics (Pk) and Pharmacodynamics (Pd) Study with Increasing Oral Doses of Glpg1972 Administered Daily for 29 Days Shows a Strong Biomarker Effect in Patients with Knee and/or Hip Oa.** | **<https://doi.org/10.1136/annrheumdis-2018-eular.3101.>** | **Excluded** | **Not related to inclusion criteria** |
| **1330** | **Decorte et al** | **Absence of Calf Muscle Metabolism Alterations in Active Cystic Fibrosis Adults with Mild to Moderate Lung Disease.** | **<https://doi.org/10.1016/j.jcf.2016.05.010.>** | **Excluded** | **Not related to inclusion criteria** |
| **1331** | **Decorte et al** | **Addition of Prophylactic Compression Garments to Standard Care to Prevent Irreversible Lower Limb Lymphoedema after Gynaeco-Oncological Therapy (Gynolymph): Protocol for a Randomised Controlled Trial Embedded within an Observation Cohort Study.** | **<https://doi.org/10.1136/bmjopen-2024-088851.>** | **Excluded** | **Not related to inclusion criteria** |
| **1332** | **Decoster et al** | **Standing and Supine Hamstring Stretching Are Equally Effective.** |  | **Excluded** | **Review articles, conference papers** |
| **1333** | **Deere et al** | **Assessing the Non-Inferiority of Prosthesis Constructs Used in Hip Replacement Using Data from the National Joint Registry of England, Wales, Northern Ireland and the Isle of Man: A Benchmarking Study.** | **<https://doi.org/10.1136/bmjopen-2018-026685.>** | **Excluded** | **Not related to inclusion criteria** |
| **1334** |  | **Assessing the Non-Inferiority of Prosthesis Constructs Used in Total and Unicondylar Knee Replacements Using Data from the National Joint Registry of England, Wales, Northern Ireland and the Isle of Man: A Benchmarking Study.** | **<https://doi.org/10.1136/bmjopen-2018-026736.>** | **Excluded** | **Review articles, conference papers** |
| **1335** | **Defrate et al** | **In Vivo Acl Mechanics and Risk Factors for Injury.** |  | **Excluded** | **Not related to the purpose of the article** |
| **1336** |  | **In Vivo Knee Motion and Acl Biomechanics During Dynamic Activities.** |  | **Excluded** | **Review articles, conference papers** |
| **1337** | **Degerblad et al** | **Reduced Bone Mineral Density in Adults with Growth Hormone (Gh) Deficiency: Increased Bone Turnover During 12 Months of Gh Substitution Therapy.** | **<https://doi.org/10.1530/eje.0.1330180.>** | **Excluded** | **Not related to inclusion criteria** |
| **1338** | **Degli Uberti et al** | **Prolactin-Releasing Activity of Dermorphin, a New Synthetic Potent Opiate-Like Peptide, in Normal Human Subjects.** | **<https://doi.org/10.1210/jcem-56-5-1032.>** | **Excluded** | **Not related to inclusion criteria** |
| **1339** | **Dehmiyani et al** | **Apathy Exacerbates Postural Control Impairments in Stroke Survivors: The Potential Effects of Cognitive Dual-Task for Improving Postural Control.** | **<https://doi.org/10.1016/j.neuropsychologia.2022.108344.>** | **Excluded** | **Not related to inclusion criteria** |
| **1340** | **Dehn et al** | **Relative Position of the Supra-Acetabular Bone to the Crestal Plane: A Radiological Analysis.** | **<https://doi.org/10.1136/jramc-2019-001251.>** | **Excluded** | **Not related to inclusion criteria** |
| **1341** | **Dejgaard et al** | **Noradrenaline and Isoproterenol Kinetics in Diabetic Patients with and without Autonomic Neuropathy.** | **<https://doi.org/10.1007/BF00873215.>** | **Excluded** | **Not related to inclusion criteria** |
| **1342** | **Del Prete et al** | **Quantification of Surgical Trauma: Comparison of Posterolateral Surgical Approach and Anterior Minimally Invasive (Amis) for Total Hip Arthroplasty Based on Markers of Inflammation (Interleukins). Preliminary Report.** | **<https://doi.org/10.5301/hipint.5000188.>** | **Excluded** | **Not related to inclusion criteria** |
| **1343** | **Delaporta et al** | **Real-World Data on the Use of Luspatercept in Greek Patients with Transfusion Dependent Thalassemia.** | **<https://doi.org/10.1182/blood-2022-167675.>** | **Excluded** | **Not related to inclusion criteria** |
| **1344** | **Delattre et al** | **An Iterative Algorithm for Joint Covariate and Random Effect Selection in Mixed Effects Models.** | **<https://doi.org/10.1515/ijb-2019-0082.>** | **Excluded** | **Not related to inclusion criteria** |
| **1345** | **DelBiondo et al** | **Evaluating Sex Differences in Quadriceps Muscle Fatiguability after Anterior Cruciate Ligament Reconstruction.** |  | **Excluded** | **Not related to the purpose of the article** |
| **1346** | **Delgadillo et al** | **Fatigability of the Knee Extensor Muscles During High-Load Fast and Low-Load Slow Resistance Exercise in Young and Older Adults.** | **<https://doi.org/10.1016/j.exger.2021.111546.>** | **Excluded** | **Not related to inclusion criteria** |
| **1347** | **Delis et al** | **Effect of Intermittent Pneumatic Compression of Foot and Calf on Walking Distance, Hemodynamics, and Quality of Life in Patients with Arterial Claudication: A Prospective Randomized Controlled Study with 1-Year Follow-Up.** | **<https://doi.org/10.1097/01.sla.0000154358.83898.26.>** | **Excluded** | **Not related to inclusion criteria** |
| **1348** | **Delis et al** | **Improving Walking Ability and Ankle Brachial Pressure Indices in Symptomatic Peripheral Vascular Disease with Intermittent Pneumatic Foot Compression: A Prospective Controlled Study with One-Year Follow-Up.** | **<https://doi.org/10.1067/mva.2000.103969.>** | **Excluded** | **Not related to inclusion criteria** |
| **1349** | **DelMastro et al** | **Lower Limb Strength Differentiates between Fallers and Nonfallers with Multiple Sclerosis.** | **<https://doi.org/10.1016/j.apmr.2025.01.001.>** | **Excluded** | **Not related to inclusion criteria** |
| **1350** | **Delrue et al** | **Do Athletes' Responses to Coach Autonomy Support and Control Depend on the Situation and Athletes' Personal Motivation?** | **<https://doi.org/10.1016/j.psychsport.2019.04.003.>** | **Excluded** | **Not related to inclusion criteria** |
| **1351** | **Demange et al** | **Fatigue Meniscal Tears** | **<https://doi.org/10.1007/s00264-015-3010-5.>** | **Excluded** | **Not related to inclusion criteria** |
| **1352** | **DeMarco et al** | **Predictors and Outcomes of Scleroderma Renal Crisis: The High-Dose Versus Low-Dose D-Penicillamine in Early Diffuse Systemic Sclerosis Trial.** | **<https://doi.org/10.1002/art.10589.>** | **Excluded** | **Not related to inclusion criteria** |
| **1353** | **Demchak et al** | **Effects of Functional Electric Stimulation Cycle Ergometry Training on Lower Limb Musculature in Acute Sci Individuals.** |  | **Excluded** | **Not related to inclusion criteria** |
| **1354** | **Demeere et al** | **Cost Minimisation and Cost Effectiveness in Anaesthesia for Total Hip Replacement Surgery, in Belgium? A Study Comparing Three General Anaesthesia Techniques.** |  | **Excluded** | **Not related to the purpose of the article** |
| **1355** | **Demers et al** | **Fatigue, Physical Fitness, and Physical Activity in Patients with Inflammatory Bowel Disease.** |  | **Excluded** | **Not related to the purpose of the article** |
| **1356** | **Demeter et al** | **Who Can Benefit from Virtual Reality to Reduce Experimental Pain? A Crossover Study in Healthy Subjects.** | **<https://doi.org/10.1002/ejp.678.>** | **Excluded** | **Not related to inclusion criteria** |
| **1357** | **DeMik et al** | **Association between Digitally Provided Education and 90-Day Return to Sexual Activity Following Total Knee Arthroplasty: A Randomized Controlled Trial.** | **<https://doi.org/10.1016/j.arth.2023.10.014.>** | **Excluded** | **Not related to inclusion criteria** |
| **1358** | **Demnitz et al** | **Is It All in the Baseline? Trajectories of Chair Stand Performance over 4 Years and Their Association with Grey Matter Structure in Older Adults.** | **<https://doi.org/10.1002/hbm.26346.>** | **Excluded** | **Not related to inclusion criteria** |
| **1359** | **Denburg et al** | **Risk of Fracture in Glomerular Disease: A Population-Based Cohort Study Using the Health Improvement Network.** |  | **Excluded** | **Review articles, conference papers** |
| **1360** | **Denegar et al** | **Responses to Superficial Heating and Cooling Differ in Men and Women with Knee Osteoarthritis.** | **<https://doi.org/10.3109/09593985.2011.586097.>** | **Excluded** | **Not related to inclusion criteria** |
| **1361** | **Denfeld et al** | **Symptom Biology and Accelerated Aging in Heart Failure.** |  | **Excluded** | **Review articles, conference papers** |
| **1362** | **Deng et al** | **Epidemiological Characteristics of Atrial Fibrillation in Southern China: Results from the Guangzhou Heart Study.** | **<https://doi.org/10.1038/s41598-018-35928-w.>** | **Excluded** | **Not related to inclusion criteria** |
| **1363** | **Deng et al** | **An Investigation of Work-Related Musculoskeletal Disorders among Sonographers in a Province of China and Related Influencing Factors.** | **<https://doi.org/10.3760/cma.j.issn.1001-9391.2018.04.011.>** | **Excluded** | **Not related to inclusion criteria** |
| **1364** | **Denmeade et al** | **Transformer: Bipolar Androgen Therapy (Bat) Versus Enzalutamide (E) for Castrationresistant Metastatic Prostate Cancer (Mcrpc).** | **<https://doi.org/10.1200/JCO.2020.38.15_suppl.5517.>** | **Excluded** | **Not related to inclusion criteria** |
| **1365** | **Denti et al** | **Conventional Versus Smart Wireless Navigation in Total Knee Replacement: Similar Outcomes in a Randomized Prospective Study.** | **<https://doi.org/10.1055/s-0038-1660813.>** | **Excluded** | **Not related to inclusion criteria** |
| **1366** | **Denti et al** | **Pharmacokinetics of Isoniazid, Pyrazinamide, and Ethambutol in Newly Diagnosed Pulmonary Tb Patients in Tanzania.** | **<https://doi.org/10.1371/journal.pone.0141002.>** | **Excluded** | **Not related to inclusion criteria** |
| **1367** | **Derakhshan et al** | **Relationship of Hyperinsulinaemia, Insulin Resistance and Β-Cell Dysfunction with Incident Diabetes and Pre-Diabetes: The Tehran Lipid and Glucose Study.** | **<https://doi.org/10.1111/dme.12560.>** | **Excluded** | **Not related to inclusion criteria** |
[truncated: 1,799,978 more chars]
