# Supplementary material for: Gender differences in the impact of fatigue on lower limb landing biomechanics and their association with anterior cruciate ligament (ACL) injuries: A systematic review and meta-analysis
Source: PLoS One. 2025 May 7;20(5):e0321925. doi: 10.1371/journal.pone.0321925 (PMC12058186; doi:10.1371/journal.pone.0321925)
Supplement: Attachment — Submitted filename: PONE-D-24-38651_reviewer-compressed.pdf [file pone.0321925.s010.pdf]

PLOS ONE

Gender Differences in the Impact of Fatigue on Lower Limb Landing Biomechanics and Their Association with Anterior Cruciate Ligament (ACL) Injuries: A Sysstematic Review and Meta-Analysis

--Manuscript Draft--

**Manuscript Number:**	PONE-D-24-38651
**Article Type:**	Research Article
**Full Title:**	Gender Differences in the Impact of Fatigue on Lower Limb Landing Biomechanics and Their Association with Anterior Cruciate Ligament (ACL) Injuries: A Sysstematic Review and Meta-Analysis
**Short Title:**	Fatigue and Gender Differences in Lower Limb Biomechanics & ACL Injury: A Systematic Review and Meta-Analysis
**Corresponding Author:**	Bojin Cheng<br>Guangzhou Sport University<br>广州市, 广东省 CHINA
**Keywords:**	Fatigue; Anterior Cruciate Ligament; Sex Characteristics
**Abstract:**	Background: This meta-analysis examines the impact of neuromuscular fatigue on gender differences in lower limb landing biomechanics and its correlation with ACL injury risk. Methods: A comprehensive search was conducted in PubMed, Scopus, Web of Science, Embase, and the Cochrane Library up to March 2024 Results: Fourteen studies were included, averaging a quality score of 6.79; nine were high quality. Key findings: males showed a significant increase in knee flexion angle at initial contact (effect size -1.23), but females did not (-0.25). Both genders had significant changes in hip external rotation (males: 1.35, females: 1.20). Ankle peak dorsiflexion angle increased (-1.69) with no gender differences. Peak Knee extension moment increased in males (0.76) and females (0.48) with an overall effect size of 0.64, but no change in peak abduction moment. Peak Hip extension moment was significant in males (0.58) and overall (0.51), with no changes in internal rotation or adduction moments. Peak vertical ground reaction force showed no significant changes for either gender. Conclusions: fatigue alters knee biomechanics in males, raising ACL injury risk, and both genders show increased hip and ankle loads post-fatigue. These results suggest the need for gender-specific fatigue management strategies to mitigate ACL injury risk and call for further research into prevention mechanisms.
**Order of Authors:**	Chengxun Liu
	Wuwen Peng
	Wenhao Qu
	Zhiyong Zhang
	Jiaxin He
	Xiaoping Duan
	Bojin Cheng
**Additional Information:**	
**Question**	**Response**
**Financial Disclosure**<br><br>Enter a financial disclosure statement that describes the sources of funding for the work included in this submission. Review the submission guidelines for detailed requirements. View published research	The author(s) received no specific funding for this work.

Powered by Editorial Manager® and ProduXion Manager® from Aries Systems Corporation

articles from *PLOS ONE* for specific examples.<br><br>This statement is required for submission and **will appear in the published article** if the submission is accepted. Please make sure it is accurate.<br><br>**Funded studies**<br>Enter a statement with the following details:<br>• Initials of the authors who received each award<br>• Grant numbers awarded to each author<br>• The full name of each funder<br>• URL of each funder website<br>• Did the sponsors or funders play any role in the study design, data collection and analysis, decision to publish, or preparation of the manuscript?<br><br>Did you receive funding for this work?	
**Competing Interests**<br><br>Use the instructions below to enter a competing interest statement for this submission. On behalf of all authors, disclose any competing interests that could be perceived to bias this work—acknowledging all financial support and any other relevant financial or non-financial competing interests.<br><br>This statement is required for submission and **will appear in the published article** if the submission is accepted. Please make sure it is accurate and that any funding sources listed in your Funding Information later in the submission form are also declared in your Financial Disclosure statement.<br><br>View published research articles from *PLOS ONE* for specific examples.	The authors have declared that no competing interests exist.

**NO authors have competing interests**<br><br>Enter: *The authors have declared that no competing interests exist.*<br><br>**Authors with competing interests**<br><br>Enter competing interest details beginning with this statement:<br><br>*I have read the journal's policy and the authors of this manuscript have the following competing interests: [insert competing interests here]*<br><br>* typeset	
**Ethics Statement**<br><br>Enter an ethics statement for this submission. This statement is required if the study involved:<br><br>• Human participants<br>• Human specimens or tissue<br>• Vertebrate animals or cephalopods<br>• Vertebrate embryos or tissues<br>• Field research<br><br>Write "N/A" if the submission does not require an ethics statement.<br><br>General guidance is provided below. Consult the submission guidelines for detailed instructions. **Make sure that all information entered here is included in the Methods section of the manuscript.**	N/A”

**Format for specific study types**<br><br>**Human Subject Research (involving human participants and/or tissue)**<br>• Give the name of the institutional review board or ethics committee that approved the study<br>• Include the approval number and/or a statement indicating approval of this research<br>• Indicate the form of consent obtained (written/oral) or the reason that consent was not obtained (e.g. the data were analyzed anonymously)<br><br>**Animal Research (involving vertebrate animals, embryos or tissues)**<br>• Provide the name of the Institutional Animal Care and Use Committee (IACUC) or other relevant ethics board that reviewed the study protocol, and indicate whether they approved this research or granted a formal waiver of ethical approval<br>• Include an approval number if one was obtained<br>• If the study involved *non-human primates*, add *additional details* about animal welfare and steps taken to ameliorate suffering<br>• If anesthesia, euthanasia, or any kind of animal sacrifice is part of the study, include briefly which substances and/or methods were applied<br><br>**Field Research**<br><br>Include the following details if this study involves the collection of plant, animal, or other materials from a natural setting:<br>• Field permit number<br>• Name of the institution or relevant body that granted permission	
**Data Availability**<br><br>Authors are required to make all data underlying the findings described fully available, without restriction, and from the time of publication. PLOS allows rare exceptions to address legal and ethical concerns. See the PLOS Data Policy and FAQ for detailed information.	Yes - all data are fully available without restriction

Powered by Editorial Manager® and ProduXion Manager® from Aries Systems Corporation

A Data Availability Statement describing where the data can be found is required at submission. Your answers to this question constitute the Data Availability Statement and **will be published in the article**, if accepted.<br><br>**Important:** Stating 'data available on request from the author' is not sufficient. If your data are only available upon request, select 'No' for the first question and explain your exceptional situation in the text box.<br><br>Do the authors confirm that all data underlying the findings described in their manuscript are fully available without restriction?	
**Describe where the data may be found in full sentences. If you are copying our sample text, replace any instances of XXX with the appropriate details.**<br><br>• If the data are **held or will be held in a public repository**, include URLs, accession numbers or DOIs. If this information will only be available after acceptance, indicate this by ticking the box below. For example: *All XXX files are available from the XXX database (accession number(s) XXX, XXX.*).<br>• If the data are all contained **within the manuscript and/or Supporting Information files**, enter the following: *All relevant data are within the manuscript and its Supporting Information files.*<br>• If neither of these applies but you are able to provide **details of access elsewhere**, with or without limitations, please do so. For example:<br><br>*Data cannot be shared publicly because of [XXX]. Data are available from the XXX Institutional Data Access / Ethics Committee (contact via XXX) for researchers who meet the criteria for access to confidential data.*<br><br>*The data underlying the results presented in the study are available from (include the name of the third party*	All relevant data are within the manuscript and its Supporting Information files.

and contact information or URL).<br>• This text is appropriate if the data are owned by a third party and authors do not have permission to share the data.<br><br>* typeset	
Additional data availability information:	Tick here if the URLs/accession numbers/DOIs will be available only after acceptance of the manuscript for publication so that we can ensure their inclusion before publication.; Tick here if your circumstances are not covered by the questions above and you need the journal's help to make your data available.

Powered by Editorial Manager® and ProduXion Manager® from Aries Systems Corporation

Manuscript *手稿

Click here to access/download;Manuscript;renamed_e0408.docx

Gender Differences in the Impact of Fatigue on Lower Limb Landing Biomechanics and Their Association with Anterior Cruciate Ligament (ACL) Injuries: A Systematic Review and Meta-Analysis

Chengxun Liu[1]¶，Wuwen Peng[1]¶，Wenhao Qu[1]¶，Zhiyong Zhang[2]，Jiaxin He[3*]，Xiaoping Duan[3*]，Bojin Cheng[3] *.

[1]Digitalized Strength and Conditioning Training Laboratory, Guangzhou Sport University, Guangzhou, China.

[2] School of Athletic Training, Guangzhou Sport University, Guangzhou, China.

[3] Guangdong Provincial Key Laboratory of Human Sports Performance Science, Guangzhou Sport University, Guangzhou, China.

[4]guangdong provincial institute of sports science, Guangzhou Sport University, Guangzhou, China.

*** Corresponding author：**

email:574401513@qq.com(J.H)

duanxpll@163.com(X.D);

bobo791017@163.com(B.C)

¶ These authors contributed equally to this work.

Abstract

Background: This meta-analysis examines the impact of neuromuscular fatigue on gender differences in lower limb landing biomechanics and its correlation with ACL injury risk. Methods: A comprehensive search was conducted in PubMed, Scopus, Web of Science, Embase, and the Cochrane Library up to March 2024 Results: Fourteen studies were included, averaging a quality score of 6.79; nine were high quality. Key findings: males showed a significant increase in knee flexion angle at initial contact (effect size -1.23), but females did not (-0.25). Both genders had significant changes in hip external rotation (males: 1.35, females: 1.20). Ankle peak dorsiflexion angle increased (-1.69) with no gender differences. Peak Knee extension moment increased in males (0.76) and females (0.48) with an overall effect size of 0.64, but no change in peak abduction moment. Peak Hip extension moment was significant in males (0.58) and overall (0.51), with no changes in internal rotation or adduction moments. Peak vertical ground reaction force showed no significant changes for either gender. Conclusions: fatigue alters knee biomechanics in males, raising ACL injury risk, and both genders show increased hip and ankle loads post-fatigue. These results suggest the need for gender-specific fatigue management strategies to mitigate ACL injury risk and call for further research into prevention mechanisms.

Keywords: **Fatigue; Anterior Cruciate Ligament; Sex Characteristics;**

Introduction

Anterior Cruciate Ligament (ACL) tears are among the most common non-contact injuries in many sports(1, 2), especially those involving rapid direction changes, deceleration, jumping, and landing(3-5). ACL injuries typically occur at the knee joint due to improper landing techniques and movement patterns, which can place excessive shear forces and stress on the ACL(6). Changes in these biomechanical characteristics are closely linked to ACL injuries, particularly increases in knee abduction angles, knee abduction moments, and hip internal rotation moments during landing, which are key factors in non-contact ACL injuries(7).

Multiple factors cause biomechanical changes in the lower limbs during landing(8-10), with fatigue being a critical one. Fatigue alters the biomechanical characteristics of the lower limbs during landing, increasing the risk of ACL injuries. Fatigue, as a complex physiological mechanism, occurs at both central and peripheral levels, significantly affecting neuromuscular pathways(11). Under fatigue, reduced neural feedback efficiency, prolonged muscle reaction time, and proprioceptive dysfunction slow down muscle strength generation and contraction speed(12, 13). These physiological changes not only affect kinematic performance(14, 15) but also weaken joint stability(16, 17), significantly impairing dynamic balance control(18). Research further supports this, showing that vertical ground reaction forces (VGRF) significantly increase under fatigue, leading to higher knee joint loads and thus a greater risk of ACL injuries(19). Moreover, fatigue reduces knee flexion angles, increases knee abduction angles, and heightens hip internal rotation angles, significantly raising stress on the knee joint and ACL(20, 21). Epidemiological studies also show that most sports injuries occur in the latter stages of activities or competitions, further

underscoring the importance of fatigue in sports injuries(22). In summary, fatigue increases the risk of ACL injuries by altering lower limb biomechanics through changes in muscle and nervous system functions.

Studies have qualitatively shown gender differences in lower limb landing mechanics. For instance, Hewett et al. found that females have smaller knee flexion angles and larger knee valgus angles during landing, leading to a significantly higher ACL injury rate than males(4). Additionally, insufficient hip flexion and increased internal rotation can indirectly increase knee joint load. Ford et al. found that female athletes have larger hip internal rotation angles during jumping and landing, making them more prone to ACL injuries(23). However, findings on gender differences in lower limb landing mechanics under fatigue conditions are inconsistent. Fagenbaum, R. et al. used an isokinetic machine for fatigue protocols and compared 6 male and 8 female basketball players. They found that females had greater knee bending accelerations during landing under fatigue. Unlike male college basketball players, female college basketball players did not show changes in knee joint muscle coordination characteristics, possibly making them more susceptible to ACL injuries during jump landings(24). In contrast, Susan L. Rozzi et al. found that although fatigue decreased joint proprioception (especially in extension) and altered muscle activation patterns, including delayed contraction times and increased specific muscle contraction areas, there were no significant gender differences(25). These discrepancies indicate the need for more systematic research to explore the impact of fatigue on gender differences in lower limb landing biomechanics.

Therefore, this study aims to systematically review and synthesize existing research through a meta-analysis to explore the specific impact of fatigue on gender differences in lower limb landing biomechanics. This will provide a deeper understanding and scientific basis for preventing ACL injuries, helping to identify high-risk populations and offering references for developing more personalized prevention and rehabilitation programs, thereby effectively reducing the incidence of ACL injuries.

Method

This article is prepared according to the PRISMA project guidelines and the recommendations by Moher et al. for systematic reviews and meta-analyses(26). The protocol has been registered on PROSPERO (ID: CRD42024545104).

Search Strategy

As of March 2024, a comprehensive search was conducted in the electronic databases of PubMed, Scopus, Web of Science, Embase, and Cochrane Library. Boolean operators "AND" and "OR" were used with the following keywords (using Pubmed as an example)(Fig 1)

Figure 1. PubMed Literature Selection Strategy.

The systematic search was carried out by three independent researchers (C.L., W.P., W.Q.). They initially screened the articles based on titles and abstracts. If the information was ambiguous, they retrieved the full text to assess relevance. After the screening process, the researchers compared their findings and resolved disagreements by jointly reassessing the studies that met the eligibility criteria.

Inclusion and Exclusion Criteria

The literature screening criteria for this meta-analysis were established according to the PICOS(27)format (Participants, Interventions, Comparisons, Outcomes, and Study Design) used in evidence-based medicine. The inclusion criteria are: (1) studies involving both male and female participants;

(2) studies utilizing a pre-post self-controlled experimental design; (3) studies implementing at least one protocol designed to induce fatigue; and (4) studies with outcomes that include kinematic, kinetic, or electromyographic analyses relevant to sports biomechanics. The exclusion criteria are: (1) studies not employing a self-controlled experimental design; (2) studies with inaccessible data; (3) studies not utilizing three-dimensional motion analysis systems or force platforms for kinematic and kinetic measurements; (4) conference papers, review articles, and opinion pieces; and (5) studies where the full text is not available.

Data Extraction

All references were managed using EndNote X9 software, and duplicates were removed. Two authors (C.L and L.X) independently extracted descriptive information from all included studies, including publication details (author, year), participant demographics (age, gender), sample size, participant characteristics, fatigue intervention protocols, implementation methods, and biomechanical outcomes (kinematics, kinetics) (Table 3). Biomechanical data necessary for calculating effect sizes (mean and standard deviation) were extracted, and corresponding authors were contacted for additional data if needed. Any discrepancies in the data extracted by C.L and L.X were confirmed by a third researcher, W.P.

Risk of Bias Assessment

The quality of the studies was assessed using a modified version of the Strengthening the Reporting of Observational Studies in Epidemiology (STROBE) guidelines for evaluating observational studies(28) . Each included study was scored based on 10 specific criteria derived from items 5, 6, 7, 8, 9, 12, 13, 14, and 15 of the original checklist (Table 1). The overall quality score was the sum of these criteria, with a maximum score of 10 points. Studies scoring 7 points or higher were considered high quality. This scoring

threshold was determined by author consensus and based on previously published modifications of the STROBE guidelines(29).

Table 1. Checklist of 10 criteria based on an adapted version of the STROBE guidelines for assessing the quality of observational studies used in this analysis

Criteria	S<br>core
Materials and Methods	
1. Describe the study setting or participating locations	1
2. Describe the period of recruitment/follow-up/data collection	1
3. Describe the sources/method of selection of participants	1
4. Give the inclusion and exclusion criteria	1
Data Sources/Measurement	
5. Clearly describe all outcome measures:	1
6. Describe measurement/testing procedures of each outcome measure	1
7. Describe comparability of assessment between groups/time-points	1
8. Explain number of participants with missing data and how this was addressed	1
9. Report number of individuals in each group/time-point (and if applicable any reasons for non-participation in ≥1 outcome measure)	1
10. Report means for each fatigue/sex grouping with a measure of variance (e.g., 95%	1

c

CI or SD)	
Total Score	1<br>0

Data Analysis

To ensure objectivity and robustness, Review Manager 5.3 software (The Nordic Cochrane Centre, Copenhagen, Denmark) was utilized for meta-analyses and subgroup analyses of data from at least two studies reporting on the same variable for both males and females. When measurement units were consistent (e.g., angles), the weighted mean difference (WMD) with a 95% confidence interval was used to assess the overall effect size. If the measurement units differed, the standardized mean difference (SMD) was applied(30). Heterogeneity among studies was evaluated using the I^2 statistic, categorized as low (25-50%), moderate (50-75%), and high (greater than 75%)(31). A fixed-effects model was used when heterogeneity was less than 25%, and a random-effects model was applied when it exceeded 25%(32). A significance level of 0.05 was employed for all statistical tests.

Meta-analyses were conducted on 18 variables related to lower limb landing mechanics to assess changes before and after fatigue intervention. These variables included:

1.Peak flexion angles of the knee, hip, and ankle joints.

2.Initial contact flexion angles of the hip and knee joints.

3.Peak abduction angles of the knee and hip joints and their angles at initial contact.

4.Hip external rotation angle at initial contact and peak internal rotation angle.

5.Peak internal adduction, internal rotation, extension, and flexion moments of the hip.

6.Peak extension and abduction moments of the knee joint.

7.Peak vertical ground reaction force (PVGRF).

To enhance the study's comparability and reliability, data included both males and females, and subgroup analyses were manually classified by gender. Each subgroup required a sufficient number of studies; subgroups with fewer than two studies were excluded from the final analysis.

Results

Through electronic database searches, 11,057 records were identified. After the initial screening, 2,245 duplicate records were removed. Titles and abstracts of the remaining 8,812 records were screened, resulting in the exclusion of 8,657 records. A detailed evaluation of the 155 remaining full-text articles led to the exclusion of 136 based on study content and quality: 22 did not involve fatigue, 98 did not include both genders, 2 were non-English articles, 3 used 2D analysis methods, and 13 did not meet study requirements. After rigorous screening, 17 studies were included in the qualitative synthesis, and 14 were used in the quantitative synthesis (Fig. 2).

Figure 2. PRISMA flow chart for inclusion and exclusion of studies

Risk of Bias in Included Articles

Table 2 details the quality scores of the included studies. Each column corresponds to a specific quality assessment criterion (N1 to N10), with each criterion scored out of 1 point, totaling a maximum of 10 points per study. The table lists the author names, their scores for each criterion, and the total score. Most studies scored between 5 and 8 out of 10, with an average score of 6.79. Nine out of the 14 studies were identified as "high quality," scoring 7 or higher.

Table2.Quality scores of studies included in the review

Study	1	2	3	4	5	6	7	8	9	N 10	Overall
Abbey C. Thomas et al.(33)										1	6
Anne Benjaminse et al.(34)										1	7
Danielle M. Brazenet al.(35)										1	8
DAVID R. BELL et al.(36)										1	8
Dominic Gehring et al.(37)										1	6
Evangelos Pappas et al.(38)										1	5

Kristı´n Briem et al.(39)											1	6
Lessi G .C et al.(40)											1	7
Marijeanne Liederbach et al(41)											1	7
Michael P. Smith et al.(42)											1	7
Ram Haddas et al. (43)											1	8
Scott G. M et al.(44)											1	7
Thomas W. Kernozek et al.(45)											1	8
Zhang Qiang et al.(46)											1	5

Characteristics of Included Studies

This meta-analysis included 14 studies involving different populations: six studies with healthy individuals, three with amateur athletes, two with collegiate athletes, and three with registered athletes, team athletes, and young individuals with a history of recurrent low back pain but currently asymptomatic. These studies encompassed a total of 496 participants, with 259 males and 237 females. Six studies used

single-leg landing, while eight used double-leg landing. Fatigue induction protocols were categorized into isolated muscle group fatigue and whole-body fatigue. The included studies provided extensive kinematic and kinetic results, covering peak flexion angles of the knee, hip, and ankle joints; initial contact flexion angles of the hip and knee joints; peak abduction angles of the knee and hip joints and their angles at initial contact; hip external rotation angle at initial contact and peak internal rotation angle; as well as peak internal adduction and internal rotation moments of the hip; peak extension and abduction moments of the knee joint; and peak vertical ground reaction force (PVGRF) (Table 3)

Table3. Demographic information from included studies

Study	Training state	Male individuals（n）	Female individuals（n）	Age （years）	Fatigue protocol	Landing Test	outcomes
Abbey C. Thomas et al. 2010(33)	Healthy people	13	12	18-22	1、Subjects performed five alternating MVCCs, recording baseline peak torque 2、MVCC continued until torque dropped below 50% of baseline three times.A 20-second rest followed each drop. 3、This cycle repeated until torque fell below 50% during the initial five reps,	Forward single-leg jump landing.	IC: hip/knee flexion angle; knee/hip IR angle；peak hip/knee flexion angle;peak hip/knee abduction angle;peak knee /hip IR angle; peak hip/knee flexion moment;peak hip/knee adduction

c

						indicating fatigue		moment;peak hip IR moment;peak knee ER moment;
Anne Benjaminse et al.2008(34)	healthy and physically active subjects	15	15	0	18-3	1、Participants warmed up for 3 minutes, then ran at 5-8 mph with a 0% incline for another 3 minutes.<br>2、The incline increased by 2.5% every 2 minutes until exhaustion.<br>3、Verbal encouragement was provided throughout.<br>4、Average speeds were 6.3 ± 0.62 mph	Stop-jump task consisted of a single-leg	IC: knee flexion angle/ hip ER angle/hip adduction angle/Knee valgus–varus angles;peak knee valgus–varus；peak knee flexion angle;<br>peak knee abduction angle;peak hip adduction angle;peak hip IR angle;

c

					for males and 5.8 ± 0.43 mph for females.		
Danielle M. Brazenet al.2010(35)	Healthy people	12	12	19.5 ±3.8	Repeat the following agility training circuit five times:<br>1、Ladder Agility Drills.<br>2、Side-to-Side Jumps.<br>3、Mini Trampoline Jumps.<br>4、Mini Hurdle Jumps.<br>5、V-shaped Vertical Jumps.	Single-leg drop landing from a 0.36m height.	Peake VGRF
DAVID R. BELL et al.2016(36)	Healthy people	20	20	18-23	1、Participants start at the first cone, spaced 4.05 meters apart.<br>2、After completing the circuit, they perform a 30-second wall sit with knees	Double-leg drop jump from a 30cm height.	Peak VGRF

					bent at 90 degrees and back against the wall.<br>3、Then, they execute 10 rapid double-leg vertical jumps.<br>4、Finally, they hold a 30-second plank on a 5mm yoga mat		
Dominic Gehring et al. 2009(37)	Healthy people	13	13	24±2.4	1、Participants used the BERI MED leg press machine and flexed/extended their knees to a 90% angle.<br>2、Leg contractions were performed at 50% of the maximum load, with a maximum of 1 repetition.<br>3、The experiment continued until	Double-leg drop landing from a 52cm height.	IC knee flexion angle;peak knee flexion angle; IC knee abduction angle;peak knee abduction angle;Knee flexion velocity 0–50 ms (°/s) ； Knee flexion velocity 50–100 ms (°/s)<br>Peak VGRF
c					16		

					participants couldn't complete the load.		
Evange<br>los Pappas et<br>al.2009(38)	Recreation<br>al athletes	14	15	20-4<br>0	1、Jumping over obstacles:<br>Participants cleared a series of five<br>consecutive obstacles measuring 5-7<br>centimeters in height.<br>2、Repetitions: The jumping sequence<br>was repeated 20 times.<br>3、Total jumps: The participants<br>completed a total of 100 jumps	Double-l<br>eg drop<br>landing from<br>a 40cm height.	Peak knee flexion angle
Kristı´<br>n Briem et<br>al.2017(39)	Registered<br>athletes	68	48	mea<br>n age,10.4	1、Starting position: Participants<br>started at both ends, with bumper bars<br>positioned at a distance of 1.5 times their	Double-l<br>eg drop jump<br>from a 25cm	IC knee flexion angle;peak knee<br>flexion angle;<br>Peak VGRF

c

					leg length.<br>2、Lateral push and slide: Participants laterally pushed off from one bumper bar and slid to the opposite side of the skateboard.<br>3、 Duration: The activity was repeated for 5 minutes.<br>4、Endurance: Participants continued the activity until they could no longer sustain it	height.	
Lessi G .C et al.2017(40)	Recreation al athletes	20	20	18-3 0	1、10 sets of bilateral squats (90° knee flexion).<br>2、2 sets of bilateral maximum effort	Single-le g drop vertical jump	IC：Knee sagittal plane；Knee frontal plane；Hip sagittal plane；Hip frontal plane；
c							

Marije anne Liederbach et al.2014(41)	Team sport athletes	20	20	20-2 2	1、Climbing 50 steps on a 30cm box. 2、Performing 15 maximum effort single-leg vertical jumps vertical jumps and 20 steps (31cm high stairs). Participants use their dominant leg for stepping	Single-leg drop landing from a 30cm height. from a 31cm height	Peak: Knee sagittal plane; Knee frontal plane; Hip sagittal plane; Hip frontal plane; Flexion (+); extension (-); abduction (+); adduction (-) Peak knee abduction angle; Knee abduction angle at IC; Peak knee abduction moment; Peak hip adduction angle; Hip adduction angle at IC; Peak hip adduction moment; Peak hip IR angle; Hip IR angle at IC; Peak hip IR moment; Knee

c

							flexion angle at IC；Peak knee flexion angle；Peak knee flexion moment
Michael P. Smith et al.2009(42)	healthy and physically active subjects	12	14	18-35	Participants performed knee flexion to 60 degrees. They repeated 15-second maximum isometric contractions followed by 5 seconds of relaxation until reaching fatigue (below 50% MVC)	Double-leg drop landing from a 50cm height.	Peak VGRF；IC knee flexion angle;
Ram Haddas et al. 2015(43)	Young adults without current symptoms but with recurrent LBP	17	15	21.65±2.30	Participants fatigued by performing submaximal free weight squats using 15% of their body weight until task failure.	Double-leg drop vertical jump from a 30cm height	Knee sagittal-plane angle at IC;Knee frontal-plane angle at IC;Knee transverse-plane (rotation) angle at IC; Peak sagittal-plane knee angle;Peakfrontal-plane knee

c

							angle;Peak knee transverse-plane (rotation) angle; Peak VGRF;Peak：Knee sagittal-plane moment/Knee frontal-plane moment/Ankle sagittal-plane moment/Ankle frontal-plane moment The convention for positive angles is flexion, abduction, and external rotation.
Scott G. M et al.2007(44)	College athlete	10	10	20.7 ±1.3	Participants performed explosive jumps on a 20cm high step, covering a 6-meter distance with a change in direction.	Double-leg drop vertical jump	IC Joint：Hip flexion/Hip abduction/Hip ER/Knee flexion/Knee adduction/Knee ER
c							

					They landed in a deep knee flexion position and quickly rebounded to the next jump. The process was repeated as many times as possible within 4 minutes.	from a 50cm height	/Ankle dorsiflexion/Ankle IR;Peak Joint:Hip flexion/Hip adductionn/Hip IR/Knee flexion/Knee abduction/Knee IR /Ankle dorsiflexion/Ankle ER; Peak Moment:Hip flexion/Hip adduction/Hip IR/Knee flexion/Knee abduction/Knee IR/Ankle dorsiflexion/Ankle ER
Thomas W. Kernozek et al.2008(45)	Recreational athletes	16	14	23.0±0.9	Participants performed as many repetitions as possible with a set weight, controlling the speed of each phase. Resting 90 seconds between sets, they continued	Single-leg drop landing from a 50cm height.	IC、peak joint：Hip abductor/Hip flexion/Knee flexion;Peakjoint Knee abduction/Ankle dorsiflexion;
c							

					until unable to lift the weight.		Peak:Knee abduction moment/Knee extension moment;PeakVGRF
Zhang Qiang et al.2021(46)	College athlete	9	9	21.3±1.0	Participants stood at the center of a 6-meter diameter circular area with six evenly spaced lights around it. A controller randomly lit a series of lights, and participants had to quickly move to touch and turn off the active light, triggering the illumination of the next light.	Double-leg drop landing from a 40cm height.	IC:Ankle plantarflexion angle/Knee flexion angle/Knee add/abduction angle/Hip flexion angle;<br>Peak:ankle dorsiflexion angle/knee flexion angle/Peak knee abduction angle/hip flexion angle/hip abduction angle;<br>Peak Moment:ankle plantarflexion/knee extension/knee

			**adduction/hip extension/hip abduction**

IC: Initial contact; IR: internal rotation; ER: external rotation; VGRF: Peak vertical ground reaction force

Kinematic Data of Landing Biomechanics

This meta-analysis included a total of 14 studies that examined the kinematic data of the knee, hip, and ankle joints, investigating the effects of fatigue and gender on these parameters. The kinematic data for each joint were represented by three-dimensional joint angles (in degrees). The specific parameters analyzed are detailed in Table 4.

Table 4.Summary of a meta-analysis evaluating the effects of fatigue before and after (including gender subgroups) and on major biomechanical variables of lower limb landing mechanics

Table 4.Summary of a meta-analysis evaluating the effects of fatigue before and after (including gender subgroups) and on major biomechanical variables of lower limb landing mechanics

Kinematic Variables	Sub-roup	No. of Studies		Subtotal I^2	Subtotal Z	Subtotal P	Subtotal (95% CI)	Total I^2	Total Z	Total P	Total (95% CI)	Effect Measure/Analysis Model
Knee flexion at IC	Male	10	200	0%	3.9	<0.001*	-1.23 [-1.85, -0.61]	16%	2.55	0.01*	-0.72 [-1.28, -0.17]	MD/Random
	Female	10	178	32%	0.51	0.61	-0.25 [-1.22, 0.72]					
Peak knee flexion	Male	10	147	84%	0.74	0.46	-1.42 [-5.19, 2.34]	77%	0.96	0.34	-0.96 [-2.93, 1.00]	MD/Random

	Female	10	143	64%	0.62	0.54	-0.75 [-3.13, 1.63]					
	Male	4	66	0%	0.58	0.56	-0.13 [-0.57, 0.31]					
Knee abduction at IC								0%	1.71	0.09	-0.26 [-0.55, 0.04]	MD/Fixed
	Female	6	89	0%	1.78	0.07	-0.36 [-0.76, 0.04]					
	Male	9	135	0%	1.52	0.13	0.41 [-0.12, 0.95]					
Peak knee abduction								0%	1.25	0.21	0.23 [-0.13, 0.60]	MD/Fixed
	Female	8	118	0%	0.27	0.79	0.07 [-0.44, 0.58]					
	Male	5	68	0%	0.38	0.7	0.48 [-1.98, 2.93]					
Hip flexion at IC								0%	0.21	0.83	0.17 [-1.44, 1.79]	MD/Fixed
	Female	5	65	0%	0.05	0.96	-0.06 [-2.20,					

							2.09]					
**Peak Hip flexion**	**Male**	**5**	**68**	**0%**	**0.36**	**0.72**	**0.63 [-2.83, 4.09]**	**0%**	**0.39**	**0.7**	**-0.53 [-3.20, 2.14]**	**MD/Fixed**
	**Female**	**5**	**65**	**0%**	**1.04**	**0.3**	**-2.21 [-6.39, 1.97]**					
**Hip abduction at IC**	**Male**	**5**	**74**	**0%**	**0.16**	**0.87**	**-0.13 [-1.70, 1.44]**	**0%**	**0.37**	**0.71**	**-0.18 [-1.14, 0.77]**	**MD/Fixed**
	**Female**	**5**	**71**	**0%**	**0.35**	**0.73**	**-0.22 [-1.43, 0.99]**					
**Peak Hip abduction**	**Male**	**4**	**53**	**0%**	**0.19**	**0.85**	**-0.19 [-2.09, 1.72]**	**1%**	**0.2**	**0.84**	**-0.14 [-1.48, 1.20]**	**MD/Random**
	**Female**	**4**	**50**	**47%**	**0.21**	**0.83**	**0.29 [-2.36, 2.94]**					

**Hip ER at IC**	**Male**	**3**	**45**	**0%**	**3.17**	**0.002***	**1.35 [0.51, 2.18]**	**0%**	**4.31**	**<0.001***	**1.27 [0.69, 1.85]**	**MD/Fixed**
	**Female**	**3**	**45**	**0%**	**2.93**	**0.003***	**1.20 [0.40, 2.01]**					
**Peak Hip IR**	**Male**	**4**	**58**	**51%**	**0.98**	**0.33**	**-1.26 [-3.77, 1.25]**	**41%**	**1.15**	**0.25**	**-1.10 [-2.96, 0.77]**	**MD/Random**
	**Female**	**3**	**37**	**0%**	**0.03**	**0.97**	**-0.05 [-2.79, 2.70]**					
**Peak Ankle dorsiflexion**	**Male**	**3**	**35**	**0%**	**1.04**	**0.3**	**-1.61 [-4.64, 1.41]**	**0%**	**1.7**	**0.09**	**-1.69 [-3.64, 0.26]**	**MD/Fixed**
	**Female**	**3**	**33**	**0%**	**1.35**	**0.18**	**-1.75 [-4.29, 0.79]**					

**Kinetics Variables**												
**Peak Hip IR moment**	**Male**	**3**	**43**	**61%**	**0.97**	**0.33**	**-0.35 [-1.05, 0.35]**	**43%**	**1.95**	**0.05**	**-0.41 [-0.83, 0.00]**	**SMD/Random**
	**Female**	**3**	**42**	**45%**	**1.48**	**0.14**	**-0.45 [-1.06, 0.15]**					
**Peak Hip flexion moment**	**Male**	**2**	**23**	**26%**	**0.83**	**0.41**	**0.29 [-0.40, 0.98]**	**0%**	**1.57**	**0.12**	**0.34 [-0.08, 0.76]**	**SMD/Random**
	**Female**	**2**	**22**	**18%**	**1.18**	**0.24**	**0.40 [-0.26, 1.07]**					
**Peak hip extension moment**	**Male**	**2**	**25**	**0%**	**2.01**	**0.04***	**0.58 [0.01, 1.15]**	**0%**	**2.43**	**0.01***	**0.51 [0.10, 0.92]**	**SMD/Fixed**

	Female	2	23	0%	1.43	0.15	0.43 [-0.16, 1.01]					
Peak hip adduction moment	Male	3	43	65%	0.8	0.43	0.30 [-0.44, 1.05]	44%	1.59	0.11	0.34 [-0.08, 0.75]	SMD/Random
	Female	3	43	37%	1.23	0.22	0.35 [-0.21, 0.91]					
Peak knee extension moment	Male	4	55	29%	3.15	0.002*	0.76 [0.29, 1.23]	4%	4.07	<0.001*	0.64 [0.33, 0.95]	SMD/Random
	Female	3	36	0%	1.99	0.05	0.48 [0.01, 0.95]					
Peak Knee abduction moment	Male	4	59	51%	0.26	0.79	-0.07 [-0.60, 0.46]	73%	0.71	0.48	-0.19 [-0.72, 0.34]	SMD/Random
	Female	4	56	85%	0.65	0.52	-0.34 [-1.39,					

							0.70]					
Peak VGRF	Male	7	158	63%	0.09	0.93	0.02 [-0.39, 0.42]	54%	0.43	0.67	0.06 [-0.20, 0.31]	SMD/Random
	Female	7	136	49%	0.53	0.59	0.10 [-0.26, 0.45]					

Most forest plots are in Appendix D. IC:Initial contact; IR:internal rotation; ER:external rotation; VGRF:Peak vertical ground reaction force; MD:Mean Difference; SMD:Standardization Mean Difference; Moment (Nm/kg or N m/kg·m or N·m/kg of BW*H or N/kg). Peak vertical ground-reaction force (BW or %BW or N or N/kg). BW, body weight; H, height;*:P<0.05

Knee Kinematics

The meta-analysis focused on changes in knee flexion and abduction at initial contact, as well as peak flexion and abduction, before and after fatigue (Fig.3). Key findings include:

1.Knee flexion angle at initial contact: Ten studies with 378 participants showed a total effect size of -0.72 (95% CI: -1.28, -0.17). Males exhibited a significant increase in angle (effect size: -1.23, 95% CI: -1.85, -0.61), while females showed an increasing trend that was not statistically significant (effect size: -0.25, 95% CI: -1.22, 0.72).

2.Peak knee flexion angle: Fatigue did not significantly affect peak flexion angle for both males and females (total effect size: -0.96, 95% CI: -2.93, 1.00; males: -1.42, 95% CI: -5.19, 2.34; females: -0.75, 95% CI: -3.13, 1.63).

3.Knee abduction angle at initial contact: The total effect size was -0.26 (95% CI: -0.55, 0.04), with both genders showing a non-significant increasing trend post-fatigue (males: -0.13, 95% CI: -0.57, 0.31; females: -0.36, 95% CI: -0.76, 0.04).

4.Peak knee abduction angle: Changes in peak abduction angle were not statistically significant for both genders (total effect size: 0.23, 95% CI: -0.13, 0.60; males: 0.41, 95% CI: -0.12, 0.95; females: 0.07, 95% CI: -0.44, 0.58).

These results indicate that although some knee joint biomechanical parameters changed under certain conditions, most results were not statistically significant.

Figure.3 The forest plot of the effects of fatigue on knee joint kinematics and gender differences

Hip Kinematics

The meta-analysis of hip biomechanics before and after fatigue covered six outcome measures: hip flexion and abduction at initial contact, peak flexion and abduction, and external and internal rotation movements(Fig.4). Key findings include:

1. Hip flexion angle at initial contact: Males (5 studies, N=68) had an effect size of 0.48 (95% CI: -1.98, 2.93), and females (5 studies, N=65) had an effect size of -0.06 (95% CI: -2.20, 2.09), showing no significant changes.

2. Peak hip flexion angle: Males (5 studies, N=68) had an effect size of 0.63 (95% CI: -2.83, 4.09), and females (5 studies, N=65) had an effect size of -2.21 (95% CI: -6.39, 1.97), showing no significant changes, though females trended towards increased angles post-fatigue.

3. Hip abduction angle at initial contact: Males (5 studies, N=74) had an effect size of -0.13 (95% CI: -1.70, 1.44), and females (5 studies, N=71) had an effect size of -0.22 (95% CI: -1.43, 0.99), showing no significant changes.

4. Peak hip abduction angle: Males (4 studies, N=53) had an effect size of -0.19 (95% CI: -2.09, 1.72), and females (4 studies, N=50) had an effect size of 0.29 (95% CI: -2.36, 2.94), showing no significant changes.

5. Hip external rotation angle at initial contact: Both genders showed significant positive changes, with males (3 studies, N=45) having an effect size of 1.35 (95% CI: 0.51, 2.18) and females (3 studies, N=45) having an effect size of 1.20 (95% CI: 0.40, 2.01), indicating a trend of angle reduction post-fatigue.

6. Peak hip internal rotation angle: Males (4 studies, N=58) had an effect size of -1.26 (95% CI: -3.77, 1.25), and females (3 studies, N=37) had an effect size of -0.05 (95% CI: -2.79, 2.70), showing no significant changes, although males trended towards increased angles.

Overall, while some changes were observed, most results regarding hip flexion and abduction were not statistically significant, with only hip external rotation at initial contact showing significant changes in both genders.

Figure.4 The forest plot of the effects of fatigue on hip joint kinematics and gender differences

Ankle Kinematics

In this meta -analysis, changes in peak ankle dorsiflexion angle before and after fatigue were evaluated across genders(Fig.5). The overall effect size was -1.69 (95% CI: -3.64, 0.26). For males (3 studies, N=35), the effect size was -1.61 (95% CI: -4.64, 1.41), and for females (3 studies, N=33), it was -1.75 (95% CI: -4.29, 0.79), both showing no significant changes, and heterogeneity (I^2) was 0% for both. These results suggest that fatigue may lead to an increase in ankle dorsiflexion angle and indicate no significant difference in changes between males and females under fatigue, with the changes not significantly influenced by sample heterogeneity.

Figure.5 The forest plot of the effects of fatigue on ankle joint kinematics and gender differences

Kinetics Data of Landing Biomechanics

The dynamic data for the hip and knee joints, as well as peak vertical ground reaction force (PVGRF),

were evaluated.

Knee Kinetics

This meta-analysis examined the effects of fatigue on knee extension and abduction torques, considering gender differences(Fig.6). For Peak knee extension moment, males (N=55) exhibited an effect size of 0.76 (95% CI: 0.29, 1.23, P=0.002), indicating a significant change, while females (N=36) had an effect size of 0.48 (95% CI: 0.01, 0.95, P=0.05), which was nearly significant. The overall effect size was 0.64 (95% CI: 0.33, 0.95, P<0.001), demonstrating a significant change. Heterogeneity was 29% for males, 0% for females, and 4% overall, indicating a significant reduction in knee extension torque due to fatigue.

For Peak Knee abduction moment, males (N=59) showed an effect size of -0.07 (95% CI: -0.60, 0.46, P=0.79), and females (N=56) had an effect size of -0.34 (95% CI: -1.39, 0.70, P=0.52). The overall effect size was -0.19 (95% CI: -0.72, 0.34, P=0.48), with none showing significant changes. Heterogeneity was 51% for males, 85% for females, and 73% overall. These results suggest a statistically significant effect of fatigue on knee extension moment in males, but not on abduction torque for either gender, with high heterogeneity observed.

Figure.6 The forest plot of the effects of fatigue on knee joint kinetics and gender differences

Hip Kinetics

This meta-analysis assessed changes in hip internal rotation, flexion, extension, and adduction torques before and after fatigue across genders(Fig.7). For peak hip internal rotation moment, males (3 studies, N=43) had an effect size of -0.35 (95% CI: -1.05, 0.35, P=0.33), and females (3 studies, N=42) had -0.45 (95% CI: -1.06, 0.15, P=0.14). The overall effect size was -0.41 (95% CI: -0.83, 0.00, P=0.05), with no significant

changes. For peak hip flexion moment, males (2 studies, N=23) had an effect size of 0.29 (95% CI: -0.40, 0.98, P=0.41), and females (2 studies, N=22) had 0.40 (95% CI: -0.26, 1.07, P=0.24). The overall effect size was 0.34 (95% CI: -0.08, 0.76, P=0.12), with no significant changes. For peak hip extension moment, males (2 studies, N=25) had an effect size of 0.58 (95% CI: 0.01, 1.15, P=0.04), and females (2 studies, N=23) had 0.43 (95% CI: -0.16, 1.01, P=0.15). The overall effect size was 0.51 (95% CI: 0.10, 0.92, P=0.01), showing significant changes in males and the overall analysis, indicating a decrease. For peak hip adduction moment, males (3 studies, N=43) had an effect size of 0.30 (95% CI: -0.44, 1.05, P=0.43), and females (3 studies, N=43) had 0.35 (95% CI: -0.21, 0.91, P=0.22). The overall effect size was 0.34 (95% CI: -0.08, 0.75, P=0.11), with no significant changes. These findings highlight the potential impact of fatigue on different hip joint moment, with significant changes particularly in male hip extension moment.

Figure.7 The forest plot of the effects of fatigue on hip joint kinetics and gender differences

Peak VGRF

The meta-analysis of peak vertical ground reaction force (PVGRF) examined gender differences in biomechanical responses to fatigue(Fig.8). The effect size for males (7 studies, N=158) was 0.02 (95% CI: -0.39, 0.42, P=0.93), and for females (7 studies, N=136), it was 0.10 (95% CI: -0.26, 0.45, P=0.59), neither indicating significant changes. The overall effect size was 0.06 (95% CI: -0.20, 0.31, P=0.67), also showing no significant changes. Heterogeneity was 63% for males, 49% for females, and 54% overall. These results indicate no significant differences in peak vertical ground reaction force between genders before and after fatigue, with moderate heterogeneity. The findings suggest that fatigue does not significantly impact peak

vertical ground reaction force across genders, and variability among study samples may affect result consistency.

Figure.8 The forest plot of the effects of fatigue on vertical ground reaction force and gender differences

Discussion

This meta-analysis systematically reviewed existing literature to understand how fatigue influences gender differences in landing biomechanics and the associated risk of ACL injuries. The results indicate that fatigue significantly affects the kinematics and kinetics of the knee, hip, and ankle joints. However, most gender differences in these effects were not statistically significant, with only a few variables showing notable changes. Specifically, fatigue significantly increased the knee flexion angle at initial contact in males but not in females. Additionally, fatigue led to a significant reduction in hip external rotation angles at initial contact for both genders. In terms of kinetics, fatigue significantly decreased the maximum extension torque in the knee and hip joints of males, while the effect in females was nearly significant. This discussion will delve into our key findings, focusing on the landing biomechanics of the knee, hip, and ankle.

In the sagittal plane biomechanics, our study found that males had a greater knee flexion angle at initial contact compared to females, both before and after fatigue. Under fatigue, both genders showed an increase in knee flexion angle, but the increase was more significant in males. For peak knee flexion angles, both genders showed an increasing trend post-fatigue, which is considered a favorable strategy to reduce ACL loading(47). However, Decker et al. noted that in a non-fatigued state, females exhibited greater knee flexion angles during landing compared to males(8). This suggests that while fatigue makes male and female knee flexion angles more similar, females might apply different mechanical loads on the knee joint under normal conditions due to their greater flexion angles. This gender difference may be attributed to higher flexibility(48) and different muscle strength distributions(49) in females, affecting their landing mechanics

and potential injury risks. Our study showed that males generally had smaller peak knee flexion angles before fatigue compared to females, likely due to greater overall muscle strength, which enhances knee stability and allows earlier stabilization. The hip joint, as a proximal joint, plays a critical role in controlling the knee joint in closed chain actions(50), and landing patterns with higher hip flexion angles are associated with lower injury risk(51, 52). Our results showed no significant difference in the effect of fatigue on hip flexion angles, but the impact on maximum hip flexion angles was greater in females. Matthew N(53) noted that the results of kinetic and kinematic variables under existing fatigue protocols lack consistency, possibly due to individual variability in responses. He observed that fatigued athletes often exhibit greater peak knee and hip flexion angles during landing, along with reduced landing forces. These findings suggest that athletes may adjust their joint movement strategies to adapt to fatigue conditions.

The importance of sagittal plane knee movements lies in their relationship with anterior-posterior knee shear forces. There is considerable debate on whether abnormal sagittal plane dynamics can cause anterior cruciate ligament (ACL) injuries. Chappell et al.(19) noted that increased anterior tibial shear forces during stop-jumping were associated with increased knee flexion angles. Our study found that the reduction in knee extension torque post-fatigue was more significant in males, with an effect size of 0.76, compared to 0.48 in females. This may be due to the downward momentum reduction caused by knee flexion during landing, along with eccentric contraction of the surrounding knee muscles(8). The body's ability to adapt and adjust kinematics can help reduce the risk of ACL injuries and protect against landing impacts. However, a study by Schmitz et al. (54)on 90-minute intermittent exercise found that as exercise duration increased, subjects landed in a more upright posture post-fatigue, with less hip and knee flexion. Reduced joint flexion was accompanied by decreased hip work and increased knee shear forces(55). Some studies noted that compared to males, females showed increased quadriceps activation before landing in the

same tasks(56). Krosshaug et al.(57)found that female basketball players exhibited significantly greater knee flexion angles at initial ground contact and within the first 50 milliseconds after contact compared to males, suggesting that females might use different biomechanical strategies during landing to better absorb impact forces. However, increased quadriceps activation during landing preparation might increase ACL loading(58). Regarding hip extension (2 studies) and flexion (2 studies) torques, as well as peak ankle dorsiflexion angles (3 studies), despite being low risk, the limited number of studies reporting these indicators prevents drawing definitive conclusions on potential biomechanical differences in landing mechanics between genders under fatigue.

Frontal plane biomechanics are also related to ACL injuries. Our study found no significant differences in knee and hip abduction angles at initial contact and peak values. However, under fatigue, females showed a greater trend of increased knee abduction angles compared to males. Evidence suggests that excessive dynamic knee valgus may increase ACL tear risk(7, 59). Hewett et al.(4) conducted a prospective study on female athletes in high-risk sports (such as soccer, basketball, and volleyball) during jumping and landing tasks. Using three-dimensional motion capture, they measured joint angles to assess neuromuscular control and kinetic methods to measure joint loads (joint torques). Among the participants, 9 athletes sustained ACL injuries. The ACL injury group had a knee abduction angle of 8° during landing, significantly higher than the non-injured group. Additionally, their knee abduction torque was 2.5 times greater, and the ground reaction force was 20% higher. The injured athletes also showed a faster increase in angular velocity, ground reaction force, and joint torque compared to non-injured athletes. Chappell and Yu et al.(60)further showed that during jumping and landing tasks, female athletes exhibited greater valgus torques compared to males. This may be due to differences in muscle activation timing between genders. Females' lateral thigh muscles (such as the lateral quadriceps and hamstrings) activate later, while the

activation timing of the medial thigh muscles is similar to that of males. In contrast, males' quadriceps are activated simultaneously, with the lateral hamstrings activating earlier than the medial hamstrings(23, 61, 62). Different muscle activation strategies may affect knee abduction and explain the increased initial and peak knee abduction angles in females. It is worth noting that compared to males, females generally have a larger Q angle, which refers to the relationship of the femoral axis relative to the tibial axis in the frontal plane of the knee joint and is often associated with increased knee valgus(63, 64). However, no significant relationship has been found between the Q angle and ACL injury rates in female athletes.

Horizontal plane biomechanics primarily focus on the movement mechanisms of the hip and knee joints. However, this study only included some hip joint mechanical indicators. We found that under fatigue, the hip external rotation angle at initial contact significantly decreased in both male and female athletes. This change could increase the load on the knee and ankle joints, thereby raising the risk of ACL injuries. Koga et al.'s study (65)using a 3D MBIM system found significant differences in hip joint positions between ACL injury cases during actual competitions and non-injury conditions, as well as during side-cutting and landing movements. In injury cases, the hip joint typically showed a smooth flexion transition after ground contact, whereas ACL injuries were characterized by significant hip internal rotation, causing internal rotation of the knee and ankle joints. This internal rotation increased the load on the knee joint, raising the risk of ACL injuries.

Additionally, Koga et al.'s research(65)indicated that during side-cutting and landing movements, hip internal rotation affects not only the hip joint itself but also negatively impacts the entire lower limb kinetic chain, increasing stress and injury risks to other joints. This suggests that future research on ACL injury

mechanisms should focus more on the dynamic performance of the hip joint and its coordination with other lower limb joints.

Conclusion

This study employed a systematic review and meta-analysis to investigate the effects of fatigue and gender differences on lower limb landing biomechanics and their relationship with ACL injury risk. While most results did not show significant changes, some important trends and significant findings were identified.

First, fatigue significantly increased the knee flexion angle at initial contact in males, which helps reduce ACL loading. Second, males showed a significant reduction in knee extension torque after fatigue, potentially affecting knee stability and increasing injury risk. Additionally, fatigue significantly decreased the hip external rotation angle at initial contact in both males and females, which may increase the load on the knee and ankle joints, raising the risk of injury. However, other hip torques (internal rotation, flexion, and adduction) did not show significant changes under fatigue. Finally, the ankle dorsiflexion angle and peak vertical ground reaction force (VGRF) did not show significant changes before and after fatigue, indicating limited effects of fatigue on these parameters.

Overall, fatigue significantly affects certain knee and hip joint parameters, especially in males. These findings underscore the importance of considering gender differences in fatigue management and sports strategies to reduce ACL injury risk. Future research should further explore the mechanisms behind these changes and assess gender-specific interventions to mitigate ACL injury risk.

Limitations

There are several limitations in this systematic review that need to be considered. First, although 14 studies were included, only a few reported ankle joint biomechanical variables, limiting the scope of our analysis. Second, the review focused solely on lower limb biomechanical indicators without considering the positioning and control of the trunk and the entire kinetic chain, which may significantly impact knee joint biomechanics. This limitation might result in an incomplete understanding of knee joint biomechanics. Finally, although the review included data from both sexes before and after fatigue, studies focusing on only one gender were not included, potentially affecting the comprehensive understanding of gender differences.

Supporting information

S1 Figure PubMed Literature Selection Strategy.

S2 Table Checklist of 10 criteria based on an adapted version of the STROBE guidelines for assessing the quality of observational studies used in this analysis.

S3 Figure PRISMA flow chart for inclusion and exclusion of studies.

S4 Table Quality scores of studies included in the review.

S5 Table Demographic information from included studies.

S6 Figure The forest plot of the effects of fatigue on knee joint kinematics and gender differences

S7 Figure The forest plot of the effects of fatigue on hip joint kinematics and gender differences

S8 Figure The forest plot of the effects of fatigue on ankle joint kinematics and gender differences

S9 Figure The forest plot of the effects of fatigue on knee joint kinetics and gender differences

S10Figure The forest plot of the effects of fatigue on hip joint kinetics and gender differences

S11 Table Summary of a meta-analysis evaluating the effects of fatigue before and after (including gender subgroups) and on major biomechanical variables of lower limb landing mechanics

Acknowledgments

Thank you to all the researchers who have contributed to this study.

Author contributions

C. L. was responsible for selecting search terms, screening the literature, evaluating article quality, extracting study characteristics, and contributing significantly to manuscript writing. W.P. provided final confirmation of data extraction and verified the search criteria and data extraction process. W.Q. proofread the language. Z.Z. performed data analysis, translated the manuscript, and proofread the text. J.H. also

conducted data analysis, translated the manuscript, and proofread the text. X.D. drafted, revised, and finalized the manuscript. B.C. was responsible for drafting, revising, and confirming the final version of the manuscript. All authors have read and approved the final version of the manuscript for publication.

References

1. Hootman JM, Dick R, Agel JJJoat. Epidemiology of collegiate injuries for 15 sports: summary and recommendations for injury prevention initiatives. 2007;42(2):311.
2. Yu B, Garrett WEJBjosm. Mechanisms of non-contact ACL injuries. 2007;41(suppl 1):i47-i51.
3. Boden BP, Dean GS, Feagin JA, Garrett WEJO. Mechanisms of anterior cruciate ligament injury. SLACK Incorporated Thorofare, NJ; 2000. p. 573-8.
4. Hewett TE, Myer GD, Ford KR, Heidt Jr RS, Colosimo AJ, McLean SG, et al. Biomechanical measures of neuromuscular control and valgus loading of the knee predict anterior cruciate ligament injury risk in female athletes: a prospective study. 2005;33(4):492-501.
5. Wetters N, Weber AE, Wuerz TH, Schub DL, Mandelbaum BRJOTiSM. Mechanism of injury and risk factors for anterior cruciate ligament injury. 2016;24(1):2-6.
6. Donelon TA, Dos' Santos T, Pitchers G, Brown M, Jones PAJSM-O. Biomechanical determinants of knee joint loads associated with increased anterior cruciate ligament loading during cutting: a systematic review and technical framework. 2020;6:1-21.
7. Paterno MV, Schmitt LC, Ford KR, Rauh MJ, Myer GD, Huang B, et al. Biomechanical measures during landing and postural stability predict second anterior cruciate ligament injury after anterior cruciate ligament reconstruction and return to sport. 2010;38(10):1968-78.
8. Decker MJ, Torry MR, Wyland DJ, Sterett WI, Steadman JRJCb. Gender differences in lower extremity kinematics, kinetics and energy absorption during landing. 2003;18(7):662-9.
9. Hertel J, Dorfman JH, Braham RAJJoss, medicine. Lower extremity malalignments and anterior cruciate ligament injury history. 2004;3(4):220.
10. Slauterbeck JR, Fuzie SF, Smith MP, Clark RJ, Xu KT, Starch DW, et al. The menstrual cycle, sex hormones, and anterior cruciate ligament injury. 2002;37(3):275.
11. Fousekis K, Tsepis E, Vagenas G. Intrinsic Risk Factors of Noncontact Ankle Sprains in Soccer A Prospective Study on 100 Professional Players. American Journal of Sports Medicine. 2012;40(8):1842-50.
12. Enoka RM, Duchateau JJTJop. Muscle fatigue: what, why and how it influences muscle function. 2008;586(1):11-23.
13. Fitts RHJTAjosm. Muscle fatigue: the cellular aspects. 1996;24(6_suppl):S9-S13.
14. Lattanzio P-J, Petrella RJJO. Knee proprioception: a review of mechanisms, measurements, and implications of muscular fatigue. 1998;21(4):463-71.
15. Miura K, Ishibashi Y, Tsuda E, Okamura Y, Otsuka H, Toh SJATJoA, et al. The effect of local and general fatigue on knee proprioception. 2004;20(4):414-8.

16. Rodacki AL, Fowler NE, Bennett SJJM, Sports Si, Exercise. Multi-segment coordination: fatigue effects. 2001;33(7):1157-67.

17. Wojtys EM, Wylie BB, Huston LJJTAjosm. The effects of muscle fatigue on neuromuscular function and anterior tibial translation in healthy knees. 1996;24(5):615-21.

18. Liederbach M, Dilgen F, Rose D. Effect of fatigue on balance in dancers with and without anterior cruciate ligament reconstruction. Abstract published in the proceedings booklet of the American Physical Therapy Association Annual Meeting, Orlando, Florida, June 2006.

19. Chappell JD, Herman DC, Knight BS, Kirkendall DT, Garrett WE, Yu B. Effect of fatigue on knee kinetics and kinematics in stop-jump tasks. American journal of sports medicine. 2005;33(7):1022 - 9.

20. Borotikar BS, Newcomer R, Koppes R, McLean SGJCb. Combined effects of fatigue and decision making on female lower limb landing postures: central and peripheral contributions to ACL injury risk. 2008;23(1):81-92.

21. Madigan ML, Pidcoe PEJJoE, Kinesiology. Changes in landing biomechanics during a fatiguing landing activity. 2003;13(5):491-8.

22. Price R, Hawkins R, Hulse M, Hodson AJBjosm. The Football Association medical research programme: an audit of injuries in academy youth football. 2004;38(4):466-71.

23. Ford KR, Myer GD, Hewett TEJM, Sports Si, Exercise. Valgus knee motion during landing in high school female and male basketball players. 2003;35(10):1745-50.

24. Fagenbaum R, Darling WG. Jump landing strategies in male and female college athletes and the implications of such strategies for anterior cruciate ligament injury. Am J Sports Med. 2003;31(2):233-40.

25. Rozzi SL, Lephart SM, Fu FHJJoat. Effects of muscular fatigue on knee joint laxity and neuromuscular characteristics of male and female athletes. 1999;34(2):106.

26. Moher D, Liberati A, Tetzlaff J, Altman DG, surgery PGJIjo. Preferred reporting items for systematic reviews and meta-analyses: the PRISMA statement. 2010;8(5):336-41.

27. Amir-Behghadami M, Janati AJEMJ. Population, Intervention, Comparison, Outcomes and Study (PICOS) design as a framework to formulate eligibility criteria in systematic reviews. 2020.

28. Von Elm E, Altman DG, Egger M, Pocock SJ, Gøtzsche PC, Vandenbroucke JPJTl. The Strengthening the Reporting of Observational Studies in Epidemiology (STROBE) statement: guidelines for reporting observational studies. 2007;370(9596):1453-7.

29. Holden S, Boreham C, Delahunt EJSm. Sex differences in landing biomechanics and postural stability during adolescence: a systematic review with meta-analyses. 2016;46:241-53.

30. Friedrich JO, Adhikari NK, Beyene JBMRM. The ratio of means method as an alternative to mean differences for analyzing continuous outcome variables in meta-analysis: a simulation study. 2008;8:1-15.

31. Higgins JP, Thompson SG, Deeks JJ, Altman DGJB. Measuring inconsistency in meta-analyses. 2003;327(7414):557-60.

32. Hedges LV, Vevea JLJPm. Fixed-and random-effects models in meta-analysis. 1998;3(4):486.

33. Thomas AC, McLean SG, Palmieri-Smith RM. Quadriceps and hamstrings fatigue alters hip and knee mechanics. J Appl Biomech. 2010;26(2):159-70.

34. Benjaminse A, Habu A, Sell TC, Abt JP, Fu FH, Myers JB, et al. Fatigue alters lower extremity kinematics during a single-leg stop-jump task. 2008;16:400-7.

35. Brazen DM, Todd MK, Ambegaonkar JP, Wunderlich R, Peterson C. The effect of fatigue on landing biomechanics in single-leg drop landings. Clin J Sport Med. 2010;20(4):286-92.

36. Bell DR, Pennuto AP, Trigsted SM. THE EFFECT OF EXERTION AND SEX ON VERTICAL GROUND REACTION FORCE VARIABLES AND LANDING MECHANICS. Journal of Strength and Conditioning Research. 2016;30(6):1661-9.

37. Gehring D, Melnyk M, Gollhofer A. Gender and fatigue have influence on knee joint control strategies during landing. Clin Biomech (Bristol, Avon). 2009;24(1):82-7.

38. Pappas E, Hagins M, Sheikhzadeh A, Nordin M, Rose DJNAjosptN. Peak biomechanical variables during bilateral drop landings: comparisons between sex (female/male) and fatigue (pre-fatigue/post-fatigue). 2009;4(2):83.

39. Briem K, Jonsdottir KV, Arnason A, Sveinsson T. Effects of Sex and Fatigue on Biomechanical Measures During the Drop-Jump Task in Children. Orthopaedic journal of sports medicine. 2017;5(1).

40. Lessi GC, Dos Santos AF, Batista LF, de Oliveira GC, Serrão FV. Effects of fatigue on lower limb, pelvis and trunk kinematics and muscle activation: Gender differences. J Electromyogr Kinesiol. 2017;32:9-14.

41. Liederbach M, Kremenic IJ, Orishimo KF, Pappas E, Hagins M. Comparison of landing biomechanics between male and female dancers and athletes, part 2: Influence of fatigue and implications for anterior cruciate ligament injury. Am J Sports Med. 2014;42(5):1089-95.

42. Smith MP, Sizer PS, James CR. Effects of fatigue on frontal plane knee motion, muscle activity, and ground reaction forces in men and women during landing. J Sports Sci Med. 2009;8(3):419-27.

43. Haddas R, James CR, Hooper TL. Lower extremity fatigue, sex, and landing performance in a population with recurrent low back pain. J Athl Train. 2015;50(4):378-84.

44. McLean SG, Fellin RE, Suedekum N, Calabrese G, Passerallo A, Joy S. Impact of fatigue on gender-based high-risk landing strategies. Med Sci Sports Exerc. 2007;39(3):502-14.

45. Kernozek TW, Torry MR, Iwasaki M. Gender differences in lower extremity landing mechanics caused by neuromuscular fatigue. Am J Sports Med. 2008;36(3):554-65.

46. Zhang Q, Ruan M, Singh NB, Huang L, Zhang X, Wu X. Progression of Fatigue Modifies Primary Contributors to Ground Reaction Forces During Drop Landing. J Hum Kinet. 2021;76:161-73.

47. Barber-Westin SD, Noyes FRJTAjosm. Effect of fatigue protocols on lower limb neuromuscular function and implications for anterior cruciate ligament injury prevention training: a systematic review. 2017;45(14):3388-96.

48. Hoge K, Costa PB, Ryan ED, Herda TJ, Walter AA, Beck TW, et al. Gender Differences In Musculotendinous Stiffness And Range Of Motion In College-Aged Men And Women. 2010;24:1.

49. Miller AEJ, MacDougall J, Tarnopolsky M, Sale DJEjoap, physiology o. Gender differences in strength and muscle fiber characteristics. 1993;66:254-62.

50. Al-Hayani AJFm. The functional anatomy of hip abductors. 2009;68(2):98-103.

51. Bathe C, Fennen L, Heering T, Greif A, Dubbeldam RJBOS, Medicine E. Training interventions to reduce the risk of injury to the lower extremity joints during landing movements in adult athletes: a systematic review and meta-analysis. 2023;9(2):e001508.

52. Sugiyama T, Kameda M, Kageyama M, Kiba K, Kanehisa H, Maeda AJJoss, et al. Asymmetry between the dominant and non-dominant legs in the kinematics of the lower extremities during a running single leg jump in collegiate basketball players. 2014;13(4):951.

53. Bourne MN, Webster KE, Hewett TEJSm. Is fatigue a risk factor for anterior cruciate ligament rupture? 2019;49(11):1629-35.

54. Schmitz RJ, Cone JC, Tritsch AJ, Pye ML, Montgomery MM, Henson RA, et al. Changes in drop-jump landing biomechanics during prolonged intermittent exercise. 2014;6(2):128-35.

55. Markolf KL, Burchfield DM, Shapiro MM, Shepard MF, Finerman GA, Slauterbeck JLJJoor. Combined knee loading states that generate high anterior cruciate ligament forces. 1995;13(6):930-5.

56. Yu B, Lin C-F, Garrett WEJCb. Lower extremity biomechanics during the landing of a stop-jump task. 2006;21(3):297-305.

57. Krosshaug T, Nakamae A, Boden BP, Engebretsen L, Smith G, Slauterbeck JR, et al. Mechanisms of anterior cruciate ligament injury in basketball: video analysis of 39 cases. 2007;35(3):359-67.

58. Kaufman KR, An K-N, Litchy W, Chao EJN. Physiological prediction of muscle forces—I. Theoretical formulation. 1991;40(3):781-92.

59. Griffin LY, Agel J, Albohm MJ, Arendt EA, Dick RW, Garrett WE, et al. Noncontact anterior cruciate ligament injuries: risk factors and prevention strategies. 2000;8(3):141-50.

60. Chappell JD, Yu B, Kirkendall DT, Garrett WEJTAjosm. A comparison of knee kinetics between male and female recreational athletes in stop-jump tasks. 2002;30(2):261-7.

61. Ford KR, Myer GD, Toms HE, Hewett TEJMSSE. Gender differences in the kinematics of unanticipated cutting in young athletes. 2005;37(1):124-9.

62. Pappas E, Sheikhzadeh A, Hagins M, Nordin MJJoss, medicine. The effect of gender and fatigue on the biomechanics of bilateral landings from a jump: peak values. 2007;6(1):77.

63. Gray J, Taunton J, McKenzie D, Clement D, McConkey J, Davidson RJIjosm. A survey of injuries to the anterior cruciate ligament of the knee in female basketball players. 1985;6(06):314-6.

64. Meister K, Talley MC, Horodyski MB, Indelicato PA, Hartzel JS, Batts JJTAjoks. Caudal slope of the tibia and its relationship to noncontact injuries to the ACL. 1998;11(4):217-9.

65. Koga H, Nakamae A, Shima Y, Bahr R, Krosshaug TJTAjosm. Hip and ankle kinematics in noncontact anterior cruciate ligament injury situations: video analysis using model-based image matching. 2018;46(2):333-40.

Figure 数字

Click here to access/download;Figure;fig 6.docx

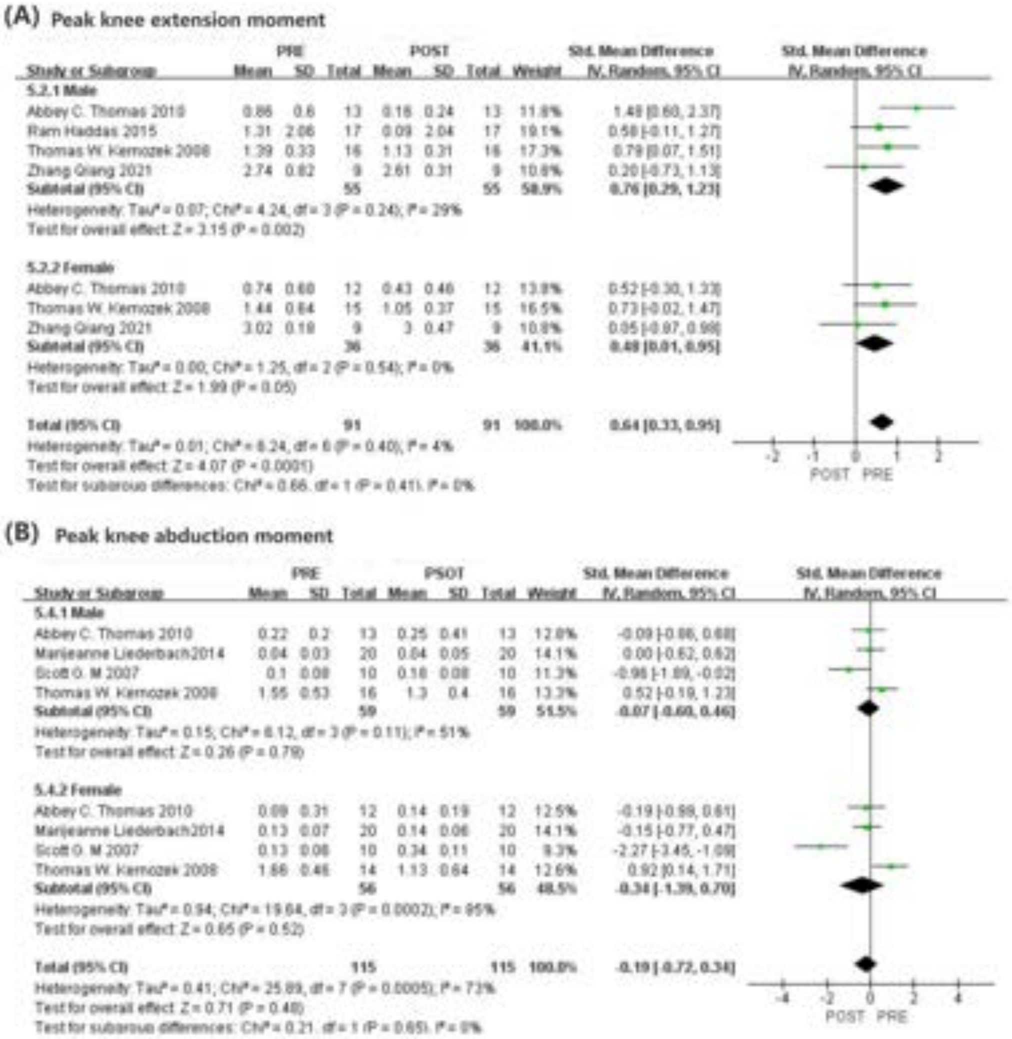

Figure.6 The forest plot of the effects of fatigue on knee joint kinetics and gender differences

Figure 数字 Click here to access/download;Figure;fig1.docx

Scientific Search Queries

#1 "fatigue"[Title/Abstract] OR "tiredness"[Title/Abstract] OR "weariness"[Title/Abstract] OR "exhaustion"[Title/Abstract] OR "induced fatigue"[Title/Abstract] OR "fatigue damage"[Title/Abstract] OR "neuromuscular fatigue"[Title/Abstract]

#2 "sexuality"[Title/Abstract] OR "sex"[Title/Abstract] OR "gender"[Title/Abstract]

#3 "landing biomechanics"[Title/Abstract] OR "kinematics"[Title/Abstract] OR "kinetics"[Title/Abstract] OR "landing"[Title/Abstract] OR "Biomechanics"[Title/Abstract] OR "lower extremity"[Title/Abstract] OR "hip"[Title/Abstract] OR "knee"[Title/Abstract] OR "ankle"[Title/Abstract]

#4 #1 and #2 and #3

Figure1 PubMed Literature Selection Strategy

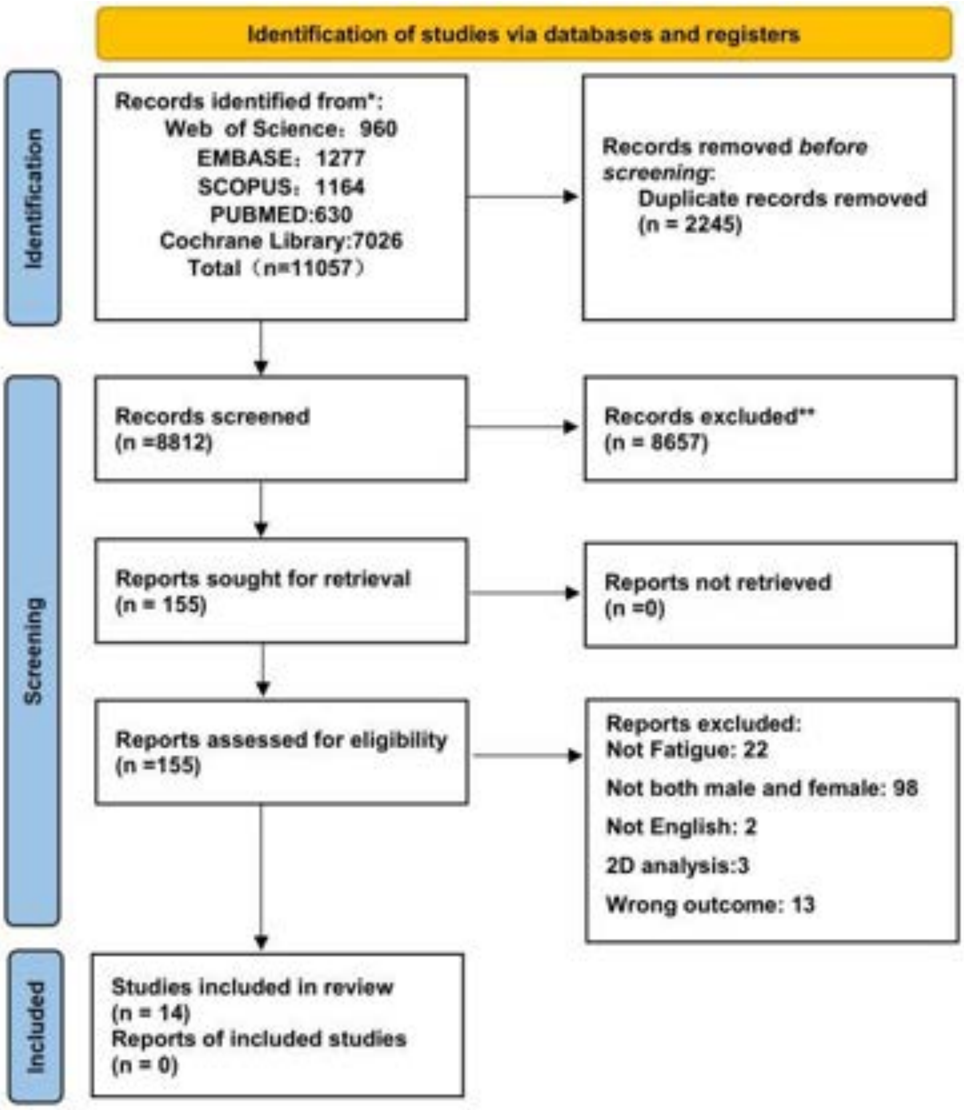

Figure2 PRISMA flow chart for inclusion and exclusion of studies.

Figure 数字 Click here to access/download;Figure;fig3.docx

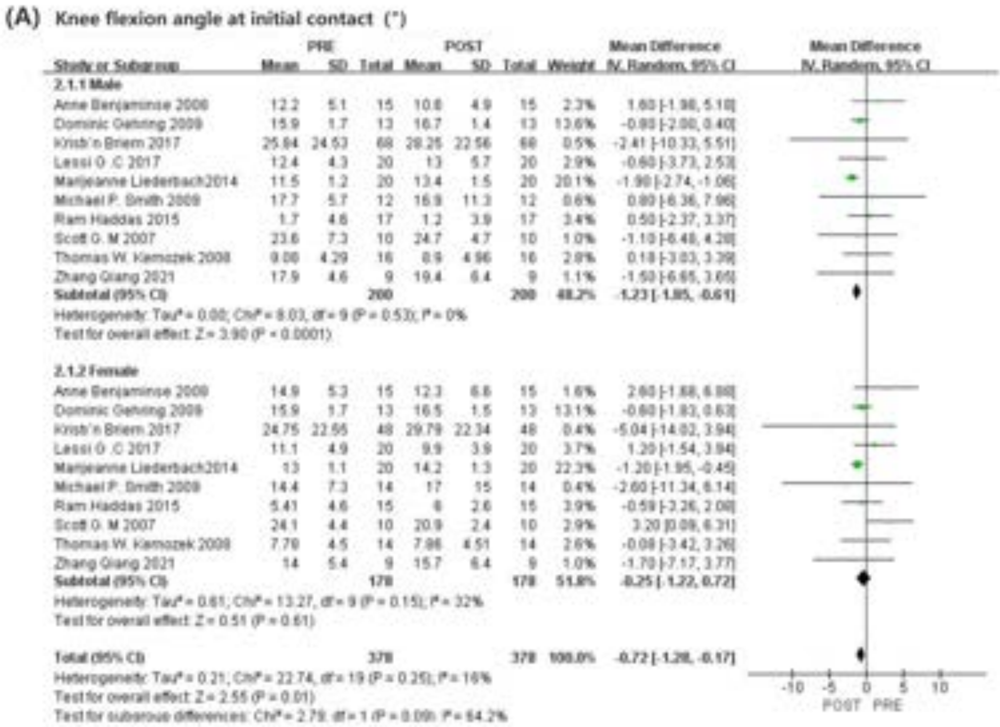

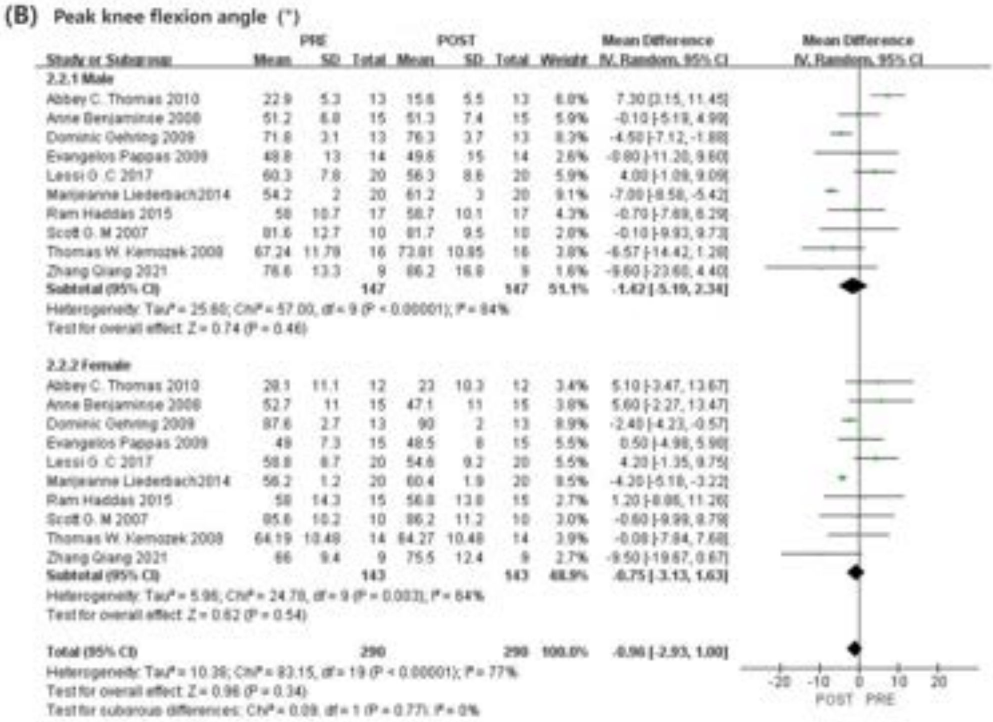

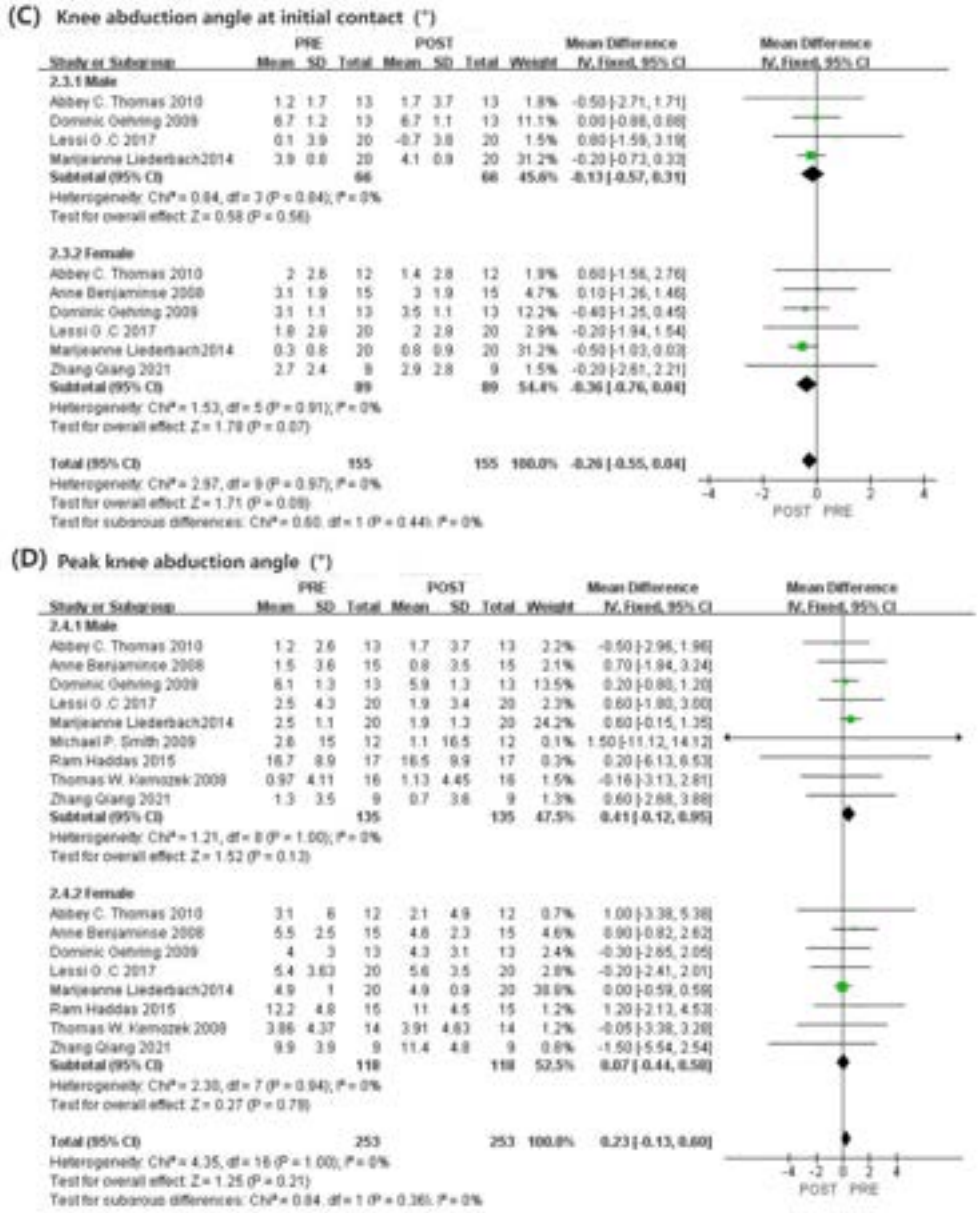

Figure3 The forest plot of the effects of fatigue on knee joint kinematics and gender differences

Figure 数字 Click here to access/download;Figure;fig4.docx

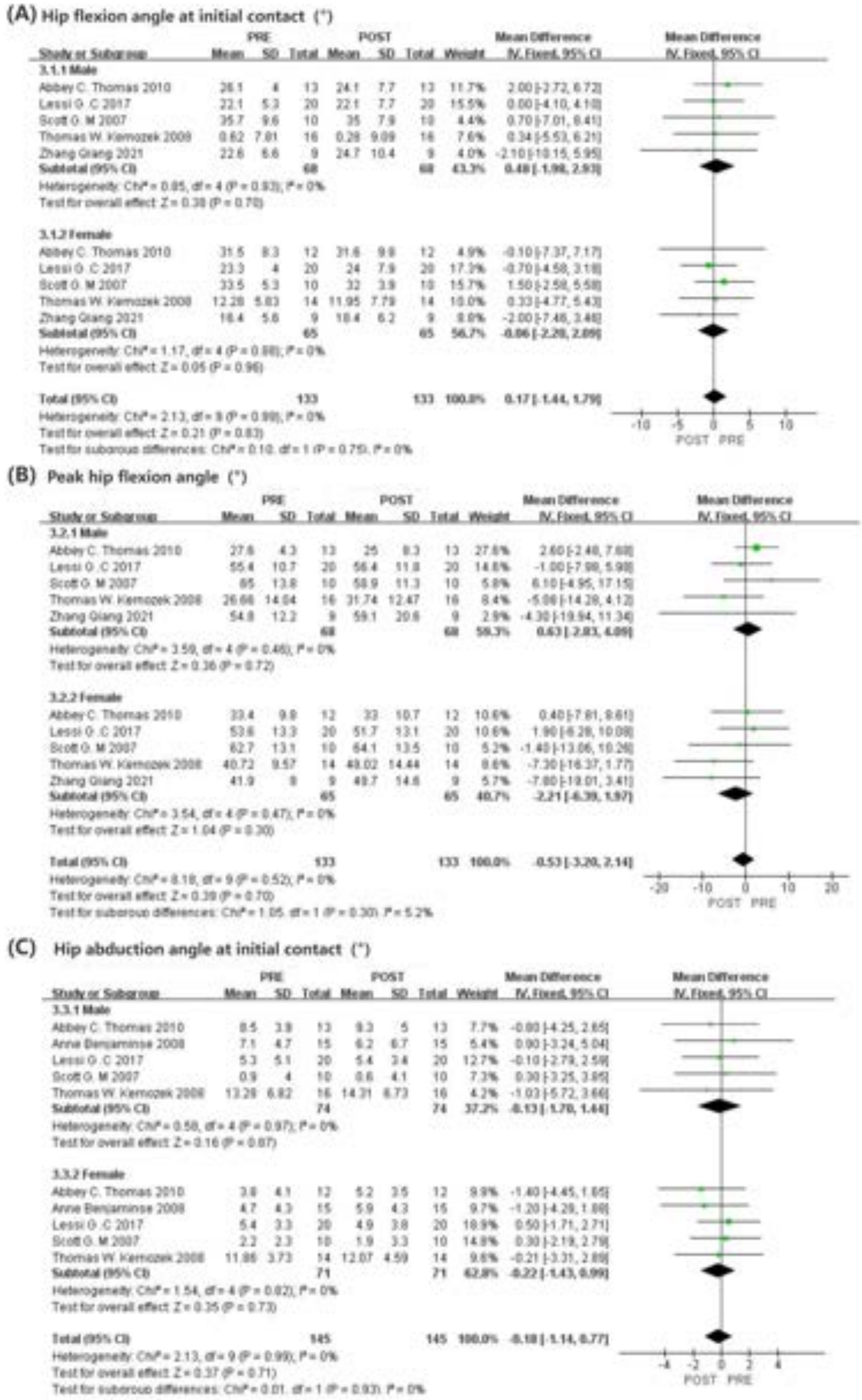

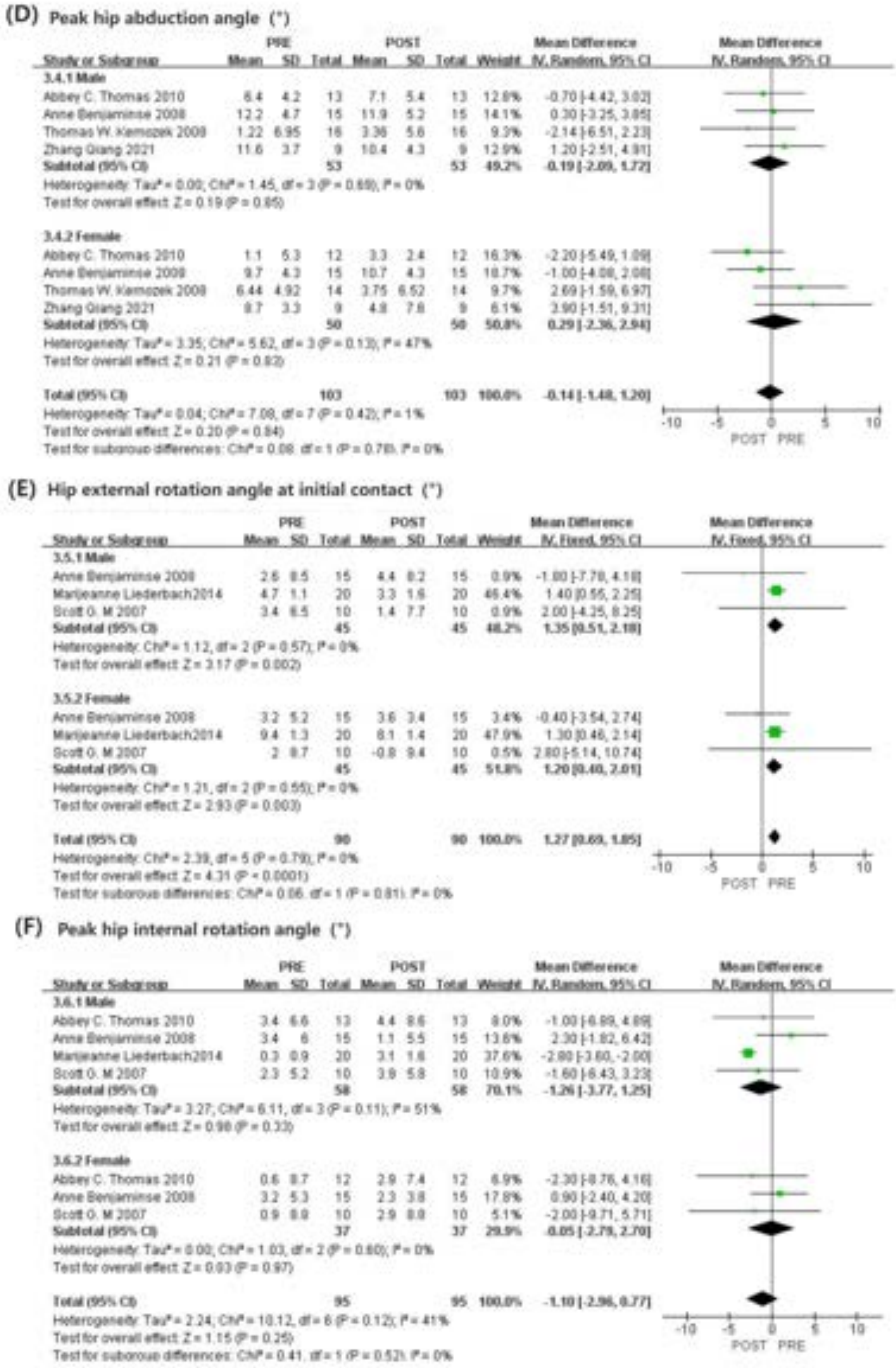

Figure4 The forest plot of the effects of fatigue on hip joint kinematics and gender differences

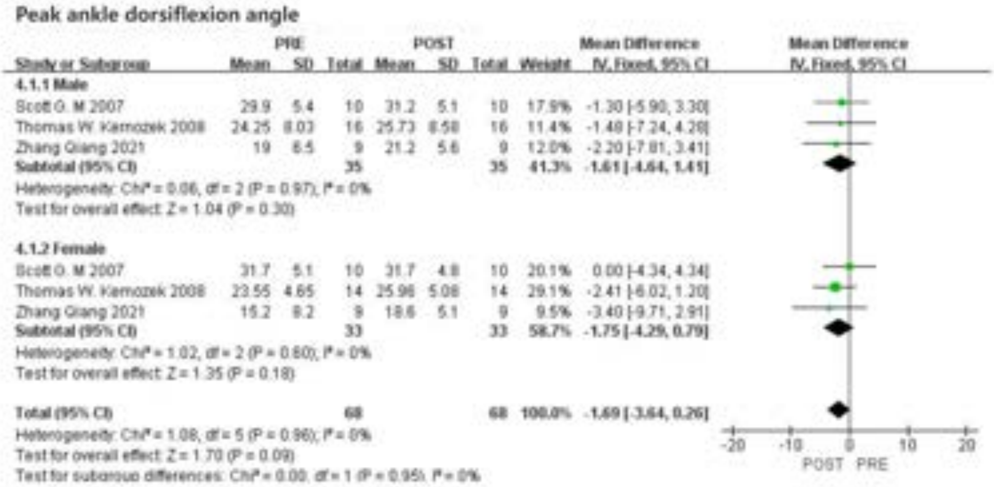

Figure5 The forest plot of the effects of fatigue on ankle joint kinematics and gender differences

Figure 数字 Click here to access/download;Figure;fig7.docx

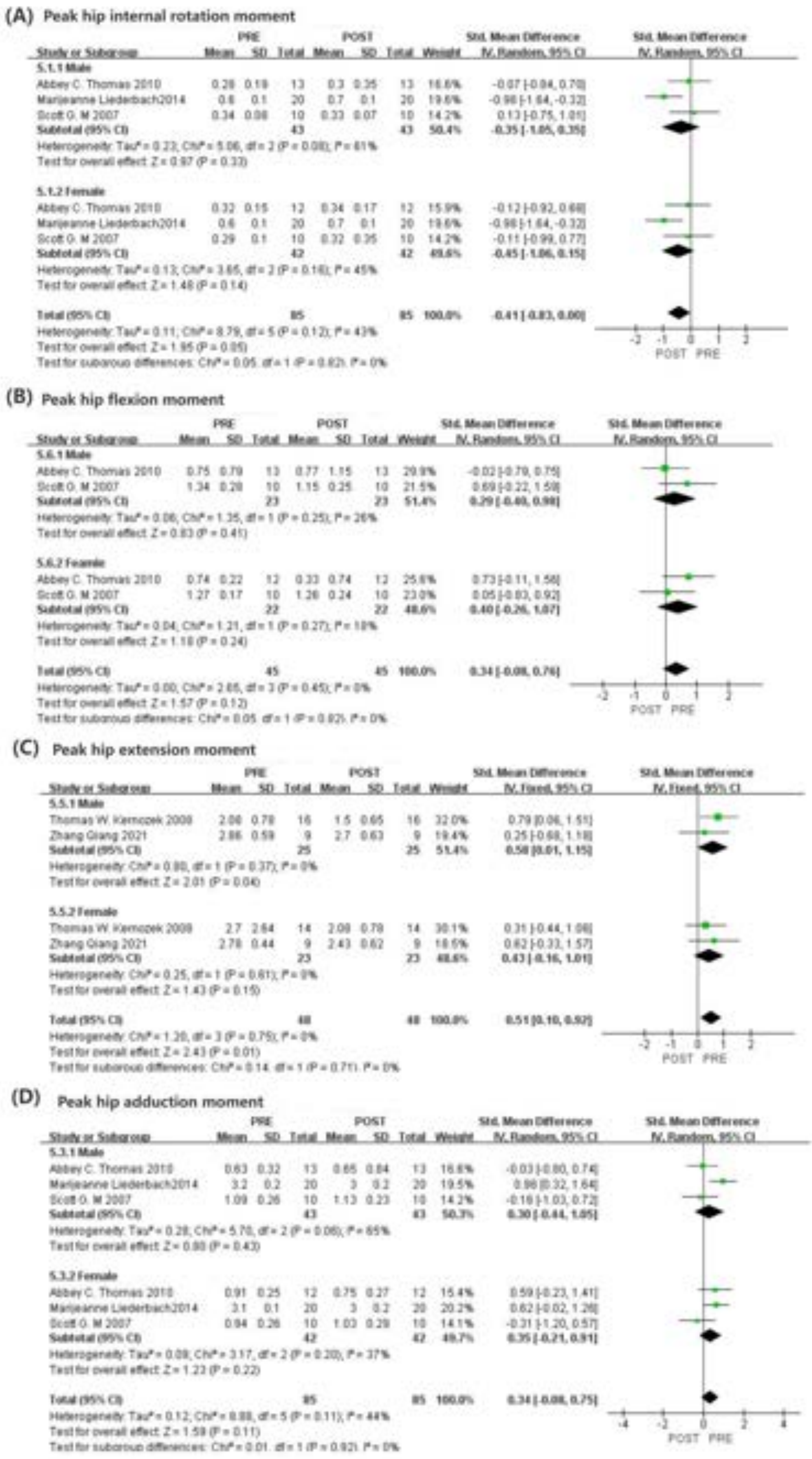

Figure.7 The forest plot of the effects of fatigue on hip joint kinetics and gender differences

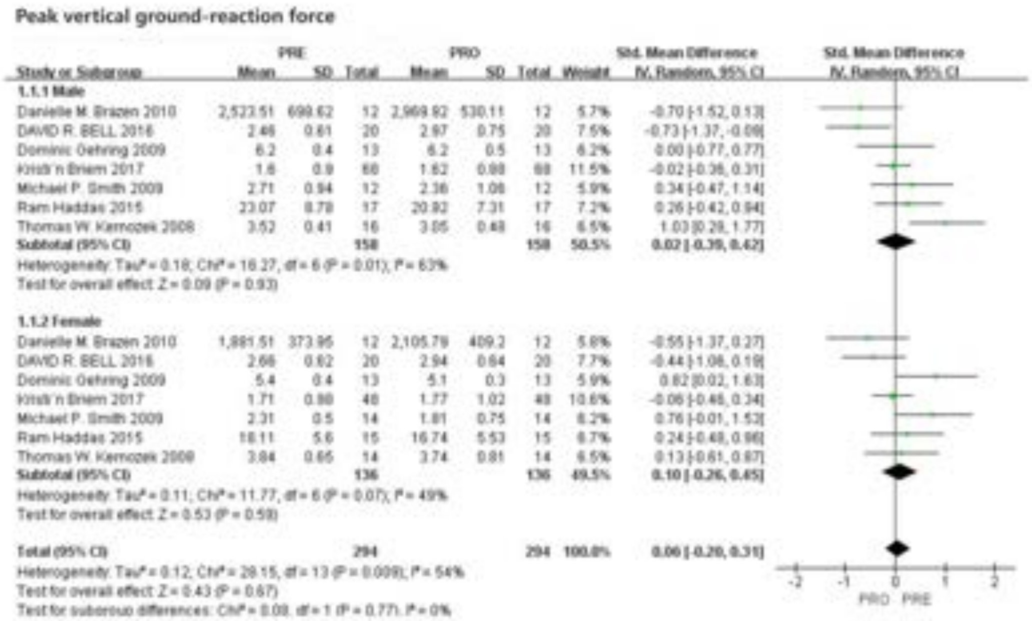

Figure.8 The forest plot of the effects of fatigue on vertical ground reaction force and gender

Click here to access/download
Supporting Information
PRISMA_2020_checklist.docx
